# Dietary patterns and inflammatory bowel disease: a global assessment of eight nutrients by region, gender, and socioeconomic status

**DOI:** 10.3389/fnut.2025.1570634

**Published:** 2025-05-01

**Authors:** Kun He, YuChan Qin, BingBing Li, HongShi Ye, Cun Li, Kouyan Qi, Nenglie Jing, Yuping Yang, Biao Nie

**Affiliations:** ^1^Department of Gastroenterology, The First Affiliated Hospital of Jinan University, Jinan University, Guangzhou, Guangdong, China; ^2^Department of Gastroenterology, Affiliated Hospital of Guangdong Medical University, Guangdong Medical University, Zhanjiang, Guangdong, China

**Keywords:** inflammatory bowel disease, dietary intake, processed meat, global dietary patterns, Bayesian

## Abstract

**Background:**

This study examines global intake patterns of eight dietary components associated with inflammatory bowel disease (IBD): fruits, non-starchy vegetables, processed meats, unprocessed red meats, saturated fats, monounsaturated fatty acids, omega-6 fatty acids, and dietary fiber. Consumption patterns were analyzed across demographic, geographic, and cultural dimensions, including region, sex, urban–rural residence, and educational level. This analysis reveals disparities in dietary intake and provides insights into the links between diet and IBD risk.

**Methods:**

This study uses meta-analysis to evaluate the relationship between inflammatory bowel disease (IBD) and eight dietary components: fruit, non-starchy vegetables, processed meat, unprocessed red meat, dietary fiber, saturated fat, monounsaturated fatty acids, and omega-6 fatty acids. Drawing on data from the Global Dietary Database (GDD), a Bayesian model was employed to estimate intake levels and uncertainties at global and regional scales, incorporating variables such as education, urbanization, and the Socio-Demographic Index (SDI). The analysis covers global intake trends from 1990 to 2018 across 185 countries, and examines the association between educational attainment and IBD-related nutrient consumption from 1900 to 2015 in 145 countries. Instead of relying on traditional hypothesis testing, the study adopts uncertainty intervals (UIs), which provide probabilistic insights into dietary patterns and their variability across populations.

**Results:**

Between 1990 and 2018, processed meat intake showed the largest global increase among the eight dietary components, rising by 26% to 29.1 g/day (95% UI: 25.6–33.1). In Asia, unprocessed red meat intake rose by 38% to 53.5 g/day (95% UI: 42.1–67.6), and processed meat increased by 28% to 21.2 g/day (95% UI: 15.6–27.1). Non-starchy vegetable consumption in Central and Eastern Europe and Central Asia grew by 49%, reaching 182.8 g/day (95% UI: 146.2–228). In high-income countries, unprocessed red meat intake increased by 25% to 32.6 g/day (95% UI: 26.4–40.5). Latin America saw a 45% rise in vegetable intake, reaching 130.2 g/day (95% UI: 113.5–150.1), while the Middle East and North Africa reported a 13% increase to 152.1 g/day (95% UI: 129.8–177.4). South Asia experienced the most rapid relative growth in processed meat consumption (56%), reaching 4.6 g/day (95% UI: 2.4–8.2), although absolute intake remained low. In Sub-Saharan Africa, fruit consumption rose by 15%, to 81.5 g/day (95% UI: 71.3–93.5). These results reveal pronounced regional variation in dietary transitions over the past three decades, underscoring the importance of context-specific strategies to address changing dietary risk factors related to IBD.

**Conclusion:**

This study found that between 1990 and 2018, processed meat intake increased the most across 185 countries, rising by 26%, mirroring the global rise in IBD burden. The intake of eight dietary components showed significant heterogeneity across global populations, with variations by age, education level, and urbanization. These findings may inform policy interventions aimed at reducing intake in high-risk groups with high consumption of dietary factors linked to IBD, particularly in high-income countries and Asia, where IBD burden is increasing rapidly. The sharp rise in processed and unprocessed red meat intake, combined with long-term underconsumption of fruits, vegetables, and dietary fiber, likely contributes significantly to the rising IBD burden.

## Introduction

Inflammatory bowel disease (IBD), which includes Crohn’s disease (CD) and ulcerative colitis (UC), is a chronic inflammatory disorder of the gastrointestinal tract. UC typically begins in the rectum and extends proximally through the colon, while CD can affect any part of the gastrointestinal tract, particularly the terminal ileum, and is characterized by discontinuous lesions. The global prevalence of IBD has risen significantly, increasing by 47.45% since 1990 (1).

Although the etiology and pathogenesis of IBD remain unclear, studies suggest that diet, as a non-genetic factor, influences disease onset and progression by modulating the microbiome (2). Western diets, which are low in fiber, high in processed foods, and rich in fat, are considered key contributors to the rising incidence of IBD, particularly in Western countries (3). In contrast, increased intake of fruits and vegetables appears to have a protective effect (4).

Different dietary components impact IBD onset and progression in various ways. For example, a high-fiber diet may prolong remission in IBD patients (5). Research has shown that diet plays a critical role in regulating gut microbiota and its metabolites: diets high in red meat, sugary desserts, fats, and refined grains worsen gut inflammation, while specific dietary components can alleviate inflammation in IBD animal models (6, 7).

Studies suggest that a high-fiber diet, particularly from vegetables and fruits, may reduce the risk of CD, while a high n-3/n-6 polyunsaturated fatty acid ratio may lower the risk of UC. Conversely, high red meat and omega-6 fatty acid diets are linked to increased IBD risk (8–10).

The global shift toward processed foods and reduced fruit and vegetable consumption, driven by globalization, may partially explain the rising prevalence of IBD. While previous studies have explored the relationship between diet and IBD, there is a lack of systematic analysis of global dietary patterns and long-term trends. This study uses meta-analysis to examine eight key dietary components—fruits, non-starchy vegetables, processed meats, unprocessed red meats, saturated fats, monounsaturated fats, omega-6 fatty acids, and dietary fiber—assessing global intake patterns and trends from 1990 to 2018, and analyzing dietary changes across regions, countries, genders, urbanization levels, and education levels. The findings aim to guide personalized interventions, policymaking, and provide dietary recommendations for IBD patients to support public health policies.

## Methods

We conducted a meta-analysis to examine the association between the intake of eight dietary factors—fruits, non-starchy vegetables, dietary fiber, saturated fat, monounsaturated fatty acids, red meat, processed meat, and omega-6 fatty acids—and the risk of developing inflammatory bowel disease (IBD). A systematic search of PubMed, Web of Science, Embase, and Cochrane was performed for prospective cohort and case–control studies published up to October 18, 2024. Our comprehensive search strategy is recorded in Supplementary Table 11.

To complement this analysis, we used median intake data and 95% confidence intervals (CIs) from the Global Dietary Database (GDD) to further evaluate the relationship between these dietary factors and IBD. Intake levels of fruits, non-starchy vegetables, processed meat, unprocessed red meat, and dietary fiber were expressed in grams per day (g/day), while saturated fat, monounsaturated fatty acids, and omega-6 fatty acids were reported as a percentage of total daily energy intake (kcal%/day).

Using a Bayesian hierarchical model, we estimated global, regional, national, and population subgroup intake levels and corresponding uncertainties for the eight nutrients. Results were expressed as population-weighted averages, supported by 4,000 posterior predictions stratified across 264 population groups per country, ensuring robust model convergence and sampling quality (11).

Education and urban–rural population distributions from the United Nations (12, 13) and Barro and Lee (14) were used as weights to calculate average nutrient intakes and 95% uncertainty intervals (UIs). Subgroup analyses were conducted across regions, countries, sexes, urbanization levels, and education levels.

Additionally, we incorporated social-demographic index (SDI) data from the Global Burden of Disease (GBD) study (15), along with IBD-related morbidity and prevalence data, to assess global dietary patterns and trends from 1990 to 2018 in 185 countries, and to examine the relationship between education level and IBD-related nutrient intake from 1900 to 2015 in 145 countries.

This approach provides a comprehensive characterization of nutrient intake variations and temporal trends through uncertainty estimation. In the Bayesian framework, a 95% UI indicates that the true mean lies within the interval with at least 95% probability. For comparisons across groups or time, a 95% UI for the difference that does not include zero suggests a high probability of a true difference.

By employing uncertainty estimation rather than conventional hypothesis testing, this study offers a more informative and probabilistic framework for capturing dietary intake patterns and trends relevant to IBD risk (16).

## Result

### Relationship between dietary components and the risk of IBD

The results of meta-analysis showed that higher intakes of fruits (OR: 0.68; 95% CI: 0.59–0.78), vegetables (OR: 0.72; 95% CI: 0.65–0.80), and dietary fiber (OR: 0.71; 95% CI: 0.60–0.84) were significantly associated with a reduced risk of IBD. In contrast, higher intakes of monounsaturated fatty acids (OR: 1.82; 95% CI: 1.21–2.73), red meat and processed meat (OR: 1.14; 95% CI: 1.01–1.29), Omega-6 fatty acids (OR: 1.34; 95% CI: 1.07–1.69), and saturated fat (OR: 1.39; 95% CI: 1.03–1.87) were significantly associated with an increased risk of IBD (Supplementary Figures 1–7). Overall, higher intakes of fruits, vegetables, and dietary fiber were significantly linked to a reduced IBD risk, while higher intakes of saturated fat, monounsaturated fatty acids, red meat, processed meat, and Omega-6 fatty acids were positively associated with IBD risk.

### Global, regional, and national intake of 8 dietary factors in 2018

In 2018, substantial regional disparities were evident in the intake of eight key dietary components. The global average fruit intake was 102.8 g/day, with High-Income Countries reporting the highest levels at 124.1 (104.0–147.7) g/day, while South Asia had the lowest at 79.1 (51.9–117.3) g/day. For non-starchy vegetables, global intake reached 149.2 (139.4–159.4) g/day, ranging from 182.8 (146.2–228) g/day in Central and Eastern Europe and Central Asia to 123.3 (112.4–134.2) g/day in High-Income Countries.

Processed meat consumption averaged 29.1 (25.6–33.1) g/day globally, but varied considerably across regions—peaking in Central and Eastern Europe and Central Asia at 59.8 (49.4–70.2) g/day and dropping to just 4.6 (2.4–8.2) g/day in South Asia. A similar pattern was observed for unprocessed red meat, with a global mean of 50.8 (45.7–56.4) g/day, the highest levels again in Central and Eastern Europe and Central Asia (91.3 [73.8–112.7]) and the lowest in South Asia (19.4 [7–32.7] g/day).

Regarding dietary fats, saturated fat intake averaged 11.0 (10.5–11.4) kcal%/day globally, ranging from 13.7 (12.8–14.4) in High-Income Countries to 6.4 (4.1–8.7) in South Asia. Monounsaturated fat intake followed a comparable trend, with a global mean of 12.0 (11.5–12.6) kcal%/day, reaching 15.2 (13.9–16.6) in Central and Eastern Europe and Central Asia and falling to 6.2 (3.9–8.5) in South Asia. Omega-6 fatty acid intake showed relatively minor variation across regions, ranging narrowly between 2.5 and 2.7 kcal%/day.

For dietary fiber, the global average was 22.5 (20.8–24.2) g/day. South Asia had the highest intake at 33.6 (17.3–49.5) g/day, while East and Southeast Asia had the lowest at 12.2 (9.7–14.8) g/day (Table 1).

**Table 1:** The average intake of eight dietary factors in 185 countries/regions in 2018, including fruits, non-starchy vegetables, processed meats, unprocessed red meats, dietary fiber (in grams/day), saturated fats, monounsaturated fatty acids, and total omega-6 fatty acids (in kcal%/day), classified by age, gender, education level, and residential area.

		Mean (95% UI)							
		Worldwide	East & Southeast Asia	Central and eastern Europe and central Asia	High-Income Countries	Latin America & Caribbean	Middle East & North Africa	South Asia	Sub-Saharan Africa
Overall	Fruits	102.8 (95.5–110.7)	93.1 (78.0–108.9)	119.6 (97.7–146.5)	124.1 (104.0–147.7)	113.1 (98.1–128.1)	109.5 (87.3–131.9)	79.1 (51.9–117.3)	81.5 (71.3–93.5)
	Non-starchy vegetables	149.2 (139.4–159.4)	171.1 (147.7–197.4)	182.8 (146.2–228)	123.3 (112.4–134.2)	130.2 (113.5–150.1)	152.1 (129.8–177.4)	180.4 (117.9–238.9)	136.6 (118.6–155)
	Total processed meats	29.1 (25.6–33.1)	21.2 (15.6–27.1)	59.8 (49.4–70.2)	32.6 (26.4–40.5)	35.3 (28.5–42.3)	27.0 (19.1–38.4)	4.6 (2.4–8.2)	13.9 (10.9–17.8)
	Unprocessed red meats	50.8 (45.7–56.4)	53.5 (42.1–67.6)	91.3 (73.8–112.7)	59.6 (52–67.1)	61.6 (49.7–74.1)	40.6 (33.8–49.1)	19.4 (7–32.7)	23.0 (19–27.9)
	Saturated fat	11.0 (10.5–11.4)	13.6 (12–15.5)	12.2 (11.6–12.9)	13.7 (12.8–14.4)	9.2 (8.5–10)	10.5 (9.6–11.3)	6.4 (4.1–8.7)	9.6 (9–10.2)
	Monounsaturated fatty acids	12.0 (11.5–12.6)	9.1 (8.3–9.9)	15.2 (13.9–16.6)	13.8 (12.8–14.8)	10.6 (9.7–11.5)	13.5 (11.7–15.2)	6.2 (3.9–8.5)	12 (11.1–12.9)
	Total omega-6 fat	2.6 (2.6–2.6)	2.6 (2.5–2.6)	2.6 (2.5–2.6)	2.6 (2.6–2.7)	2.6 (2.6–2.7)	2.7 (2.6–2.8)	2.5 (2.4–2.7)	2.6 (2.6–2.7)
	Dietary fiber	22.5 (20.8–24.2)	12.2 (9.7–14.8)	23.5 (19.1–27.6)	18.9 (16.8–21.1)	16.9 (14.5–19.7)	29.5 (22.6–36.4)	33.6 (17.3–49.5)	27.4 (24.2–31.1)
Sex									
Female	Fruits	109.2 (101.4–117.4)	101.6 (83.7–118.8)	125.9 (102.5–154.7)	136.3 (114.9–160.8)	117.9 (102.3–133.5)	115.9 (94–137.9)	80.3 (53.2–120)	85.6 (74.7–97.7)
	Non-starchy vegetables	155.3 (145.3–166.1)	180.8 (155.5–210.4)	182.3 (148.3–224.2)	134.3 (122.2–146.2)	133.2 (115.6–153.5)	159.1 (135.9–186.1)	178.9 (112.9–241.6)	145.7 (124.9–169.1)
	Total processed meats	26.9 (23.7–30.6)	21.2 (16.6–26.8)	53.6 (43.2–64.3)	28.4 (22.8–35.4)	34.1 (27.3–40.9)	24.4 (17–35.2)	4.2 (2.2–7.7)	13.0 (10.2–16.6)
	Unprocessed red meats	49.6 (44.7–55)	52.6 (41.3–66.7)	91.4 (73.4–113.2)	53.9 (46.7–61.2)	60.6 (48.2–73.1)	39.5 (32.6–47.5)	19.3 (7.5–31.2)	22.4 (18.9–26.7)
	Saturated fat	11.2 (10.7–11.7)	13.9 (12.3–15.7)	12.3 (11.6–12.9)	14 (13.1–14.9)	9.4 (8.7–10.2)	10.9 (10–11.8)	6.5 (4.2–8.8)	9.8 (9.2–10.5)
	Monounsaturated fatty acids	11.8 (11.3–12.3)	9.3 (8.6–10)	14.8 (13.5–16.1)	13.8 (12.8–14.7)	10.6 (9.7–11.5)	12.7 (11–14.4)	6.2 (3.9–8.4)	11.7 (10.8–12.6)
	Total omega-6 fat	2.6 (2.6–2.6)	2.6 (2.5–2.6)	2.6 (2.5–2.7)	2.6 (2.6–2.7)	2.6 (2.6–2.7)	2.7 (2.6–2.8)	2.5 (2.4–2.7)	2.6 (2.6–2.6)
	Dietary fiber	22.5 (20.8–24.3)	12.4 (9.8–15)	23.4 (19.8–27.6)	19.1 (17–21.6)	17.1 (14.7–19.9)	29 (22.1–35.8)	33.7 (17.6–49.9)	27.6 (24.3–31.3)
Male	Fruits	96.1 (89.4–103.2)	81.4 (67.2–95.2)	103.9 (83.5–129.8)	110.9 (93.1–132.6)	107.9 (93.8–122.3)	103.3 (82.5–124.4)	78.3 (51.2–117)	83.3 (72.9–95.1)
	Non-starchy vegetables	142.8 (133.5–152.8)	162.4 (139.8–188.7)	161.3 (129.6–200.5)	111.9 (100.9–122.8)	126.8 (111.2–145.2)	145.4 (123.5–171.6)	180.5 (119.3–241.6)	140.7 (121.5–163.3)
	Total processed meats	31.2 (27.5–35.5)	21.3 (16.9–26.9)	65 (53.9–76.3)	36.5 (29.2–45.2)	36.5 (29.5–43.5)	28.9 (20.2–40.8)	4.9 (2.6–8.8)	15.0 (11.6–19.3)
	Unprocessed red meats	52 (46.8–57.8)	20.1 (16.5–24.5)	96.2 (78.4–118.3)	65.4 (56.9–74.1)	62.2 (50–74.3)	41.5 (34–50.5)	18.8 (5.3–32.4)	21.6 (18.1–25.9)
	Saturated fat	10.7 (10.3–11.2)	13.6 (12–15.3)	11.9 (11.3–12.6)	13.3 (12.6–14)	9.0 (8.2–9.7)	10.2 (9.4–11.1)	6.3 (4.2–8.5)	9.4 (8.8–10.1)
	Monounsaturated fatty acids	12.2 (11.7–12.8)	9.2 (8.5–10)	15.7 (14.3–17.1)	13.8 (12.6–15)	10.6 (9.7–11.5)	14.1 (12.1–16)	6.3 (4.1–8.5)	12 (11.1–13)
	Total omega-6 fat	2.6 (2.6–2.6)	2.6 (2.5–2.6)	2.6 (2.5–2.6)	2.6 (2.6–2.7)	2.6 (2.6–2.7)	2.7 (2.6–2.8)	2.5 (2.4–2.7)	2.6 (2.6–2.6)
	Dietary fiber	22.3 (20.7–24)	12.0 (9.4–14.7)	23.6 (20–27.6)	18.5 (16.5–20.9)	16.8 (14.4–19.4)	29.8 (22.8–36.8)	33.9 (17.6–50)	27.0 (23.7–30.6)
Area of residence									
Rural	Fruits	98.8 (91.6–106.6)	84.2 (70.1–98.2)	120.7 (98.7–147.6)	141.3 (119.4–166.9)	105.6 (93–119.9)	94.1 (76.6–112)	70.8 (46.9–104.6)	73.4 (64.2–83.8)
	Non-starchy vegetables	149.4 (139.6–159.9)	172.5 (147.8–201.8)	186.1 (148.2–233.4)	123.5 (112–134.9)	129.4 (112.3–148.1)	147.0 (125.3–171.5)	178.1 (113.6–238.1)	138.5 (120.5–156.6)
	Total processed meats	26.1 (22.9–29.8)	20.8 (14.7–26.7)	60 (49.6–70.2)	35.9 (28.9–44.5)	26.1 (20.5–31.7)	10.6 (7.3–15.2)	6.2 (3.2–11.3)	12.6 (9.9–16)
	Unprocessed red meats	46.9 (41.9–52.5)	51.9 (40.4–66.2)	85.5 (69–106)	59.1 (51.5–66.6)	54.5 (43.5–65.5)	38.3 (31.5–46.1)	15.5 (5.1–26.2)	18.7 (15.5–22.6)
	Saturated fat	10.5 (10.1–11)	13.6 (11.9–15.6)	11.9 (11.3–12.6)	13.4 (12.7–14.2)	8.3 (7.6–9)	10.1 (9.2–10.9)	5.7 (3.5–7.9)	9.3 (8.7–9.9)
	Monounsaturated fatty acids	11.9 (11.3–12.4)	9.0 (8.2–9.8)	15.1 (13.7–16.4)	13.6 (12.6–14.6)	10.3 (9.4–11.1)	13.5 (11.6–15.3)	6.2 (4.1–8.4)	11.8 (11–12.7)
	Total omega-6 fat	2.6 (2.6–2.6)	2.6 (2.5–2.6)	2.6 (2.5–2.6)	2.6 (2.6–2.7)	2.6 (2.6–2.7)	2.8 (2.7–2.8)	2.5 (2.4–2.6)	2.5 (2.5–2.6)
	Dietary fiber	22.4 (20.8–24.1)	11.8 (9.3–14.3)	24.2 (20.7–28.5)	19.1 (17–21.5)	19.3 (16.7–22.4)	24.7 (19.6–31.1)	32.8 (16.4–49.3)	27.4 (24.2–31)
Urban	Fruits	107.7 (100.4–115.5)	99.6 (83.4–115.7)	118.3 (96.6–145.7)	119.6 (100.5–142.3)	117.8 (103.9–133.7)	114.2 (91.7–136.6)	98 (64.4–146.7)	91.8 (80.4–104.7)
	Non-starchy vegetables	148.8 (139–159.1)	170.6 (146–197.3)	181.4 (144.1–226.2)	123.2 (111.8–134.6)	130.6 (113.9–149.3)	154.6 (131.6–181.2)	183.8 (116.3–247.3)	134.3 (116.2–152.8)
	Total processed meats	30.6 (26.9–35)	21.8 (15.7–28)	59.5 (49.4–69.3)	31.7 (25.6–39.4)	40.2 (32.5–47.8)	31.1 (22–43.8)	1.4 (0.7–2.5)	15.1 (11.8–19.1)
	Unprocessed red meats	54.1 (49–59.8)	55.7 (44.1–70)	95 (76.9–117.6)	59.8 (51.9–67.5)	65 (52.8–77.6)	41.4 (34.2–49.7)	28.1 (8.9–47.1)	28.4 (23.5–34.2)
	Saturated fat	11.3 (10.9–11.8)	13.7 (12.1–15.6)	12.4 (11.8–13.1)	13.7 (12.9–14.5)	9.8 (9–10.5)	10.7 (9.8–11.5)	7.8 (5.1–10.4)	10.1 (9.5–10.7)
	Monounsaturated fatty acids	12.2 (11.6–12.7)	9.2 (8.4–10)	15.4 (14–16.7)	13.8 (12.8–14.9)	10.8 (9.9–11.7)	13.5 (11.8–15.3)	6.4 (4–8.7)	12.2 (11.3–13.1)
	Total omega-6 fat	2.7 (2.6–2.7)	2.6 (2.5–2.6)	2.6 (2.5–2.6)	2.6 (2.6–2.7)	2.7 (2.6–2.7)	2.7 (2.6–2.8)	2.6 (2.4–2.7)	2.7 (2.7–2.8)
	Dietary fiber	22.4 (20.7–24.2)	12.2 (9.6–14.8)	23.1 (19.5–27)	18.8 (16.7–21.2)	15.6 (13.4–18)	31.3 (23.8–38.7)	35.7 (19.1–52.2)	27.3 (23.9–31)
Education level									
0–6 years (low)	Fruits	88.9 (83–95.3)	77.8 (67.7–88.2)	92.2 (77.7–109.3)	118.1 (100.3–136)	96.2 (85.1–109.4)	90.2 (73.2–107.2)	58 (20.3–93.9)	71.3 (62–82.4)
	Non-starchy vegetables	136.7 (128–146)	164.1 (136.5–191.8)	174.3 (135.3–220.9)	117.2 (106.3–128.5)	102.8 (89.4–116.1)	134.6 (110.7–159.2)	154.6 (107.1–204.3)	134.9 (117.2–152.3)
	Total processed meats	28.9 (24.9–33.5)	15.5 (11.2–21.1)	62.2 (48.8–75.5)	29.1 (23.3–36.1)	35.6 (28–43.6)	26.5 (17.6–39.2)	5.2 (2.6–10.1)	15.1 (10.9–20.7)
	Unprocessed red meats	47.7 (42.4–53.6)	45.9 (36.4–58)	84.8 (65.9–108.7)	61.5 (52.7–70)	53 (42.2–63.3)	40.2 (33–48.9)	16.2 (4.4–27.3)	21.0 (16.4–26.7)
	Saturated fat	10.6 (10.1–11.1)	12.4 (10.3–14.6)	12.3 (11.6–13.1)	13.2 (12.4–13.9)	8.5 (7.7–9.4)	10.1 (9.1–11.1)	6.1 (3.5–8.8)	9.6 (8.8–10.4)
	Monounsaturated fatty acids	12.1 (11.5–12.7)	8.7 (7.6–9.8)	15.8 (14.2–17.5)	13.3 (12.3–14.3)	10.6 (9.6–11.6)	13.1 (11.4–14.7)	6.2 (3.6–9)	12.4 (11.4–13.5)
	Total omega-6 fat	2.6 (2.6–2.6)	2.6 (2.5–2.7)	2.6 (2.5–2.7)	2.6 (2.6–2.7)	2.6 (2.6–2.7)	2.7 (2.6–2.8)	2.5 (2.3–2.7)	2.6 (2.6–2.7)
	Dietary fiber	22.6 (20.8–24.6)	12.4 (9.6–15.3)	24.7 (19.8–29.6)	20.6 (18.4–22.9)	15.9 (13.3–19.1)	26.7 (19.9–33.3)	33.3 (13–52.9)	28.7 (24.8–33.3)
6-12 years (medium)	Fruits	101.3 (95.2–107.8)	100.6 (87.6–113.4)	96.8 (81.1–115.2)	106.5 (90.1–122.7)	113.7 (99.6–129.7)	105.1 (85.9–124.7)	84.8 (30.4–139.5)	92.6 (80.3–106.4)
	Non-starchy vegetables	144.5 (135.2–154.1)	174.6 (143.2–205.3)	179.4 (140.6–229.9)	118.8 (107.6–130.2)	114.0 (99.1–128.2)	142.9 (118.2–169.5)	162.6 (110.6–218.7)	145.4 (126.5–163.6)
	Total processed meats	31.0 (26.9–35.7)	18.9 (13.7–25.9)	58.9 (46.1–72.2)	34.2 (27.6–42.4)	38.1 (29.7–46.8)	30.5 (20.5–44.5)	5.6 (2.8–10.9)	17.0 (12.2–23.1)
	Unprocessed red meats	52.5 (47.1–58.5)	50.5 (40.3–63.3)	82.2 (64.3–104.5)	61.2 (53–69.7)	63.9 (51.5–75.9)	44.7 (36.6–54.4)	21.3 (5.4–37.7)	29.6 (23.3–37.6)
	Saturated fat	11.0 (10.5–11.5)	12.6 (10.4–14.8)	12.7 (12–13.4)	13.7 (13–14.5)	9.2 (8.2–10)	10.4 (9.4–11.4)	6.7 (4.1–9.5)	9.7 (8.8–10.5)
	Monounsaturated fatty acids	12.2 (11.6–12.8)	9.3 (8.3–10.5)	15.3 (13.6–17)	13.8 (12.8–14.8)	11.0 (10.0–12.0)	13.0 (11.3–14.6)	6.5 (3.7–9.2)	12.0 (11.0–13.0)
	Total omega-6 fat	2.6 (2.6–2.6)	2.6 (2.5–2.6)	2.6 (2.5–2.7)	2.6 (2.6–2.7)	2.7 (2.6–2.7)	2.7 (2.6–2.8)	2.5 (2.3–2.7)	2.7 (2.6–2.7)
	Dietary fiber	21.9 (20.1–23.8)	12.4 (9.6–15.2)	23.2 (18.5–27.8)	19.3 (17.2–21.4)	15.7 (13–18.9)	26.3 (20.1–32.5)	33.2 (11.8–52.7)	28 (24–32.6)
>12 years (high)	Fruits	118.5 (111.4–126.1)	122.4 (106–138.4)	93.1 (78.7–109.9)	116.8 (99.4–133.7)	133.3 (116.9–151.7)	115.7 (94.4–136.9)	109.8 (38.8–177.9)	126.0 (109.4–145.2)
	Non-starchy vegetables	159.8 (149.7–170.4)	181.4 (149.6–213.5)	170.3 (132.6–217.7)	132.7 (120.7–144.8)	140.7 (122.7–158.5)	156.7 (129.4–185.2)	183.3 (126.6–244.6)	173.2 (151.4–196.2)
	Total processed meats	31 (27–35.7)	22.2 (16.1–30.3)	52.4 (39.3–65.6)	32 (25.6–39.9)	42.8 (33.8–51.9)	31.1 (21.3–45.5)	6 (2.9–11.7)	17.0 (12.5–23.1)
	Unprocessed red meats	57.8 (52.4–63.5)	51.1 (40.4–64.3)	85.2 (66.9–107.8)	56 (48.2–63.7)	73 (59.1–86.7)	51.6 (42–63.5)	26.4 (7.4–46.8)	43.0 (34.1–53.9)
	Saturated fat	11.2 (10.7–11.8)	13.3 (11.1–15.6)	12.7 (12.0–13.4)	13.8 (13.0–14.5)	9.6 (8.7–10.5)	10.8 (9.7–11.8)	7.0 (4.1–9.9)	9.7 (8.8–10.5)
	Monounsaturated fatty acids	12.2 (11.7–12.8)	9.9 (8.8–11)	14.5 (12.8–16.1)	13.9 (12.9–15)	11.7 (10.7–12.8)	13.9 (12.2–15.6)	6.6 (4–9.2)	11.5 (10.5–12.5)
	Total omega-6 fat	2.6 (2.6–2.7)	2.6 (2.5–2.7)	2.6 (2.5–2.7)	2.6 (2.6–2.7)	2.7 (2.6–2.8)	2.7 (2.6–2.8)	2.5 (2.4–2.7)	2.6 (2.6–2.7)
	Dietary fiber	22.8 (21–24.7)	13.2 (10.1–16.1)	21.8 (17.6–26)	20.7 (18.6–23.1)	17.1 (14.1–20.6)	27.7 (20.9–34.2)	35.3 (14.1–55.6)	29.1 (25.1–33.5)

In previous global dietary database reports, Central Europe, Eastern Europe, and Central Asia were referred to as the former Soviet Union, while Southeast Asia and East Asia were collectively called Asia. UI, Uncertainty Interval.

At the national level, differences were even more striking. Fruit intake ranged from 471.7 (420.6–528.7) g/day in Bosnia and Herzegovina to only 4.2 (3.7–4.7) g/day in Papua New Guinea. Croatia led in non-starchy vegetable consumption at 445.8 (372.5–531.5) g/day, in stark contrast to Guinea’s 21.1 (17.2–25.8) g/day. Mongolia reported the highest processed meat intake at 119.6 (112.5–127.0) g/day, while Bangladesh had the lowest at 0.4 (0.3–0.4) g/day.

Unprocessed red meat intake was greatest in Barbados at 234.0 (192.0–285.0) g/day and lowest in India at 3.0 (2.4–3.8) g/day. Samoa topped the list for saturated fat at 26.9 (25.5–28.3) kcal%/day, whereas Nepal recorded the lowest at 3.0 (2.7–3.4) kcal%/day. Monounsaturated fat intake spanned from 27.6 (27.1–28.0) kcal%/day in Sweden to 3.8 (3.2–4.4) kcal%/day in Vietnam. Chile had the highest omega-6 intake at 3.2 (3.1–3.2), while Romania had the lowest at 2.2 (2.1–2.2) kcal%/day.

**Figure 1:**
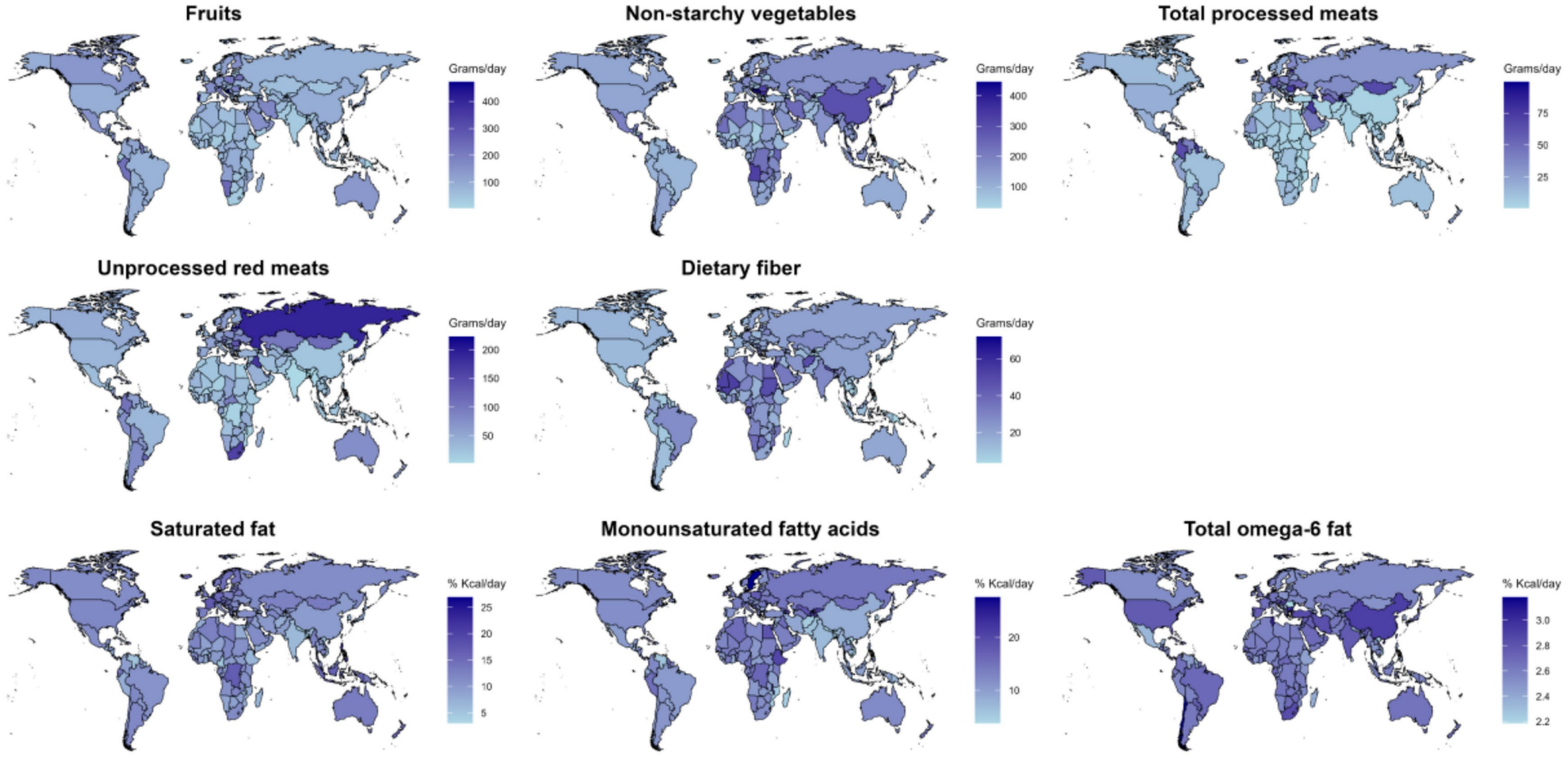
In 2018, the average intake of eight dietary factors globally and regionally was assessed: fruits, non-starchy vegetables, processed meats, unprocessed red meats, and dietary fiber (in grams/day), and saturated fats, monounsaturated fatty acids, and total omega-6 fatty acids (in kcal%/day).

Dietary fiber intake showed the broadest range across countries, from 71.9 (66.9–77.3) g/day in Palestine to 3.8 (3.2–4.5) g/day in Papua New Guinea (Supplementary Table 1; Figure 1).

Collectively, these findings underscore substantial global and regional variation in dietary patterns, emphasizing the need for regionally tailored dietary guidelines and interventions.

### Intake of 8 dietary nutrients by age group and sex

In 2018, sex-based differences in the intake of the eight dietary factors largely mirrored global trends, although regional variation was observed (Table 1; Supplementary Tables 4, 5).

By age and region, fruit intake ranged from 128.6 g/day (89.8–175.9) among individuals aged 95–100 in High-Income Countries to just 8.1 g/day (5.6–11.3) among infants (0–1 years) in Sub-Saharan Africa. Non-starchy vegetable consumption peaked at 189.7 g/day (135.3–256.7) in the 20–24 age group in South Asia and dropped to 4.6 g/day (3.2–6.4) in the 0–1 age group in the Middle East and North Africa.

Processed meat intake was highest at 53.9 g/day (37.8–73.2) among adults aged 25–29 in Central and Eastern Europe and Central Asia, while the lowest value, 1.1 g/day (0.7–1.8), was recorded in South Asia among infants. Unprocessed red meat intake followed a similar pattern, ranging from 83.1 g/day (55.6–117.5) in young adults aged 20–24 in Central and Eastern Europe and Central Asia to 0.7 g/day (0.4–1.0) in infants in Sub-Saharan Africa.

Saturated fat intake peaked at 14.2 kcal%/day (10.2–19.1) in the 0–1 age group in High-Income Countries and was lowest at 4.8 kcal%/day (3.3–6.6) among 25–29-year-olds in South Asia. Monounsaturated fat intake reached a maximum of 13.0 kcal%/day (9.3–17.7) among adults aged 35–39 in Central and Eastern Europe and Central Asia and declined to 4.2 kcal%/day (2.9–5.7) among those aged 95–100 in East and Southeast Asia.

**Figure 2:**
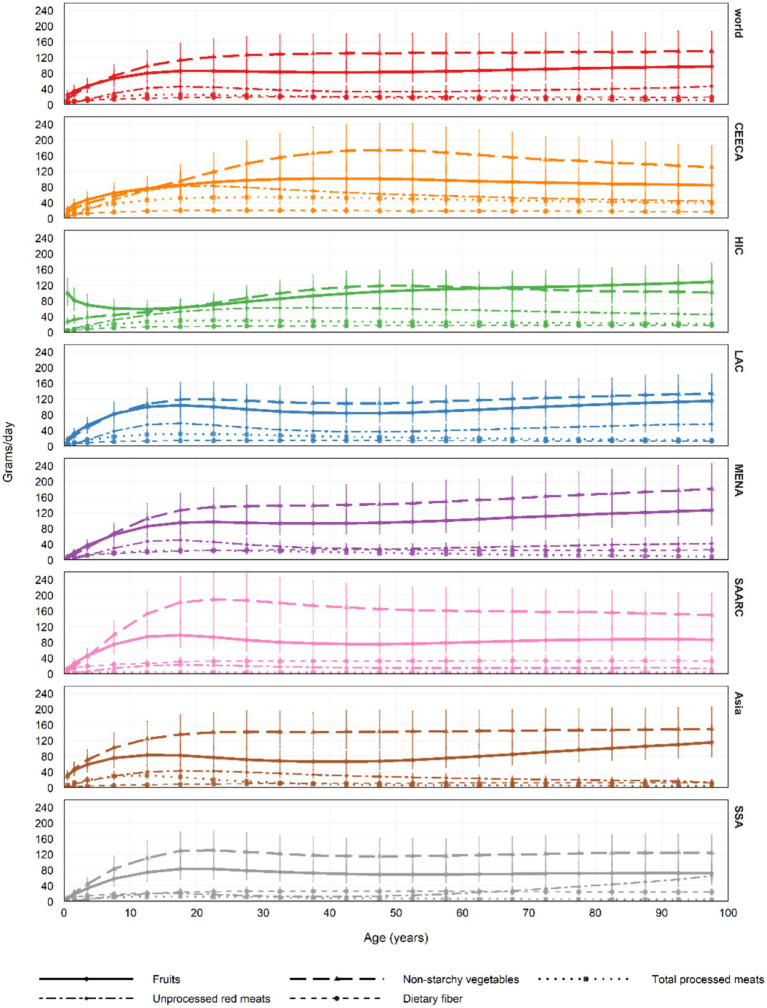
In 2018, the average intake of five dietary factors across all age groups globally and regionally was assessed: fruits, non-starchy vegetables, processed meats, unprocessed red meats, and dietary fiber (in grams/day), with error bars representing the 95% uncertainty intervals (UI). In previous global dietary database reports, Central Europe, Eastern Europe, and Central Asia were referred to as the former Soviet Union, while Southeast Asia and East Asia were collectively called Asia. UI, Uncertainty Interval.

**Figure 3:**
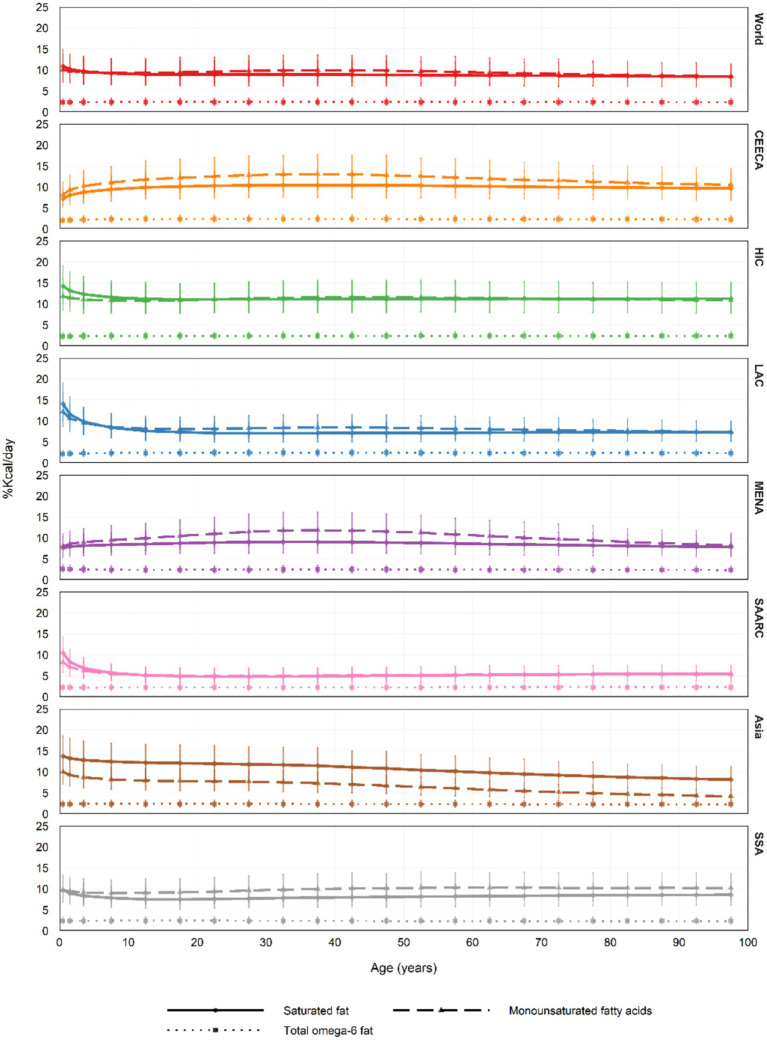
In 2018, the average intake of three dietary factors (saturated fat, monounsaturated fatty acids, and total Omega-6 fatty acids) across all age groups globally and by region was reported in Kcal%/day, with error bars representing the 95% uncertainty interval (UI). In previous Global Dietary Database reports, Central Europe, Eastern Europe, and Central Asia were referred to as the former Soviet Union, while Southeast Asia and East Asia were collectively labeled as Asia. UI, uncertainty interval.

Omega-6 fatty acid intake showed minimal variation in the 0–1 age group in Central and Eastern Europe and Central Asia, with values ranging from 2.1 (1.4–2.9) to 2.0 (1.3–2.8) kcal%/day. Dietary fiber intake was highest at 32.9 g/day (22.9–45.1) in the 75–79 age group in South Asia, and lowest at 2.0 g/day (1.3–2.8) among infants in Asia (Figures 2, 3; Supplementary Table 8).

Overall, sex differences in intake patterns remained consistent with global, regional, and national trends (Supplementary Table 9, Supplementary Table 10).

### Trends in the intake of eight dietary components from 1990 to 2018

From 1990 to 2018, processed meat was the fastest-growing dietary factor globally, showing a 26% increase (Table 1; Supplementary Table 2). Regionally, non-starchy vegetable intake rose substantially—by 49% in Central and Eastern Europe and Central Asia, 45% in Latin America and the Caribbean, and 13% in the Middle East and North Africa. Unprocessed red meat consumption increased by 38% in East and Southeast Asia, while processed meat intake in South Asia rose sharply by 56%. In High-Income Countries, processed meat intake grew by 25%, and fruit consumption in Sub-Saharan Africa climbed by 15%.

At the national level, changes were even more pronounced (Supplementary Tables 1, 3). Fruit intake in Bosnia and Herzegovina surged by 1,050%, while non-starchy vegetable consumption in China increased tenfold (1000%). The Maldives saw a doubling of processed meat intake and a 391% rise in dietary fiber consumption. Myanmar exhibited an exceptional 1970% increase in unprocessed red meat intake. In Bangladesh, saturated fat and monounsaturated fatty acid intake rose by 48 and 129%, respectively. In Suriname, omega-6 fat intake grew modestly by 8%.

Across global, regional, and national levels, the intake growth patterns of the eight dietary factors remained generally consistent across sexes (Table 1; Supplementary Tables 4–7).

### Intake of 8 dietary factors by urbanicity

Data from 2018 reveal substantial regional differences in dietary patterns between urban and rural populations. In most regions, fruit intake tends to be higher in urban areas, with the widest gap observed in the Middle East and North Africa. Conversely, in High-Income Countries and in Central and Eastern Europe and Central Asia, rural populations consume more fruit.

Non-starchy vegetable intake is generally greater in rural areas, except in the Middle East and North Africa, where urban residents consume more. In other regions, differences are minimal. Both processed and unprocessed red meat consumption is typically elevated in urban settings; however, rural areas in High-Income Countries and North Africa report higher intake of processed meat. For unprocessed red meat, urban areas show greater consumption, particularly in North Africa.

The proportion of energy from saturated fat and monounsaturated fatty acids is markedly higher in urban areas, especially in North Africa. Omega-6 fatty acid intake also tends to be higher in urban regions, with the exception of rural populations in the Middle East.

Dietary fiber intake varies: it is higher in rural areas of High-Income Countries and Latin America, while in the Middle East and Sub-Saharan Africa, urban areas report higher intake. Other regions do not show significant differences.

**Figure 4:**
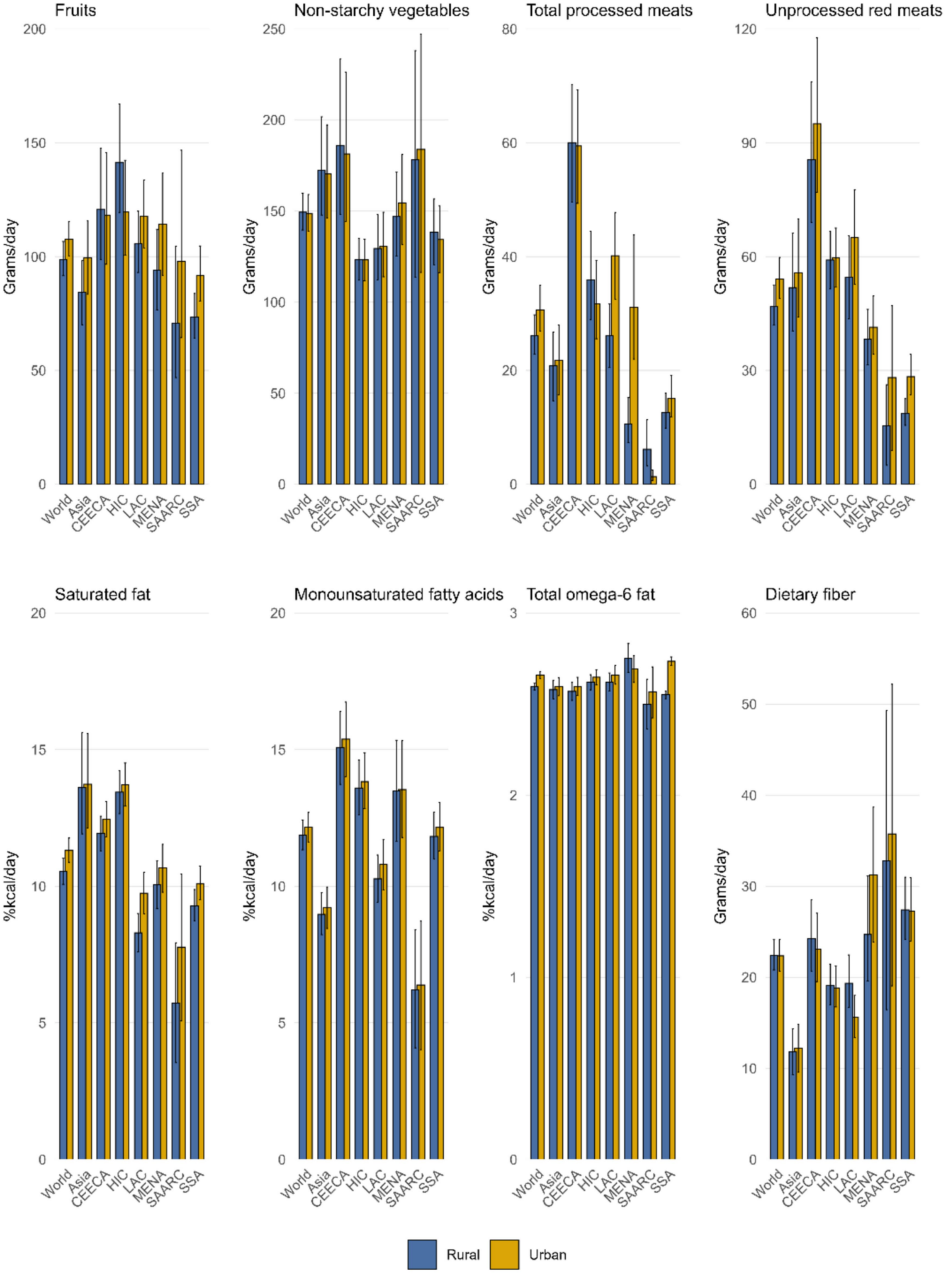
In 2018, the average intake of eight dietary factors across all age groups globally and by region was reported, including fruits, non-starchy vegetables, processed meats, unprocessed red meat, dietary fiber (in grams per day), and saturated fat, monounsaturated fatty acids, and total Omega-6 fatty acids (in Kcal%/day).

These findings underscore the influence of urban–rural living environments on dietary behavior and highlight the regional diversity of dietary patterns (Table 1; Figure 4).

### Intake of 8 dietary factors by education level

Educational level significantly shapes the intake patterns of the eight dietary nutrients across regions. In Central and Eastern Europe and Central Asia, fruit consumption peaks among individuals with medium education, whereas in high-income countries, the same group reports the lowest intake. In most other regions, fruit intake tends to rise with increasing education levels.

For non-starchy vegetables, intake is again highest among the medium education group in Central and Eastern Europe and Central Asia but generally exhibits a positive correlation with education elsewhere. Processed meat intake shows a declining trend with higher education in Central and Eastern Europe and Central Asia, while in high-income countries, the medium education group reports the highest levels. In other regions, processed meat consumption rises in line with educational attainment.

Unprocessed red meat intake decreases with higher education in high-income countries, yet increases in other regions, with the lowest intake reported by the medium education group in Central and Eastern Europe and Central Asia. Saturated fat intake generally rises with education, whereas monounsaturated fat intake demonstrates an inverse trend in Central and Eastern Europe, Central Asia, and Sub-Saharan Africa.

Omega-6 fatty acid intake shows minimal variation across education levels. For dietary fiber, intake is typically lower among individuals with medium education but increases with higher education in Central and Eastern Europe and Central Asia.

**Figure 5:**
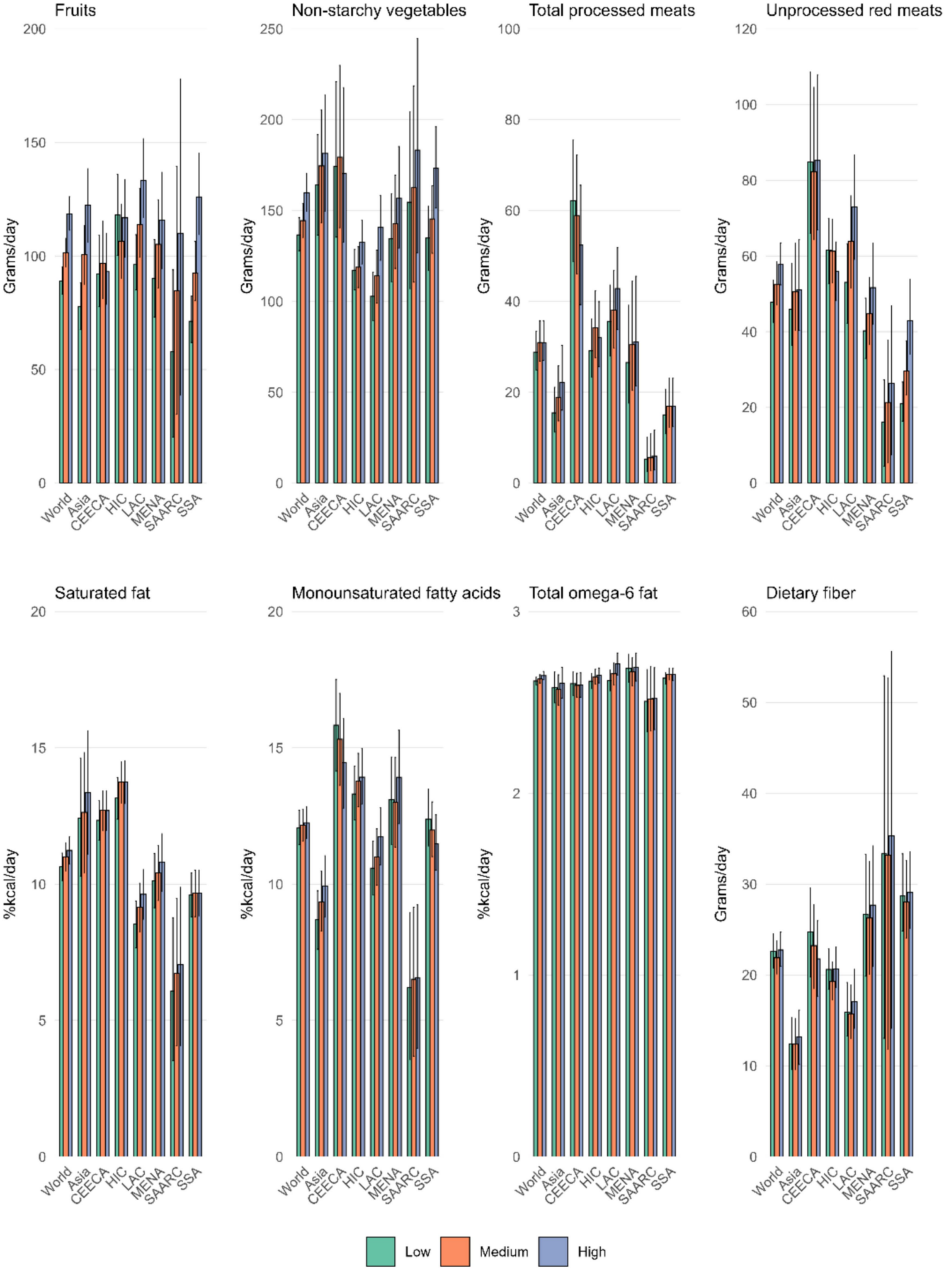
In 2015, the average intake of eight dietary factors across all age groups globally and by region, categorized by education level, was reported. These factors include fruits, non-starchy vegetables, processed meats, unprocessed red meat, and dietary fiber (in grams per day), as well as saturated fat, monounsaturated fatty acids, and total Omega-6 fatty acids (in Kcal%/day).

These findings underscore the important roles of both educational attainment and regional cultural context in shaping dietary behaviors (Table 1; Figure 5).

### Trends and associations of eight dietary factors with socio-demographic development index, incidence, and prevalence of inflammatory bowel disease

Between 1990 and 2018, the Social-Demographic Index (SDI) was positively correlated with the intake of fruit, processed meat, unprocessed red meat, and saturated fat, with the strongest correlation observed for processed meat in 1990 (*R* = 0.56).

**Figure 6:**
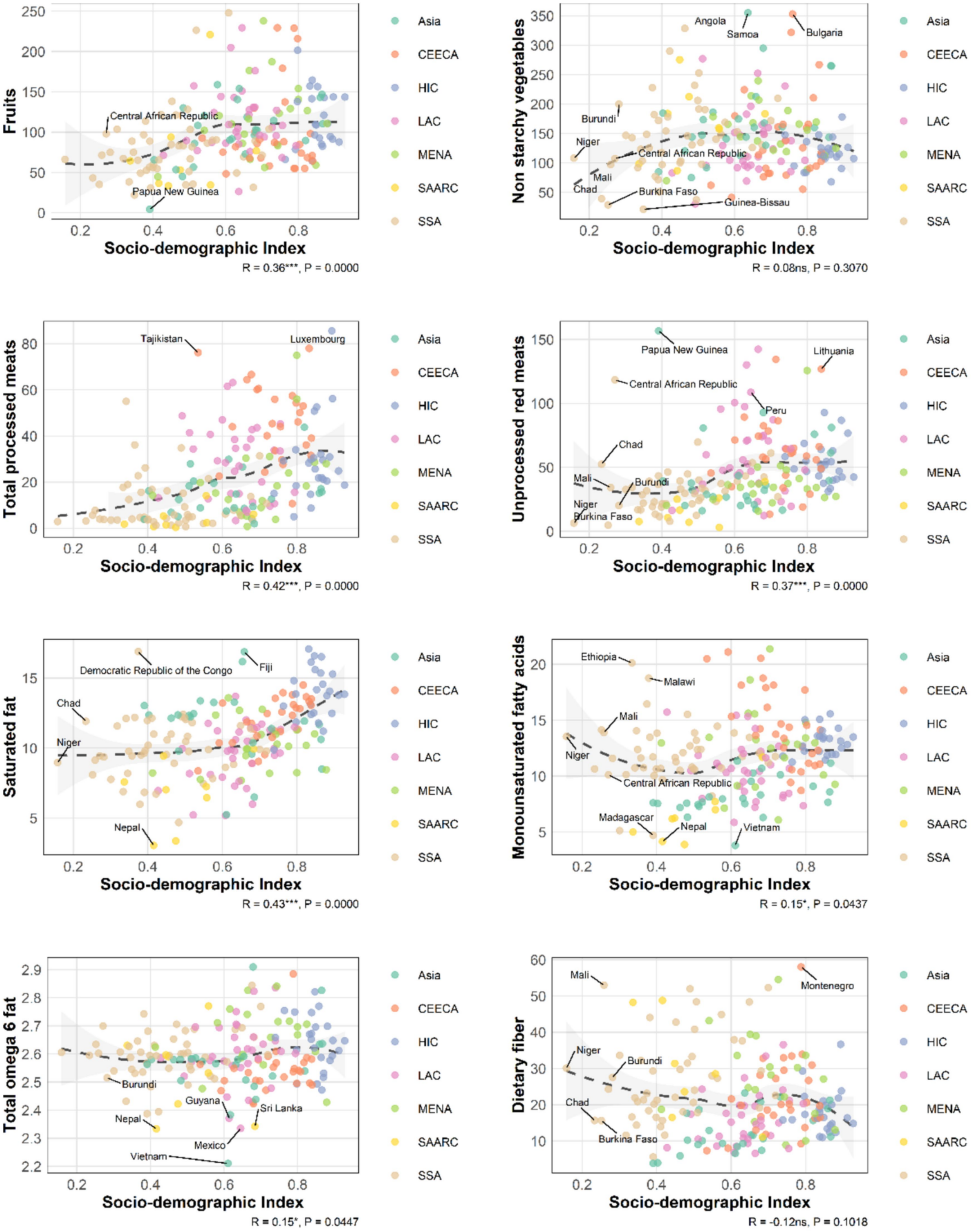
Spearman correlation was assessed in this analysis between SDI and the intakes of 8 dietary components among a total of 185 countries (2018).

Furthermore, processed meat, unprocessed red meat, saturated fat, and monounsaturated fatty acids were positively associated with both the incidence and prevalence of IBD, with the correlation for saturated fat strengthening over time (Figure 6; Supplementary Figures 8–12).

## Discussion

Between 1990 and 2018, processed meat was the dietary factor with the highest global growth, increasing by 26%. This rise parallels the global increase in inflammatory bowel disease (IBD) incidence. Among the 185 countries included in the study, processed meats, unprocessed red meat, saturated fats, and monounsaturated fatty acids showed a positive correlation with IBD prevalence and incidence in both 1990 and 2018.

Although fruits, non-starchy vegetables, and dietary fiber are generally considered protective, no significant negative correlation was found between their intake and IBD incidence. This may be due to global fruit and vegetable intake being well below recommended levels (17), and meta-analyses often underestimate dietary risk factors, which may not reflect real-world consumption patterns. Furthermore, risk factors like processed meats, red meat, and high-fat diets may exacerbate IBD through mechanisms such as gut microbiota imbalance and intestinal barrier damage. For instance, high-fat diets have been shown to impair gut barrier function in ulcerative colitis patients (18), potentially offsetting the protective effects of certain dietary factors.

The rapid increase in meat consumption, coupled with inadequate intake of fruits, vegetables, and fiber, likely contributes to the rising IBD burden. Regionally, unprocessed red meat consumption in Asia increased by 38%, and processed meat by 28%, which may be primary dietary drivers of the rising IBD burden in the region. Economic growth during this period led to increased meat consumption, while advancements in food processing technologies improved access to processed meats (19–21). For example, China accounts for approximately 70% of global processed duck meat production, including products like roasted duck, smoked duck, and duck necks (22).

In Central and Eastern Europe and Central Asia, non-starchy vegetable intake increased by 49%, while processed meats and saturated fats remained prevalent, among the highest globally. These trends may reflect regional dietary habits, such as the prominence of processed meats in Poland’s diet (23), and research from Kazakhstan showing that meat consumption among younger populations is nearly double the recommended level (24).

In high-income countries, processed meat intake increased by 25%, continuing to be a major dietary component despite public health efforts to reduce consumption (25), albeit with limited success. In Latin America, non-starchy vegetable intake grew by 45%, but this was accompanied by a rapid increase in red and processed meat consumption. Red meat became the second-largest food category after vegetables, while the proportion of vegetables in diets declined (26).

Traditional diets in the Middle East and North Africa (MENA) and South Asia, characterized by high fiber and low fat, may offer some protection against IBD. Although modern Mediterranean diets now include more red meat and processed foods, they still emphasize plant-based foods and healthy fats (27). In Sub-Saharan Africa, traditional diets persist, but economic growth and changing social norms have led to a 27% increase in red meat consumption. Research in the region indicates that, especially among younger populations, meat consumption is seen as a symbol of status, pleasure, and socializing, suggesting that rising meat consumption is not only driven by economic growth but also tied to cultural identity and social psychology (28).

Studies have revealed significant urban–rural differences in dietary patterns related to IBD, reflecting disparities in food access, diet structure, and lifestyle. Urban areas, particularly in MENA, show higher intakes of fruits and non-starchy vegetables due to diverse food availability and easier access. In contrast, rural areas rely more on locally grown non-starchy vegetables, with lower fruit consumption. Urban areas tend to consume more processed and unprocessed red meats, while rural areas consume more unprocessed red meats. Saturated fat and monounsaturated fatty acid intake are higher in urban areas, particularly in former Soviet regions, while rural areas have lower fat intake, mostly from traditional sources. Omega-6 fatty acid intake shows little variation and is largely derived from plant oils. Dietary fiber intake is higher in rural areas, particularly in high-income countries, while urban areas in Sub-Saharan Africa show higher fiber intake.

Education level also influences dietary habits, with regional variations. In high-income countries, higher education levels tend to correlate with healthier diets, such as increased fruit and vegetable consumption and reduced intake of red and processed meats. However, in Sub-Saharan Africa, former Soviet regions, and some Asian countries, higher education levels often correlate with increased consumption of red and processed meats. This may be influenced by local cultural contexts and socio-economic conditions. For example, in former Soviet countries, traditional diets and limited food access lead to preferences for high-saturated fat and processed meat diets, despite higher education levels. In some low-and middle-income countries, multinational corporations’ marketing strategies have significantly influenced meat consumption, especially through “glocalization” that adapts products to local consumption cultures and regulations. European data shows that combining online and television marketing results in high returns, and apps now offer personalized food recommendations based on individual preferences (29–31). Additionally, fast food advertisements featuring meat images can influence consumer preferences, and government efforts to promote meat consumption through public-private partnerships may not always align with public health objectives (32, 33).

Although high-income countries have begun promoting plant-based and health-conscious diets, efforts to reduce processed meat consumption have had limited success (34). However, measures such as price regulation and food labeling have shown some promise (35, 36). Future public health policies should focus on curbing the rapid growth of meat consumption, especially processed meats, through more effective interventions such as “healthy diet” labeling systems, restrictions on unhealthy food advertising, and increased availability of vegetables, fruits, and high-fiber foods, particularly in low-and middle-income countries.

### Limitations

This study reveals global, regional, gender, urban–rural, and cultural differences in dietary patterns related to inflammatory bowel disease (IBD), but there are several limitations. First, dietary factors from the Global Dietary Database (GDD) may differ significantly from those in our meta-analysis due to variations in dietary intake measurement methods. Second, the study primarily relies on secondary data from specific regions, which may not fully represent dietary patterns and IBD incidence in low-and middle-income countries, potentially affecting the generalizability of the findings. Future research should expand data collection, particularly in regions with limited data, to better assess the global relationship between dietary patterns and IBD. Third, some data were based on self-reported dietary surveys, which may introduce recall bias or social desirability bias. Respondents may overestimate healthy eating behaviors or underestimate unhealthy ones, which could distort the accuracy of dietary patterns and their association with IBD. Future studies should prioritize more objective data collection methods, such as 24-h dietary recalls, food diaries, or tracking devices, to minimize bias. These estimates may over-or underestimate actual intake compared to individual-level data and may be less reliable when describing differences between population subgroups (37–39). Future research should focus on more precise monitoring of dietary intake. Although the study shows a positive correlation between dietary patterns and IBD burden, it does not establish causality. Since the study is based on cross-sectional data, potential confounding factors such as genetics, environmental pollution, and lifestyle may also affect IBD incidence. Longitudinal studies are needed to explore the causal effects of dietary patterns on IBD incidence. Additionally, this study explored the potential impact of multinational corporations on meat consumption (especially processed meats) in low-and middle-income countries. However, marketing strategies employed by multinational corporations can vary greatly across regions, and differences in cultural context, consumer attitudes, and media exposure may influence their effectiveness. Further research is needed to investigate these factors. A detailed analysis of multinational corporations’ marketing strategies will help better understand their impact on meat consumption. Finally, the study emphasizes the significant influence of education level and socio-economic status on dietary patterns, but these factors are often interrelated and may vary regionally. For instance, in high-income countries, higher education levels are associated with healthier diets, while in some low-and middle-income countries, higher education may still be constrained by cultural traditions and economic conditions, leading to dietary choices that diverge from expectations. Future research should focus on the complex interplay between education level, cultural background, food supply, and socio-economic context, using multidimensional analysis methods to fully understand the formation and evolution of dietary habits.

## Conclusion

This study found that between 1990 and 2018, processed meat intake showed the largest increase across 185 countries, rising by 26%, which parallels the global rise in IBD burden. The intake of eight dietary factors exhibited significant heterogeneity across global populations, with variations by age, education level, and urbanization. These findings may inform policy interventions aimed at reducing intake in high-risk groups, particularly in high-income countries and Asia, where the IBD burden is increasing rapidly. The rapid rise in both processed and unprocessed red meat intake, coupled with the long-term underconsumption of fruits, vegetables, and dietary fiber, may be key contributors to the growing global IBD burden.

## Data Availability

The datasets presented in this study can be found in online repositories. The names of the repository/repositories and accession number(s) can be found in the article/Supplementary material.
