# Supplementary material for: Dietary patterns and inflammatory bowel disease: a global assessment of eight nutrients by region, gender, and socioeconomic status
Source: Front Nutr. 2025 May 1;12:1570634. doi: 10.3389/fnut.2025.1570634 (PMC12079903; doi:10.3389/fnut.2025.1570634)
Supplement: Supplementary file 1 [file Data_Sheet_1.pdf]

Supplementary Material

Table of contents

Supplementary Table 1: National mean (95% UI) intakes of eight nutrients in 2018 across 185 countries and regions. 3

Supplementary Table 2: The average intake of eight dietary factors in 185 countries/regions in 1990, including fruits, non-starchy vegetables, processed meats, unprocessed red meats, dietary fiber (in grams/day), saturated fats, monounsaturated fatty acids, and total omega-6 fatty acids (in kcal%/day), classified by age, gender, education level, and residential area. 10

Supplementary Table 3: National mean (95% UI) intakes of eight nutrients in 1990 across 185 countries and regions. 14

Supplementary Table 4: National mean (95% UI) intakes of eight nutrients among males in 2018 across 185 countries and regions. 21

Supplementary Table 5: National mean (95% UI) intakes of eight nutrients among females in 2018 across 185 countries and regions. 28

Supplementary Table 6: National mean (95% UI) intakes of eight nutrients among females in 1990 across 185 countries and regions. 35

Supplementary Table 7: National mean (95% UI) intakes of eight nutrients among males in 1990 across 185 countries and regions. 42

Supplementary Table 8: National mean (95% UI) intakes of eight nutrients in 2018 across 185 countries, stratified by area of residence for all age groups. 49

Supplementary Table 9: National mean (95% UI) intakes of eight nutrients among males in 2018 across 185 countries, stratified by area of residence for all age groups. 56

Supplementary Table 10: National mean (95% UI) intakes of eight nutrients among females in 2018 across 185 countries, stratified by area of

residence for all age groups. 63

Supplementary Table 11: Search strategies including the key terms and the queries for each database. 70

Supplementary Figure 1: Forest plot of the association between Fruit consumption and the risk of IBD using a random-effects model. 81

Supplementary Figure 2: Forest plot of the association between Vegetables consumption and the risk of IBD using a fixed-effects model. 82

Supplementary Figure 4: Forest plot of the association between Saturated fat consumption and the risk of IBD using a fixed-effects model. 84

Supplementary Figure 5: Forest plot of the association between Monounsaturated fatty acids consumption and the risk of IBD using a fixed-effects model. 84

Supplementary Figure 6: Forest plot of the association between Omega-6 consumption and the risk of IBD using a random-effects model. 85

Supplementary Figure 7: Forest plot of the association between Dietary Fiber consumption and the risk of IBD using a random-effects model. 86

Supplementary Figure 8: Spearman correlation was assessed in this analysis between SDI and the intakes of 8 dietary components among a total of 185 countries (1990). 87

Supplementary Figure 9: In this analysis, the Spearman correlation was assessed between the IBD incidence rate and the intake of 8 dietary components across 185 countries. (1990) 88

Supplementary Figure 10: In this analysis, the Spearman correlation was assessed between the IBD prevanlence rate and the intake of 8 dietary components across 185 countries. (1990) 89

Supplementary Figure 11: In this analysis, the Spearman correlation was assessed between the IBD incidence rate and the intake of 8 dietary components across 185 countries. (2018) 90

Supplementary Figure 12: In this analysis, the Spearman correlation was assessed between the IBD prevalence rate and the intake of 8 dietary components across 185 countries. (2018) 91

Supplementary Table 1: National mean (95% UI) intakes of eight nutrients in 2018 across 185 countries and regions.

Country/Region	Fruits	Non-starchy vegetables	Total processed meats	Unprocessed red meats	Saturated fat	Monounsaturated fatty acids	Total omega-6 fat	Dietary fiber
**Afghanistan**	64.5(55.1 - 75.7)	98.6(76.8 - 125.8)	1.79 (1.58-2.03)	7.6 (6.07-9.44)	7.6(7 - 8.2)	5.0(4.7 - 5.3)	2.6(2.6 - 2.6)	48.2(43.3 - 53.5)
**Albania**	129.6(113.2 - 148.2)	121.6(96.1 - 154.1)	66.61 (57.76-76.8)	57.98 (47.27-68.49)	11.8(11.3 - 12.2)	14.2(13.5 - 14.9)	2.4(2.4 - 2.5)	28.1(25.5 - 31.1)
**Algeria**	85.9(70.3 - 105.3)	214.1(164.2 - 276.9)	7.14 (5.77-8.49)	36.99 (28.1-48.35)	12.7(12.4 - 13)	15.6(14.9 - 16.4)	2.6(2.6 - 2.6)	24.7(21.3 - 28.8)
**Angola**	121.3(101 - 144.9)	328.5(268.5 - 401.7)	1.32 (1.08-1.56)	29.59 (20.74-41.53)	11(10.7 - 11.3)	10.5(10.3 - 10.7)	2.7(2.6 - 2.7)	29.7(26.4 - 33.4)
**Antigua and Barbuda**	95.6(82.3 - 111.3)	230.5(190.3 - 278.2)	33.81 (28.51-39.05)	31.42 (24.41-40.26)	10(9.2 - 10.8)	8.1(7.6 - 8.5)	2.6(2.6 - 2.7)	24.1(21.7 - 26.6)
**Argentina**	94.1(81 - 109)	123.8(102.7 - 149.2)	20.04 (16.55-23.57)	87.17 (67.32-111.95)	11.7(10.9 - 12.6)	11.0(10.5 - 11.6)	2.6(2.6 - 2.7)	11.5(10.3 - 12.7)
**Armenia**	85.8(74.6 - 98.4)	91(72.1 - 114.9)	100.93 (91.17-111.89)	56.45 (44.43-71.95)	11.4(10.9 - 11.8)	18.8(17.9 - 19.7)	2.5(2.5 - 2.6)	32.8(29.6 - 36.2)
**Australia**	141.4(125.9 - 156.9)	113.1(95.3 - 133.8)	14.65 (12.42-17.21)	52.36 (42.39-64.69)	13.1(12.8 - 13.5)	12.2(12 - 12.4)	2.7(2.6 - 2.7)	18.8(16.6 - 21.3)
**Austria**	109.6(97.2 - 121.4)	110.3(93.2 - 130.9)	50.75 (43.14-59.98)	92.82 (74.37-115.34)	16.6(16.2 - 17)	13.3(13.2 - 13.5)	2.8(2.8 - 2.8)	21.0(18.6 - 23.8)
**Azerbaijan**	100(87.2 - 114.8)	76.3(60.4 - 96.6)	94.59 (84.49-106.08)	82.42 (67.48-97.05)	8.9(8.5 - 9.3)	20.5(19.6 - 21.4)	2.4(2.4 - 2.5)	16.7(15.1 - 18.5)
**Bahrain**	123.3(100.8 - 151.4)	188.7(144.5 - 244.5)	18.39 (14.96-21.84)	37.17 (28.27-48.81)	10.3(10.1 - 10.5)	13(12.3 - 13.7)	2.7(2.7 - 2.7)	8.9(7.7 - 10.3)
**Bangladesh**	53(45.2 - 62.2)	212.3(165.5 - 273.5)	0.37 (0.33-0.42)	7.02 (5.61-8.72)	3.4(3 - 3.8)	3.9(3.6 - 4.1)	2.4(2.4 - 2.4)	23.6(21.1 - 26.4)
**Barbados**	127(108.7 - 148.1)	138.1(115 - 166.3)	10.69 (8.84-12.54)	64.6 (50.18-82.81)	9.5(8.7 - 10.3)	8.3(7.9 - 8.8)	2.8(2.8 - 2.9)	36.5(33.0 - 40.5)
**Belarus**	229.3(199.9 - 263)	79.1(62.5 - 100.1)	33.69 (28.33-40.03)	64.3 (52.05-76.15)	13.5(13.1 - 14)	13.4(12.6 - 14.1)	2.6(2.5 - 2.6)	29.5(26.7 - 32.6)
**Belgium**	108.9(96.9 - 120.9)	107.5(90.6 - 127.6)	25.71 (21.86-30.23)	51.82 (41.88-64.08)	15.6(15.2 - 16)	13.5(13.3 - 13.6)	2.7(2.7 - 2.7)	16.5(14.5 - 18.6)
**Belize**	86.3(74.1 - 101)	151.6(125.3 - 182)	46.97 (40.56-53.41)	100.57 (78.46-128.31)	7.9(7.2 - 8.6)	10.7(10.2 - 11.2)	2.6(2.5 - 2.6)	15.2(13.7 - 16.8)
**Benin**	75(62.3 - 89.6)	103.7(83.9 - 127.6)	18.9 (15.57-22.28)	28.49 (20.15-40.11)	8.2(7.9 - 8.5)	14.1(13.8 - 14.3)	2.6(2.6 - 2.6)	14.4(12.7 - 16.2)
**Bhutan**	93.9(80 - 109.8)	275.3(214.2 - 353.4)	1.52 (1.34-1.72)	16.49 (13.26-20.52)	7(6.5 - 7.6)	6.2(5.9 - 6.6)	2.6(2.6 - 2.6)	31.4(28.1 – 35.0)
**Bolivia**	87.2(75.2 - 100.5)	115.1(95.6 - 139.2)	12.71 (10.62-14.89)	95.52 (74.48-122.21)	11.8(10.9 - 12.6)	11.1(10.6 - 11.7)	2.6(2.5 - 2.6)	8.6(7.8 - 9.5)
**Bosnia and Herzegovina**	471.7(420.6 - 528.7)	408.3(327.2 - 509.2)	24.28 (20.36-29.01)	134.29 (105.57-169.88)	9.5(9.1 - 9.9)	18.1(17.3 - 19)	2.5(2.4 - 2.5)	17.0(15.4 - 18.9)
**Botswana**	49.4(41 - 59.6)	112.8(91.7 - 138.4)	7.88 (6.36-9.35)	43.33 (30.38-60.59)	8.8(8.6 - 9.1)	11.8(11.5 - 12)	2.7(2.7 - 2.7)	37.6(33.4 - 42.2)
**Brazil**	85(72.8 - 98.7)	92.5(76.4 - 112.3)	34.64 (28.68-40.61)	71.23 (55.64-91.18)	10.6(9.8 - 11.4)	10.5(10 - 11)	2.7(2.7 - 2.7)	27.4(24.8 - 30.3)
**Brunei**	89.7(79.2 - 99.9)	160(138.9 - 184.3)	43.74 (35.38-54.14)	75.83 (61.95-89.84)	11.9(10.9 - 12.9)	9.1(8 - 10.1)	2.5(2.5 - 2.6)	15.7(13.2 - 18.6)
**Bulgaria**	88(76.5 - 101.4)	353.1(280.2 - 440.1)	29.69 (24.84-35.51)	61.79 (50.19-73.16)	12.6(12.1 - 13.1)	9.4(8.9 - 10)	3.0(3.0- 3.1)	18.5(16.8 - 20.5)
**Burkina Faso**	35.1(29.2 - 42)	28.7(23.3 - 35.2)	5.69 (4.61-6.75)	4.71 (3.32-6.63)	8.1(7.8 - 8.3)	14.1(13.9 - 14.4)	2.6(2.6 - 2.7)	15.6(13.8 - 17.6)
**Burundi**	74.8(62.7 - 89.3)	199.8(163.6 - 246.3)	3.78 (3.1-4.46)	20.11 (14.45-28.14)	9.3(9.1 - 9.6)	11.6(11.4 - 11.8)	2.5(2.5 - 2.5)	27.6(24.5 - 31.2)
**Cambodia**	53.4(47.1 - 59.7)	120.4(103.5 - 139.6)	6.9 (5.18-9.25)	20.12 (16.42-23.86)	12.1(11.1 - 13.1)	7.2(6.3 - 8.1)	2.6(2.6 - 2.6)	5.9(5.0 – 7.0)

**Cameroon**	83.3(69.4 - 99.6)	130.4(106.2 - 160.4)	2.27 (1.86-2.67)	21.35 (14.98-30)	4.7(4.5 - 4.9)	15.5(15.3 - 15.8)	2.6(2.6 - 2.6)	31.4(27.9 - 35.5)
**Canada**	142.8(127.3 - 158.6)	124.5(104.6 - 147.4)	21.14 (17.96-24.98)	39.79 (32.24-49.28)	11.2(10.9 - 11.6)	11.9(11.8 - 12.1)	2.5(2.5 - 2.5)	14.8(13.1 - 16.7)
**Cape Verde**	226.4(189.1 - 271.6)	190.5(155.6 - 232.9)	5.02 (4.08-5.95)	32 (22.68-44.93)	11.8(11.5 - 12.1)	12.4(12.1 - 12.7)	2.7(2.7 - 2.7)	59.3(53.7 - 65.7)
**Central African Republic**	97.9(81.9 - 116.7)	107.5(87.4 - 132.1)	4 (3.26-4.73)	118.34 (83.8-151.55)	9.4(9.2 - 9.7)	10.1(9.9 - 10.3)	2.7(2.7 - 2.7)	24.3(21.5 - 27.4)
**Chad**	43.2(36.1 - 51.6)	39.2(32 - 48.2)	3.13 (2.57-3.71)	52.44 (37.01-73.55)	11.9(11.6 - 12.2)	10.6(10.4 - 10.9)	2.6(2.6 - 2.6)	15.6(13.9 - 17.6)
**Chile**	108.2(93.1 - 125.5)	137.7(114.3 - 165.7)	45.22 (37.65-52.79)	19.52 (15.29-24.91)	11.3(10.5 - 12.2)	10.7(10.2 - 11.2)	3.2(3.1 - 3.2)	21.1(19.1 - 23.4)
**China**	95.9(83.9 - 108.2)	294.9(253 - 342.3)	4.15 (3.13-5.51)	92.81 (75-110.37)	9.6(8.7 - 10.5)	8.3(7.3 - 9.3)	2.9(2.9 - 2.9)	20.7(17.5 - 24.6)
**Colombia**	100.3(86.2 - 116.7)	96(79.3 - 115.2)	63.13 (52.72-73.6)	97.42 (75.72-125.04)	9.3(8.5 - 10)	12.4(11.8 - 13)	2.7(2.6 - 2.7)	17.2(15.6 - 19)
**Comoros**	84.6(70.3 - 101.4)	100.2(81.1 - 123.2)	4.28 (3.52-5.06)	39.38 (27.55-55.54)	9.5(9.2 - 9.8)	11.7(11.4 - 11.9)	2.5(2.5 - 2.6)	22.0(19.5 - 24.9)
**Congo**	89.8(74.4 - 107.6)	148(120 - 182.3)	21.47 (17.52-25.19)	37.24 (26.29-52.65)	9.9(9.6 - 10.2)	14.5(14.2 - 14.7)	2.5(2.5 - 2.5)	27.3(24.1 - 30.7)
**Costa Rica**	130.8(112.2 - 151.7)	164.7(136.1 - 200.4)	38.69 (33.01-44.56)	73.98 (57.75-94.89)	11.5(10.6 - 12.3)	10.4(9.9 - 11)	2.6(2.6 - 2.6)	22.5(20.4 - 24.9)
**Cote d'Ivoire**	60.5(50.3 - 72.3)	96.8(78.6 - 119.8)	3.93 (3.21-4.65)	21.36 (15.2-29.67)	10.2(9.9 - 10.5)	13.8(13.6 - 14.1)	2.5(2.5 - 2.5)	21.2(18.8 - 23.9)
**Croatia**	54.9(47.9 - 62.7)	445.8(372.5 - 531.5)	100.71 (85.37-119.53)	233.93 (191.98-284.78)	13.1(12.6 - 13.6)	12.6(11.9 - 13.3)	2.5(2.5 - 2.6)	33.9(30.7 - 37.4)
**Cuba**	127.8(110.1 - 148.2)	110.3(91.1 - 133)	35.09 (29.64-40.53)	142.3 (112.88-178.26)	7.1(6.5 - 7.8)	14.2(13.6 - 14.9)	2.6(2.5 - 2.6)	6.6(6.0 - 7.2)
**Cyprus**	64.2(56.9 - 71.6)	114.2(96.5 - 135)	8.72 (7.41-10.27)	63.91 (51.86-78.9)	12.4(12.1 - 12.7)	13.6(13.4 - 13.8)	2.7(2.7 - 2.8)	11.2(9.9 - 12.6)
**Czech Republic**	68.4(59.4 - 78.4)	94.6(75.2 - 119)	36.12 (30.46-42.77)	55.88 (43.9-70.71)	13.7(13.2 - 14.1)	10.9(10.3 - 11.6)	2.6(2.5 - 2.6)	16.3(14.7 - 18)
**Democratic Republic of the Congo**	96.6(80.8 - 115.8)	227.9(184.8 - 278.8)	22.78 (18.78-26.9)	19.23 (13.61-26.83)	16.9(16.5 - 17.3)	16.4(16.1 - 16.7)	2.6(2.6 - 2.6)	23.1(20.4 - 26.1)
**Denmark**	94.6(84 - 105.5)	114.9(96.8 - 135.7)	30.93 (26.33-36.37)	47.04 (38.12-57.72)	14.1(13.7 - 14.4)	14.3(14.1 - 14.5)	2.6(2.6 - 2.6)	21.8(19.3 - 24.7)
**Djibouti**	138.3(115.7 - 165.1)	103.2(84.1 - 126.8)	16.21 (13.26-19.15)	33.16 (23.52-46.51)	10.6(10.3 - 10.9)	11.3(11.1 - 11.5)	2.6(2.6 - 2.6)	42.7(38.1 - 48)
**Dominica**	106(91.3 - 123.4)	102.6(84.6 - 124.5)	9.61 (8-11.24)	23.19 (18.12-29.72)	11.3(10.5 - 12.1)	12.7(12.1 - 13.3)	2.5(2.4 - 2.5)	15.0(13.6 - 16.5)
**Dominican Republic**	144.1(123.7 - 167)	104.7(86.6 - 125.7)	29.17 (24.54-33.81)	49.63 (38.85-63.54)	11.2(10.4 - 12.1)	10.3(9.8 - 10.9)	2.7(2.7 - 2.7)	17.6(15.9 - 19.4)
**Ecuador**	26.3(22.5 - 30.5)	110.6(91.5 - 134.2)	3.35 (2.76-3.93)	52.53 (40.87-67.39)	13.7(12.8 - 14.6)	8.6(8.2 - 9.1)	2.6(2.6 - 2.6)	19.7(17.8 - 21.8)
**Egypt**	97.5(79.2 - 118.8)	168.2(128.6 - 219.8)	5.49 (4.42-6.53)	29.39 (22.5-38.08)	7.7(7.5 - 7.9)	18.6(17.7 - 19.5)	2.6(2.6 - 2.6)	39.4(34.0 - 45.6)
**El Salvador**	124.2(106.6 - 144.4)	129.5(106.6 - 157.1)	40.66 (34.64-46.64)	47.54 (36.77-60.92)	8.1(7.4 - 8.8)	10.9(10.4 - 11.4)	2.6(2.6 - 2.6)	11.3(10.2 - 12.5)
**Equatorial Guinea**	89.1(74.4 - 106.5)	148.8(121.5 - 182.3)	3.44 (2.82-4.05)	42.39 (29.96-59.65)	10.5(10.2 - 10.8)	11.9(11.7 - 12.2)	2.7(2.6 - 2.7)	29.3(26.1 - 32.9)
**Eritrea**	56.6(47.3 - 67.4)	71.6(57.9 - 87.7)	3.79 (3.12-4.46)	36.5 (25.73-50.96)	9.6(9.3 - 9.9)	11.7(11.5 - 11.9)	2.6(2.5 - 2.6)	29.3(26.0 – 33.0)
**Estonia**	98.1(85.6 - 112.7)	266.7(211.7 - 336.5)	77.94 (65.43-93.22)	66.14 (53.96-78.39)	13.3(12.9 - 13.8)	12.3(11.6 - 13)	2.5(2.4 - 2.5)	16.2(14.6 – 18.0)
**Ethiopia**	38.2(31.9 - 45.8)	91.9(74.7 - 112)	17.71 (14.65-20.83)	16.78 (11.79-23.81)	6.9(6.7 - 7.1)	20.1(19.8 - 20.5)	2.4(2.4 - 2.4)	16.6(14.7 - 18.7)
**Federated States of Micronesia**	158.6(138.8 - 179.1)	141.1(121.5 - 163.5)	19.53 (14.86-25.62)	26.78 (21.81-31.74)	13.3(12.2 - 14.2)	8.0(7.0 - 8.9)	2.6(2.5 - 2.6)	9.3(7.9 - 11.1)
**Fiji**	118.7(104.5 - 132.7)	212.6(183.2 - 245.9)	8.58 (6.45-11.38)	17.23 (13.88-20.59)	16.9(15.7 - 18)	10.3(9.2 - 11.4)	2.5(2.5 - 2.5)	11.6(9.7 - 13.8)

**Finland**	156.4(138.7 - 174)	118.1(99.7 - 139.5)	52.51 (44.54-61.75)	54.46 (44.07-67.4)	14.2(13.8 - 14.6)	11.4(11.3 - 11.6)	2.5(2.5 - 2.5)	19.8(17.5 - 22.4)
**France**	157.1(142.3 - 173.1)	147.7(124.6 - 174.9)	28.27 (23.94-33.34)	43.25 (34.83-53.76)	17.1(16.7 - 17.5)	13.6(13.4 - 13.7)	2.7(2.7 - 2.7)	16.3(14.4 - 18.4)
**Gabon**	69.3(57.6 - 82.5)	126.2(102 - 155.3)	5.62 (4.59-6.65)	53.58 (37.71-75.36)	11.6(11.3 - 11.9)	13.5(13.2 - 13.7)	2.7(2.7 - 2.7)	48.3(43.2 - 54.1)
**Georgia**	67.9(59.1 - 77.5)	82.5(65.3 - 103.6)	60.57 (52.03-70.25)	13.35 (11.02-15.78)	9.0(8.7 - 9.4)	16.1(15.4 - 16.9)	2.5(2.5 - 2.5)	18.6(16.8 - 20.5)
**Germany**	143.7(127.5 - 159.8)	178(150.4 - 211)	56.15 (47.45-66.26)	42.23 (33.81-52.42)	15.3(14.9 - 15.7)	12.5(12.3 - 12.7)	2.6(2.6 - 2.7)	23.8(21.0 - 26.8)
**Ghana**	86.4(71.7 - 103.2)	140.3(114.2 - 172.5)	2.02 (1.64-2.39)	23.42 (16.36-33.28)	7.5(7.2 - 7.7)	8.2(8.0 - 8.4)	2.6(2.6 - 2.6)	28.0(24.8 - 31.5)
**Greece**	78.7(70 - 87.4)	83.7(70.8 - 99)	4.99 (4.22-5.87)	68.75 (55.23-84.95)	14.8(14.4 - 15.2)	12.7(12.5 - 12.9)	2.7(2.7 - 2.8)	29.3(25.8 - 33.1)
**Grenada**	121.6(104.8 - 140.8)	252.1(209.1 - 304.2)	7.71 (6.44-9.05)	23.07 (18.1-29.38)	12(11.2 - 12.9)	7.2(6.8 - 7.6)	2.6(2.6 - 2.7)	19.6(17.7 - 21.7)
**Guatemala**	77.1(66.3 - 89.8)	95.7(79.4 - 114.9)	34.32 (28.92-39.59)	59.88 (46.64-76.25)	5.2(4.7 - 5.8)	8.0(7.6 - 8.5)	2.7(2.7 - 2.7)	24.3(21.9 - 26.9)
**Guinea**	67.2(55.9 - 80.3)	142.6(116.2 - 175.1)	3.58 (2.91-4.24)	34.34 (24.33-48.7)	11.6(11.3 - 11.9)	10.1(9.9 - 10.3)	2.6(2.6 - 2.6)	11.6(10.2 - 13.1)
**Guinea-Bissau**	22.0(18.4 - 26.3)	21.1(17.2 - 25.8)	12.45 (10.16-14.78)	21.99 (15.72-30.75)	9.8(9.5 - 10.1)	12.4(12.1 - 12.6)	2.5(2.5 - 2.6)	20.9(18.5 - 23.5)
**Guyana**	204.7(175.9 - 237.9)	147.6(121.4 - 178.2)	61.54 (53.96-69.19)	45.61 (35.58-58.31)	9.8(9.1 - 10.6)	15.4(14.8 - 16.1)	2.4(2.3 - 2.4)	8.5(7.7 - 9.4)
**Haiti**	71.2(61.4 - 82.6)	82.9(68.2 - 100.2)	13.2 (10.92-15.4)	16.17 (12.6-20.65)	7.0(6.4 - 7.6)	15.7(15 - 16.5)	2.6(2.6 - 2.6)	8.1(7.3 – 9.0)
**Honduras**	89.7(77.3 - 104)	30.3(25.1 - 36.8)	48.74 (41.96-55.41)	31.33 (24.36-40.32)	10(9.2 - 10.8)	10.5(10 - 11)	2.5(2.5 - 2.6)	17.4(15.8 - 19.2)
**Hungary**	55.2(48 - 63.5)	55.4(43.5 - 69.8)	10.69 (8.92-12.81)	18.93 (14.84-24.31)	13.8(13.3 - 14.3)	10.4(9.8 - 11)	2.9(2.8 - 2.9)	9.2(8.4 - 10.2)
**Iceland**	82.4(73.2 - 91.6)	67.8(57.3 - 80.2)	21.95 (18.64-25.78)	54.29 (43.75-67.41)	16.5(16.1 - 16.9)	15.6(15.4 - 15.7)	2.5(2.5 - 2.5)	14.3(12.6 - 16.2)
**India**	34.5(29.5 - 40.3)	157.3(121.8 - 202.1)	2.43 (2.15-2.73)	3.02 (2.42-3.77)	6.4(5.9 - 7.1)	7(6.6 - 7.4)	2.8(2.8 - 2.8)	28.5(25.5 - 31.7)
**Indonesia**	72.9(64.1 - 81.8)	160.4(138.3 - 185.9)	26.58 (20.62-34.23)	20.72 (16.88-24.66)	16.2(15 - 17.3)	9.5(8.5 - 10.6)	2.6(2.6 - 2.6)	11.6(9.8 - 13.8)
**Iran**	176.3(143.5 - 217.2)	239.4(183.1 - 310.6)	5.26 (4.26-6.26)	15.62 (11.85-20.46)	9.9(9.7 - 10.2)	9.0(8.4 - 9.6)	2.8(2.8 - 2.9)	24.2(20.7 - 28)
**Iraq**	116.3(94.6 - 142.1)	137.1(104.5 - 178.8)	30.33 (25.06-35.71)	50.08 (37.94-65.48)	9.5(9.2 - 9.7)	15.6(14.7 - 16.4)	2.7(2.7 - 2.8)	15.3(13.2 - 17.7)
**Ireland**	85.0(75.6 - 94.2)	114.4(97 - 135.1)	32.08 (27.27-37.62)	33.44 (27.05-41.25)	15.3(15 - 15.7)	13.4(13.2 - 13.5)	2.6(2.6 - 2.6)	12.5(11 - 14.1)
**Israel**	140.4(114.2 - 172.4)	88.7(68 - 115.7)	74.95 (61.35-88.92)	125.61 (96.4-163.25)	8.2(8 - 8.5)	14.8(14 - 15.6)	2.7(2.6 - 2.7)	10.1(8.7 - 11.7)
**Italy**	201.5(179.2 - 224.1)	136.1(115.3 - 161.3)	18.4 (15.53-21.63)	46.73 (37.67-57.63)	11.8(11.5 - 12.2)	15(14.9 - 15.2)	2.5(2.5 - 2.5)	17.3(15.3 - 19.6)
**Jamaica**	176.0(151.7 - 204.1)	148.5(122.8 - 179.2)	11.24 (9.29-13.15)	12.23 (9.5-15.72)	6.0(5.4 - 6.6)	9.6(9.1 - 10)	2.8(2.8 - 2.9)	14.1(12.7 - 15.6)
**Japan**	110.3(96.3 - 124.5)	264.8(228.2 – 308.0)	15.68 (11.77-20.77)	81.13 (65.83-96.34)	8.5(7.7 - 9.3)	11.2(9.9 - 12.4)	2.6(2.6 - 2.7)	11.2(9.4 - 13.3)
**Jordan**	187.3(152.7 - 229.6)	132.0(101.0 - 172.7)	31.1 (25.74-36.49)	51.11 (39.11-66.63)	8.2(8 - 8.4)	6.1(5.6 - 6.6)	2.7(2.6 - 2.7)	54.5(47.2 - 62.9)
**Kazakhstan**	58.0(50.5 - 66.7)	161.0(128.0 - 203.4)	40.09 (34.11-47.23)	86.22 (67.94-109.72)	12.5(12 - 12.9)	13.7(13 - 14.5)	2.6(2.5 - 2.6)	27.2(24.5 - 30)
**Kenya**	103.3(86 - 123.8)	252.7(206.0 - 309.8)	0.69 (0.56-0.81)	24.07 (16.87-34.13)	10.5(10.2 - 10.8)	13.9(13.6 - 14.1)	2.5(2.5 - 2.5)	24.0(21.4 - 27.1)
**Kiribati**	108.2(94.7 - 121.6)	91.8(79.4 - 106.1)	20.43 (15.6-26.8)	28.76 (23.28-34.22)	12.5(11.4 - 13.4)	7.7(6.8 - 8.6)	2.6(2.6 - 2.6)	10.0(8.4 – 12.0)
**Kuwait**	59.2(48 - 72.8)	113(86.2 - 146.7)	28.62 (23.6-33.79)	26.27 (20.11-33.98)	11(10.7 - 11.3)	10.1(9.5 - 10.7)	2.6(2.6 - 2.6)	27.1(23.3 - 31.3)

**Kyrgyzstan**	75.1(65.2 - 86.3)	41.4(32.8 - 52.4)	22.57 (18.94-26.94)	34.85 (27.39-44.05)	10.4(9.9 - 10.8)	21.1(20.1 - 22.1)	2.5(2.4 - 2.5)	8.0(7.3 - 8.8)
**Laos**	130.1(114.2 - 146.3)	143.9(123.9 - 167.3)	12.44 (9.35-16.47)	32.3 (26.22-38.46)	9.8(8.9 - 10.7)	6.3(5.6 – 7.0)	2.5(2.5 - 2.5)	10.2(8.6 - 12.2)
**Latvia**	55(47.7 - 63.2)	210.7(167.2 - 266.2)	52.97 (44.17-63.18)	180.15 (141.98-228.62)	14.5(14.0 – 15.0)	12(11.3 - 12.7)	2.6(2.5 - 2.6)	31.0(28 - 34.2)
**Lebanon**	238(193.5 - 294.1)	370.1(282.9 - 484.2)	8.94 (7.26-10.66)	22.34 (17.04-29.18)	11.0(10.7 - 11.2)	21.4(20.4 - 22.3)	3.0(3.0 - 3.1)	30.4(26.3 - 35.1)
**Lesotho**	66.8(55.5 - 80.1)	191.5(154.8 - 235.9)	3.53 (2.88-4.19)	40.05 (28.31-56.21)	12(11.7 - 12.4)	10.6(10.4 - 10.8)	2.6(2.6 - 2.6)	40.8(36.2 - 45.9)
**Liberia**	49.2(40.9 - 59.2)	125.6(101.2 - 155.1)	36.14 (30.28-42.01)	39.13 (27.96-55.04)	12.2(11.9 - 12.5)	10.7(10.5 - 11)	2.6(2.6 - 2.7)	21.3(18.9 - 23.9)
**Libya**	56.1(45.5 - 69.1)	121.5(92.3 - 159)	11.93 (9.61-14.25)	34.37 (26.09-45.05)	12.8(12.5 - 13)	12.5(11.8 - 13.2)	2.6(2.5 - 2.6)	28.2(24.2 - 32.8)
**Lithuania**	64.4(56.1 - 74.1)	117.7(92.3 - 149.8)	39.07 (33.14-46.19)	126.84 (103.68-150.14)	13.5(13 - 14)	14.1(13.4 - 14.9)	2.5(2.4 - 2.5)	16.5(14.9 - 18.2)
**Luxembourg**	91.3(81.1 - 101.2)	90.3(76.4 - 106.8)	85.55 (72.69-100.5)	86.68 (70.04-106.67)	12.5(12.1 - 12.8)	13.1(13 - 13.3)	2.6(2.6 - 2.6)	36.6(32.4 - 41.3)
**Macedonia**	84.9(73.7 - 97.4)	137.9(109.1 - 174.4)	55.91 (47.79-65.2)	43.8 (35.99-51.73)	11.2(10.8 - 11.7)	12.5(11.9 - 13.2)	2.6(2.6 - 2.7)	33.0(29.9 - 36.2)
**Madagascar**	88.8(74 - 105.7)	172(139.6 - 210.9)	4.77 (3.92-5.64)	43.33 (30.51-60.8)	6.8(6.6 - 7.1)	4.7(4.6 - 4.8)	2.4(2.4 - 2.4)	5.6(5.0 - 6.3)
**Malawi**	113.7(94.8 – 136.0)	182(148.7 - 223.1)	4.71 (3.81-5.58)	22.92 (16.25-32.16)	6(5.8 - 6.2)	18.8(18.4 - 19.1)	2.5(2.5 - 2.6)	18.3(16.3 - 20.7)
**Malaysia**	114.4(101.3 - 127.7)	161.4(138.9 - 187.1)	12.63 (9.52-16.82)	16.58 (13.41-19.77)	11.4(10.4 - 12.4)	10.1(9 - 11.2)	2.5(2.5 - 2.5)	22.2(18.6 - 26.4)
**Maldives**	221(189.3 - 257.7)	159.6(124.5 - 204.7)	14.09 (12.46-15.94)	26.02 (20.81-32.54)	7.2(6.7 - 7.9)	7.7(7.4 - 8.1)	2.5(2.5 - 2.5)	60.9(55.3 - 67)
**Mali**	71.4(59.4 - 85.6)	97.1(79.6 - 118.3)	7.76 (6.32-9.19)	34.17 (24.05-47.88)	9.1(8.9 - 9.4)	13.9(13.6 - 14.2)	2.6(2.6 - 2.6)	53(47.2 - 59.2)
**Malta**	137.9(122.4 - 153.1)	84(70.7 - 99.8)	34.01 (28.76-40.03)	54.94 (44.37-68.03)	13.9(13.5 - 14.2)	12.2(12 - 12.4)	2.6(2.6 - 2.6)	22.3(19.7 - 25.2)
**Marshall Islands**	120.4(105 - 135.5)	186.1(160.5 - 215.2)	8.87 (6.69-11.81)	26.94 (21.79-32.04)	13.4(12.3 - 14.4)	8.1(7.2 - 9.1)	2.6(2.6 - 2.6)	12.9(10.8 - 15.4)
**Mauritania**	34.6(28.9 - 41.3)	231.9(188.1 - 286.2)	34.83 (29.4-40.55)	29.48 (20.87-41.43)	12.2(11.9 - 12.6)	12.7(12.5 - 13)	2.6(2.6 - 2.6)	51.9(46.4 - 58)
**Mauritius**	71.5(59.7 - 85.8)	171.6(139.7 - 210.1)	31.41 (25.76-37.18)	61.17 (43.11-86.41)	9.9(9.6 - 10.2)	11.2(11 - 11.4)	2.8(2.8 - 2.8)	52.3(46.8 - 58.4)
**Mexico**	150.9(129.9 - 175.5)	196.2(163.1 - 237)	15.01 (12.47-17.5)	46.93 (36.53-60.36)	11.1(10.3 - 12)	10.3(9.8 - 10.8)	2.3(2.3 - 2.4)	9.4(8.5 - 10.4)
**Moldova**	90.7(78.7 - 104.4)	82.1(65.2 - 103.1)	60.04 (51.6-70.03)	78.4 (63.8-92.28)	10.9(10.4 - 11.3)	17.9(17 - 18.8)	2.6(2.5 - 2.6)	11.8(10.7 - 13.1)
**Mongolia**	48.2(42 - 55.4)	180(143.2 - 226.2)	119.58 (112.46-126.98)	71.4 (58.57-84.52)	13.2(12.8 - 13.7)	15.3(14.5 - 16.1)	2.5(2.5 - 2.6)	21.6(19.5 - 23.8)
**Montenegro**	229.0(200.3 - 262.6)	125.8(100.3 - 157.8)	57.43 (48.02-68.44)	179.26 (140.51-227.16)	12.3(11.9 - 12.8)	8.7(8.2 - 9.2)	2.5(2.5 - 2.6)	58.0(53 - 63.4)
**Morocco**	102.1(83.2 - 125.3)	178.5(136.7 - 232.5)	19.8 (16.19-23.41)	24.81 (19.02-32.06)	7.8(7.5 - 8)	13.5(12.7 - 14.2)	2.5(2.5 - 2.5)	43.2(37.3 - 50.1)
**Mozambique**	103.7(86.4 - 124.5)	146.9(119 - 181.5)	3.56 (2.89-4.23)	32.52 (23.12-45.82)	7.8(7.5 - 8)	5.1(5 - 5.2)	2.5(2.5 - 2.6)	33.6(29.8 - 37.9)
**Myanmar**	85.8(75.3 - 96.5)	173.3(149.1 - 201.2)	27.88 (21.63-35.77)	80.73 (65.99-95.61)	12.2(11.2 - 13.2)	7.3(6.4 - 8.2)	2.6(2.6 - 2.6)	18.6(15.6 – 22.0)
**Namibia**	247.9(207.8 - 296.3)	152.9(124.4 - 187.6)	6.13 (5.03-7.27)	38.93 (27.7-54.22)	8.6(8.4 - 8.9)	13.9(13.6 - 14.1)	2.6(2.6 - 2.6)	37.9(33.6 - 42.7)
**Nepal**	36.5(31.3 - 42.6)	184(142.7 - 234.8)	0.68 (0.6-0.77)	10.64 (8.51-13.25)	3(2.7 - 3.4)	4.2(3.9 - 4.4)	2.3(2.3 - 2.3)	48.8(43.6 - 54.4)
**Netherlands**	110.9(98.7 - 123.4)	110.3(93.1 - 129.7)	20.49 (17.46-24.05)	40.71 (32.62-50.65)	13.7(13.4 - 14.1)	11.9(11.8 - 12.1)	2.7(2.7 - 2.7)	12.4(10.9 – 14.0)
**New Zealand**	164.5(145.9 - 182.8)	125.6(106.8 - 147.5)	29.81 (25.29-35.18)	48.37 (39.03-59.98)	16.1(15.7 - 16.5)	15.1(14.9 - 15.3)	2.7(2.7 - 2.7)	17.9(15.7 - 20.2)

**Nicaragua**	157.5(135 - 182.5)	276.8(230.1 - 332.3)	41.39 (35.38-47.43)	36.91 (28.78-47.18)	8.2(7.5 - 8.9)	11.8(11.3 - 12.4)	2.6(2.5 - 2.6)	10.5(9.5 - 11.6)
**Niger**	66.1(55.3 - 78.8)	108(88 - 132.6)	2.9 (2.36-3.43)	6.35 (4.51-8.93)	9(8.7 - 9.2)	13.5(13.3 - 13.8)	2.6(2.6 - 2.6)	30.1(26.6 - 33.9)
**Nigeria**	35.5(29.6 - 42.5)	97.8(79.5 - 119.7)	4.83 (3.93-5.71)	33.61 (23.91-47.25)	9.8(9.5 – 10.0)	12.4(12.2 - 12.6)	2.5(2.5 - 2.6)	17.8(15.8 – 20.0)
**Norway**	87.7(78.1 - 97.6)	126.4(106.8 - 149.6)	24.86 (21.16-29.19)	76.65 (61.65-94.67)	13.8(13.4 - 14.2)	12.8(12.7 - 13)	2.6(2.6 - 2.6)	16.4(14.5 - 18.5)
**Oman**	129.1(104.7 - 158.7)	166.0(127.3 - 215.8)	24.0 (19.57-28.51)	41.2 (31.44-53.41)	10.1(9.9 - 10.3)	12.8(12.1 - 13.6)	2.7(2.7 - 2.7)	30.2(26.1 - 34.9)
**Pakistan**	33.4(28.6 - 39.3)	86.8(67.5 - 110.9)	5.33 (4.73-6.03)	25.09 (20.07-31.44)	9.4(8.8 - 10.2)	6.2(5.9 - 6.5)	2.6(2.6 - 2.6)	16.5(14.8 - 18.4)
**Palestine**	120.3(98 - 148.3)	165.8(126.3 - 218)	15.29 (12.41-18.17)	36.36 (27.72-48.02)	12.3(12.1 - 12.6)	7.1(6.7 - 7.6)	2.7(2.7 - 2.7)	71.9(66.9 - 77.3)
**Panama**	131(112.4 - 152.3)	152.5(125.9 - 184.5)	42.74 (36.66-48.94)	74.93 (58.21-95.85)	8.9(8.2 - 9.6)	12.2(11.6 - 12.8)	2.7(2.6 - 2.7)	15.7(14.2 - 17.3)
**Papua New Guinea**	4.2(3.7 - 4.7)	84.9(73.5 - 97.9)	3.85 (2.88-5.13)	156.66 (132.56-180.83)	13(12 - 14)	7.6(6.7 - 8.5)	2.6(2.5 - 2.6)	3.8(3.2 - 4.5)
**Paraguay**	94.6(81.3 - 110.1)	69.3(57.3 - 84)	24.63 (20.71-28.66)	130.01 (101.49-165.51)	9.8(9.1 - 10.6)	9.5(9 - 10)	2.8(2.7 - 2.8)	10.8(9.8 - 11.9)
**Peru**	229.4(196.6 - 267.3)	96.3(79.6 - 115)	26.96 (22.68-31.28)	108.73 (85.02-139.01)	7.6(7 - 8.3)	15.3(14.7 - 16)	2.6(2.5 - 2.6)	10.5(9.5 - 11.6)
**Philippines**	91.8(80.7 - 102.8)	111.9(95.9 - 129.7)	29.86 (22.4-39.43)	20.05 (16.27-23.89)	21.5(20.3 - 22.7)	11.3(10.1 - 12.5)	2.4(2.4 - 2.4)	8.4(7.1 – 10.0)
**Poland**	215.9(188.8 - 246.5)	159.6(126.5 - 201.1)	54.32 (45.63-64.46)	58.8 (47.81-70.04)	12.9(12.4 - 13.4)	17.7(16.9 - 18.6)	2.5(2.4 - 2.5)	13.7(12.3 - 15.1)
**Portugal**	422.7(387.4 - 461.2)	147.4(124.3 - 173.5)	15.13 (12.79-17.92)	58.26 (46.72-72.5)	9.9(9.6 - 10.2)	11.8(11.6 - 12)	2.7(2.6 - 2.7)	15.8(14.0 - 17.9)
**Qatar**	126.7(102.8 - 156.1)	169.7(129.6 - 219.4)	18.33 (15.02-21.83)	34.13 (26.02-44.34)	10.2(10 - 10.4)	13.0(12.3 - 13.7)	2.7(2.7 - 2.7)	17.5(15.1 - 20.3)
**Romania**	179.2(156.2 - 205.9)	321.5(254.9 - 406.4)	44.04 (36.95-52.33)	60.89 (47.9-77.19)	11.2(10.7 - 11.6)	14.4(13.6 - 15.1)	2.2(2.1 - 2.2)	20.2(18.3 - 22.3)
**Russia**	84.5(73.6 - 96.7)	165.8(131.2 - 208.4)	46.13 (38.74-54.78)	165.47 (129.63-210.19)	11.3(10.9 - 11.8)	14.5(13.8 - 15.3)	2.5(2.5 - 2.6)	20.6(18.6 - 22.8)
**Rwanda**	121.7(101.4 - 145.3)	289.9(235.9 - 355.3)	1.32 (1.08-1.56)	7.85 (5.58-11)	6.7(6.5 - 6.9)	24.2(23.8 - 24.6)	2.4(2.4 - 2.4)	11.6(10.4 - 13.1)
**Saint Lucia**	75.8(65.3 - 88.1)	89.6(74.3 – 108.0)	6.96 (5.78-8.1)	37.62 (29.38-47.82)	10.5(9.7 - 11.3)	7.7(7.3 - 8.1)	2.4(2.4 - 2.5)	14.6(13.2 - 16.2)
**Saint Vincent and the Grenadines**	142.9(122.3 - 165.9)	103.2(85.4 - 124.4)	14.96 (12.5-17.43)	55.8 (43.32-71.5)	9.9(9.2 - 10.6)	9.1(8.6 - 9.6)	2.6(2.5 - 2.6)	19.9(18.0 - 21.9)
**Samoa**	153.9(135.5 - 173.2)	354.9(306.6 - 412.6)	14.85 (11.15-19.76)	23.92 (19.23-28.5)	26.9(25.5 - 28.3)	7.5(6.7 - 8.4)	2.6(2.6 - 2.6)	23.2(19.6 - 27.6)
**Sao Tome and Principe**	128.2(107.4 - 153.6)	37.4(30.6 - 46.2)	15.16 (12.42-17.88)	12.34 (8.79-17.29)	12.4(12.1 - 12.7)	10.8(10.6 - 11)	2.7(2.7 - 2.7)	33.3(29.6 - 37.3)
**Saudi Arabia**	154(125.8 - 188.2)	155.9(118.4 - 205.1)	55.97 (48.02-64.03)	33.15 (25.26-43.38)	11.3(11 - 11.5)	13.4(12.7 - 14.2)	2.7(2.7 - 2.7)	33.6(28.9 - 39.1)
**Senegal**	52.7(43.8 - 63.1)	76.8(62.6 - 93.9)	15.57 (12.76-18.34)	13.28 (9.41-18.71)	9.5(9.2 - 9.8)	13.5(13.2 - 13.8)	2.7(2.7 - 2.8)	44.1(39.2 - 49.4)
**Serbia**	137.3(119.9 - 157.3)	429.6(350.4 - 525.6)	27.66 (23.24-32.88)	64.98 (51.08-82.45)	13.9(13.5 - 14.4)	10.6(10 - 11.2)	2.5(2.5 - 2.6)	33.4(30.2 - 37)
**Seychelles**	103.9(86.9 - 124.1)	209.7(170.3 - 257.2)	10.75 (8.74-12.78)	26.91 (19.33-37.81)	9.8(9.5 - 10.1)	11.8(11.5 - 12)	2.6(2.6 - 2.6)	22.6(20.0 - 25.4)
**Sierra Leone**	88.7(74.2 - 105.8)	123.3(100.3 - 152)	55 (47.76-62.36)	16.7 (11.7-23.71)	12.1(11.8 - 12.4)	10.7(10.5 - 11)	2.6(2.6 - 2.6)	21.9(19.4 - 24.7)
**Singapore**	143.2(125.6 - 160.7)	143.6(123.4 - 166.3)	15.22 (11.4-20.2)	39.58 (32.09-46.92)	11.7(10.7 - 12.7)	7.7(6.7 - 8.6)	2.6(2.6 - 2.7)	18.9(15.9 - 22.4)
**Slovakia**	73.9(64.4 - 84.4)	91(72.3 - 113.6)	50.07 (41.8-59.91)	28.93 (23.46-34.41)	12.8(12.4 - 13.3)	11.2(10.5 - 11.8)	2.5(2.5 - 2.6)	26.7(24.1 - 29.6)
**Slovenia**	70.9(61.8 - 81.2)	102.6(80.9 - 129.6)	31.59 (26.43-37.58)	49.04 (39.81-58.1)	13(12.6 - 13.5)	11.1(10.5 - 11.7)	2.6(2.5 - 2.6)	19.7(17.8 - 21.8)

**Solomon Islands**	65.7(57.8 - 73.4)	184.5(159.2 - 213.9)	16.19 (12.21-21.17)	39.24 (31.81-46.6)	12.4(11.4 - 13.4)	7.5(6.6 - 8.5)	2.6(2.6 - 2.6)	4.0(3.3 - 4.7)
**South Africa**	31.4(26.1 - 37.6)	130.1(105.9 - 159.7)	13.75 (11.24-16.26)	169.67 (135.18-204.7)	11.4(11.1 - 11.7)	12.1(11.8 - 12.3)	2.8(2.8 - 2.9)	19.1(17 - 21.4)
**South Korea**	102.4(88.6 - 116.3)	130(112.6 - 150.6)	1.95 (1.47-2.58)	36.05 (29.17-42.83)	6.5(5.8 - 7.1)	6.3(5.5 - 7.1)	2.4(2.4 - 2.5)	10.6(8.9 - 12.6)
**South Sudan**	65.1(54.5 - 77.8)	148.4(120.7 - 182.2)	3.99 (3.28-4.71)	31.73 (22.48-44.48)	9.4(9.2 - 9.7)	11.6(11.4 - 11.8)	2.5(2.5 - 2.5)	32.2(28.5 - 36.3)
**Spain**	66.1(58.6 - 73.5)	94.9(80.2 - 112.2)	29.18 (24.91-34.17)	48.64 (39.47-59.88)	13(12.6 - 13.4)	11.2(11.1 - 11.4)	2.8(2.7 - 2.8)	15.9(14.1 - 17.9)
**Sri Lanka**	76.9(65.7 - 89.7)	183.4(141.4 - 234.6)	6.05 (5.34-6.84)	38.85 (31.02-48.71)	9.9(9.2 - 10.7)	11.7(11.2 - 12.2)	2.3(2.3 - 2.4)	11.1(9.9 - 12.4)
**Sudan**	81.7(68.3 - 97.7)	134.9(109.9 - 165.7)	5.94 (4.85-7.03)	26.18 (18.58-36.75)	10.8(10.5 - 11.1)	13.2(12.9 - 13.5)	2.6(2.5 - 2.6)	48.3(43.1 - 54)
**Suriname**	104.7(90.1 - 121.7)	182.8(151.8 - 220.7)	38.42 (32.81-44.2)	70.57 (55.22-90.43)	7.3(6.7 - 8)	12(11.5 - 12.6)	2.7(2.7 - 2.7)	12.0(10.8 - 13.2)
**Swaziland**	90.3(74.8 - 108.5)	168.8(136.8 - 207.3)	2.18 (1.77-2.6)	31.36 (22.22-43.78)	7.8(7.6 - 8.1)	10.7(10.5 - 10.9)	2.5(2.5 - 2.5)	44.8(40 - 50.4)
**Sweden**	121(107.5 - 134.7)	144(121.4 - 171.6)	29.54 (25.08-34.79)	62.91 (50.86-77.96)	14.6(14.2 - 15)	27.6(27.2 - 28)	2.5(2.5 - 2.5)	20.0(17.6 - 22.7)
**Switzerland**	143.4(127.5 - 159.2)	107.5(90.9 - 127.4)	18.7 (15.87-21.89)	42.45 (34.3-52.38)	13.8(13.5 - 14.2)	13.5(13.3 - 13.7)	2.6(2.6 - 2.7)	14.8(13.1 - 16.8)
**Syria**	110(89.7 - 134.9)	144.3(109.3 - 189.3)	12.49 (10.13-14.85)	34.32 (26.07-44.89)	13.6(13.3 - 13.9)	9.1(8.6 - 9.7)	2.8(2.7 - 2.8)	33.6(28.9 - 38.9)
**Taiwan**	146.8(129.2 - 165)	265.0(228.8 - 305.9)	17.15 (12.85-22.58)	61.3 (49.22-73.43)	10.6(9.7 - 11.5)	8.6(7.6 - 9.6)	2.8(2.8 - 2.8)	21.2(17.8 - 25.2)
**Tajikistan**	92.7(80.9 - 106.6)	62(49.1 - 77.5)	76.14 (66.16-87.27)	45.45 (35.82-57.6)	8.4(8 - 8.7)	20.5(19.6 - 21.4)	2.6(2.5 - 2.6)	7.3(6.6 - 8.1)
**Tanzania**	65.6(54.7 - 78.7)	178.2(144.3 - 220.5)	2.72 (2.22-3.23)	46.33 (32.98-64.9)	11(10.7 - 11.3)	9.7(9.5 - 9.9)	2.6(2.6 - 2.6)	15.6(13.8 - 17.6)
**Thailand**	102.7(90 - 115.5)	167.7(144.9 - 193.9)	9.4 (7.12-12.4)	31.99 (26.09-38.02)	10.4(9.5 - 11.3)	10.7(9.5 - 11.9)	2.5(2.5 - 2.5)	7.4(6.3 - 8.8)
**The Bahamas**	83.3(71.4 - 97.2)	104.3(86.4 - 126.4)	15.59 (12.92-18.28)	47.93 (37.49-61.47)	11.5(10.6 - 12.4)	7.4(6.9 - 7.8)	2.6(2.6 - 2.6)	20.3(18.3 - 22.4)
**The Gambia**	31.0(25.7 - 37)	50.9(41.4 - 62.9)	26.17 (21.52-30.74)	11.48 (8.19-16.06)	9.8(9.5 - 10.1)	10(9.8 - 10.3)	2.7(2.7 - 2.7)	20.1(17.8 - 22.6)
**Timor-Leste**	56.5(49.7 - 63.5)	147.2(127.2 - 170.3)	18.34 (13.91-23.89)	16 (12.94-19.03)	12.3(11.3 - 13.3)	7.4(6.6 - 8.3)	2.6(2.6 - 2.6)	9.0(7.6 - 10.6)
**Togo**	54.6(45.5 - 65.3)	154.2(125.1 - 189.4)	16.81 (13.78-19.78)	21.56 (15.26-30.42)	12.1(11.8 - 12.5)	10.7(10.5 - 10.9)	2.6(2.6 - 2.6)	13.9(12.3 - 15.7)
**Tonga**	140.5(123.7 - 157)	143.8(124.5 - 166.6)	8.84 (6.64-11.7)	27.43 (22.32-32.49)	13.3(12.2 - 14.3)	8.0(7.1 - 8.9)	2.6(2.5 - 2.6)	9.3(7.8 - 11.1)
**Trinidad and Tobago**	68.4(58.6 - 79.5)	85.3(70.8 - 102.9)	33.5 (28.18-38.76)	64.54 (49.89-83.15)	9.6(8.9 - 10.4)	8.5(8 - 8.9)	2.6(2.6 - 2.6)	7.9(7.2 - 8.7)
**Tunisia**	66.4(53.9 - 81.3)	206.9(158 - 270.7)	0.75 (0.6-0.89)	36.93 (28.15-48.07)	10(9.7 - 10.2)	13.3(12.5 - 14)	3.0(3.0- 3.1)	39(33.6 - 45.3)
**Turkey**	83.2(67.7 - 102.1)	119.1(90.6 - 156.1)	3.82 (3.07-4.54)	47.97 (36.82-62.9)	11(10.7 - 11.2)	11.6(10.9 - 12.3)	2.8(2.8 - 2.9)	22.2(19.1 - 25.6)
**Turkmenistan**	67.7(59.1 - 77.7)	113.5(89.7 - 143.3)	64.43 (55.46-74.9)	74.34 (58.18-94.66)	11.9(11.4 - 12.3)	13.6(12.9 - 14.4)	2.5(2.5 - 2.6)	13(11.7 - 14.4)
**Uganda**	55.9(46.5 - 66.6)	117.6(95.7 - 144.3)	3.57 (2.92-4.23)	18.83 (13.38-26.35)	12.0(11.6 - 12.3)	10.6(10.4 - 10.9)	2.6(2.6 - 2.6)	16.5(14.7 - 18.5)
**Ukraine**	87.0(75.4 - 100.1)	165.4(129.8 - 207.9)	45.36 (38.64-52.94)	33.91 (27.83-40.35)	10.7(10.3 - 11.2)	14.7(13.9 - 15.5)	2.6(2.5 - 2.6)	20.1(18.1 - 22.2)
**United Arab Emirates**	110.6(90.8 - 135.7)	124.7(95.1 - 161.8)	27.97 (23.04-32.87)	27.29 (20.86-35.74)	8.4(8.2 - 8.7)	9.3(8.6 - 10)	2.4(2.4 - 2.5)	13.0(11.2 - 15.2)
**United Kingdom**	96.4(85.5 - 107.3)	112.1(94.5 - 132.3)	30.55 (25.74-36.13)	37.59 (30.3-46.66)	12.5(12.1 - 12.9)	14.1(13.9 - 14.3)	2.6(2.6 - 2.6)	11.0(9.7 - 12.4)
**United States**	92.8(82.6 - 102.8)	124.5(105.4 - 146.6)	21.78 (18.54-25.62)	31.14 (25.32-38.54)	11.8(11.4 - 12.2)	11.9(11.7 - 12)	2.8(2.8 - 2.8)	13.4(11.9 - 15.2)

**Uruguay**	96.1(82.4 - 111.3)	159.9(132.3 - 193.5)	43.58 (36.91-50)	70.73 (54.95-90.9)	11.5(10.7 - 12.3)	11.6(11.1 - 12.2)	2.6(2.5 - 2.6)	33.4(30.1 - 37)
**Uzbekistan**	81.7(71.3 - 93.9)	224.4(178.5 - 281)	44.01 (37.35-51.64)	89.27 (69.81-113.31)	10.4(10 - 10.8)	18.1(17.3 - 19)	2.5(2.5 - 2.6)	19.8(17.9 - 21.9)
**Vanuatu**	44.9(39.6 - 50.1)	74.9(64.6 - 86.9)	5.04 (3.76-6.68)	37.6 (30.52-44.88)	12.4(11.3 - 13.4)	7.6(6.7 - 8.5)	2.6(2.6 - 2.6)	17.7(14.9 - 20.9)
**Venezuela (Bolivarian Republic of)**	109.3(93.5 - 127.5)	91.9(75.5 - 111.3)	28.41 (24.01-33)	43.8 (33.98-56.41)	5.2(4.7 - 5.7)	5.9(5.5 - 6.3)	2.4(2.4 - 2.5)	6.8(6.2 - 7.5)
**Vietnam**	90.1(78.7 - 101.1)	174.6(150.8 - 201.7)	7.48 (5.61-10.01)	43.37 (35.37-51.46)	5.2(4.6 - 5.8)	3.8(3.2 - 4.4)	2.2(2.2 - 2.2)	6.5(5.5 - 7.8)
**Yemen**	44.5(36.5 - 54.9)	69.6(53 - 91.8)	14.24 (11.6-16.78)	20.23 (15.43-26.35)	8.2(8.0 - 8.4)	13.0(12.3 - 13.6)	2.6(2.6 - 2.7)	18.7(16.1 - 21.6)
**Zambia**	51.3(42.9 - 61.6)	206.1(167.5 - 253.3)	9.28 (7.63-10.98)	69.73 (50.03-96.75)	12.2(11.8 - 12.5)	10.7(10.4 - 10.9)	2.6(2.6 - 2.6)	20.6(18.3 - 23.3)
**Zimbabwe**	83.0(69.7 - 99.4)	146.1(120.2 - 179)	5.81 (4.73-6.88)	44.7 (32-62.21)	2.7(2.7 - 2.7)	18.0(16.1 - 20.3)	2.7(2.7 - 2.7)	18.0(16.1 - 20.3)

Supplementary Table 2: The average intake of eight dietary factors in 185 countries/regions in 1990, including fruits, non-starchy vegetables, processed meats, unprocessed red meats, dietary fiber (in grams/day), saturated fats, monounsaturated fatty acids, and total omega-6 fatty acids (in kcal%/day), classified by age, gender, education level, and residential area.

		Mean(95%UI)							
**Population subgroup**	**Dietary factors**	**Worldwide**	**East & Southeast Asia**	**Central and eastern Europe and central Asia**	**High-Income Countries**	**Latin America & Caribbean**	**Middle East & North Africa**	**South Asia**	**Sub-Saharan Africa**
**Overall**	**Fruits**	86.0(80.4-92.0)	83.8(72.3-95.4)	75.5(64.1-88.5)	109.4(94.6-124.2)	97.1(87.0-107.2)	108.8(81.9-144.0)	47.4(23.4-71.5)	71.1(62.9-80.2)
	**Non-starchy vegetables**	123.0(114.8-132)	148.2(124.9-174.9)	123(100.8-149.5)	106.1(95.1-116.8)	90(74.7-105.1)	134.8(115.2-157.6)	158.8(91.1-225)	130.5(113-150.7)
	**Total processed meats**	23.1(20.3-26.3)	16.6(12.5-21.1)	44.2(37.3-52.4)	26.4(20.7-32.1)	28.9(23.1-35.8)	24.4(15.7-36.9)	2.9(0.8-5.2)	11(8.8-13.9)
	**Unprocessed red meats**	48.5(43.1-54.3)	38.7(31.3-47.1)	88.9(69.2-113.5)	64.3(57.6-71.7)	56.7(45-71.1)	47.1(34.7-63.4)	21.5(2.4-39.7)	20.7(16.9-25.4)
	**Saturated fat**	11.4(10.9-11.9)	13.9(12.1-15.9)	12.9(12.5-13.3)	14.1(13.2-15)	9.2(8.5-10)	9.8(8.9-10.7）	6.4(3.9-8.7)	10.9(10.3-11.5)
	**Monounsaturated fatty acids**	12.4(11.8-13)	9.2(8.5-10)	16.2(14.3-18.4)	13.5(12.6-14.4)	10.7(9.7-11.9)	12.9(10.8-14.9)	6.1(3.6-8.6)	13.1(12-14.2)
	**Total omega-6 fat**	2.6(2.6-2.6)	2.6(2.5-2.6）	2.6(2.5-2.6)	2.6(2.6-2.7)	2.6(2.6-2.7)	2.7(2.6-2.8)	2.5(2.4-2.7)	2.6(2.6-2.6)
	**Dietary fiber**	18.3(16.6-20.)	7.4(6-9.1）	16.9(13.5-21.3)	18.7(16.1-21.8)	12.4(10.3-14.8)	25.8(19.1-32.6)	20.1(14.5-30.1)	24.3(21.1-28)
**Sex**									
**Female**	**Fruits**	91.7(85.5-98.3)	93.3(80.8-106)	80.8(68.7-95.1)	121.8(105.3-138.1)	101.2(90.6-112)	114.2(86.1-151.5)	48.0(22.2-73.1)	74.0(65.1-84.3)
	**Non-starchy vegetables**	128.2(119.6-137.5)	156.8(131.7-186.6)	127.3(105-154.7)	115.4(104-126.9)	91.9(76-107.9)	140.3(120.7-162.6)	159.8(91.5-228.4)	135(117.2-155)
	**Total processed meats**	21.3(18.8-24.2)	16.1(12.1-21.5)	39.1(32.7-46.8)	23.1(18-28.3)	27.9(22.4-34.9)	22.4(14.5-34.5)	2.7(0.6-4.8)	10.5(8.4-13.2)
	**Unprocessed red meats**	47.2(42.1-53)	38.2(30.6-47.5)	87.5(68.3-111.6)	58.1(51.9-65)	56.1(44.7-70.4)	45.7(33.9-61.1)	21.9(4-39.7)	20.8(17.1-25.2)
	**Saturated fat**	11.6(11.2-12.2)	14.3(12.6-16.3)	12.9(12.5-13.3)	14.4(13.5-15.5)	9.4(8.7-10.2)	10.2(9.2-11.1)	6.5(4.1-8.9)	11.1(10.4-11.8)

	**Monounsaturated fatty acids**	12.2(11.6-12.8)	9.4(8.6-10.2)	15.7(13.9-17.7)	13.5(12.7-14.3)	10.7(9.7-11.9)	12.1(10.4-13.9)	6(3.5-8.6)	12.8(11.7-14)
	**Total omega-6 fat**	2.6(2.6-2.6)	2.6(2.5-2.6)	2.6(2.5-2.6)	2.6(2.6-2.7)	2.6(2.6-2.7)	2.7(2.6-2.8)	2.5(2.3-2.7)	2.6(2.6-2.6)
	**Dietary fiber**	18.2.64-20)	7.5(6.1-9.1)	16.8(13.3-21.1)	19(16.4-22.2)	12.5(10.5-15)	25.2(18.3-31.9)	20.8(14.5-29.7)	24.6(21.3-28.4)
**Male**	**Fruits**	80.1(75-85.7)	75.4(65.6-85.4)	64.5(55.1-75.2)	96.4(85.6-108.1)	92.9(83.3-102.3)	103.6(77-136.8)	46.6(22-70.6)	71.1(62.8-80.7)
	**Non-starchy vegetables**	117.7(109.8-126.3)	140.8(117.6-168.4)	110.8(90.6-135.4)	96.1(84.9-106.9)	87.6(73-102.3)	129.5(110-151.8)	158.9(91.9-226.4)	129(112.1-147.9)
	**Total processed meats**	24.7(21.7-28)	16(12.2-20.7)	48.3(40.2-58.1)	29.8(23-36.5)	29.7(23.8-36.9)	25.9(17.1-38.6)	3.1(0.7-5.5)	12.1(9.6-15.2)
	**Unprocessed red meats**	49.7(44.3-55.5)	37.6(30.1-46.9)	92.2(72.2-118)	70.6(63-79.2)	56.8(45.8-70.2)	48.1(35.9-64.3)	21(1.5-40)	20.1(16.5-24.5)
	**Saturated fat**	11.2(10.7-11.7)	13.9(12.2-15.8)	12.5(12.1-12.9)	13.7(12.9-14.6)	9(8.3-9.7)	9.5(8.6-10.4)	6.3(3.9-8.7)	10.7(10-11.4)
	**Monounsaturated fatty acids**	12.6(11.9-13.2)	9.3(8.5-10.1)	16.6(14.7-18.9)	13.4(12.4-14.5)	10.7(9.6-11.9)	13.5(11.2-15.7)	6.2(3.7-8.7)	13.2(12.1-14.4)
	**Total omega-6 fat**	2.6(2.6-2.6)	2.6(2.5-2.6)	2.6(2.5-2.6)	2.6(2.6-2.7)	2.6(2.5-2.6)	2.7(2.6-2.8)	2.5(2.4-2.6)	2.6(2.6-2.6)
	**Dietary fiber**	18.1(16.5-19.8)	7.3(5.9-9)	17(13.6-21.2)	18.4(15.7-21.4)	12.2(10.2-14.6)	26.4(19.1-33.7)	21.1(14.4-30.5)	24(20.8-27.8)
**Area of residence**									
**Rural**	**Fruits**	83.4(77.5-89.5)	77.3(67.2，87.6)	76.5(64.7-90.6)	124.1(107.3-141)	91.5(81.6-101.1)	95.6(70.9，128.3)	43.9(21.6，66.1)	66.2(58.6，74.9)
	**Non-starchy vegetables**	122.9(114.5-131.9)	148.8(122.9-179.4）	124.8(102.9-152.1）	106.1(95.3-116.9）	89.5(74.5-104.4）	128.8(110.1-150.2）	159.6(93.9-229.3）	131.4(114.7-150.6）
	**Total processed meats**	20.6(18.1-23.6)	15.8(11.9-20.7)	44.6(37.4-53.1)	29.2(22.8-35.5)	22(17.4-27.7)	11.4(7.2-17.9)	3.6(1-6.1)	10.2(8.1-12.7)
	**Unprocessed red meats**	45.2(40.1-50.9)	37.9(28.8-47)	83.1(64.8-105.8)	63.8(57.1-71)	51.1(40.8-64.1)	43.9(32.4-58.9)	18.2(1.1-34.2)	17.6(14.3-21.6)
	**Saturated fat**	45.2(40.2-50.8)	37.9(28.6-47.1)	83.2(64.9-106.9)	63.8(57.2-71.3)	51.2(40.9-64.3)	44(32.5-59)	18.5(1.7-35.5)	17.6(14.4-21.5)
	**Monounsaturated fatty acids**	12.2(11.6-12.9)	9.2(8.5-9.9)	16(14.2-18.1)	13.3(12.5-14.2)	10.4(9.3-11.6)	12.7(10.8-14.7)	6.1(3.6-8.5)	12.9(11.8-14.1)
	**Total omega-6 fat**	2.6(2.6-2.6)	2.6(2.5-2.6)	2.5(2.5-2.6)	2.6(2.6-2.7)	2.6(2.5-2.6)	2.7(2.6-2.8)	2.5(2.4-2.6)	2.6(2.5-2.6)

	**Dietary fiber**	18.1(16.5-19.8)	6.9(5.6-8.4)	17.4(13.8-21.8)	19(16.3-22.2)	13.9(11.6-16.6)	21.6(15.8-27.6)	20.5(14.2-29.3)	24.4(21.1-28.1)
**Urban**									
	**Fruits**	91.2(85.5-97.4)	91.7(79.3，103.5)	74.8(63.4-88.5)	104.4(91.0-118.4)	102.1(91.1-112.9)	114.3(86.3-149.6)	60.9(29.8，92.9)	82.8(73.2-93.7)
	**Non-starchy vegetables**	122.6(114.3-131.4)	147.8(123.9-176.5)	121.7(98.6-149.1)	106(95.2-116.9)	90.2(74.9-105.3)	137.3(117-160)	164.5(98.7-234)	127.5(110.2-146.9)
	**Total processed meats**	24.6(21.6-28)	16.8(12.9-21.7)	44(37-52)	25.6(20-31.1)	33.8(27.3-41.8)	28.9(19.4-42.7)	0.8(0.2-1.4)	12.3(9.8-15.4)
	**Unprocessed red meats**	52.1(46.8-58.1)	40.8(31.2-50.2)	92.6(72.6-117.8)	64.5(57.8-71.8)	60.3(48.5-75.1)	48.1(35.8-64.3)	34(5.3-63.2)	26.7(21.9-32.4)
	**Saturated fat**	52.1(46.7-58.2)	40.7(31.2-50.2)	92.5(72.9-116.9)	64.5(57.6-72.1)	60.1(48.5-74.4)	48(35.7-64)	33.9(5.3-62.7)	26.6(21.7-32.5)
	**Monounsaturated fatty acids**	12.6(11.9-13.2)	9.4(8.7-10.2)	16.3(14.4-18.5)	13.5(12.7-14.4)	10.9(9.8-12.2)	12.9(11-14.9)	6.3(3.8-8.8)	13.3(12.2-14.4)
	**Total omega-6 fat**	2.6(2.6-2.7)	2.6(2.5-2.6)	2.6(2.5-2.6)	2.6(2.6-2.7)	2.6(2.6-2.7)	2.7(2.6-2.7)	2.6(2.4-2.7)	2.8(2.7-2.8)
	**Dietary fiber**	18.2(16.6-20)	7.4(6-9.2)	16.6(13.1-20.7)	18.6(16-21.8)	11.1(9.3-13.3)	28(20.9-35.1)	22.5(15.7-31.8)	24.2(21-27.8)
**Education level**									
**0-6 years(low)**	**Fruits**	79.1(73.3-85.2)	70.3(60.6-79.9)	76.9(62.6-94.4)	114.9(98.7-130.9)	85.5(76-95)	80.0(60.2-100.2)	33.8(12.2-55.2)	62.8(55.3-70.4)
	**Non-starchy vegetables**	116.8(107.9-126.4)	134(106.8-161.3)	133.7(103.7-170.4)	100(89.4-110.2)	73.4(60.5-86)	118.2(99.1-137.5)	138.6(74.1-203)	138.3(118.8-159.8)
	**Total processed meats**	23.9(20.5-27.8)	13(9-18.6)	50.7(42.2-61.1)	23.6(18.6-28.9)	28.9(22.6-37)	24.9(14.9-40.5)	2.9(0.6-5.3)	12.1(9-16.2)
	**Unprocessed red meats**	50.6(44.1-57.9)	35.7(24.5-46.8)	105.3(76.7-134)	67.1(60.4-74.9)	52.6(40.3-68.1)	46.3(33.3-64.3)	17.8(0.2-34.7)	18.4(14.5-23.5)
	**Saturated fat**	10.9(10.4-11.4)	12.4(10.1-14.8)	12.7(12.1-13.3)	13.6(12.7-14.5)	8.4(7.7-9.2)	9.3(8.3-10.4)	6.1(3.2-9)	10.6(9.8-11.5)
	**Monounsaturated fatty acids**	12.2(11.5-12.9)	8.8(7.8-9.8)	16(14.1-18.1)	13(12.2-13.9)	10.9(9.7-12.2)	12.6(10.7-14.6)	6(3.1-8.9)	13.1(11.8-14.5)
	**Total omega-6 fat**	2.6(2.6-2.6)	2.6(2.5-2.6)	2.6(2.5-2.6)	2.6(2.6-2.6)	2.6(2.5-2.6)	2.7(2.6-2.8)	2.5(2.3-2.7)	2.6(2.6-2.6)
	**Dietary fiber**	18.5(16.6-20.6)	7.6(5.8-10)	19.6(15.4-25)	19.3(16.6-22.4)	11.6(9.3-14.3)	24.7(17.6-32.1)	20.4(13.3-30.3)	24.5(20.4-29.3)
**6-12years(medium)**	**Fruits**	89.5(83.6-95.8)	91.0(77.9-104.4)	80.7(65.5-98.7)	103.5(89.2-117.6)	101.1(90-112.1)	93(69.1-117.5)	49.7(18-80.7)	81.5(72-91.5)

	**Non-starchy vegetables**	123.5(114.2-133.6)	142.3(112.8-171.9)	136.6(106.2-174.7)	101.3(91.1-111.6)	81.4(67.7-95.4)	125.1(105.3-144.7)	148.0(82.2-218.9)	148.5(128.1-171.9)
	**Total processed meats**	25.4(22-29.4)	15.9(11.1-22.4)	47.5(39.4-57.2)	27.7(21.6-33.8)	30.9(24.1-39.5)	27.6(16.9-43.9)	3.2(0.6-5.8)	13.7(10.2-18.3)
	**Unprocessed red meats**	54.9(48.4-62.2)	39.2(26.8-51.9)	102.2(74.4-129.7)	66.7(59.6-74.5)	62.3(48.7-80.7)	50.6(36.4-69.5)	23.1(0.3-46.2)	26.5(20.6-33.9)
	**Saturated fat**	11.2(10.7-11.8)	12.7(10.2-15.2)	13.1(12.4-13.7)	14.2(13.3-15.1)	9(8.2-9.8)	9.6(8.6-10.7)	6.8(3.8-9.7)	10.7(9.9-11.6)
	**Monounsaturated fatty acids**	12.3(11.6-13)	9.4(8.4-10.5)	15.5(13.7-17.6)	13.5(12.6-14.4)	11.3(10.1-12.7)	12.6(10.6-14.5)	6.4(3.5-9.4)	12.6(11.4-14)
	**Total omega-6 fat**	2.6(2.6-2.6)	2.6(2.5-2.6)	2.6(2.5-2.6)	2.6(2.6-2.7)	2.6(2.6-2.7)	2.6(2.6-2.7)	2.5(2.3-2.7)	2.6(2.6-2.7)
	**Dietary fiber**	17.9(16.1-19.8)	7.6(5.8-9.9)	18.5(14.3-23.8)	18(15.4-20.9)	11.5(9.3-14.1)	24.2(16.9-31.7)	20.5(13.2-31)	24(20-28.6)
**＞12years (high)**	**Fruits**	104.6(97.9-111.3)	110.8(95.5-126.9)	77.4(62.9-95)	113.4(98.4-128.9)	118.5(106-131.5)	103.0(77.0-130.2)	64.7(24-106.8)	111.4(97.8-125.1)
	**Non-starchy vegetables**	136.8(126.5-147.8)	148(117.5-178.2)	129.7(101.3-166.6)	113(101.3-125)	100.5(83.7-117)	137.5(116.2-158.7)	164(83.8-242.9)	174.6(151.4-202)
	**Total processed meats**	25.4(22-29.4)	18.8(11.4-26.1)	41.7(34.1-50.9)	25.9(20.3-31.6)	34.9(27.6-44.1)	28(17.4-44.6)	3.3(0.5-6.1)	13.8(10.3-18.2)
	**Unprocessed red meats**	59.9(53.1-67.5)	39.6(26.9-52.1)	105.7(77.2-134.9)	61(54.6-68.3)	70.3(54.7-89.9)	57.1(41.5-78.5)	28.3(0-56.3)	39.5(31.1-50.4)
	**Saturated fat**	11.5(10.9-12)	13.4(10.9-15.9)	13.1(12.4-13.7)	14.2(13.2-15.1)	9.5(8.7-10.4)	10(8.9-11.1)	7.1(4-10.2)	10.7(9.8-11.6)
	**Monounsaturated fatty acids**	12.4(11.7-13)	10.1(9-11.1)	14.7(12.9-16.6)	13.6(12.8-14.5)	12.1(10.7-13.5)	13.4(11.4-15.5)	6.3(3.2-9.4)	12.1(10.9-13.5)
	**Total omega-6 fat**	2.6(2.6-2.6)	2.6(2.5-2.7)	2.6(2.5-2.6)	2.6(2.6-2.7)	2.7(2.6-2.7)	2.7(2.6-2.8)	2.5(2.3-2.7)	2.6(2.6-2.7)
	**Dietary fiber**	18.6(16.8-20.7)	8.1(6.1-10.7)	17.3(13.5-22.2)	19.3(16.5-22.5)	12.4(10-15.3)	25.6(17.8-33.5)	21.8(14.4-32.4)	24.9(20.8-29.6)

In previous global dietary database reports, Central Europe, Eastern Europe, and Central Asia were referred to as the former Soviet Union, while Southeast Asia and East Asia were collectively called Asia. UI, Uncertainty Interval.

Supplementary Table 3: National mean (95% UI) intakes of eight nutrients in 1990 across 185 countries and regions.

Country/Region	Fruits	Non-starchy vegetables	Total processed meats	Unprocessed red meats	Saturated fat	Monounsaturated fatty acids	Total omega-6 fat	Dietary fiber
**Afghanistan**	77.8(66.4 - 90.8)	163.6(127.6 - 208.7)	2.5(2.2 - 2.8)	35.1(28 - 43.7)	7.8(7.2 - 8.5)	5.0(4.7 - 5.3)	2.6(2.6 - 2.6)	60.1(54.6 - 66.3)
**Albania**	18.0(15.7 - 20.6)	73.5(58.2 - 92.3)	23.4(19.6 - 27.9)	6.6(5.3 - 7.8)	9.4(9 - 9.8)	12.1(11.6 - 12.7)	2.5(2.4 - 2.5)	6.3(5.7 – 7.0)
**Algeria**	71.9(58.8 - 88.6)	140.4(106.6 - 183.6)	8.6(6.9 - 10.2)	23.4(17.9 - 30.5)	12.9(12.6 - 13.2)	19.7(18.7 - 20.8)	2.6(2.6 - 2.6)	16.3(14.0 - 18.8)
**Angola**	45.3(37.7 - 54.2)	144(116.6 - 177)	3.2(2.6 - 3.7)	25.6(18.1 - 36.1)	12.5(12.2 - 12.8)	10.6(10.3 - 10.8)	2.6(2.6 - 2.6)	17.4(15.4 - 19.5)
**Antigua and Barbuda**	89.0(76.6 - 103.3)	201.5(167 - 242.7)	30.9(26.2 - 35.5)	35.3(27.5 - 45.3)	11.1(10.4 - 12)	8.7(8.2 - 9.2)	2.6(2.6 - 2.7)	19.1(17.3 - 21.2)
**Argentina**	92.4(79.4 - 108)	128.1(105.8 - 154.5)	11.9(9.9 - 13.9)	80.1(62.5 - 102.1)	10.8(10 - 11.6)	11(10.5 - 11.6)	2.6(2.6 - 2.6)	8.3(7.5 - 9.1)
**Armenia**	37.1(32.2 - 42.6)	92.5(73.5 - 116.1)	22.7(19 - 27.1)	22.1(17.4 - 27.8)	12.8(12.3 - 13.3)	20.6(19.5 - 21.7)	2.5(2.5 - 2.6)	6.8(6.2 - 7.5)
**Australia**	152.5(135.8 - 169.7)	111.9(94 - 132.9)	10.9(9.2 - 12.8)	77.4(62.4 - 96.1)	13.3(12.9 - 13.6)	11.2(11 - 11.3)	2.6(2.6 - 2.6)	17.5(15.5 - 19.7)
**Austria**	108.5(95.9 - 121.2)	75(63.1 - 88.8)	47.1(39.6 - 55.8)	114.3(92.4 - 141.8)	17(16.6 - 17.4)	13.2(13 - 13.3)	2.8(2.8 - 2.8)	19.1(16.9 - 21.6)
**Azerbaijan**	103.8(90.4 - 119.1)	58.5(46.2 - 73.8)	77.7(68 - 88.8)	34.2(27.8 - 40.5)	12.7(12.3 - 13.2)	31.6(30.6 - 32.6)	2.5(2.5 - 2.6)	6.2(5.6 - 6.9)
**Bahrain**	107.5(87.6 - 131.8)	189.3(144.2 - 246.8)	14.0(11.4 - 16.6)	36.2(27.6 - 47.5)	9.7(9.5 - 10)	12.2(11.5 - 12.8)	2.7(2.7 - 2.7)	7.5(6.5 - 8.7)
**Bangladesh**	49.1(42.1 - 57.4)	215.3(168 - 275.1)	0.5(0.4 - 0.5)	6.2(4.9 - 7.8)	2.3(2 - 2.6)	1.7(1.6 - 1.8)	2.4(2.3 - 2.4)	17.5(15.6 - 19.5)
**Barbados**	126.4(108.5 - 147.2)	140.4(116.8 - 168.7)	11.5(9.5 - 13.4)	222.9(176.8 - 279)	10(9.2 - 10.8)	8.4(7.9 - 8.9)	2.8(2.8 - 2.8)	37.2(33.6 - 40.9)
**Belarus**	87.4(76.2 - 100.3)	80.5(64 - 101.1)	25.7(21.7 - 30.6)	68.5(56.3 - 80.9)	13(12.6 - 13.5)	11.5(10.9 - 12.1)	2.5(2.5 - 2.6)	6.1(5.6 - 6.8)
**Belgium**	111.6(98.5 - 124.3)	80.8(68.3 - 95.2)	26.6(22.7 - 31.3)	60.8(48.6 - 75.2)	16.1(15.7 - 16.5)	13.9(13.7 - 14.1)	2.7(2.7 - 2.7)	18.8(16.6 - 21.4)
**Belize**	112.2(96.5 - 130)	85.9(71.6 - 103.2)	30.1(25.2 - 35)	69.5(54.5 - 88.7)	9.7(9 - 10.5)	10.7(10.1 - 11.3)	2.5(2.4 - 2.5)	8.8(8 - 9.7)
**Benin**	59.1(49.4 - 70.5)	98.8(80.3 - 121.3)	17.4(14.3 - 20.4)	46.9(33.2 - 66.5)	12.5(12.1 - 12.8)	16.7(16.4 - 17.1)	2.6(2.6 - 2.6)	7.9(7 – 9.0)
**Bhutan**	80.9(69 - 94.9)	273.3(212.6 - 347.9)	1.8(1.6 - 2.1)	20.2(16.3 - 25.1)	6.2(5.7 - 6.8)	5.9(5.6 - 6.2)	2.6(2.5 - 2.6)	23.2(20.8 - 25.9)
**Bolivia**	86.8(74.9 - 100.6)	113.5(94.1 - 136.6)	6.8(5.6 - 8)	74.5(58.3 - 94.4)	11.1(10.3 - 12)	11.1(10.5 - 11.6)	2.5(2.5 - 2.6)	6(5.4 - 6.6)
**Bosnia and Herzegovina**	41(35.8 - 47)	120.5(95.2 - 152.2)	7.8(6.5 - 9.4)	28.1(22.3 - 35.5)	11.9(11.4 - 12.3)	23.9(22.6 - 25)	2.5(2.5 - 2.6)	4.3(3.9 - 4.8)
**Botswana**	45.8(38.2 - 54.7)	115(93.5 - 141.5)	15.6(12.7 - 18.5)	50.9(36.2 - 71.6)	10.4(10.1 - 10.7)	11.4(11.2 - 11.6)	2.6(2.6 - 2.6)	51.5(46.2 - 57.3)
**Brazil**	83.9(72.1 - 97.8)	93.2(76.5 - 112.3)	11.4(9.4 - 13.3)	35.9(28.1 - 45.7)	8.2(7.5 - 8.9)	11.8(11.3 - 12.4)	2.7(2.6 - 2.7)	13.5(12.2 - 14.9)
**Brunei**	90.2(79.4 - 101)	139.7(120.4 - 161.8)	33.1(26 - 41.9)	79.7(65.3 - 94.4)	11.4(10.5 - 12.4)	8.6(7.6 - 9.5)	2.5(2.5 - 2.5)	12.2(10.3 - 14.5)
**Bulgaria**	88.4(77.1 - 101.4)	437.9(362.1 - 528.6)	25.3(21.2 - 30.1)	103(84.5 - 121.6)	12.7(12.2 - 13.2)	9.5(8.9 - 10.1)	3(3 - 3.1)	22.3(20.1 - 24.7)
**Burkina Faso**	34.1(28.5 - 40.7)	69.7(57 - 84.8)	2.1(1.7 - 2.4)	8.9(6.3 - 12.7)	12.2(11.9 - 12.6)	17.6(17.3 - 18)	2.6(2.6 - 2.6)	11.7(10.4 - 13.2)
**Burundi**	78.5(65.6 - 93.7)	199.4(162.6 - 244.1)	2.2(1.8 - 2.6)	17.2(12.2 - 24.2)	11.3(11 - 11.6)	13.6(13.4 - 13.9)	2.5(2.5 - 2.6)	28.5(25.2 - 32.1)

**Cambodia**	53.3(46.8 - 59.8)	120.3(103.6 - 140)	6.7(5 - 8.9)	20.6(16.9 - 24.6)	12.5(11.5 - 13.5)	7.1(6.2 – 8.0)	2.6(2.5 - 2.6)	5.1(4.3 - 6)
**Cameroon**	76.3(63.4 - 91.4)	134(110.2 - 163.8)	5.9(4.9 - 7)	46.1(32.5 - 65.3)	4.9(4.7 - 5.1)	16.5(16.2 - 16.8)	2.5(2.5 - 2.6)	19.6(17.4 - 22.2)
**Canada**	99.3(88.4 - 110.4)	124.5(105.3 - 147.8)	14(11.9 - 16.5)	47.4(38.2 - 59)	11.6(11.3 - 12)	12.1(11.9 - 12.3)	2.5(2.5 - 2.5)	11.8(10.4 - 13.3)
**Cape Verde**	72.4(60.5 - 86.6)	52(42.5 - 63.8)	4.7(3.8 - 5.5)	21.7(15.4 - 30.5)	10.5(10.2 - 10.7)	12.6(12.3 - 12.8)	2.6(2.6 - 2.6)	59.7(53.7 - 66.4)
**Central African Republic**	79.1(65.9 - 94.8)	125.7(102.1 - 154.5)	3.9(3.2 - 4.6)	91.7(65.9 - 126.3)	9.8(9.5 - 10.1)	9.9(9.7 - 10.1)	2.7(2.7 - 2.7)	20.8(18.4 - 23.5)
**Chad**	230.5(191.6 - 275.6)	136.3(111.2 - 166.8)	4.6(3.7 - 5.5)	54.8(38.6 - 77.2)	12.3(12 - 12.6)	10.5(10.3 - 10.7)	2.6(2.6 - 2.6)	15.9(14.1 - 17.8)
**Chile**	107.6(92.2 - 125)	137.3(112.9 - 166.3)	19(15.6 - 22.2)	15.4(12.1 - 19.7)	9.4(8.7 - 10.2)	9.2(8.8 - 9.7)	3.2(3.1 - 3.2)	15.9(14.4 - 17.6)
**China**	87.5(76.7 - 98.8)	26.8(23.1 - 30.9)	3.3(2.5 - 4.4)	21.8(17.7 - 25.9)	7(6.3 - 7.8)	6.8(6.1 - 7.5)	2.8(2.8 - 2.9)	4.4(3.7 - 5.3)
**Colombia**	97.7(83.6 - 113.4)	96.1(79.2 - 116.4)	65.4(54.4 - 76)	106.4(82.8 - 137.2)	8.2(7.5 - 8.9)	13.2(12.7 - 13.8)	2.6(2.5 - 2.6)	18(16.2 - 19.8)
**Comoros**	84.5(70.6 - 100.8)	100.1(81.3 - 123)	2.6(2.1 - 3.1)	35.3(25 - 49.2)	11.5(11.2 - 11.9)	13.8(13.5 - 14.1)	2.6(2.6 - 2.6)	19.2(17.1 - 21.8)
**Congo**	87.8(72.9 - 104.9)	149.2(121.2 - 183.1)	9.9(8 - 11.6)	16(11.4 - 22.4)	8.6(8.4 - 8.9)	13.4(13.2 - 13.7)	2.5(2.5 - 2.5)	24.1(21.4 - 27.2)
**Costa Rica**	114.9(98.6 - 133.2)	79.9(65.6 - 96.8)	32.3(27.4 - 37.3)	65.3(50.9 - 83.7)	10.5(9.7 - 11.3)	9.5(9 - 9.9)	2.5(2.5 - 2.5)	13.5(12.2 - 14.9)
**Cote d'Ivoire**	58.5(48.5 - 70.1)	97(78.5 - 119.4)	4(3.3 - 4.8)	24.6(17.5 - 34.5)	9.3(9 - 9.6)	14(13.7 - 14.3)	2.5(2.5 - 2.5)	22(19.5 - 24.8)
**Croatia**	53.9(47.1 - 61.8)	75.2(59.7 - 94.2)	27.4(23.1 - 32.7)	63.6(51.7 - 75.2)	11.9(11.5 - 12.4)	12.1(11.4 - 12.7)	2.5(2.5 - 2.6)	27.7(25 - 30.6)
**Cuba**	71.5(61.6 - 83.2)	31.3(26 - 37.7)	41.8(35.6 - 47.9)	95.3(74 - 121.7)	9.8(9.1 - 10.6)	21.1(20.2 - 22.1)	2.7(2.6 - 2.7)	2.9(2.6 - 3.2)
**Cyprus**	212.7(188.5 - 236.5)	154.7(130.1 - 183.3)	7.7(6.5 - 9)	71(57.1 - 88)	12.2(11.8 - 12.5)	12.3(12.1 - 12.4)	2.7(2.7 - 2.7)	10.6(9.3 - 12)
**Czech Republic**	68.4(59.5 - 78.3)	95.5(75.7 - 120.7)	35.6(30.1 - 42.2)	92.4(72.8 - 117.6)	14.2(13.8 - 14.7)	10.3(9.7 - 10.9)	2.5(2.5 - 2.6)	25.3(22.9 - 28.1)
**Democratic Republic of the Congo**	93.8(77.7 - 112.6)	230.5(187.1 - 284.7)	10.6(8.6 - 12.6)	8.7(6.1 - 12.3)	17.6(17.2 - 18)	16.4(16.1 - 16.7)	2.6(2.6 - 2.6)	11.5(10.2 - 13)
**Denmark**	98.9(87.7 - 110.1)	71.6(60.4 - 84.5)	29(24.6 - 34.2)	79.7(64.3 - 99)	15.5(15.1 - 15.9)	15.1(14.9 - 15.3)	2.6(2.6 - 2.6)	15.2(13.4 - 17.2)
**Djibouti**	33.9(28.2 - 40.5)	107(87.2 - 130.7)	6(4.9 - 7.1)	24.5(17.5 - 33.8)	12.9(12.5 - 13.2)	14.7(14.4 - 15)	2.7(2.7 - 2.7)	62(56.1 - 68.6)
**Dominica**	95.5(82.1 - 111)	102.9(85.1 - 124.9)	25.2(21.2 - 29.2)	38.8(30 - 49.8)	10.9(10.1 - 11.8)	13(12.4 - 13.7)	2.5(2.5 - 2.5)	12.6(11.4 - 13.9)
**Dominican Republic**	99.1(85.4 - 115)	17.9(14.8 - 21.7)	18(15 - 20.9)	44.9(34.9 - 58)	9.3(8.6 - 10.1)	8.5(8.1 - 8.9)	2.7(2.7 - 2.8)	9.6(8.7 - 10.6)
**Ecuador**	114.3(98.6 - 132.4)	108.9(90.2 - 130.8)	2.8(2.3 - 3.3)	48.4(37.7 - 62)	11.7(10.9 - 12.5)	7.8(7.4 - 8.2)	2.6(2.5 - 2.6)	14.2(12.9 - 15.7)
**Egypt**	96.9(78.7 - 118.8)	168.9(129.7 - 218.9)	3.5(2.8 - 4.1)	15.2(11.6 - 19.8)	7.1(6.9 - 7.3)	17.1(16.3 - 17.9)	2.6(2.6 - 2.6)	21.3(18.3 - 24.6)
**El Salvador**	102.6(88.3 - 119.3)	68.6(56.6 - 83.3)	23.8(19.9 - 27.7)	20.7(16.1 - 26.6)	7.7(7.1 - 8.4)	10.5(10 - 11)	2.6(2.5 - 2.6)	8.7(7.9 - 9.6)
**Equatorial Guinea**	73(60.9 - 87.3)	151.4(123 - 185.9)	4.2(3.4 - 4.9)	30.6(21.8 - 42.8)	10.9(10.6 - 11.2)	11.7(11.5 - 12)	2.6(2.6 - 2.6)	21.9(19.4 - 24.7)
**Eritrea**	55.4(46 - 66.6)	71.9(58.9 - 87.5)	2.1(1.7 - 2.5)	29(20.6 - 40.4)	11.5(11.2 - 11.8)	13.7(13.4 - 14)	2.6(2.6 - 2.6)	24.9(22.1 - 28.1)
**Estonia**	95.2(82.9 - 109.6)	111.3(88.4 - 139.7)	87.8(74 - 103.9)	97.9(80.3 - 115.8)	13(12.6 - 13.5)	12.4(11.7 - 13.1)	2.6(2.5 - 2.6)	14.8(13.4 - 16.4)
**Ethiopia**	36.8(30.6 - 44)	93(75.6 - 113.9)	4.8(3.9 - 5.7)	16.7(11.8 - 23.7)	12.2(11.9 - 12.6)	33.3(32.9 - 33.7)	2.6(2.5 - 2.6)	25.9(22.9 - 29.2)

**Federated States of Micronesia**	109.3(91.9 - 126.6)	180.1(154.5 - 209.8)	10.7(7.9 - 14.2)	17.2(13.5 - 20.9)	13.9(13 - 14.8)	7.8(6.6 - 8.9)	2.6(2.6 - 2.6)	6.9(5.8 - 8.2)
**Fiji**	116(102.3 - 129.6)	159.2(137.2 - 184.1)	15.7(12 - 20.6)	51.1(41.5 - 60.4)	17.4(16.2 - 18.5)	10.9(9.7 - 12.1)	2.6(2.6 - 2.6)	4.8(4 - 5.7)
**Finland**	106.2(93.9 - 118.2)	82.9(69.6 - 98.3)	35.1(29.8 - 41.3)	49.4(39.8 - 61.3)	15.1(14.7 - 15.5)	11.9(11.7 - 12)	2.5(2.5 - 2.5)	18.5(16.4 - 20.9)
**France**	51.6(46.8 - 56.9)	146.9(124.4 - 173.5)	25.5(21.6 - 30.2)	55.1(44.5 - 68.3)	16.9(16.5 - 17.3)	13.7(13.5 - 13.8)	2.7(2.6 - 2.7)	18.9(16.8 - 21.4)
**Gabon**	93.0(77.8 - 111.2)	127.1(103.7 - 154.7)	6.9(5.7 - 8.2)	56.3(39.9 - 79.5)	12.8(12.5 - 13.2)	13.8(13.5 - 14.1)	2.7(2.7 - 2.7)	43.4(38.7 - 48.7)
**Georgia**	69.2(60.3 - 79.1)	80.8(63.5 - 102.2)	34.6(29 - 41)	20.8(17.1 - 24.7)	12.7(12.3 - 13.2)	19.7(18.7 - 20.7)	2.5(2.5 - 2.6)	9.7(8.8 - 10.7)
**Germany**	158.4(140.1 - 176.8)	88.6(74.6 - 105.1)	54.8(46.4 - 64.4)	56.9(46.1 - 69.9)	15.7(15.4 - 16.2)	12.5(12.3 - 12.7)	2.6(2.6 - 2.7)	60.1(52.9 - 68.4)
**Ghana**	80.2(67 - 95.8)	143(116 - 175.7)	1.1(0.9 - 1.3)	39.4(27.8 - 55.3)	9.7(9.4 - 9.9)	8.3(8.1 - 8.5)	2.6(2.6 - 2.6)	8.7(7.7 - 9.8)
**Greece**	146.4(130 - 162.8)	113.6(95.8 - 134.9)	3.6(3.1 - 4.2)	65.3(52.5 - 81.3)	13.9(13.5 - 14.2)	12.3(12.2 - 12.5)	2.7(2.7 - 2.7)	21.7(19.1 - 24.5)
**Grenada**	137.2(117.9 - 159.6)	116.1(96 - 139.6)	6.8(5.7 - 8)	22.9(18 - 29.4)	11.7(10.9 - 12.6)	6.4(6.1 - 6.7)	2.5(2.5 - 2.6)	14.5(13.1 - 16)
**Guatemala**	61.8(53.2 - 71.7)	95.8(79 - 116)	24(20.2 - 28)	19.7(15.4 - 25.4)	3.9(3.5 - 4.4)	6.9(6.5 - 7.3)	2.7(2.7 - 2.7)	17.2(15.5 - 18.9)
**Guinea**	66.5(55.5 - 79.3)	125.4(102.4 - 153.1)	4.2(3.5 - 5)	34.1(24.3 - 47.9)	12.4(12.1 - 12.8)	10.6(10.4 - 10.8)	2.6(2.6 - 2.6)	17.3(15.4 - 19.5)
**Guinea-Bissau**	51.8(43.1 - 62)	21.1(17.1 - 26)	17.3(14.2 - 20.4)	3.5(2.4 - 4.9)	8.6(8.3 - 8.9)	10(9.8 - 10.2)	2.5(2.4 - 2.5)	21.1(18.7 - 23.8)
**Guyana**	105(90.4 - 121.8)	88(72.6 - 106.3)	73.6(66 - 82.4)	43.4(34 - 55.4)	7.8(7.1 - 8.5)	9.9(9.6 - 10.3)	2.3(2.2 - 2.3)	2.9(2.6 - 3.2)
**Haiti**	70.4(60.6 - 81.6)	86.9(72.1 - 104.8)	9.9(8.3 - 11.6)	14.8(11.5 - 19.1)	9.6(8.9 - 10.4)	19.5(18.6 - 20.5)	2.6(2.5 - 2.6)	13.1(11.9 - 14.5)
**Honduras**	87(75 - 100.6)	30.8(25.5 - 37.2)	26.2(22 - 30.3)	13.5(10.5 - 17.4)	8.9(8.2 - 9.7)	9.6(9.1 - 10.1)	2.5(2.5 - 2.6)	11.9(10.8 - 13.2)
**Hungary**	66.4(57.8 - 75.9)	64.7(51.6 - 80.9)	19.7(16.5 - 23.5)	30(23.5 - 38.3)	13.8(13.4 - 14.3)	10.2(9.6 - 10.8)	2.8(2.8 - 2.9)	23.5(21.3 - 25.9)
**Iceland**	81.6(72.5 - 90.9)	69.4(58.6 - 82.5)	29.5(25 - 34.6)	47.9(38.9 - 59.1)	16.8(16.4 - 17.2)	13.7(13.6 - 13.9)	2.4(2.4 - 2.4)	13.6(12 - 15.4)
**India**	32.9(28 - 38.6)	159.5(123.3 - 205.1)	3.3(2.9 - 3.7)	3.2(2.5 - 4)	5.5(5 - 6.1)	6.6(6.2 - 6.9)	2.7(2.7 - 2.7)	25.7(23 - 28.7)
**Indonesia**	68.8(60.3 - 77.2)	162.5(140.8 - 186.8)	12.2(9.1 - 16.2)	20.1(16.3 - 24)	14.9(13.7 - 16)	10.2(9.1 - 11.4)	2.6(2.6 - 2.6)	7(5.8 - 8.3)
**Iran**	169.7(139.2 - 207.5)	176.5(135.1 - 230.9)	3.8(3.1 - 4.6)	29.5(22.4 - 38.3)	8.9(8.6 - 9.1)	10.6(9.9 - 11.2)	2.8(2.8 - 2.9)	7.6(6.6 - 8.9)
**Iraq**	117.5(95.6 - 145)	205.1(156.4 - 269.1)	74.9(66.8 - 82.9)	172.3(134.5 - 222.1)	10.1(9.9 - 10.3)	13.8(13.1 - 14.5)	2.6(2.6 - 2.6)	45.7(39.6 - 52.4)
**Ireland**	84(74.9 - 93.4)	115.2(97.2 - 135.9)	34.2(29 - 40.2)	36.6(29.7 - 45)	15.7(15.3 - 16.1)	13.4(13.2 - 13.6)	2.6(2.6 - 2.6)	15.9(14 - 18.1)
**Israel**	202.9(165.9 - 246.8)	130.7(100.2 - 170)	99.4(86.7 - 113.8)	114.7(87.6 - 150.3)	7.2(7 - 7.4)	18.5(17.5 - 19.4)	2.6(2.6 - 2.6)	16.2(14 - 18.8)
**Italy**	192.9(172.2 - 213.9)	135.4(115 - 159.1)	18.5(15.7 - 21.7)	44.8(36.1 - 55.4)	11.7(11.4 - 12.1)	15(14.9 - 15.2)	2.4(2.4 - 2.5)	11.1(9.8 - 12.6)
**Jamaica**	166.7(143.3 - 193.6)	157.3(130.1 - 190.5)	8.1(6.7 - 9.5)	18.3(14.2 - 23.2)	5.8(5.3 - 6.4)	8.4(8.1 - 8.9)	2.8(2.8 - 2.8)	10.6(9.6 - 11.7)
**Japan**	104.9(91.4 - 119)	264.4(227.7 - 305.1)	14.9(11.2 - 19.5)	35.1(28.5 - 41.8)	7.5(6.7 - 8.2)	10(9 - 11.1)	2.6(2.6 - 2.6)	12.4(10.4 - 14.8)
**Jordan**	180.4(147.8 - 219.9)	131.9(100 - 172.3)	10.5(8.5 - 12.5)	49(37.5 - 64.3)	6.4(6.2 - 6.6)	5(4.6 - 5.3)	2.6(2.5 - 2.6)	46.2(39.8 - 53.6)
**Kazakhstan**	55.5(48.2 - 63.6)	114.6(90.1 - 145.2)	34.8(29.3 - 41.4)	100.2(78.4 - 126.8)	12.9(12.5 - 13.4)	12.4(11.8 - 13)	2.5(2.5 - 2.6)	13.2(11.9 - 14.6)
**Kenya**	100.9(83.8 - 120.9)	59.7(48.7 - 73.1)	0.7(0.6 - 0.9)	22.7(16.2 - 31.9)	12.3(12 - 12.6)	17.9(17.5 - 18.3)	2.6(2.6 - 2.6)	22.7(20.1 - 25.5)

**Kiribati**	72.3(60.5 - 83.7)	109.7(94 - 128.1)	10.5(7.8 - 13.9)	17.4(13.7 - 21.1)	13(12.1 - 13.9)	7.4(6.2 - 8.5)	2.6(2.6 - 2.6)	8(6.7 - 9.6)
**Kuwait**	58.3(47.4 - 71.5)	79.5(60.9 - 103.4)	4(3.2 - 4.7)	10.7(8.1 - 14)	9.8(9.6 - 10.1)	8.5(8 - 8.9)	2.6(2.6 - 2.6)	6(5.1 - 6.9)
**Kyrgyzstan**	74.7(65.2 - 85.6)	42.3(33.5 - 53.4)	35.4(30 - 42.1)	83.6(65.8 - 106.5)	12.9(12.4 - 13.3)	25.9(24.7 - 27.1)	2.5(2.5 - 2.5)	9(8.1 - 9.9)
**Laos**	122.1(107.4 - 137.2)	152.8(131.7 - 177.2)	0.8(0.6 - 1.1)	11.2(9 - 13.4)	12.6(11.6 - 13.6)	7.2(6.4 - 8.1)	2.6(2.5 - 2.6)	8.2(6.9 - 9.7)
**Latvia**	42(36.6 - 48.2)	145.9(116 - 182.9)	44.1(37.1 - 52.6)	205.8(165.1 - 257.1)	13.1(12.6 - 13.5)	10.3(9.7 - 10.8)	2.5(2.5 - 2.6)	8.9(8 - 9.8)
**Lebanon**	472(402.9 - 554.9)	364.2(276.9 - 478.7)	5.9(4.8 - 7.1)	17.2(13.1 - 22.5)	11.1(10.9 - 11.4)	19.6(18.8 - 20.4)	3(3 - 3.1)	53.5(46.3 - 61.8)
**Lesotho**	63.7(53 - 76.2)	446.7(363.8 - 545.9)	3.1(2.6 - 3.7)	23.8(16.9 - 34)	12.3(12 - 12.6)	10.4(10.2 - 10.6)	2.6(2.6 - 2.6)	40.5(36 - 45.6)
**Liberia**	56.7(47.3 - 68)	125.4(101.5 - 154.9)	17.2(14.1 - 20.1)	40.8(29 - 57.4)	12.7(12.3 - 13)	10.7(10.4 - 10.9)	2.6(2.6 - 2.7)	13.4(11.9 - 15.1)
**Libya**	54.9(44.6 - 67.5)	120.7(92.1 - 156.9)	11.5(9.3 - 13.6)	34.5(26.2 - 45.5)	10.2(9.9 - 10.4)	11.2(10.7 - 11.8)	2.5(2.5 - 2.6)	50.4(43.8 - 57.8)
**Lithuania**	57.4(49.8 - 66.2)	70.5(55.9 - 88.8)	27.4(23.2 - 32.6)	143.3(117.1 - 168.9)	11.9(11.4 - 12.3)	13.3(12.6 - 14)	2.5(2.5 - 2.6)	4(3.6 - 4.4)
**Luxembourg**	91.8(81.2 - 102.2)	105.1(88.5 - 124.4)	29.6(25 - 34.7)	63.9(51.4 - 79.2)	15.7(15.3 - 16)	14.5(14.3 - 14.7)	2.7(2.7 - 2.7)	18.6(16.4 - 20.9)
**Macedonia**	57.8(50.4 - 66.2)	138.6(109.5 - 175.5)	29.6(25 - 35.1)	28.7(22.4 - 36.5)	12.0(11.5 - 12.4)	11.9(11.3 - 12.5)	2.5(2.5 - 2.6)	22.8(20.6 - 25.2)
**Madagascar**	87.6(72.9 - 104.9)	170.8(139.2 - 211.4)	5.7(4.7 - 6.7)	44.1(31.3 - 61.8)	12.3(12 - 12.7)	10.5(10.3 - 10.8)	2.6(2.6 - 2.6)	28.1(25 - 31.6)
**Malawi**	110.9(92.2 - 133.6)	239.2(194 - 294.5)	2.5(2.1 - 3)	18.9(13.3 - 26.8)	5.5(5.3 - 5.7)	15.4(15.1 - 15.7)	2.5(2.5 - 2.5)	11(9.8 - 12.4)
**Malaysia**	111.1(97.8 - 124.5)	128.4(110.8 - 149.1)	16.4(12.2 - 21.9)	18.5(15.1 - 22)	12.5(11.5 - 13.5)	9.7(8.6 - 10.8)	2.5(2.5 - 2.5)	22.6(19.1 - 27)
**Maldives**	20.9(17.7 - 24.5)	35.7(27.5 - 46.6)	0.9(0.8 - 1)	5.7(4.5 - 7.2)	8.3(7.6 - 9)	9.3(8.8 - 9.9)	2.6(2.6 - 2.6)	12.4(11 - 14)
**Mali**	67.4(56.3 - 80.2)	98.8(80.2 - 121.2)	9.7(8 - 11.4)	24.8(17.5 - 35)	8.5(8.2 - 8.7)	15.1(14.8 - 15.4)	2.6(2.6 - 2.7)	51.5(46.1 - 57.6)
**Malta**	138.9(123.2 - 154.4)	85.1(71.7 - 101)	27.3(23.1 - 32.1)	66.1(53.1 - 81.8)	14.1(13.8 - 14.5)	12.4(12.2 - 12.6)	2.7(2.7 - 2.7)	12.6(11.2 - 14.3)
**Marshall Islands**	95.5(83.3 - 107.7)	186.2(160.9 - 214.5)	8.2(6.1 - 11)	38.8(31.4 - 46.3)	13.8(12.7 - 14.9)	8.1(7.2 - 9)	2.6(2.6 - 2.6)	7.5(6.3 - 8.9)
**Mauritania**	19.6(16.4 - 23.4)	67.8(54.9 - 83.1)	28.5(23.8 - 33.5)	36.6(26 - 51.2)	12.5(12.2 - 12.8)	15.2(14.9 - 15.6)	2.6(2.6 - 2.6)	67.8(61.9 - 74.2)
**Mauritius**	69.7(58.2 - 83.3)	174.1(142.1 - 213.5)	17.3(14.1 - 20.6)	64.4(45.7 - 90.6)	9.4(9.2 - 9.7)	9.8(9.6 - 10)	2.7(2.7 - 2.7)	26.7(23.7 - 30.1)
**Mexico**	81.4(69.8 - 94.3)	87.7(72.6 - 105.2)	18.5(15.5 - 21.7)	29.6(23.1 - 38)	9.6(8.8 - 10.4)	11.3(10.8 - 11.9)	2.3(2.3 - 2.4)	16.1(14.6 - 17.9)
**Moldova**	64.3(55.9 - 73.7)	79.2(62.4 - 100.5)	53(45.4 - 61.7)	156.7(123.5 - 197.1)	12.9(12.5 - 13.4)	17.3(16.4 - 18.2)	2.5(2.5 - 2.6)	13.2(11.9 - 14.6)
**Mongolia**	45.7(39.8 - 52.5)	186.3(148 - 233.7)	66.9(57.8 - 77.1)	114.3(90.2 - 145.1)	12.3(11.8 - 12.7)	14.7(14 - 15.5)	2.5(2.5 - 2.6)	32.5(29.4 - 35.9)
**Montenegro**	53.5(46.6 - 61.3)	117.9(93.8 - 148.4)	29.9(25 - 35.8)	72.5(59.6 - 86)	11.9(11.5 - 12.4)	8.5(8.1 - 9)	2.5(2.5 - 2.6)	33.1(30.1 - 36.5)
**Morocco**	61.4(50 - 75.4)	170.4(129.7 - 223.4)	10.4(8.4 - 12.2)	18.6(14.2 - 24.1)	6.8(6.6 - 7)	13.6(12.9 - 14.4)	2.5(2.4 - 2.5)	51.6(44.6 - 59.7)
**Mozambique**	101.1(83.9 - 121.6)	148(120.8 - 181.4)	1.3(1.1 - 1.5)	7.1(5 - 9.9)	9.7(9.4 - 10)	6.8(6.7 - 7)	2.5(2.5 - 2.6)	28.4(25.2 - 32.1)
**Myanmar**	83.6(73.3 - 94)	101.5(87.5 - 117.6)	3.4(2.5 - 4.5)	3.9(3.2 - 4.6)	12.6(11.6 - 13.6)	7.2(6.3 - 8.1)	2.6(2.6 - 2.6)	6.1(5.1 - 7.2)
**Namibia**	83.0(69.2 - 99.3)	155.5(127.3 - 190.2)	13.1(10.7 - 15.5)	55.8(39.3 - 77.8)	12.5(12.1 - 12.8)	18.4(18.1 - 18.8)	2.6(2.6 - 2.6)	29.3(26 - 33)
**Nepal**	14(11.9 - 16.4)	187.2(145.6 - 240.5)	0.6(0.5 - 0.7)	9.7(7.7 - 12)	3.9(3.5 - 4.3)	5.5(5.2 - 5.8)	2.3(2.3 - 2.3)	14.7(13.2 - 16.4)

**Netherlands**	111.7(99.4 - 123.9)	110.7(93.7 - 130.5)	19.7(16.7 - 23.2)	34.2(27.7 - 42.2)	14.7(14.3 - 15.1)	11.1(11 - 11.3)	2.7(2.7 - 2.8)	17.6(15.6 - 19.9)
**New Zealand**	171.9(152.2 - 191.8)	127(107 - 150.6)	26.5(22.5 - 31.3)	59(47.7 - 73.2)	17.5(17.1 - 17.9)	15(14.8 - 15.2)	2.7(2.7 - 2.7)	21.1(18.5 - 23.9)
**Nicaragua**	109.5(94 - 127.3)	116.5(96.8 - 140.9)	22(18.3 - 25.6)	71.5(55.6 - 91.1)	10.1(9.4 - 11)	15.1(14.4 - 15.9)	2.6(2.5 - 2.6)	8.9(8.1 - 9.9)
**Niger**	63.1(52.6 - 75.7)	109.6(89.2 - 134.8)	6.1(5 - 7.2)	12.3(8.7 - 17.3)	12.2(11.9 - 12.5)	16.2(15.9 - 16.5)	2.6(2.6 - 2.6)	21.8(19.3 - 24.5)
**Nigeria**	34.2(28.6 - 41)	99.4(80.7 - 122.1)	8.5(7 - 10.1)	31(21.9 - 43.5)	9.7(9.4 - 10)	13.2(13 - 13.5)	2.6(2.5 - 2.6)	10.6(9.4 - 11.9)
**Norway**	88.9(79 - 98.8)	75.5(63.6 - 89.5)	20.4(17.3 - 24)	61(49.1 - 75.9)	13.8(13.5 - 14.2)	11.4(11.2 - 11.5)	2.7(2.6 - 2.7)	20.8(18.4 - 23.6)
**Oman**	101.7(82.7 - 125.1)	166.8(127 - 218.8)	10.9(8.8 - 13)	25.3(19.4 - 33)	9.6(9.3 - 9.8)	11.9(11.3 - 12.6)	2.7(2.7 - 2.7)	22.8(19.6 - 26.6)
**Pakistan**	33.1(28.1 - 38.9)	87.2(67.9 - 112.5)	4.7(4.2 - 5.4)	20.6(16.3 - 25.8)	7.5(6.9 - 8.1)	5.2(5 - 5.5)	2.6(2.6 - 2.6)	14.2(12.8 - 15.9)
**Palestine**	100.9(82.1 - 124)	164.8(126.1 - 216)	12.1(9.7 - 14.3)	34.1(26.3 - 44.3)	12.1(11.8 - 12.4)	6.7(6.3 - 7.1)	2.7(2.7 - 2.7)	24.7(21.3 - 28.6)
**Panama**	119(102.1 - 137.6)	90(74.3 - 109)	36.1(30.6 - 41.6)	68.8(53.5 - 87.9)	9.4(8.6 - 10.1)	12.4(11.8 - 13)	2.7(2.7 - 2.7)	8.2(7.4 - 9)
**Papua New Guinea**	52.5(46.5 - 58.4)	84.7(73.3 - 98)	6.7(5 - 8.9)	32.9(26.5 - 39.5)	13.5(12.4 - 14.5)	7.6(6.7 - 8.5)	2.6(2.6 - 2.6)	13.1(11 - 15.6)
**Paraguay**	163.1(140.2 - 189.1)	76.1(62.8 - 92.4)	30.8(26 - 35.8)	72.2(56.2 - 92.7)	8.6(7.9 - 9.3)	8.5(8.1 - 9)	2.6(2.6 - 2.7)	13(11.7 - 14.3)
**Peru**	69(59.2 - 80.4)	98.4(82 - 119.1)	24(20 - 27.9)	77.3(59.8 - 99.5)	7.4(6.7 - 8)	16.9(16.1 - 17.6)	2.6(2.6 - 2.6)	2.6(2.4 - 2.9)
**Philippines**	111.2(98.5 - 124.3)	111.7(96.8 - 129.6)	11.3(8.5 - 15.1)	18.7(15.1 - 22.2)	20.8(19.5 - 22.1)	9.1(8.2 - 10)	2.4(2.3 - 2.4)	6.2(5.2 - 7.4)
**Poland**	172.4(149.9 - 198.1)	158.7(126.1 - 199.5)	38.5(32 - 46)	76.1(61.3 - 90.8)	13.2(12.7 - 13.7)	18.1(17.2 - 19)	2.5(2.4 - 2.5)	12.9(11.7 - 14.2)
**Portugal**	120.3(106.7 - 133.8)	149.5(125.9 - 176.7)	6.8(5.8 - 8)	47.2(38.3 - 58.4)	8.9(8.6 - 9.2)	12.3(12.2 - 12.5)	2.7(2.7 - 2.7)	12.6(11.1 - 14.3)
**Qatar**	113.4(92.9 - 137.8)	170.0(129.8 - 222.4)	14.1(11.5 - 16.7)	35.1(27.1 - 45.4)	9.6(9.4 - 9.8)	12.1(11.4 - 12.8)	2.7(2.7 - 2.7)	19.5(16.8 - 22.6)
**Romania**	176.8(154 - 202.7)	325.0(256.0 - 408.7)	73.9(61.7 - 88.2)	138.0(108.7 – 175.0)	11.6(11.2 - 12.1)	15.1(14.3 - 15.9)	2.2(2.2 - 2.2)	12.4(11.2 - 13.7)
**Russia**	67(58.4 - 76.9)	100.1(79.4 - 125.6)	29.8(25 - 35.6)	192.3(152.6 - 242.1)	13(12.6 - 13.5)	13.9(13.1 - 14.6)	2.5(2.5 - 2.6)	11.7(10.6 - 13)
**Rwanda**	118.9(99 - 142.5)	297.7(241.9 - 367.6)	1(0.8 - 1.2)	4.4(3.2 - 6.3)	12.1(11.8 - 12.5)	34.9(34.5 - 35.3)	2.5(2.5 - 2.6)	7.6(6.7 - 8.5)
**Saint Lucia**	142.7(122.6 - 166.4)	76.8(63.6 - 92.3)	4.5(3.7 - 5.3)	27.2(21.2 - 35)	8.4(7.7 - 9.1)	6.6(6.3 – 7.0)	2.4(2.4 - 2.5)	21.4(19.5 - 23.6)
**Saint Vincent and the Grenadines**	59.4(51.3 - 69)	49.9(41.1 - 60.3)	7.4(6.1 - 8.7)	29.9(23.4 - 38.5)	11.9(11.1 - 12.8)	8.2(7.8 - 8.7)	2.5(2.5 - 2.6)	5.5(5 - 6.1)
**Samoa**	127.2(110.9 - 143.3)	365.7(315.1 - 425.1)	7.7(5.8 - 10.2)	53.3(43.1 - 63.6)	27.5(26.1 - 28.9)	7.5(6.6 - 8.3)	2.6(2.6 - 2.6)	9.6(8.1 - 11.3)
**Sao Tome and Principe**	54.1(44.7 - 65.2)	32.9(26.6 - 40.7)	6.6(5.2 - 8)	3.5(2.3 - 4.7)	12.5(12.2 - 12.8)	10.6(10.4 - 10.8)	2.6(2.6 - 2.6)	23.5(20.7 - 26.6)
**Saudi Arabia**	96(78.3 - 118.2)	240(183.9 - 315.8)	43.7(36.7 - 50.6)	42.8(32.7 - 55.5)	11.4(11.2 - 11.7)	12.3(11.6 - 13)	2.7(2.7 - 2.7)	39.3(33.8 - 45.5)
**Senegal**	50.8(42.4 - 60.4)	78.3(63.6 - 95.8)	19(15.6 - 22.4)	12.7(8.9 - 17.6)	7.8(7.6 - 8.1)	11(10.8 - 11.2)	2.6(2.6 - 2.6)	37.5(33.3 - 42.4)
**Serbia**	134.2(116.8 - 154.6)	220.8(175.2 - 276.8)	28.4(23.9 - 33.8)	82.4(65.2 - 104.1)	15.7(15.1 - 16.2)	11.3(10.6 - 12)	2.5(2.5 - 2.6)	41.5(37.4 - 45.9)
**Seychelles**	113.1(94.2 - 135.6)	210.2(170.1 - 258.7)	7.4(6 - 8.7)	24.3(17.2 - 33.8)	11.8(11.4 - 12.1)	13.9(13.6 - 14.2)	2.6(2.6 - 2.6)	20.2(17.9 - 22.7)
**Sierra Leone**	87.1(72.5 - 104.7)	124.3(100.2 - 152.7)	31.7(26.2 - 37)	15(10.6 - 21.3)	12.4(12.1 - 12.8)	10.6(10.4 - 10.8)	2.6(2.6 - 2.6)	21.8(19.3 - 24.5)

**Singapore**	138.6(120.3 - 156.3)	144.3(123.9 - 167.2)	9.4(7 - 12.5)	19.7(16 - 23.4)	10.3(9.4 - 11.3)	6.7(6 - 7.5)	2.6(2.6 - 2.6)	18.8(15.7 - 22.2)
**Slovakia**	60.1(52.4 - 68.9)	91.1(72.4 - 114.7)	24.5(20.5 - 29.3)	47.5(38.6 - 56.4)	14.1(13.7 - 14.6)	11.1(10.4 - 11.7)	2.5(2.5 - 2.6)	42.4(38.5 - 46.7)
**Slovenia**	69.5(60.2 - 79.9)	100(79.1 - 126.6)	16.8(14.1 - 20)	43(35.3 - 50.8)	10.9(10.5 - 11.4)	10.4(9.8 - 10.9)	2.5(2.5 - 2.6)	13.3(12 - 14.7)
**Solomon Islands**	55.7(49.3 - 62)	181(156.6 - 209.7)	14.8(11.2 - 19.3)	34.5(28.1 - 40.9)	12.7(11.7 - 13.7)	7.5(6.6 - 8.4)	2.6(2.5 - 2.6)	3.2(2.7 - 3.9)
**South Africa**	64.5(53.6 - 77.5)	129.7(105.3 - 158.5)	12.1(9.8 - 14.3)	160(125.7 - 196.3)	10.1(9.8 - 10.4)	10.9(10.7 - 11.1)	2.7(2.7 - 2.8)	23.6(20.9 - 26.6)
**South Korea**	67.2(58 - 76.6)	130.5(112.3 - 151.4)	3(2.3 - 4)	27.6(22.3 - 32.9)	4.9(4.3 - 5.4)	9.3(8.3 - 10.3)	2.3(2.3 - 2.3)	5.1(4.3 - 6.1)
**South Sudan**	68.2(56.9 - 81.7)	148.5(121.1 - 182.8)	2.3(1.9 - 2.7)	26.9(19 - 37.4)	11.4(11.1 - 11.7)	13.7(13.4 - 13.9)	2.6(2.5 - 2.6)	27.6(24.5 - 31)
**Spain**	105.1(93 - 117)	94.2(79.3 - 111.9)	20.7(17.7 - 24.3)	51.5(41.4 - 64.1)	12.4(12.1 - 12.8)	10.8(10.7 - 11)	2.7(2.7 - 2.7)	25.2(22.3 - 28.5)
**Sri Lanka**	70.7(60.7 - 82.4)	186.2(143.9 - 238.4)	6.3(5.5 - 7.1)	55.1(43.6 - 69.3)	10.8(10 - 11.6)	10.6(10.2 - 11.1)	2.3(2.3 - 2.3)	8.5(7.6 - 9.5)
**Sudan**	78.7(65.4 - 94.3)	135.2(110.2 - 165)	3.3(2.7 - 3.9)	29.4(20.9 - 41.1)	10.9(10.6 - 11.2)	12.5(12.3 - 12.8)	2.5(2.5 - 2.5)	28.9(25.7 - 32.6)
**Suriname**	61.5(52.8 - 71.5)	189.1(156.5 - 229.5)	28.1(23.6 - 32.6)	40.3(31.3 - 51.6)	7.5(6.9 - 8.2)	9.3(8.9 - 9.7)	2.5(2.5 - 2.6)	13.9(12.6 - 15.3)
**Swaziland**	89(73.3 - 107.3)	148.2(119.9 - 183.4)	8.9(7.1 - 10.8)	27.7(19.5 - 38.7)	8.8(8.6 - 9.1)	9.6(9.5 - 9.8)	2.5(2.5 - 2.5)	27.9(24.6 - 31.5)
**Sweden**	109.5(97.1 - 122.3)	79.7(67.4 - 94.6)	19.3(16.4 - 22.8)	61.3(49.4 - 75.4)	15.4(15 - 15.8)	23.8(23.4 - 24.1)	2.5(2.5 - 2.6)	13.1(11.5 - 14.9)
**Switzerland**	162.6(144.3 - 181.6)	103.6(87 - 123)	17.2(14.6 - 20.3)	64.1(51.7 - 78.9)	14.5(14.1 - 14.9)	13.5(13.3 - 13.6)	2.6(2.6 - 2.6)	15.5(13.7 - 17.6)
**Syria**	98.2(80.2 - 120.2)	143.6(109.6 - 187.3)	8.2(6.7 - 9.8)	32.7(25.1 - 42.8)	13.2(12.9 - 13.5)	8.8(8.2 - 9.3)	2.7(2.7 - 2.8)	26.3(22.7 - 30.4)
**Taiwan**	140.0(123.2 - 156.9)	238.6(207.1 - 275.7)	13.4(10 - 17.8)	50.8(40.8 - 60.4)	7.9(7.2 - 8.7)	7.1(6.3 - 7.8)	2.8(2.7 - 2.8)	5.5(4.6 - 6.6)
**Tajikistan**	81.3(71 - 92.7)	63(50 - 79.2)	87.9(78.1 - 99.7)	25.2(19.8 - 31.7)	12.8(12.4 - 13.3)	28.2(27.1 - 29.4)	2.5(2.4 - 2.5)	11(9.9 - 12.2)
**Tanzania**	62.7(52.3 - 75.1)	178.9(145.9 - 220.2)	2.1(1.7 - 2.5)	43.8(31.1 - 61.1)	12.3(12 - 12.6)	10.5(10.3 - 10.8)	2.6(2.6 - 2.6)	13.1(11.6 - 14.7)
**Thailand**	91.7(80.5 - 102.7)	169.8(147 - 195.5)	8(6 - 10.6)	27.2(22.2 - 32.3)	11.1(10.2 - 12.1)	10.3(9.2 - 11.4)	2.4(2.4 - 2.4)	2.9(2.4 - 3.4)
**The Bahamas**	68.5(58.8 - 79.8)	104.9(86.5 - 126.4)	13.6(11.3 - 15.9)	90.9(70.5 - 116.6)	11.1(10.3 - 11.9)	7(6.6 - 7.4)	2.5(2.5 - 2.6)	13.6(12.3 - 14.9)
**The Gambia**	12(10 - 14.5)	61.5(49.6 - 76)	15.9(12.5 - 19.2)	17.8(12.6 - 25.1)	12.4(12.2 - 12.8)	10.6(10.4 - 10.9)	2.6(2.6 - 2.6)	42.0(37.2 - 47.5)
**Timor-Leste**	57.1(49.6 - 64.6)	148.7(128 - 172.3)	7.7(5.7 - 10.1)	22.9(18.6 - 27.2)	12.7(11.7 - 13.7)	7.4(6.5 - 8.3)	2.6(2.6 - 2.6)	6.1(5.1 - 7.3)
**Togo**	57.5(47.9 - 68.9)	175(141.2 - 214.3)	14.3(11.7 - 16.9)	26.3(18.6 - 37)	12.4(12.1 - 12.7)	10.5(10.3 - 10.7)	2.6(2.6 - 2.6)	14.1(12.6 - 15.9)
**Tonga**	109.3(97.5 - 122)	142.5(122.3 - 165.1)	8.1(6.1 - 10.8)	36.5(28.7 - 44.2)	13.9(13 - 14.9)	7.8(6.5 - 9)	2.6(2.6 - 2.6)	5.6(4.7 - 6.7)
**Trinidad and Tobago**	67.5(58 - 78.3)	62.2(51.4 - 75.2)	16.3(13.6 - 19.1)	29.7(23 - 38)	10.5(9.7 - 11.3)	8.9(8.4 - 9.4)	2.6(2.6 - 2.6)	8.5(7.7 - 9.4)
**Tunisia**	64.1(52.3 - 78.4)	114.4(86.2 - 149.2)	0.6(0.5 - 0.7)	37.4(28.4 - 48.8)	8.6(8.4 - 8.8)	13(12.2 - 13.7)	3.1(3 - 3.1)	27.0(23.2 - 31.4)
**Turkey**	79.2(64.6 - 97)	110(84.8 - 143.9)	3.2(2.6 - 3.8)	40.3(30.8 - 52.4)	9.3(9.1 - 9.6)	11.2(10.5 - 11.8)	2.8(2.8 - 2.8)	19.4(16.8 - 22.4)
**Turkmenistan**	38.1(33.2 - 43.6)	77.6(61.6 - 98.2)	57(48.9 - 66.8)	39.6(31 - 50.4)	12.9(12.5 - 13.4)	13.8(13.1 - 14.5)	2.5(2.5 - 2.5)	6.7(6 - 7.4)
**Uganda**	56.0(46.8 – 67.0)	115.7(94 - 142.2)	0.7(0.6 - 0.9)	10.9(7.7 - 15.3)	12.2(11.9 - 12.6)	10.5(10.3 - 10.7)	2.6(2.5 - 2.6)	10.6(9.4 - 11.9)
**Ukraine**	73.6(64 - 84.4)	167.4(132.1 - 210.4)	52.1(44.7 - 60.7)	86(70.5 - 101.5)	13(12.6 - 13.5)	14.2(13.4 - 15)	2.5(2.5 - 2.6)	8.1(7.3 - 8.9)

**United Arab Emirates**	136.2(111.5 - 165.3)	160.4(122.7 - 208.9)	52.1(44.5 - 59.8)	111.2(85.5 - 145.4)	9.6(9.4 - 9.8)	9.5(8.7 - 10.3)	2.4(2.4 - 2.5)	18.7(16.1 - 21.6)
**United Kingdom**	97.1(85.8 - 108.4)	113(95.2 - 133.4)	25.9(22 - 30.5)	39.9(32.3 - 49.3)	13.7(13.3 - 14.1)	13.8(13.7 - 14)	2.6(2.6 - 2.6)	14.5(12.8 - 16.3)
**United States**	94.3(83.8 - 104.6)	124.7(105.7 - 147.4)	18.8(15.9 - 22.1)	37.6(30.3 - 46.6)	11.5(11.1 - 11.8)	12.8(12.6 - 13)	2.7(2.7 - 2.7)	14.9(13.2 - 17)
**Uruguay**	94.9(81.2 - 110.7)	44.6(36.7 - 53.7)	29(24.1 - 33.6)	116.2(90.9 - 147.7)	10.9(10.1 - 11.8)	11.7(11.1 - 12.3)	2.6(2.6 - 2.6)	11.5(10.4 - 12.7)
**Uzbekistan**	79.5(69.3 - 91.2)	139.4(110 - 176)	42.3(35.9 - 49.7)	38.2(30 - 48.6)	12.9(12.4 - 13.4)	24.2(23.1 - 25.4)	2.5(2.5 - 2.5)	14.9(13.5 - 16.4)
**Vanuatu**	43.6(39 - 48.6)	74.0(63.4 – 86.0)	9.5(7.1 - 12.7)	34.8(27 - 42.4)	12.9(12 - 13.8)	7.3(6.2 - 8.4)	2.6(2.6 - 2.6)	11.1(9.3 - 13.3)
**Venezuela (Bolivarian Republic of)**	132.2(113.5 - 154.4)	44.5(36.9 - 53.7)	39.9(33.8 - 46)	57.3(44.7 - 73.4)	4.6(4.2 - 5.2)	5.7(5.3 - 6.1)	2.5(2.5 - 2.5)	7.6(6.9 - 8.4)
**Vietnam**	76.2(66.5 - 86)	180.9(156 - 207.7)	1.0(0.7 - 1.3)	36.3(29.4 - 43.2)	5.5(4.9 - 6.1)	3.7(3.1 - 4.3)	2.2(2.2 - 2.2)	2.8(2.3 - 3.3)
**Yemen**	14.0(11.4 - 17.3)	68.5(52.2 - 89.4)	7.1(5.8 - 8.5)	17.0(12.9 - 22.1)	8.2(8 - 8.5)	12.7(12 - 13.3)	2.6(2.6 - 2.7)	24.0(20.7 - 27.9)
**Zambia**	83.6(69.5 - 100)	265.4(216.3 - 326.7)	9.5(7.8 - 11.3)	49.4(34.9 - 69.4)	12.5(12.2 - 12.9)	10.6(10.3 - 10.8)	2.6(2.6 - 2.6)	26.7(23.6 - 30)
**Zimbabwe**	81.6(67.6 - 98)	147.6(120.3 - 181.9)	4(3.2 - 4.7)	43.4(30.7 - 61.2)	12.5(12.1 - 12.8)	13.8(13.5 - 14.1)	2.6(2.6 - 2.6)	18.8(16.6 - 21.2)

Supplementary Table 4: National mean (95% UI) intakes of eight nutrients among males in 2018 across 185 countries and regions.

Country/Region	Fruits	Non-starchy vegetables	Total processed meats	Unprocessed red meats	Saturated fat	Monounsaturated fatty acids	Total omega-6 fat	Dietary fiber
**Afghanistan**	62.5(53.3 – 73.0)	95.7(74.5 - 122.6)	1.7(1.5 - 1.9)	7.1(5.7 - 8.8)	7.3(6.8 – 8.0)	4.9(4.7 - 5.2)	2.6(2.6 - 2.6)	47.7(42.8 – 53.0)
**Albania**	116.5(101.5 - 133.1)	113.5(89.6 - 143.2)	72(62.3 - 83.4)	61.7(48.1 - 78.8)	11.5(11.1 - 12)	14.6(13.9 - 15.3)	2.4(2.4 - 2.5)	27.9(25.2 - 30.7)
**Algeria**	80.6(65.6 - 98.4)	201.9(154.4 - 262.9)	7.3(5.9 - 8.6)	37(28.3 - 48.3)	12.3(12 - 12.6)	16.1(15.3 - 16.9)	2.6(2.6 - 2.6)	24.5(21.1 - 28.5)
**Angola**	119(98.8 - 142.3)	322.7(263.6 - 393.1)	1.3(1.1 - 1.6)	28.3(19.9 - 39.9)	10.7(10.4 - 11)	10.6(10.4 - 10.9)	2.6(2.6 - 2.7)	29(25.6 - 32.6)
**Antigua and Barbuda**	91.5(78.4 - 106.5)	225.5(185.8 - 271.4)	33.6(28.6 - 38.7)	31.3(24.4 - 39.9)	9.7(8.9 - 10.4)	8(7.6 - 8.4)	2.6(2.6 - 2.7)	23.5(21.3 - 25.9)
**Argentina**	92.2(79.4 - 107.1)	122.9(101.9 - 147.4)	22.6(18.8 - 26.3)	89.9(69.9 - 114.9)	11.4(10.6 - 12.3)	11.4(10.8 - 11.9)	2.6(2.6 - 2.6)	11.9(10.8 - 13.1)
**Armenia**	76.6(66.7 - 87.7)	85.5(67.9 - 107.8)	108.7(98 - 120.7)	56.7(44.4 - 72.2)	11.1(10.7 - 11.5)	19.2(18.3 - 20.2)	2.5(2.4 - 2.5)	32.4(29.3 - 35.8)
**Australia**	129.2(117 - 142.3)	108.5(92 - 127.3)	18.7(15.8 - 22)	63.3(50.8 - 78.6)	13(12.6 - 13.3)	12.1(11.9 - 12.2)	2.7(2.6 - 2.7)	18.8(16.6 - 21.3)
**Austria**	94.5(85.5 - 104.5)	97(82.5 - 114.7)	57.1(48.5 - 67.5)	103.5(83.6 - 128.7)	16.4(16 - 16.8)	13.3(13.1 - 13.4)	2.8(2.8 - 2.8)	20(17.6 - 22.7)
**Azerbaijan**	87.5(76.1 - 100.5)	72.8(57.3 - 91.8)	100.4(89.7 - 112)	85(66.8 - 108.6)	8.7(8.3 - 9)	21(20.2 - 21.9)	2.4(2.4 - 2.5)	16.6(15 - 18.3)
**Bahrain**	114.7(93.6 - 139.8)	178.1(136.2 - 231.9)	18.6(15.1 - 22.2)	37.0(28.3 - 47.9)	10.0(9.7 - 10.2)	13.3(12.6 - 14)	2.7(2.7 - 2.7)	9.1(7.9 - 10.5)
**Bangladesh**	51.6(44.1 - 60.5)	202.4(156.6 - 260.5)	0.4(0.3 - 0.4)	7(5.6 - 8.8)	3.4(3 - 3.8)	4.1(3.8 - 4.4)	2.4(2.4 - 2.5)	23.2(20.8 - 25.9)
**Barbados**	120(102.9 - 140.4)	135.2(111.7 - 162.5)	11.3(9.3 - 13.2)	64.1(49.9 - 81.6)	9.3(8.6 - 10.1)	8.2(7.7 - 8.7)	2.8(2.8 - 2.9)	36.1(32.6 - 39.9)
**Belarus**	202.1(176.6 - 230.6)	73.5(58.5 - 91.8)	36.4(30.6 - 43.3)	66.2(52 - 84)	13.3(12.8 - 13.7)	13.7(13 - 14.5)	2.6(2.5 - 2.6)	29.1(26.4 - 32.2)
**Belgium**	95.3(86 - 105.5)	96(81.2 - 113.2)	28.7(24.2 - 33.9)	55(44.4 - 68.6)	15.4(15 - 15.8)	13.4(13.2 - 13.6)	2.7(2.7 - 2.7)	15.6(13.8 - 17.7)
**Belize**	82.4(70.8 - 95.8)	147.7(121.8 - 178.8)	46.9(39.8 - 53.9)	100.2(78.4 - 127.6)	7.6(7 - 8.4)	10.6(10 - 11.1)	2.6(2.5 - 2.6)	14.8(13.4 - 16.3)
**Benin**	73.2(61.4 - 87.6)	101(82.3 - 124.2)	18.7(15.5 - 22)	26.2(18.6 - 36.4)	7.9(7.7 - 8.2)	14.2(13.9 - 14.5)	2.6(2.6 - 2.6)	13.9(12.3 - 15.7)
**Bhutan**	91.4(78.4 - 107.2)	274(213.8 - 351.8)	1.5(1.3 - 1.7)	15.6(12.4 - 19.5)	6.8(6.3 - 7.4)	6.3(5.9 - 6.6)	2.6(2.6 - 2.6)	30.8(27.6 - 34.4)
**Bolivia**	86(73.8 - 100.1)	116.9(96.9 - 141)	12.4(10.3 - 14.5)	101.1(78.9 - 129.7)	11.3(10.5 - 12.2)	10.9(10.4 - 11.5)	2.6(2.5 - 2.6)	8.4(7.6 - 9.3)
**Bosnia and Herzegovina**	462.6(407.9 - 525.2)	393.2(312.4 - 490.7)	27.8(23.3 - 33.2)	137.9(108.5 - 175)	9.3(8.9 - 9.7)	18.6(17.7 - 19.5)	2.5(2.4 - 2.5)	16.9(15.2 - 18.6)
**Botswana**	49.8(41.6 - 59.7)	109.7(89.3 - 134.2)	8(6.5 - 9.4)	41.9(29.7 - 59.2)	8.6(8.3 - 8.9)	11.9(11.7 - 12.1)	2.7(2.7 - 2.7)	37.1(33.1 - 41.7)
**Brazil**	79.9(68.6 - 92.7)	89.2(74.1 - 107.2)	36.4(30.5 - 42.5)	76.3(59.3 - 98.4)	10.4(9.6 - 11.2)	10.5(10 - 11)	2.7(2.7 - 2.7)	27.5(24.9 - 30.4)
**Brunei**	80.4(70.8 - 89.8)	152.1(131.4 - 176.4)	42.7(34.2 - 53.7)	74(59.8 - 88.3)	11.7(10.7 - 12.7)	9(8 - 10)	2.5(2.5 - 2.6)	15.2(12.8 - 18.1)
**Bulgaria**	78.8(68.6 - 90.8)	339.1(268 - 427)	34.4(28.9 - 41)	67.1(52.5 - 85.2)	12.4(11.9 - 12.9)	9.7(9.1 - 10.3)	3(2.9 - 3.1)	19.8(17.9 - 21.9)
**Burkina Faso**	34.6(28.9 - 41.4)	27.3(22.2 - 33.6)	5.6(4.6 - 6.6)	4.6(3.2 - 6.4)	7.8(7.6 - 8.1)	14.3(14 - 14.5)	2.6(2.6 - 2.6)	15.4(13.7 - 17.4)

**Burundi**	73.9(61.7 - 89)	193.6(156.6 - 237.3)	3.6(3 - 4.3)	20(14.1 - 28.4)	9(8.8 - 9.3)	11.7(11.4 - 11.9)	2.5(2.5 - 2.5)	26.4(23.4 - 29.7)
**Cambodia**	48.3(42.6 - 53.9)	111.6(96 - 129.4)	6.3(4.7 - 8.4)	17.9(14.6 - 21.4)	11.8(10.8 - 12.9)	7.0(6.2 - 7.9)	2.6(2.6 - 2.6)	5.7(4.8 - 6.7)
**Cameroon**	82.5(68.7 - 99.1)	128.1(103.8 - 156.9)	2.3(1.9 - 2.7)	20.1(14.2 - 28.1)	4.5(4.3 - 4.7)	15.9(15.6 - 16.2)	2.6(2.6 - 2.6)	30.1(26.7 - 33.9)
**Canada**	128.1(116.4 - 140.8)	110.9(93.9 - 130.9)	25(21.2 - 29.3)	44.3(35.8 - 54.9)	11.1(10.7 - 11.4)	12.1(12 - 12.3)	2.5(2.5 - 2.5)	14.2(12.5 - 16.1)
**Cape Verde**	223.2(186.8 - 266.8)	187.2(151.7 - 229.8)	5(4.1 - 5.9)	30.5(21.6 - 43)	11.5(11.2 - 11.8)	12.5(12.2 - 12.8)	2.7(2.7 - 2.7)	59.8(53.7 - 66.5)
**Central African Republic**	96(80 - 114.8)	104.2(84.8 - 127)	4(3.2 - 4.8)	117.7(82.9 - 152.6)	9.2(8.9 - 9.4)	10.2(10 - 10.4)	2.7(2.7 - 2.7)	23.6(21 - 26.5)
**Chad**	43(36.1 - 51.3)	38.5(31.1 - 47.4)	3.1(2.5 - 3.6)	51(36 - 71.6)	11.6(11.3 - 11.9)	10.7(10.5 - 10.9)	2.6(2.6 - 2.6)	15.1(13.4 - 17)
**Chile**	101.7(87.4 - 118.3)	134.2(110.8 - 162.8)	46.5(38.4 - 54.6)	20.9(16.2 - 26.7)	11(10.2 - 11.8)	10.3(9.8 - 10.8)	3.2(3.1 - 3.2)	21.4(19.3 - 23.6)
**China**	83.8(74.1 - 93.7)	281.2(242 - 325.9)	4.1(3.1 - 5.5)	93.6(75.3 - 111.5)	9.5(8.6 - 10.4)	8.2(7.2 - 9.2)	2.9(2.9 - 2.9)	20.4(17.2 - 24.2)
**Colombia**	94.7(81.6 - 110.6)	92.2(76.3 - 111.5)	62.8(52 - 73.5)	91.4(70.8 - 117.1)	9(8.3 - 9.8)	12.1(11.6 - 12.7)	2.7(2.6 - 2.7)	16.7(15.1 - 18.4)
**Comoros**	82.7(69.1 - 98.6)	97.9(79.5 - 120.6)	4.2(3.5 - 5)	37.9(26.6 - 53.4)	9.3(9 - 9.5)	11.8(11.5 - 12)	2.5(2.5 - 2.6)	21.3(18.9 - 23.9)
**Congo**	87.5(73 - 104.8)	145.1(118.5 - 178.1)	21.6(17.8 - 25.6)	36.8(25.8 - 52.1)	9.6(9.3 - 9.9)	14.6(14.3 - 14.9)	2.5(2.5 - 2.5)	26.6(23.7 - 29.9)
**Costa Rica**	125.8(108.2 - 146.3)	160(132.2 - 193.1)	38.4(32.5 - 44.5)	74.1(58.2 - 94.6)	11.1(10.3 - 12)	10.3(9.8 - 10.9)	2.6(2.6 - 2.6)	22(19.9 - 24.3)
**Cote d'Ivoire**	59.7(50.1 - 71.5)	94.9(77.1 - 117.1)	3.9(3.2 - 4.6)	20.6(14.5 - 28.9)	9.9(9.7 - 10.2)	14(13.7 - 14.2)	2.5(2.5 - 2.5)	20.6(18.3 - 23.3)
**Croatia**	49(42.9 - 55.9)	441.8(366.7 - 531.3)	111.5(95 - 131.1)	233.5(190.4 - 284.5)	12.8(12.4 - 13.3)	12.9(12.2 - 13.6)	2.5(2.5 - 2.6)	33.7(30.4 - 37.2)
**Cuba**	120.6(103.8 - 139.5)	106.8(88.1 - 129.8)	34.9(29.3 - 40.7)	144.5(113.3 - 184.1)	6.8(6.2 - 7.5)	14.2(13.5 - 14.8)	2.6(2.5 - 2.6)	6.4(5.8 - 7.1)
**Cyprus**	57.1(51.6 - 63)	103.4(87.4 - 122)	9.9(8.4 - 11.6)	68.9(55.6 - 85.1)	12(11.7 - 12.4)	13.5(13.3 - 13.7)	2.7(2.7 - 2.7)	10.9(9.6 - 12.4)
**Czech Republic**	58.9(51.5 - 67.4)	83.1(65.8 - 104.8)	38.9(32.9 - 46.2)	56.5(44.5 - 71.3)	13.4(12.9 - 13.9)	11.2(10.5 - 11.9)	2.6(2.5 - 2.6)	16.1(14.5 - 17.7)
**Democratic Republic of the Congo**	94.1(78.4 - 112)	225.2(182.7 - 277.4)	22.6(18.7 - 26.7)	19.2(13.4 - 27.2)	16.3(15.9 - 16.7)	16.2(15.9 - 16.5)	2.6(2.6 - 2.6)	23.3(20.8 - 26.3)
**Denmark**	82.5(74.9 - 90.9)	101.3(85.3 - 119.8)	32(27.2 - 37.4)	50.1(40.4 - 62)	14(13.6 - 14.3)	14.2(14 - 14.4)	2.6(2.6 - 2.6)	21.3(18.8 - 24)
**Djibouti**	135.8(113.3 - 161.9)	99.8(81 - 122.6)	15.9(13.1 - 18.8)	31.7(22.4 - 44.4)	10.3(10 - 10.6)	11.3(11.1 - 11.6)	2.6(2.6 - 2.6)	41.9(37.2 - 47)
**Dominica**	103.2(88.8 - 119.5)	100.3(82.5 - 121)	9.3(7.7 - 10.9)	23.1(18.1 - 29.8)	11(10.2 - 11.8)	12.6(12 - 13.2)	2.5(2.4 - 2.5)	14.7(13.2 - 16.2)
**Dominican Republic**	137.6(118.6 - 159.5)	112.7(93.5 - 136.2)	28.7(24 - 33.4)	49.9(38.7 - 64)	10.9(10.1 - 11.7)	10.2(9.7 - 10.7)	2.7(2.7 - 2.7)	17.2(15.5 - 19.1)
**Ecuador**	25.4(21.7 - 29.7)	107.8(88.9 - 130)	3.5(2.9 - 4)	53.0(41.0 - 67.9)	13.4(12.5 - 14.3)	8.5(8.1 - 9)	2.6(2.6 - 2.6)	19.4(17.5 - 21.3)
**Egypt**	91.1(74.5 - 111.5)	160.2(122.9 - 207.7)	5.7(4.6 - 6.8)	29(21.9 - 38.3)	7.4(7.1 - 7.6)	19.2(18.3 - 20.1)	2.6(2.6 - 2.6)	39.2(33.6 - 45.7)
**El Salvador**	118(101.5 - 136.5)	126.2(104.2 - 151.9)	40.3(34 - 46.5)	47.5(37.1 - 60.5)	7.8(7.2 - 8.5)	10.8(10.3 - 11.3)	2.6(2.6 - 2.6)	11(10 - 12.2)
**Equatorial Guinea**	87.6(73.4 - 104.6)	144.7(116.9 - 177.9)	3.4(2.8 - 4)	40.9(29.5 - 56.9)	10.3(10 - 10.6)	12(11.7 - 12.2)	2.7(2.6 - 2.7)	28.7(25.5 - 32.2)
**Eritrea**	56.9(47.2 - 68.4)	70.7(57.5 - 86.7)	3.8(3.1 - 4.4)	35.3(25 - 49.3)	9.4(9.1 - 9.6)	11.8(11.6 - 12.1)	2.6(2.5 - 2.6)	28.5(25.4 - 32.2)
**Estonia**	82.8(72.2 - 94.7)	242.6(192 - 304.8)	91.3(76.7 - 108.4)	71.3(55.7 - 90.9)	13.4(12.9 - 13.9)	12.6(12 - 13.3)	2.5(2.4 - 2.5)	15.9(14.4 - 17.6)
**Ethiopia**	39.5(32.8 - 47.4)	89.5(72.7 - 109.8)	17.4(14.4 - 20.5)	16.3(11.5 - 23.1)	6.7(6.4 - 6.9)	20.3(19.9 - 20.7)	2.4(2.4 - 2.4)	16.4(14.5 - 18.4)

**Federated States of Micronesia**	138.9(122.7 - 155.3)	133.6(114.9 - 155.1)	18.6(14 - 24.6)	25.9(21 - 30.9)	13.0(11.9 – 14.0)	7.8(7.0 - 8.8)	2.6(2.5 - 2.6)	9.1(7.7 - 10.8)
**Fiji**	105.2(92.5 - 117.7)	200.5(173.2 - 231.7)	8.1(6.1 - 11)	16.8(13.6 - 20)	16.6(15.4 - 17.8)	10.2(9.1 - 11.3)	2.5(2.5 - 2.5)	11.3(9.5 - 13.4)
**Finland**	135.3(121.9 - 149.5)	104.3(88.1 - 122.8)	61.7(52.4 - 72.4)	61.6(49.7 - 76.4)	14.1(13.7 - 14.5)	11.6(11.4 - 11.8)	2.5(2.5 - 2.5)	19.5(17.2 - 22)
**France**	154.9(140.2 - 170.8)	149.4(126.1 - 177)	35.6(30.2 - 41.9)	54.9(44.2 - 67.8)	14.8(14.4 - 15.2)	11.5(11.4 - 11.7)	2.7(2.7 - 2.7)	19.6(17.2 - 22.1)
**Gabon**	67.8(56.7 - 81.1)	121(98.7 - 148.1)	5.6(4.5 - 6.6)	48.9(34.7 - 68.9)	11.3(11 - 11.6)	13.6(13.3 - 13.9)	2.7(2.7 - 2.7)	48.1(42.8 - 54)
**Georgia**	60.2(52.5 - 69.4)	76.6(60.8 - 96.5)	66.0(56.7 - 76.5)	14(11 - 17.9)	8.8(8.5 - 9.2)	16.6(15.8 - 17.3)	2.5(2.4 - 2.5)	18.4(16.6 - 20.3)
**Germany**	127.2(115.2 - 140.1)	160(135.3 - 189)	63.7(54.1 - 75)	47.6(38.6 - 58.6)	15.1(14.7 - 15.5)	12.6(12.5 - 12.8)	2.6(2.6 - 2.6)	23.4(20.6 - 26.5)
**Ghana**	85.5(71.6 - 102.7)	137.5(111.4 - 169.2)	2.2(1.8 - 2.6)	22.1(15.6 - 30.9)	7.5(7.3 - 7.7)	8.6(8.4 - 8.9)	2.6(2.6 - 2.6)	27.6(24.5 - 31.1)
**Greece**	69.3(63 - 76.4)	75(63.3 - 88.6)	5.3(4.5 - 6.2)	73.1(58.6 - 90.3)	14.4(14.1 - 14.9)	12.6(12.5 - 12.8)	2.7(2.7 - 2.7)	29(25.6 - 32.8)
**Grenada**	116.3(100 - 135.3)	245.9(204.2 - 296.4)	7.6(6.3 - 8.9)	23.2(17.9 - 30)	11.7(10.9 - 12.5)	7.1(6.8 - 7.5)	2.6(2.6 - 2.6)	19.2(17.4 - 21.2)
**Guatemala**	71.8(61.7 - 83.5)	100.4(83.4 - 120.9)	33.7(28.3 - 39.1)	60.5(46.9 - 77.3)	5(4.5 - 5.5)	7.6(7.1 - 8)	2.7(2.7 - 2.8)	22.7(20.6 - 25.1)
**Guinea**	65.8(55 - 79)	138(112.3 - 170)	3.6(2.9 - 4.2)	33(23.6 - 46.2)	11.3(11 - 11.6)	10.2(10 - 10.4)	2.6(2.6 - 2.6)	11.2(10 - 12.7)
**Guinea-Bissau**	21.6(18.1 - 25.8)	20.7(16.8 - 25.5)	12.4(10.2 - 14.7)	20.9(14.9 - 29.5)	9.5(9.2 - 9.8)	12.5(12.2 - 12.8)	2.5(2.5 - 2.6)	20.3(18 - 22.9)
**Guyana**	194.6(167.6 - 226.9)	144.7(119.2 - 174)	62.4(54.4 - 70.5)	47.4(36.8 - 60.5)	9.5(8.8 - 10.3)	15.4(14.7 - 16)	2.4(2.3 - 2.4)	8.3(7.5 - 9.2)
**Haiti**	70.3(60.5 - 81.6)	83.8(69.3 - 101.6)	12.8(10.7 - 14.9)	15.9(12.4 - 20.3)	6.7(6.1 - 7.3)	15.6(14.9 - 16.3)	2.6(2.5 - 2.6)	7.9(7.1 - 8.7)
**Honduras**	87.9(75.1 - 102.2)	29.9(24.8 - 35.9)	49.7(42.6 - 56.6)	33.4(25.9 - 42.7)	9.6(8.9 - 10.4)	10.4(9.9 - 11)	2.5(2.5 - 2.5)	17.1(15.4 - 18.9)
**Hungary**	48.7(42.4 - 55.9)	50.4(40.1 - 63.3)	11.5(9.6 - 13.7)	18.8(14.9 - 23.8)	13.9(13.5 - 14.4)	10.6(10 - 11.3)	2.9(2.8 - 2.9)	9(8.1 - 9.9)
**Iceland**	71.1(64.4 - 78.4)	60.9(51.4 - 72.1)	24.3(20.6 - 28.7)	58.3(46.9 - 72.5)	16.6(16.2 - 17)	15.5(15.3 - 15.7)	2.5(2.5 - 2.5)	13.7(12.1 - 15.5)
**India**	36.8(31.4 - 42.9)	153.3(118.2 - 196.5)	2.4(2.1 - 2.7)	2.3(1.8 - 2.8)	6.3(5.8 - 6.9)	7.1(6.7 - 7.5)	2.8(2.8 - 2.8)	31.7(28.3 - 35.3)
**Indonesia**	64.4(56.9 - 72)	149.9(129 - 173.6)	26(20 - 33.8)	19.9(16.2 - 23.6)	15.6(14.5 - 16.8)	9(8 - 10.1)	2.6(2.6 - 2.6)	11.5(9.7 - 13.7)
**Iran**	163.2(132.4 - 201.2)	215.9(164.4 - 279.8)	5.4(4.4 - 6.4)	16(12.2 - 20.8)	9.7(9.5 - 10)	8.9(8.3 - 9.5)	2.8(2.8 - 2.9)	23.6(20.3 - 27.4)
**Iraq**	107.5(87 - 131.7)	128.5(98 - 167.2)	31.7(26 - 37.1)	50.3(38.6 - 65.6)	9.1(8.9 - 9.4)	16.1(15.2 - 16.9)	2.7(2.7 - 2.8)	15.1(13 - 17.4)
**Ireland**	75.1(68 - 82.9)	102.1(86.2 - 120.9)	35.9(30.5 - 42.3)	37.1(29.8 - 46)	14.4(14 - 14.7)	13.3(13.2 - 13.5)	2.6(2.6 - 2.6)	12.5(11 - 14.1)
**Israel**	129.5(105.9 - 160)	82.3(63.1 - 107.2)	77.7(63 - 92.2)	133.3(102 - 172.3)	7.7(7.5 - 7.9)	17.6(16.7 - 18.4)	2.7(2.6 - 2.7)	10(8.6 - 11.7)
**Italy**	191.5(173.6 - 211.2)	126.5(106.6 - 149.6)	21(17.8 - 24.8)	50.8(40.8 - 63.1)	10.8(10.5 - 11.2)	12.7(12.6 - 12.9)	2.5(2.4 - 2.5)	18.4(16.3 - 20.8)
**Jamaica**	166.7(144 - 193.1)	145.1(119.2 - 176.5)	10.9(9 - 12.8)	12.5(9.8 - 16.1)	5.7(5.1 - 6.3)	9.5(9 - 10)	2.8(2.8 - 2.9)	13.8(12.5 - 15.3)
**Japan**	91.3(80.8 - 102.1)	247.5(214.5 - 286.2)	14(10.5 - 18.4)	78.7(63.9 - 93.2)	8.1(7.3 - 8.9)	10.3(9.1 - 11.6)	2.6(2.6 - 2.6)	10(8.4 - 11.9)
**Jordan**	177.1(144.2 - 215.9)	132.7(101.7 - 172.4)	32.6(26.9 - 38.5)	49.6(38 - 64.5)	7.9(7.7 - 8.1)	6.5(6 - 6.9)	2.7(2.6 - 2.7)	54.3(46.6 - 63)
**Kazakhstan**	51.1(44.8 - 58.5)	148.1(117.4 - 186)	44.1(37.3 - 52.2)	84.6(66.8 - 107.1)	12.2(11.8 - 12.7)	14.1(13.4 - 14.9)	2.6(2.5 - 2.6)	27.0(24.3 - 29.8)
**Kenya**	101.1(84.5 - 121)	247(199.9 - 304.5)	0.8(0.6 - 0.9)	22.3(15.9 - 31.1)	10.2(9.9 - 10.5)	14(13.8 - 14.3)	2.5(2.5 - 2.5)	23.3(20.7 - 26.3)

**Kiribati**	94.4(83.6 - 105.5)	86.5(74.8 - 99.7)	19.7(15.1 - 25.9)	27.9(22.5 - 33.2)	12.2(11.2 - 13.2)	7.6(6.7 - 8.5)	2.6(2.6 - 2.6)	9.7(8.2 - 11.6)
**Kuwait**	57.1(46.5 - 70)	108(83.3 - 139.4)	29(23.6 - 34.3)	25.8(19.8 - 33.7)	10.7(10.4 - 10.9)	10.3(9.7 - 10.9)	2.6(2.6 - 2.6)	27(23.4 - 31.3)
**Kyrgyzstan**	67.1(58.5 - 76.9)	39(30.8 - 49.3)	24.3(20.4 - 29.1)	32.7(25.7 - 41.8)	10.1(9.7 - 10.5)	21.6(20.6 - 22.6)	2.5(2.4 - 2.5)	7.9(7.2 - 8.7)
**Laos**	113.9(100.7 - 127.2)	135.0(116.5 - 156.1)	12.7(9.5 - 16.8)	32.8(26.7 - 39.1)	9.5(8.7 - 10.4)	6.3(5.6 - 7)	2.5(2.5 - 2.5)	9.6(8 - 11.4)
**Latvia**	48.5(42.3 - 55.6)	193.8(153.7 - 243.9)	60.7(50.9 - 72.2)	188.7(148.8 - 238.1)	14.2(13.7 - 14.7)	12.4(11.7 - 13)	2.6(2.5 - 2.6)	30.7(27.7 - 34)
**Lebanon**	236.2(193.1 - 289.6)	371.4(284.1 - 488.6)	11.7(9.5 - 14)	26.6(20.1 - 34.6)	10(9.7 - 10.3)	22.9(22 - 23.9)	3(2.9 - 3)	35.9(30.9 - 41.6)
**Lesotho**	65.8(54.9 - 78.8)	182.5(147.4 - 223.6)	3.5(2.9 - 4.2)	36.5(25.5 - 51.3)	11.7(11.4 - 12)	10.7(10.5 - 11)	2.6(2.6 - 2.6)	40.1(35.6 - 45.1)
**Liberia**	50.2(42 - 60.3)	129.1(104.8 - 158.4)	36.6(30.7 - 42.5)	40.8(29 - 57.5)	11.9(11.6 - 12.2)	10.8(10.6 - 11.1)	2.6(2.6 - 2.7)	20.7(18.4 - 23.4)
**Libya**	53.3(43.3 - 65.2)	116.5(88.8 - 154.1)	12.2(9.9 - 14.5)	34.4(26.3 - 44.9)	12.4(12.1 - 12.6)	12.9(12.2 - 13.6)	2.6(2.5 - 2.6)	27.8(24 - 32.2)
**Lithuania**	55.6(48.5 - 63.7)	107.8(85.5 - 135.9)	42.6(36.1 - 50.2)	132.1(103.3 - 167.7)	13.3(12.9 - 13.8)	14.5(13.7 - 15.3)	2.5(2.4 - 2.5)	16.6(15 - 18.3)
**Luxembourg**	81(73.5 - 89.2)	81.4(69.1 - 96.1)	96(81.6 - 112.7)	94.6(75.9 - 116.5)	12.1(11.7 - 12.4)	13.1(12.9 - 13.3)	2.6(2.6 - 2.6)	35.7(31.7 - 40.3)
**Macedonia**	75.0(65.3 - 85.6)	125.9(100.3 - 157.1)	60.8(52.1 - 70.9)	44.1(34.5 - 55.9)	11.0(10.5 - 11.4)	12.8(12.2 - 13.5)	2.6(2.6 - 2.6)	32.8(29.6 - 36.3)
**Madagascar**	90.3(75.8 - 107)	178.8(144.5 - 219.7)	4.7(3.9 - 5.6)	46.5(33 - 65.5)	6.6(6.4 - 6.8)	4.7(4.7 - 4.8)	2.4(2.4 - 2.4)	5.5(4.8 - 6.1)
**Malawi**	110.3(92.1 - 131.3)	174.9(142.1 - 214.6)	4.6(3.8 - 5.5)	21.6(15.4 - 30.2)	5.7(5.5 - 5.9)	19(18.6 - 19.3)	2.5(2.5 - 2.5)	17.7(15.6 - 19.9)
**Malaysia**	101(89 - 113.5)	152.7(131.7 - 176.7)	12.1(9 - 16.1)	17(13.9 - 20.1)	12.1(11.2 - 13.1)	10.3(9.2 - 11.4)	2.5(2.5 - 2.6)	24.8(20.8 - 29.6)
**Maldives**	218.9(187 - 256.3)	167.3(130.1 - 216.3)	13.9(12.3 - 15.7)	24.8(19.7 - 30.7)	7.1(6.5 - 7.7)	7.7(7.4 - 8.1)	2.5(2.5 - 2.5)	61.4(55.8 - 67.6)
**Mali**	74.1(61.9 - 88.7)	99.4(80.3 - 121.8)	7.5(6.1 - 8.8)	30.8(21.8 - 43.1)	8.9(8.6 - 9.1)	14(13.7 - 14.3)	2.6(2.6 - 2.6)	53.1(47.3 - 59.4)
**Malta**	119.3(108 - 131.5)	75.2(63.5 - 88.7)	38(32.3 - 44.6)	59.5(47.9 - 73.6)	13.4(13 - 13.7)	12.6(12.4 - 12.8)	2.6(2.6 - 2.6)	21.7(19.1 - 24.4)
**Marshall Islands**	105.3(92.8 - 118.2)	174.4(150.4 - 202.1)	8.3(6.2 - 11)	26.1(21.3 - 31.1)	13.1(12.1 - 14.1)	8(7.1 - 9)	2.6(2.5 - 2.6)	12.5(10.5 - 14.8)
**Mauritania**	34.8(29.1 - 41.7)	228.2(185 - 280)	35.2(29.4 - 41.1)	28.2(20 - 39.7)	12(11.6 - 12.3)	12.8(12.6 - 13.1)	2.6(2.6 - 2.6)	52(46.4 - 58)
**Mauritius**	67.4(56.4 - 80.4)	162.5(131.8 - 200.2)	35(28.8 - 41.3)	58.5(41.5 - 82.4)	9.7(9.4 - 9.9)	11.4(11.1 - 11.6)	2.8(2.7 - 2.8)	52.3(46.8 - 58.5)
**Mexico**	144.1(123.8 - 167.5)	184.5(151.9 - 222.8)	15.4(12.8 - 18)	50.3(39.1 - 64.3)	10.9(10.1 - 11.8)	10.1(9.6 - 10.6)	2.3(2.3 - 2.4)	9.2(8.3 - 10.1)
**Moldova**	80.6(69.9 - 92.4)	76.6(60.6 - 96.1)	65.4(56.2 - 75.8)	82.2(64.5 - 104.7)	10.6(10.2 - 11)	18.4(17.5 - 19.3)	2.6(2.5 - 2.6)	11.7(10.6 - 12.9)
**Mongolia**	42.2(36.9 - 48.5)	168.7(133.5 - 210.7)	124.1(117.1 - 131.4)	72(56.5 - 91.6)	13(12.5 - 13.4)	15.7(14.9 - 16.5)	2.5(2.4 - 2.5)	21.3(19.3 - 23.6)
**Montenegro**	201.5(175.7 - 230.6)	116.9(92.5 - 147.1)	64.9(54.4 - 77.1)	182.8(143.3 - 231.3)	12.1(11.6 - 12.5)	8.9(8.4 - 9.4)	2.5(2.5 - 2.6)	58.5(53.3 - 64.2)
**Morocco**	88.9(72.6 - 108)	162.4(123.6 - 211.8)	20.4(16.6 - 24.1)	26(19.9 - 33.8)	7.4(7.2 - 7.6)	13.9(13.2 - 14.6)	2.5(2.4 - 2.5)	43.2(37.3 - 50)
**Mozambique**	97(81.1 - 116.7)	142(115.1 - 173.7)	3.5(2.8 - 4.1)	31.3(22.1 - 44.1)	7.6(7.4 - 7.8)	5.4(5.3 - 5.6)	2.5(2.5 - 2.5)	33.1(29.3 - 37.5)
**Myanmar**	77.3(68.1 - 86.3)	165.5(142.7 - 191.9)	26.5(20.4 - 34.3)	78.4(63.5 - 93.1)	11.9(10.9 - 12.9)	7.1(6.3 - 8)	2.6(2.6 - 2.6)	18.1(15.2 - 21.5)
**Namibia**	269.4(222.9 - 323.2)	161.6(131.2 - 197.8)	6.2(5.1 - 7.3)	41.3(29.1 - 58)	8.4(8.1 - 8.6)	14.1(13.8 - 14.3)	2.6(2.5 - 2.6)	37.5(33.2 - 42.3)
**Nepal**	33.9(29 - 39.7)	185.6(143.7 - 237.7)	0.7(0.6 - 0.8)	10.1(8.1 - 12.6)	3(2.7 - 3.4)	4.2(4 - 4.4)	2.3(2.3 - 2.3)	47.6(42.7 - 53.1)

**Netherlands**	100.8(91 - 111.4)	105.4(89 - 124.2)	22.5(19.1 - 26.6)	43.3(35 - 53.7)	13.7(13.3 - 14.1)	11.9(11.8 - 12.1)	2.7(2.7 - 2.7)	12.4(11 - 14.1)
**New Zealand**	138.9(126.1 - 153)	110.9(93.6 - 131.4)	29.3(24.9 - 34.5)	46.4(37.7 - 57.2)	14.5(14.1 - 14.9)	15(14.8 - 15.2)	2.6(2.6 - 2.6)	16.1(14.2 - 18.1)
**Nicaragua**	149(128.4 - 172.8)	269.5(222.8 - 324.1)	41.3(35.1 - 47.6)	37(28.8 - 47.3)	7.9(7.2 - 8.6)	11.8(11.2 - 12.4)	2.6(2.5 - 2.6)	10.3(9.3 - 11.3)
**Niger**	64.9(54.2 - 77.4)	103.9(84.6 - 126.6)	2.8(2.3 - 3.3)	6.2(4.3 - 8.7)	8.6(8.4 - 8.9)	13.6(13.3 - 13.8)	2.6(2.6 - 2.6)	28.7(25.5 - 32.2)
**Nigeria**	34.7(29.1 - 41.5)	94.3(77 - 115.3)	4.8(3.9 - 5.6)	30.4(21.5 - 42.9)	9.5(9.2 - 9.8)	12.5(12.3 - 12.7)	2.5(2.5 - 2.6)	17.3(15.4 - 19.5)
**Norway**	76.3(69.2 - 84.2)	111.4(94.3 - 131.8)	27.9(23.6 - 33)	81.2(65.6 - 100.2)	13.6(13.2 - 14)	12.8(12.6 - 12.9)	2.6(2.6 - 2.6)	15.7(13.9 - 17.7)
**Oman**	121.4(99 - 148.4)	158.3(121.2 - 207.7)	24.6(20.2 - 29)	41.4(31.6 - 54.2)	9.8(9.5 - 10)	13.2(12.5 - 13.9)	2.7(2.7 - 2.7)	29.9(25.8 - 34.6)
**Pakistan**	33.0(28.0 - 38.6)	86.7(67.0 – 113.0)	5.2(4.6 - 5.9)	24.2(19.4 - 30.2)	9.2(8.6 - 9.9)	6.2(5.8 - 6.5)	2.6(2.6 - 2.6)	15.8(14.1 - 17.7)
**Palestine**	111.5(90.5 - 136.2)	156.2(119.5 - 204.2)	15.8(12.9 - 18.7)	36.4(27.8 - 47.7)	11.9(11.6 - 12.2)	7.4(7 - 7.8)	2.7(2.7 - 2.7)	72(67.1 - 77.1)
**Panama**	124.2(106.7 - 144)	148.1(121.7 - 178.7)	43.4(37.2 - 49.8)	75(58.4 - 95.9)	8.6(7.9 - 9.4)	12.1(11.5 - 12.7)	2.7(2.6 - 2.7)	15.4(13.9 - 17)
**Papua New Guinea**	3.8(3.3 - 4.2)	81.6(70.7 - 94.3)	3.5(2.7 - 4.7)	155.6(130 - 180.7)	12.7(11.7 - 13.7)	7.5(6.6 - 8.4)	2.6(2.5 - 2.6)	3.7(3.1 - 4.4)
**Paraguay**	91.2(78.6 - 105.7)	66.9(55.4 - 80.3)	24.4(20.3 - 28.4)	131.1(102.1 - 166.3)	9.5(8.8 - 10.2)	9.4(8.9 - 9.9)	2.7(2.7 - 2.8)	10.6(9.5 - 11.7)
**Peru**	215.2(185.1 - 248.8)	90.7(75.3 - 109.5)	26.5(22.2 - 30.9)	102.7(80.3 - 131.2)	7.3(6.7 - 8)	15.3(14.6 - 16)	2.5(2.5 - 2.6)	10.3(9.3 - 11.3)
**Philippines**	86.2(75.9 - 96.7)	110.8(95.8 - 128.1)	28.7(21.4 - 38.1)	19.6(15.9 - 23.3)	20.4(19.1 - 21.6)	11.3(10.1 - 12.4)	2.4(2.4 - 2.4)	8.4(7.1 - 10)
**Poland**	196.5(170.7 - 225.3)	160.4(127.1 - 202.1)	67.6(56.7 - 80.3)	70.7(55.8 - 89.6)	12.3(11.9 - 12.8)	20.3(19.4 - 21.2)	2.5(2.4 - 2.5)	15.4(14 - 17)
**Portugal**	399(363.5 - 438.4)	139.6(118.5 - 164.9)	16.7(14.2 - 19.7)	68(54.9 - 84.3)	9.9(9.5 - 10.2)	12(11.8 - 12.1)	2.6(2.6 - 2.7)	15.5(13.7 - 17.5)
**Qatar**	120.3(97.9 - 146.7)	163.9(124 - 215.1)	18.5(15.1 - 21.9)	33.8(25.8 - 44.4)	9.9(9.6 - 10.1)	13.2(12.5 - 13.9)	2.7(2.7 - 2.7)	17.3(14.9 - 20.1)
**Romania**	152.4(132.6 - 175.2)	300.9(237.2 - 380.2)	49.9(41.9 - 59.6)	61.2(47.7 - 78)	11.3(10.8 - 11.7)	14.4(13.7 - 15.1)	2.2(2.1 - 2.2)	20.5(18.5 - 22.6)
**Russia**	73.8(64.3 - 84.6)	154.2(122 - 195.1)	53.1(44.5 - 63.1)	169(131.4 - 217)	11.1(10.6 - 11.5)	14.9(14.1 - 15.7)	2.5(2.5 - 2.6)	20.3(18.5 - 22.5)
**Rwanda**	116.9(97.1 - 140.4)	280.6(227.2 - 345)	1.3(1 - 1.5)	7.5(5.3 - 10.5)	6.4(6.3 - 6.6)	24.4(24 - 24.8)	2.4(2.4 - 2.4)	11.2(9.9 - 12.6)
**Saint Lucia**	72.3(62.1 - 84)	87.4(72.1 - 106)	6.8(5.6 - 7.9)	37.4(29.2 - 47.9)	10.2(9.4 - 11)	7.6(7.2 - 8.1)	2.4(2.4 - 2.5)	14.3(12.9 - 15.8)
**Saint Vincent and the Grenadines**	135.7(116.4 - 158.3)	100.7(83.5 - 121.9)	14.7(12.2 - 17.2)	56(43.7 - 71.3)	9.6(8.9 - 10.4)	9(8.5 - 9.5)	2.6(2.5 - 2.6)	19.5(17.7 - 21.5)
**Samoa**	136.4(120.4 - 152.5)	334.5(287.8 - 388.1)	14(10.6 - 18.4)	23.2(18.9 - 27.6)	26.7(25.3 - 28.1)	7.4(6.5 - 8.3)	2.6(2.6 - 2.6)	22.9(19.1 - 27.2)
**Sao Tome and Principe**	129.6(108.1 - 155.3)	38.4(31.3 - 47)	15(12.3 - 17.6)	13.3(9.3 - 19)	12.1(11.8 - 12.4)	10.9(10.6 - 11.1)	2.7(2.7 - 2.7)	32.4(28.6 - 36.6)
**Saudi Arabia**	143.2(117 - 174.7)	145.7(111 - 188.9)	58.9(50.2 - 67.5)	33.1(25.2 - 43.5)	11(10.7 - 11.2)	13.8(13 - 14.6)	2.7(2.7 - 2.7)	33.9(29.3 - 39.1)
**Senegal**	51.4(43 - 61.3)	72.8(59.3 - 88.9)	15.4(12.7 - 18)	12.5(8.8 - 17.6)	9.2(9 - 9.5)	13.6(13.3 - 13.9)	2.7(2.7 - 2.7)	43.8(38.9 - 49.1)
**Serbia**	117(101.9 - 134.3)	423.8(342.8 - 521.8)	31.5(26.3 - 37.4)	64.7(50.9 - 82.7)	13.5(13 - 14)	11.1(10.5 - 11.7)	2.5(2.5 - 2.6)	32.5(29.4 - 35.9)
**Seychelles**	100.8(84.4 - 120.3)	201.4(164.1 - 246.4)	11.2(9.1 - 13.2)	28.9(20.4 - 40.7)	9.5(9.2 - 9.8)	11.9(11.7 - 12.1)	2.6(2.6 - 2.6)	21.9(19.4 - 24.7)
**Sierra Leone**	83.8(69.8 - 100)	119.9(97.5 - 147.6)	56.4(48.5 - 64.3)	15.4(11 - 22)	11.8(11.5 - 12.1)	10.8(10.6 - 11)	2.6(2.6 - 2.6)	21.3(18.9 - 23.9)
**Singapore**	125.1(110.2 - 139.9)	134.6(116.2 - 156)	16(12 - 21.2)	40(32.4 - 47.6)	11.7(10.7 - 12.7)	7.5(6.6 - 8.4)	2.6(2.6 - 2.6)	18.3(15.4 - 21.6)

**Slovakia**	64.7(56.5 - 74.2)	83.3(65.6 - 104.9)	59.3(49.7 - 70.7)	30.5(23.9 - 38.6)	12.6(12.1 - 13)	11.4(10.8 - 12.1)	2.5(2.5 - 2.6)	26.5(24 - 29.1)
**Slovenia**	60.6(52.8 - 69.4)	93.1(74.1 - 116.6)	35.6(29.9 - 42.4)	49.9(39 - 63.8)	12.8(12.4 - 13.3)	11.3(10.7 - 12)	2.6(2.5 - 2.6)	19.6(17.7 - 21.6)
**Solomon Islands**	59.1(52 - 66.3)	175(151.3 - 202.8)	15.3(11.5 - 20.2)	38(30.7 - 45.2)	12.1(11.1 - 13.1)	7.4(6.5 - 8.3)	2.6(2.6 - 2.6)	3.9(3.3 - 4.6)
**South Africa**	30.3(25.3 - 36.1)	124(100.5 - 151.9)	13.8(11.3 - 16.4)	163(126.5 - 198.1)	11.4(11.1 - 11.7)	12.2(11.9 - 12.4)	2.8(2.8 - 2.8)	19.5(17.3 - 22)
**South Korea**	83.3(73.2 - 93.2)	126.9(109.3 - 147.1)	2(1.5 - 2.6)	37.4(30.3 - 44.3)	6.4(5.7 - 7.1)	6.2(5.4 - 7.1)	2.4(2.4 - 2.4)	10.2(8.6 - 12.1)
**South Sudan**	63.8(53.3 - 76.2)	143.6(116.7 - 175.9)	3.9(3.2 - 4.7)	30.6(21.5 - 43.3)	9.2(8.9 - 9.5)	11.7(11.5 - 11.9)	2.5(2.5 - 2.5)	31.2(27.7 - 35.1)
**Spain**	58.8(53.3 - 64.9)	83.4(70.9 - 98.5)	32.7(27.7 - 38.5)	53.3(42.8 - 66)	12.8(12.4 - 13.2)	11.2(11 - 11.4)	2.7(2.7 - 2.8)	15.3(13.6 - 17.4)
**Sri Lanka**	75.4(64.6 - 88.1)	189.9(147.7 - 244.9)	6.6(5.8 - 7.5)	40.7(32.6 - 50.8)	9.7(9 - 10.4)	11.8(11.3 - 12.3)	2.3(2.3 - 2.3)	10.9(9.7 - 12.1)
**Sudan**	80.4(66.9 - 95.9)	132.9(107.3 - 162.8)	5.9(4.8 - 7)	25.2(18 - 35)	10.6(10.3 - 10.8)	13.3(13.1 - 13.6)	2.5(2.5 - 2.6)	48.1(42.9 - 54)
**Suriname**	100.2(86.6 - 116.2)	177.4(147.4 - 213.5)	38.7(32.7 - 44.5)	70.6(55.1 - 90)	7(6.4 - 7.7)	11.9(11.4 - 12.5)	2.7(2.7 - 2.7)	11.7(10.6 - 12.9)
**Swaziland**	88.4(73.5 - 105.8)	166(134.7 - 204.6)	2.2(1.8 - 2.6)	33(23.3 - 46.7)	7.6(7.3 - 7.8)	10.9(10.7 - 11.1)	2.5(2.5 - 2.5)	44.5(39.6 - 49.9)
**Sweden**	103.5(93.6 - 114.3)	127.9(107.9 - 151.3)	33.1(27.9 - 39)	70.7(56.8 - 87.7)	14.6(14.2 - 15)	31.4(31.2 - 31.7)	2.5(2.5 - 2.5)	19.5(17.2 - 22)
**Switzerland**	125.2(113.3 - 137.9)	95.9(80.8 - 114)	21(17.8 - 24.8)	46.7(37.6 - 57.7)	13.4(13 - 13.8)	12.6(12.4 - 12.8)	2.6(2.6 - 2.6)	14.9(13.1 - 16.9)
**Syria**	101.7(82.9 - 124.6)	135.8(104.1 - 177.2)	12.9(10.5 - 15.3)	34.5(26.3 - 45)	13.2(12.9 - 13.5)	9.5(8.9 - 10)	2.7(2.7 - 2.8)	33.3(28.6 - 39)
**Taiwan**	130.8(115.4 - 146.1)	245.1(211.4 - 282.5)	16.7(12.4 - 22.3)	64.9(52.3 - 76.8)	10.6(9.7 - 11.5)	8.5(7.5 - 9.4)	2.8(2.8 - 2.8)	19.6(16.6 - 23.3)
**Tajikistan**	83.3(72.9 - 95.2)	58.8(46.5 - 73.5)	82(71.6 - 93.8)	45.5(35.9 - 57.8)	8.1(7.8 - 8.5)	20.9(20 - 21.8)	2.6(2.5 - 2.6)	7.2(6.5 - 8)
**Tanzania**	72.5(60.5 - 87.4)	178(144.8 - 218.3)	2.7(2.2 - 3.2)	42.5(30 - 59.5)	10.7(10.4 - 11)	9.8(9.6 - 10)	2.6(2.6 - 2.6)	15.2(13.5 - 17.1)
**Thailand**	90.6(79.9 - 101)	158.4(136.6 - 183.5)	8.9(6.7 - 11.8)	31.5(25.7 - 37.5)	10.1(9.2 - 11)	10.6(9.4 - 11.7)	2.5(2.5 - 2.5)	7.2(6 - 8.5)
**The Bahamas**	80.3(69 - 93.2)	102.1(84.4 - 123.3)	15.1(12.5 - 17.6)	47.8(37.4 - 60.9)	11.2(10.4 - 12)	7.2(6.8 - 7.7)	2.6(2.6 - 2.6)	19.9(17.9 - 22)
**The Gambia**	30.9(25.8 - 36.9)	50.2(40.9 - 61.5)	25.7(21.1 - 30.3)	11.1(7.8 - 15.6)	9.6(9.3 - 9.9)	10.1(9.9 - 10.3)	2.7(2.7 - 2.7)	19.6(17.4 - 22)
**Timor-Leste**	48.3(42.7 - 53.8)	137.4(118.6 - 159)	17.6(13.4 - 23.2)	15(12.1 - 18)	12(11 - 13.1)	7.4(6.5 - 8.3)	2.6(2.6 - 2.6)	8.8(7.4 - 10.4)
**Togo**	54.1(45 - 64.4)	149.8(121.8 - 183.6)	16.9(13.8 - 20)	20.9(14.8 - 29.7)	11.9(11.5 - 12.2)	10.8(10.6 - 11)	2.6(2.6 - 2.6)	13.6(12.1 - 15.3)
**Tonga**	126.1(111.5 - 140.4)	136.6(117.9 - 158.7)	8.2(6.1 - 10.9)	26.3(21.4 - 31.4)	13(11.9 - 14)	7.8(6.9 - 8.8)	2.6(2.5 - 2.6)	9(7.6 - 10.7)
**Trinidad and Tobago**	65.8(56.5 - 76.6)	82.9(68.6 - 100.3)	32.7(27.4 - 38)	64.3(49.8 - 83.2)	9.3(8.6 - 10.1)	8.4(7.9 - 8.8)	2.6(2.6 - 2.6)	7.7(7 - 8.5)
**Tunisia**	59.8(48.9 - 73.2)	192.4(147.2 - 249.5)	0.8(0.7 - 1)	36.6(27.8 - 47.8)	9.6(9.4 - 9.8)	13.9(13.1 - 14.6)	3.1(3 - 3.1)	39.7(34.2 - 46)
**Turkey**	75.2(61.3 - 92.2)	106.3(80.6 - 139.2)	4.4(3.6 - 5.2)	49.7(37.9 - 64.7)	10.6(10.3 - 10.9)	12.1(11.4 - 12.8)	2.8(2.8 - 2.8)	22.1(18.9 - 25.7)
**Turkmenistan**	59(51.4 - 67.7)	104.3(82.7 - 131.3)	70.2(60.6 - 81.2)	75.1(58.9 - 95.9)	11.6(11.2 - 12)	14(13.3 - 14.7)	2.5(2.5 - 2.6)	12.8(11.6 - 14.2)
**Uganda**	58.9(49.4 - 70.2)	118.8(96.6 - 145.2)	3.5(2.9 - 4.2)	20.1(14 - 28.7)	11.6(11.3 - 12)	10.7(10.5 - 10.9)	2.6(2.6 - 2.6)	16.0(14.2 – 18.0)
**Ukraine**	75(65.6 - 86)	150.1(118.3 - 189.2)	49.3(42 - 58)	35.2(27.5 - 44.9)	10.5(10.1 - 10.9)	15(14.3 - 15.8)	2.5(2.5 - 2.6)	19.8(17.9 - 21.9)
**United Arab Emirates**	103.4(84.5 - 126.2)	118.5(90 - 154.8)	28.3(23.2 - 33.3)	27.2(20.7 - 35.5)	8.3(8.1 - 8.5)	9.8(9.2 - 10.5)	2.4(2.4 - 2.5)	12.9(11 - 15)

**United Kingdom**	83.1(75.2 - 91.4)	99.6(84.3 - 117.9)	34(28.8 - 40)	40(32.2 - 49.3)	12.2(11.8 - 12.5)	14(13.8 - 14.2)	2.6(2.6 - 2.6)	10.7(9.4 - 12.1)
**United States**	88.7(80.2 - 97.8)	116.7(98.4 - 138.6)	25.1(21.3 - 29.6)	35.2(28.5 - 43.9)	11.8(11.5 - 12.2)	12(11.8 - 12.1)	2.8(2.8 - 2.8)	13(11.5 - 14.7)
**Uruguay**	89.4(77 - 103.7)	150.1(125.1 - 181)	43.4(36.7 - 50.2)	70.6(54.9 - 90.5)	11.1(10.3 - 12)	11.5(11 - 12.1)	2.6(2.5 - 2.6)	32.7(29.5 - 36.2)
**Uzbekistan**	73(63.5 - 84)	208.8(165 - 263.7)	47.9(40.7 - 56.3)	86.5(68 - 110.1)	10.1(9.7 - 10.6)	18.6(17.7 - 19.5)	2.5(2.5 - 2.6)	19.6(17.7 - 21.6)
**Vanuatu**	41.1(36.1 - 45.9)	71.5(61.9 - 82.6)	4.7(3.5 - 6.3)	36.5(29.5 - 43.6)	12.1(11.1 - 13.1)	7.5(6.6 - 8.3)	2.6(2.6 - 2.6)	17.1(14.4 - 20.3)
**Venezuela (Bolivarian Republic of)**	104.5(90.2 - 121)	89.8(74.1 - 108.8)	27.6(23.1 - 32.3)	43.4(33.8 - 55.3)	5.1(4.6 - 5.7)	6.2(5.8 - 6.5)	2.4(2.4 - 2.5)	6.9(6.2 - 7.6)
**Vietnam**	77.2(68.2 - 86.4)	164(141.2 - 189.9)	7(5.2 - 9.3)	41.6(33.7 - 49.5)	5(4.4 - 5.6)	3.7(3.1 - 4.3)	2.2(2.2 - 2.2)	6.5(5.4 - 7.6)
**Yemen**	42.0(34.3 - 51.5)	66.6(50.9 - 86.7)	14.4(11.7 - 17.1)	19.8(15.2 - 25.8)	7.8(7.6 - 8.1)	13.3(12.7 – 14.0)	2.6(2.6 - 2.6)	18.3(15.7 - 21.2)
**Zambia**	51.9(43.5 - 62.2)	206.8(168.8 - 252.6)	9.4(7.7 - 11.1)	74.0(52.7 - 102.1)	11.9(11.6 - 12.2)	10.8(10.6 – 11.0)	2.6(2.6 - 2.6)	20.1(17.8 - 22.6)
**Zimbabwe**	83.2(69.8 - 99.6)	150.9(122.1 - 184.9)	5.9(4.8 – 7.0)	51.0(36.1 - 71.4)	8.7(8.4 - 8.9)	12.3(12.0 - 12.5)	2.7(2.7 - 2.7)	17.7(15.7 - 19.9)

Supplementary Table 5: National mean (95% UI) intakes of eight nutrients among females in 2018 across 185 countries and regions.

Country/Region	Fruits	Non-starchy vegetables	Total processed meats	Total processed meats	Saturated fat	Monounsaturated fatty acids	Total omega-6 fat	Dietary fiber
**Afghanistan**	64.9(55.8 - 76.1)	97.0(74.9 - 124.2)	1.5(1.3 - 1.6)	7.7(6.1 - 9.6)	7.6(7 - 8.3)	4.7(4.5 - 5)	2.6(2.6 - 2.6)	47.7(42.7 - 52.9)
**Albania**	141.8(124 - 162.1)	128.3(101.7 - 160.7)	61.1(52.2 - 71.2)	54.2(42.7 - 68.5)	11.9(11.5 - 12.4)	13.8(13.1 - 14.5)	2.4(2.4 - 2.5)	27.8(25.1 - 30.7)
**Algeria**	90.5(73.8 - 110.2)	227.7(173.1 - 296.8)	5.9(4.8 - 7)	35.5(27.1 - 46.1)	13.1(12.8 - 13.4)	15(14.3 - 15.7)	2.6(2.6 - 2.6)	24.8(21.3 - 28.6)
**Angola**	121.2(100.9 - 145.5)	330.6(271.2 - 405.8)	1.1(0.9 - 1.4)	29.4(21 - 41)	11.1(10.8 - 11.5)	10.3(10 - 10.5)	2.7(2.6 - 2.7)	29.6(26.2 - 33.3)
**Antigua and Barbuda**	99.1(85.5 - 114.8)	235.3(195.2 - 283.4)	31.5(26.6 - 36.2)	30.3(23.5 - 38.9)	10.2(9.4 - 11)	8(7.6 - 8.4)	2.7(2.6 - 2.7)	23.9(21.6 - 26.3)
**Argentina**	95.5(81.5 - 111.4)	124.5(102.7 - 150.6)	17.9(14.8 - 20.9)	84.7(65.5 - 109.4)	11.9(11.1 - 12.8)	10.7(10.2 - 11.3)	2.7(2.6 - 2.7)	11.1(10.1 - 12.2)
**Armenia**	92.9(80.8 - 106.4)	95.3(75.6 - 120)	98.8(87.9 - 110.7)	56(44.1 - 71)	11.5(11 - 11.9)	18.2(17.3 - 19.1)	2.5(2.5 - 2.6)	32.2(29.1 - 35.7)
**Australia**	151.7(137.8 - 167)	117.2(99.2 - 137.6)	10.9(9.2 - 12.7)	42.1(34 - 52)	13.2(12.9 - 13.6)	12.0(11.8 - 12.2)	2.7(2.6 - 2.7)	18.9(16.6 - 21.5)
**Austria**	121.7(110.5 - 134.4)	121.1(102.4 - 142.6)	45.1(38.2 - 53.2)	83.5(67.6 - 103)	16.7(16.3 - 17.1)	13.2(13.1 - 13.4)	2.8(2.8 - 2.8)	21.9(19.3 - 24.7)
**Azerbaijan**	110.4(96.1 - 126.7)	78.8(62.4 - 100)	90.6(80.1 - 102.9)	80.0(62.2 - 101.9)	9.0(8.6 - 9.4)	20.0(19.2 - 20.9)	2.4(2.4 - 2.5)	16.5(14.9 - 18.2)
**Bahrain**	131.4(107 - 161)	198.5(151.5 - 259.1)	14.9(12.1 - 17.6)	35.5(27.2 - 46.5)	10.6(10.3 - 10.8)	12.4(11.7 - 13.1)	2.7(2.7 - 2.8)	8.5(7.4 - 9.9)
**Bangladesh**	54.2(46.1 - 63.7)	221.4(170.7 - 283.4)	0.3(0.3 - 0.4)	7.0(5.6 - 8.8)	3.3(2.9 - 3.7)	3.7(3.4 - 3.9)	2.4(2.4 - 2.4)	24(21.5 - 26.9)
**Barbados**	133(114.2 - 154.6)	140.8(116.6 - 170)	10.1(8.3 - 11.8)	64.5(50.4 - 82.2)	9.6(8.9 - 10.4)	8.2(7.8 - 8.7)	2.8(2.8 - 2.9)	36.9(33.5 - 40.7)
**Belarus**	248.8(217.1 - 284.7)	82.7(65.5 - 103.8)	28.8(24.3 - 34)	62.6(49.1 - 79.8)	13.7(13.2 - 14.2)	12.9(12.2 - 13.7)	2.6(2.5 - 2.6)	29.1(26.3 - 32.3)
**Belgium**	120.6(109.4 - 132.9)	117.3(99.4 - 138.4)	22.9(19.4 - 27)	48.8(39.2 - 60.6)	15.8(15.3 - 16.2)	13.3(13.2 - 13.5)	2.7(2.7 - 2.7)	17.2(15.2 - 19.4)
**Belize**	89.8(77.3 - 104.6)	155.9(129.3 - 188)	44.1(37.3 - 50.9)	97.3(75.8 - 125)	8.1(7.4 - 8.8)	10.6(10.1 - 11.1)	2.6(2.5 - 2.6)	15(13.6 - 16.5)
**Benin**	76.7(63.6 - 91.9)	105.8(86.1 - 129.7)	16.5(13.6 - 19.3)	30(21.3 - 41.8)	8.3(8 - 8.5)	13.7(13.5 - 14)	2.6(2.6 - 2.6)	14.2(12.5 - 16)
**Bhutan**	96.4(82.2 - 113.2)	277.7(215.7 - 355.9)	1.3(1.1 - 1.5)	16.9(13.5 - 21.1)	7.1(6.6 - 7.7)	6(5.7 - 6.4)	2.6(2.6 - 2.6)	30.9(27.8 - 34.5)
**Bolivia**	88.4(76 - 103.1)	113(93.2 - 136.8)	11.5(9.5 - 13.5)	90.5(70.4 - 115.8)	12.2(11.3 - 13.1)	11.3(10.8 - 11.9)	2.6(2.5 - 2.6)	8.8(7.9 - 9.7)
**Bosnia and Herzegovina**	480.5(432.5 - 533.5)	418.8(337.9 - 516.6)	21(17.6 - 25)	130.8(103.1 - 165.7)	9.6(9.3 - 10)	17.7(16.8 - 18.5)	2.5(2.4 - 2.5)	16.8(15.2 - 18.6)
**Botswana**	48.9(40.9 - 58.8)	114.4(93.2 - 139.8)	7(5.7 - 8.2)	43.8(31.1 - 61.6)	9(8.7 - 9.3)	11.5(11.3 - 11.8)	2.7(2.7 - 2.7)	37.6(33.4 - 42.3)
**Brazil**	88.9(76.5 - 103.2)	95.3(78.3 - 115.7)	33.1(27.4 - 38.6)	67.5(52.6 - 86.5)	10.8(10 - 11.7)	10.5(10 - 11.1)	2.7(2.7 - 2.7)	27.2(24.6 - 30.1)
**Brunei**	99.2(87.6 - 111.3)	167.9(144.5 - 195.1)	42.8(34 - 53.6)	75.6(62.1 - 89.8)	12.1(11.1 - 13.1)	9(8 - 10.1)	2.5(2.5 - 2.6)	15.6(13.1 - 18.6)
**Bulgaria**	95.9(83.6 - 109.8)	365.1(290.4 - 460.9)	25.2(21.1 - 30.1)	57.2(45 - 72.8)	12.7(12.3 - 13.2)	9.2(8.6 - 9.7)	3(3 - 3.1)	17.4(15.8 - 19.2)
**Burkina Faso**	35.5(29.5 - 42.4)	29.7(24.1 - 36.4)	4.9(4 - 5.8)	4.8(3.4 - 6.7)	8.2(7.9 - 8.4)	13.8(13.5 - 14.1)	2.6(2.6 - 2.7)	15.8(14 - 17.6)

**Burundi**	75.8(63.4 - 90.4)	204.1(165.8 - 251.4)	3.2(2.6 - 3.8)	20.1(14.3 - 28.3)	9.4(9.1 - 9.7)	11.2(11 - 11.5)	2.5(2.5 - 2.5)	26.8(23.9 - 30.1)
**Cambodia**	57.2(50.6 - 63.9)	127.7(110.3 - 147.2)	6.4(4.8 - 8.5)	21.9(17.9 - 26)	12.3(11.3 - 13.2)	7.1(6.2 - 7.9)	2.6(2.6 - 2.6)	5.8(4.9 - 6.9)
**Cameroon**	83.9(69.9 - 100.9)	132.8(107.9 - 162.9)	2(1.6 - 2.4)	22.1(15.7 - 31.2)	4.9(4.7 - 5)	15.2(14.9 - 15.5)	2.6(2.6 - 2.6)	32.6(28.9 - 36.6)
**Canada**	155.6(141.1 - 172.1)	135.5(114.4 - 159.9)	17.7(15 - 20.9)	35.9(29 - 44.4)	11.3(11 - 11.7)	11.8(11.6 - 11.9)	2.5(2.5 - 2.5)	15.3(13.5 - 17.2)
**Cape Verde**	228.5(190.5 - 273.1)	194(158.1 - 237.7)	4.3(3.6 - 5.1)	31.8(22.6 - 44.5)	11.9(11.6 - 12.2)	12(11.8 - 12.3)	2.7(2.7 - 2.7)	59.9(53.8 - 66.7)
**Central African Republic**	97.8(81.4 - 117.1)	107.7(87.3 - 131.8)	3.5(2.9 - 4.1)	119.7(85.5 - 153.7)	9.5(9.3 - 9.8)	9.8(9.6 - 10.1)	2.7(2.7 - 2.7)	24(21.3 - 27)
**Chad**	43.3(35.9 - 51.8)	39.9(32.4 - 49.1)	2.7(2.2 - 3.2)	53.6(37.7 - 75.4)	12(11.7 - 12.4)	10.3(10.1 - 10.5)	2.6(2.6 - 2.6)	15.4(13.7 - 17.4)
**Chile**	113.4(97.8 - 131.6)	140(115.8 - 169.8)	43.9(36.6 - 51.3)	18.2(14.1 - 23.5)	11.6(10.8 - 12.5)	11(10.5 - 11.6)	3.2(3.2 - 3.2)	20.9(18.9 - 23.2)
**China**	106.6(93.8 - 119.1)	306.6(264.7 - 356.2)	4.2(3.1 - 5.5)	91.7(73.9 - 109)	9.6(8.8 - 10.5)	8.3(7.3 - 9.3)	2.9(2.9 - 2.9)	21.1(17.7 - 25.2)
**Colombia**	105.2(90.2 - 123)	99.2(81.9 - 119.4)	63.5(52.9 - 74.1)	102.3(79.9 - 130.8)	9.5(8.7 - 10.3)	12.6(12 - 13.2)	2.7(2.6 - 2.7)	17.6(16 - 19.4)
**Comoros**	86.2(71.9 - 103)	101.7(82.3 - 125.4)	3.7(3 - 4.4)	40.2(28.5 - 56.6)	9.6(9.4 - 9.9)	11.4(11.1 - 11.6)	2.6(2.5 - 2.6)	21.6(19.3 - 24.3)
**Congo**	91.7(76.4 - 109.8)	149.7(121.1 - 183.4)	19.1(15.7 - 22.5)	37.3(26.3 - 52.7)	10(9.7 - 10.3)	14.2(13.9 - 14.5)	2.5(2.5 - 2.5)	27.2(24.1 - 30.7)
**Costa Rica**	135.2(116.4 - 156.8)	167.5(138.6 - 202)	35.9(30.4 - 41.6)	71.5(55.3 - 91.5)	11.7(10.8 - 12.6)	10.3(9.8 - 10.9)	2.6(2.6 - 2.7)	22.4(20.3 - 24.7)
**Cote d'Ivoire**	61.1(51 - 72.9)	98.1(80 - 119.5)	3.4(2.8 - 4)	22(15.5 - 31.1)	10.4(10 - 10.6)	13.5(13.3 - 13.8)	2.5(2.5 - 2.5)	21(18.6 - 23.7)
**Croatia**	59.5(51.9 - 68)	450.3(379.2 - 532.4)	93.9(78.2 - 111.7)	233.8(192.2 - 286.3)	13.2(12.8 - 13.7)	12.2(11.5 - 12.9)	2.6(2.5 - 2.6)	33.5(30.3 - 36.9)
**Cuba**	131.3(112.9 - 152.5)	111(92 - 133.8)	32.5(27.3 - 37.7)	140.4(110 - 179.5)	7.2(6.6 - 7.9)	14.2(13.6 - 14.8)	2.6(2.5 - 2.6)	6.5(5.9 - 7.2)
**Cyprus**	70.2(63.8 - 77.5)	123.9(104.7 - 146)	7.6(6.5 - 9)	58.6(46.9 - 72.6)	12.7(12.3 - 13)	13.5(13.3 - 13.7)	2.8(2.7 - 2.8)	11.2(9.8 - 12.6)
**Czech Republic**	76.5(66.4 - 87.6)	104.1(82.2 - 131.8)	30.7(25.6 - 36.6)	53.7(41.8 - 68.4)	13.8(13.4 - 14.3)	10.5(9.9 - 11.1)	2.6(2.5 - 2.6)	16(14.5 - 17.7)
**Democratic Republic of the Congo**	98.5(81.8 - 118.2)	229.8(185.9 - 281.1)	19.9(16.3 - 23.3)	19.4(13.8 - 27.1)	17.4(17 - 17.8)	16.6(16.3 - 16.9)	2.6(2.6 - 2.6)	23.0(20.3 – 26.0)
**Denmark**	104.9(95.1 - 116)	126.9(107.3 - 150.1)	25.5(21.6 - 29.9)	42(34 - 52)	14.1(13.8 - 14.5)	14.1(14 - 14.3)	2.6(2.6 - 2.6)	22.3(19.7 - 25.2)
**Djibouti**	137.9(115.6 - 164.8)	103.3(84.3 - 126.7)	13.9(11.4 - 16.4)	32.9(23.3 - 46.1)	10.7(10.4 - 11)	11(10.8 - 11.2)	2.6(2.6 - 2.6)	42.4(37.8 - 47.8)
**Dominica**	108.5(93.5 - 125.7)	104.8(86.7 - 125.7)	8.7(7.2 - 10.1)	22.4(17.5 - 28.7)	11.5(10.7 - 12.4)	12.6(12 - 13.2)	2.5(2.4 - 2.5)	14.9(13.5 - 16.5)
**Dominican Republic**	149.9(128.8 - 173.9)	97.2(80.1 - 117.5)	26.6(22.2 - 30.9)	49.4(38.3 - 63.6)	11.4(10.6 - 12.3)	10.2(9.7 - 10.7)	2.7(2.7 - 2.7)	17.5(15.9 - 19.3)
**Ecuador**	26.9(23.1 - 31.2)	112.8(93.2 - 135.7)	3.3(2.7 - 3.8)	51.8(40.4 - 66.2)	13.9(13.1 - 14.9)	8.6(8.1 - 9)	2.6(2.6 - 2.7)	19.7(17.8 - 21.7)
**Egypt**	103.1(83.5 - 126.9)	174.6(133.6 - 226.7)	4.6(3.8 - 5.5)	29.7(22.7 - 38.8)	7.9(7.7 - 8.1)	18(17.1 - 18.8)	2.6(2.6 - 2.6)	39.4(34.1 - 45.5)
**El Salvador**	129(111.6 - 149.8)	131.4(109.2 - 159.1)	37.4(31.7 - 43.2)	46(35.8 - 58.7)	8.2(7.6 - 9)	10.8(10.3 - 11.3)	2.6(2.6 - 2.6)	11.2(10.2 - 12.4)
**Equatorial Guinea**	89.1(74.4 - 106.3)	150.3(121.8 - 184.7)	3(2.4 - 3.5)	42.7(30.3 - 60.5)	10.7(10.4 - 10.9)	11.6(11.3 - 11.8)	2.7(2.6 - 2.7)	29.1(25.8 - 32.9)
**Eritrea**	56.3(47 - 67.1)	71.8(58.6 - 88)	3.3(2.7 - 3.9)	36.7(26 - 52)	9.8(9.5 - 10.1)	11.4(11.2 - 11.7)	2.6(2.6 - 2.6)	29(25.8 - 32.6)
**Estonia**	109.7(95.4 - 125.7)	284.4(223.8 - 359.7)	67.3(56.2 - 80.4)	61.9(48.2 - 78.7)	13.3(12.8 - 13.8)	12.1(11.4 - 12.7)	2.5(2.5 - 2.5)	16.5(14.9 - 18.2)
**Ethiopia**	36.7(30.6 - 43.6)	93.8(76.3 - 115.3)	15.3(12.6 - 18.1)	17.1(12.1 - 24)	7(6.8 - 7.2)	19.7(19.3 - 20)	2.4(2.4 - 2.5)	16.8(14.9 - 18.9)

**Federated States of Micronesia**	173.8(153.3 - 194)	147.0(127 - 169.7)	18.6(14.1 - 24.4)	26.5(21.5 - 31.4)	13.4(12.4 - 14.5)	7.9(7.0 - 8.8)	2.6(2.6 - 2.6)	9.3(7.9 - 11.1)
**Fiji**	130.7(115.5 - 145.9)	224.4(193.2 - 260.8)	8.1(6.1 - 10.7)	17.2(13.9 - 20.4)	17.1(15.9 - 18.3)	10.2(9.1 - 11.4)	2.5(2.5 - 2.5)	11.6(9.7 - 13.8)
**Finland**	173.8(157.4 - 191.5)	129.3(109.8 - 152.5)	44.3(37.8 - 52.2)	48.1(38.8 - 59.4)	14.2(13.8 - 14.6)	11.3(11.1 - 11.4)	2.5(2.5 - 2.5)	20.1(17.8 - 22.8)
**France**	159(143.8 - 175.3)	145.9(123.8 - 171.2)	22(18.7 - 25.8)	33.4(27 - 41.6)	19(18.6 - 19.4)	15.3(15.2 - 15.5)	2.7(2.7 - 2.7)	13.5(12 - 15.3)
**Gabon**	70.7(59 - 84.6)	129.7(105.6 - 159.9)	4.9(4 - 5.8)	56.8(40.1 - 80)	11.8(11.4 - 12.1)	13.1(12.8 - 13.4)	2.7(2.7 - 2.7)	48.5(43.3 - 54.3)
**Georgia**	73.4(63.6 - 84.1)	87.0(69.1 - 108.8)	55.4(47.1 - 65.3)	12.8(10 - 16.2)	9.2(8.8 - 9.5)	15.8(15 - 16.5)	2.5(2.5 - 2.6)	18.3(16.6 - 20.2)
**Germany**	157.7(142.7 - 174.1)	193.3(163.4 - 228.2)	49.5(41.9 - 58.4)	37.5(30.4 - 46.4)	15.4(15 - 15.8)	12.4(12.2 - 12.6)	2.7(2.6 - 2.7)	24.2(21.3 - 27.3)
**Ghana**	87.4(73.1 - 104)	143.3(116.5 - 176.3)	1.8(1.5 - 2.2)	24.6(17.4 - 34.3)	7.4(7.2 - 7.6)	7.8(7.6 - 8)	2.6(2.6 - 2.6)	28.3(25.2 - 31.9)
**Greece**	86.8(78.4 - 95.8)	91.2(77.2 - 107.7)	4.7(4.0 - 5.5)	64.8(52.5 - 80.2)	15.1(14.7 - 15.5)	12.6(12.4 - 12.7)	2.8(2.8 - 2.8)	29.5(26.1 - 33.4)
**Grenada**	126.4(108.7 - 146.9)	257.6(211.8 - 311.5)	7(5.8 - 8.2)	22.5(17.5 - 28.8)	12.2(11.4 - 13.1)	7.1(6.8 - 7.6)	2.6(2.6 - 2.7)	19.5(17.6 - 21.6)
**Guatemala**	81.3(70 - 94.1)	92(75.9 - 111.1)	31.4(26.3 - 36.5)	59.3(46 - 76.1)	5.4(4.9 - 6)	8.4(7.9 - 8.9)	2.7(2.6 - 2.7)	25.5(23.1 - 28.2)
**Guinea**	67(55.8 - 80)	143.1(116.4 - 175.6)	3.1(2.5 - 3.7)	34.3(24.1 - 48.5)	11.7(11.4 - 12)	9.9(9.7 - 10.1)	2.6(2.6 - 2.6)	11.4(10.2 - 12.8)
**Guinea-Bissau**	22.2(18.6 - 26.5)	21.5(17.5 - 26.4)	10.9(8.9 - 12.9)	22.7(16.1 - 31.9)	9.9(9.6 - 10.2)	12.1(11.8 - 12.3)	2.6(2.5 - 2.6)	20.6(18.4 - 23.1)
**Guyana**	213.4(183.3 - 247.9)	149.4(123.7 - 179.9)	59.1(51.3 - 66.7)	43.9(34.2 - 56.5)	10(9.2 - 10.8)	15.3(14.7 - 16)	2.4(2.3 - 2.4)	8.4(7.6 - 9.3)
**Haiti**	71.8(61.9 - 83.7)	82.3(68.3 – 99.0)	11.8(9.8 - 13.8)	16.4(12.8 - 20.9)	7.1(6.5 - 7.8)	15.6(14.8 - 16.3)	2.6(2.6 - 2.6)	8.0(7.3 - 8.9)
**Honduras**	91.2(78.6 - 105.1)	30.7(25.3 - 37.1)	46.7(39.9 - 53.5)	29.5(22.9 - 37.6)	10.1(9.4 - 10.9)	10.4(9.9 - 10.9)	2.5(2.5 - 2.6)	17.3(15.6 - 19.2)
**Hungary**	60.2(52.3 - 69.2)	59.2(46.9 - 74.4)	10(8.3 - 11.9)	19(14.9 - 24.1)	13.7(13.2 - 14.2)	10(9.4 - 10.6)	2.9(2.8 - 3)	9.4(8.5 - 10.5)
**Iceland**	92.2(83.4 - 101.8)	74.1(62.5 - 88.1)	19.8(16.7 - 23.4)	50.4(40.8 - 62.4)	16.4(16 - 16.9)	15.4(15.3 - 15.6)	2.5(2.5 - 2.5)	14.9(13.2 - 16.8)
**India**	32.2(27.6 - 37.5)	161.9(126.1 - 208.4)	2.1(1.8 - 2.3)	3.8(3 - 4.7)	6.6(6 - 7.2)	6.8(6.5 - 7.2)	2.8(2.8 - 2.8)	25.4(22.7 - 28.5)
**Indonesia**	79.9(70.2 - 89.2)	169.2(146.4 - 195.6)	25.9(19.8 - 33.5)	21.5(17.4 - 25.5)	16.7(15.5 - 17.8)	9.9(8.8 - 11)	2.6(2.6 - 2.6)	11.8(9.9 - 14)
**Iran**	189.5(154.5 - 231.8)	262(201.5 - 338)	5.2(4.2 - 6.1)	15.3(11.7 - 20)	10.2(9.9 - 10.4)	9.2(8.6 - 9.8)	2.8(2.8 - 2.9)	24.8(21.5 - 28.7)
**Iraq**	123.7(101.5 - 150.8)	144.2(110.2 - 189.2)	26.1(21.4 - 30.8)	48.2(36.5 - 62.7)	9.7(9.4 - 9.9)	14.9(14.1 - 15.7)	2.7(2.7 - 2.8)	15(12.9 - 17.4)
**Ireland**	93.5(84.9 - 103.2)	124.8(105.7 - 147.7)	28.2(23.9 - 33.1)	30.1(24.2 - 37.6)	16.2(15.8 - 16.6)	13.3(13.1 - 13.5)	2.6(2.6 - 2.6)	12.4(11 - 14.1)
**Israel**	149.3(122.1 - 183)	94.1(72 - 123.1)	72.8(59.3 - 86.3)	118.6(90.8 - 155.2)	8.7(8.5 - 8.9)	12.3(11.6 - 13)	2.7(2.7 - 2.7)	10.1(8.7 - 11.7)
**Italy**	209.4(189.6 - 232)	144.4(122 - 170.1)	16.2(13.8 - 19)	43.1(34.7 - 53.6)	12.7(12.4 - 13.1)	17.1(16.9 - 17.3)	2.5(2.5 - 2.5)	16.3(14.4 - 18.5)
**Jamaica**	184.8(159.6 - 214)	151.8(125.3 - 184.1)	11.4(9.5 - 13.4)	11.9(9.2 - 15.2)	6.3(5.7 - 6.9)	9.5(9.1 - 10)	2.8(2.8 - 2.9)	14.3(13 - 15.8)
**Japan**	125.8(111.4 - 140.3)	279.8(241.2 - 321.6)	17.4(13 - 22.9)	83.3(67.3 - 99.2)	8.9(8 - 9.7)	11.9(10.5 - 13.2)	2.7(2.6 - 2.7)	12.1(10.2 - 14.5)
**Jordan**	194.8(159.7 - 238.6)	131.3(100 - 171.4)	26.6(21.7 - 31.5)	52(39.6 - 68.1)	8.4(8.2 - 8.7)	5.7(5.3 - 6.1)	2.7(2.6 - 2.7)	54.7(47.2 - 63.6)
**Kazakhstan**	62.8(54.7 - 72.2)	169.9(134.3 - 214.1)	35.3(29.8 - 41.7)	87.8(69.2 - 111.3)	12.6(12.1 - 13.1)	13.3(12.6 - 14.1)	2.6(2.6 - 2.6)	26.9(24.3 - 29.8)
**Kenya**	104.6(87 - 124.7)	257(209.6 - 315)	0.6(0.5 - 0.7)	25.3(18 - 35.3)	10.6(10.3 - 10.9)	13.6(13.3 - 13.8)	2.5(2.5 - 2.5)	24.6(21.9 - 27.6)

**Kiribati**	116.8(103 - 130.6)	95.2(82 - 110.1)	19.7(14.9 - 25.9)	28.7(23.2 - 34.1)	12.6(11.6 - 13.7)	7.6(6.7 - 8.5)	2.6(2.6 - 2.6)	10(8.4 - 11.8)
**Kuwait**	62.2(50.8 - 75.8)	119.6(91.4 - 157)	23.5(19.1 - 27.9)	27(20.8 - 35.2)	11.3(11 - 11.6)	9.5(9 - 10.1)	2.6(2.6 - 2.6)	26.9(23.2 - 31.1)
**Kyrgyzstan**	81(70.8 - 92.4)	43.2(34.3 - 54.3)	19(15.9 - 22.5)	36.4(28.6 - 45.7)	10.5(10.1 - 10.9)	20.5(19.6 - 21.5)	2.5(2.4 - 2.5)	7.9(7.1 - 8.8)
**Laos**	143.6(126.5 - 160.6)	151.2(130.6 - 174.5)	12.3(9.2 - 16.3)	31.7(25.6 - 37.5)	9.9(9.0 - 10.8)	6.3(5.6 – 7.0)	2.5(2.5 - 2.5)	10.8(9.1 - 12.8)
**Latvia**	59.5(51.9 - 68.2)	222.2(176.3 - 278.2)	46.7(39.3 - 55.5)	174.5(137.8 - 221.1)	14.6(14.1 - 15.2)	11.6(11 - 12.3)	2.6(2.5 - 2.6)	30.6(27.7 - 33.8)
**Lebanon**	237.4(194.2 - 291.2)	367.2(280 - 482.3)	6.2(5.1 - 7.3)	18.5(14.2 - 24.2)	11.9(11.6 - 12.1)	19.9(19 - 20.8)	3.1(3.1 - 3.1)	25.3(21.8 - 29.4)
**Lesotho**	67.1(56 - 80.6)	197.4(160.3 - 241.6)	3.1(2.5 - 3.7)	41.6(29.4 - 58.9)	12.1(11.8 - 12.5)	10.3(10.1 - 10.6)	2.6(2.6 - 2.6)	40.5(36 - 45.6)
**Liberia**	48.4(40.5 - 57.9)	122(99.2 - 150.8)	32.5(27.3 - 38)	37.9(26.7 - 52.8)	12.4(12.1 - 12.7)	10.5(10.2 - 10.7)	2.7(2.6 - 2.7)	21.1(18.7 - 23.7)
**Libya**	58.6(47.6 - 72.5)	126(96 - 166.3)	9.9(8 - 11.7)	33.1(25.2 - 43)	13.1(12.7 - 13.3)	11.9(11.3 - 12.6)	2.6(2.5 - 2.6)	27.7(23.8 - 32.1)
**Lithuania**	70.8(61.5 - 81.1)	124.6(98.5 - 157.2)	34.1(28.7 - 40.3)	122.2(96.2 - 154.5)	13.6(13.2 - 14.2)	13.7(13 - 14.4)	2.5(2.5 - 2.5)	16.3(14.8 - 18)
**Luxembourg**	100(90.4 - 110.3)	97.8(83.2 - 115.6)	76.1(64.2 - 90.3)	79.6(64.3 - 98.7)	12.7(12.4 - 13.1)	13(12.9 - 13.2)	2.6(2.6 - 2.6)	36.5(32.2 - 41.2)
**Macedonia**	93.8(81.5 - 107.9)	148.0(117.3 - 186.3)	50.6(43.1 - 59.5)	42.1(32.8 - 53.4)	11.4(10.9 - 11.8)	12.1(11.5 - 12.8)	2.6(2.6 - 2.7)	32.6(29.6 - 35.9)
**Madagascar**	87(72.6 - 103.2)	165.3(134.7 - 201.7)	4.1(3.4 - 4.9)	40.6(29.1 - 56.2)	6.9(6.7 - 7.2)	4.6(4.5 - 4.7)	2.4(2.4 - 2.4)	5.6(4.9 - 6.3)
**Malawi**	116(96.7 - 138.4)	188.1(154.1 - 230.5)	4(3.3 - 4.8)	23.7(16.9 - 33.3)	6(5.8 - 6.2)	18.3(18 - 18.6)	2.5(2.5 - 2.6)	18(16 - 20.2)
**Malaysia**	128.4(113.8 - 143.1)	169.9(146.6 - 196.5)	13.2(9.9 - 17.3)	16.1(13 - 19.2)	10.6(9.6 - 11.5)	9.9(8.8 - 11.1)	2.5(2.5 - 2.5)	19.3(16.4 - 22.9)
**Maldives**	223.2(191.1 - 260.3)	148.7(116.6 - 190.3)	12(10.7 - 13.6)	27.5(22.1 - 34.4)	7.4(6.8 - 8)	7.5(7.1 - 7.9)	2.5(2.5 - 2.5)	61.2(55.6 - 67.4)
**Mali**	68.8(57.3 - 82.1)	95.3(77.5 - 116.9)	6.5(5.3 - 7.8)	37.1(26.1 - 52)	9.2(9 - 9.5)	13.5(13.3 - 13.8)	2.6(2.6 - 2.6)	53.5(47.9 - 59.8)
**Malta**	154(139.2 - 170.2)	91.6(77.3 - 107.7)	30(25.5 - 35.3)	50.8(41.1 - 62.7)	14.3(13.9 - 14.6)	11.8(11.7 - 12)	2.6(2.6 - 2.6)	22.9(20.2 - 25.9)
**Marshall Islands**	131.1(115.5 - 146.9)	192.8(166.5 - 222.7)	8.3(6.2 - 11.1)	26.7(21.7 - 31.8)	13.5(12.5 - 14.6)	8.1(7.1 - 9)	2.6(2.6 - 2.6)	12.8(10.7 - 15.2)
**Mauritania**	34.5(28.8 - 41.1)	233.7(188.6 - 285.5)	31.2(25.9 - 36.5)	29.4(20.7 - 41)	12.4(12.1 - 12.7)	12.4(12.2 - 12.7)	2.6(2.6 - 2.6)	52.3(46.7 - 58.4)
**Mauritius**	74.5(62.3 - 89.2)	179.4(146.6 - 218.2)	28(22.8 - 33.2)	62.8(44.1 - 88)	10.1(9.8 - 10.3)	11(10.7 - 11.2)	2.8(2.8 - 2.8)	52.5(46.9 - 58.6)
**Mexico**	155.9(133.6 - 180.9)	206.5(171.6 - 248.7)	14.6(12.1 - 17.2)	43.7(34.2 - 56)	11.4(10.6 - 12.2)	10.5(9.9 - 11)	2.3(2.3 - 2.4)	9.5(8.6 - 10.5)
**Moldova**	98.3(85.4 - 113.2)	86.2(68.3 - 109.2)	55.1(47 - 64.2)	75.5(59.3 - 96.1)	11(10.6 - 11.4)	17.4(16.5 - 18.4)	2.6(2.5 - 2.6)	11.7(10.5 - 12.9)
**Mongolia**	52.9(46 - 60.5)	188.8(150.4 - 237.9)	119.6(111.2 - 128.4)	68.3(54 - 86.5)	13.4(12.9 - 13.8)	14.9(14.2 - 15.7)	2.5(2.5 - 2.6)	21.3(19.3 - 23.6)
**Montenegro**	252.5(220.2 - 289)	132.7(104.7 - 167.5)	49.8(41.8 - 59)	176.7(139.3 - 224.2)	12.5(12 - 12.9)	8.4(8 - 8.9)	2.5(2.5 - 2.6)	58(53.1 - 63.4)
**Morocco**	112.7(91.7 - 138.3)	191.2(145.9 - 250.8)	16.6(13.7 - 19.7)	23.8(18.2 - 31.2)	8(7.8 - 8.3)	13.1(12.4 - 13.8)	2.5(2.5 - 2.5)	42.9(37 - 49.6)
**Mozambique**	108.6(91 - 129.8)	149.8(121.7 - 185.5)	3.1(2.5 - 3.6)	33.3(23.4 - 47.5)	7.8(7.6 - 8.1)	4.9(4.8 - 5)	2.5(2.5 - 2.6)	33.9(30.1 - 38.1)
**Myanmar**	92.3(81.5 - 103.2)	179.1(154.6 - 207.8)	26.6(20.5 - 34)	79.9(64.4 - 95.3)	12.4(11.4 - 13.4)	7.2(6.3 - 8)	2.6(2.6 - 2.6)	18.5(15.5 - 22)
**Namibia**	231.2(193.1 - 275.8)	146.5(119.3 - 180)	5.4(4.5 - 6.4)	37.7(26.6 - 53.4)	8.8(8.5 - 9)	13.6(13.4 - 13.9)	2.6(2.6 - 2.6)	38(33.7 - 42.7)
**Nepal**	38.4(32.7 - 45.2)	181.5(141.9 - 232)	0.6(0.5 - 0.6)	11.1(8.8 - 13.7)	3(2.7 - 3.4)	4.1(3.9 - 4.3)	2.3(2.3 - 2.3)	49.7(44.5 - 55.3)

**Netherlands**	119.5(108.4 - 132)	114.4(96.6 - 135.2)	18.6(15.7 - 22)	38.3(30.8 - 47.7)	13.8(13.4 - 14.2)	11.9(11.8 - 12.1)	2.7(2.7 - 2.7)	12.3(10.9 - 13.9)
**New Zealand**	186.8(168.6 - 207.1)	138.3(116.7 - 164)	30.1(25.4 - 35.3)	49.9(40.3 - 61.9)	17.6(17.2 - 18)	14.9(14.7 - 15.1)	2.7(2.7 - 2.7)	19.4(17.1 - 22)
**Nicaragua**	162.1(139.2 - 188.2)	280.3(231.8 - 338.8)	38.6(32.9 - 44.3)	35.7(27.8 - 45.6)	8.3(7.6 - 9)	11.8(11.2 - 12.4)	2.6(2.5 - 2.6)	10.5(9.5 - 11.5)
**Niger**	67(56.1 - 80.2)	111.4(91 - 135.6)	2.4(2 - 2.9)	6.5(4.6 - 9.2)	9(8.8 - 9.3)	13.1(12.8 - 13.4)	2.6(2.6 - 2.6)	29.3(26 - 33.1)
**Nigeria**	36.2(30.1 - 43.3)	101.1(82.8 - 123.3)	4.2(3.4 - 4.9)	36.7(25.8 - 52.1)	9.9(9.6 - 10.2)	12.1(11.9 - 12.3)	2.6(2.5 - 2.6)	17.6(15.6 - 19.9)
**Norway**	97.6(88.2 - 107.7)	140.1(118.5 - 165.9)	21.9(18.6 - 25.8)	72.4(58.4 - 89.7)	14(13.6 - 14.3)	12.7(12.5 - 12.9)	2.6(2.6 - 2.6)	17(15 - 19.2)
**Oman**	137.8(111.9 - 169.2)	175.1(133.5 - 230.6)	20.0(16.1 - 23.7)	39.6(30.2 – 52.0)	10.4(10.2 - 10.7)	12.2(11.5 - 12.9)	2.7(2.7 - 2.8)	29.7(25.5 - 34.5)
**Pakistan**	34(28.9 - 39.9)	87.4(68.3 - 111.3)	4.5(4.0 - 5.1)	25.9(20.7 - 32.5)	9.6(8.9 - 10.3)	6.0(5.7 - 6.3)	2.6(2.6 - 2.6)	17.1(15.3 - 19.1)
**Palestine**	126.9(104 - 155.6)	173.6(132.9 - 226.3)	12.6(10.3 - 14.9)	35.1(26.9 - 46.2)	12.6(12.3 - 12.9)	6.8(6.4 - 7.2)	2.7(2.7 - 2.7)	71.9(67 - 77.1)
**Panama**	135.5(116.9 - 157.3)	154.1(127.9 - 186)	40.4(34.4 - 46.3)	72.6(56.6 - 92.6)	9.1(8.4 - 9.9)	12.1(11.5 - 12.8)	2.7(2.6 - 2.7)	15.6(14.1 - 17.3)
**Papua New Guinea**	4.6(4 - 5.1)	88.3(76.3 - 102.3)	3.6(2.7 - 4.7)	157.4(132.4 - 182.7)	13.2(12.1 - 14.2)	7.5(6.6 - 8.4)	2.6(2.5 - 2.6)	3.7(3.2 - 4.4)
**Paraguay**	97.5(84.1 - 113)	71.3(58.9 - 85.9)	22.7(19.1 - 26.3)	127.3(99.5 - 161.8)	10(9.2 - 10.8)	9.4(9 - 9.9)	2.8(2.7 - 2.8)	10.7(9.8 - 11.9)
**Peru**	242.1(208.5 - 280.6)	101.2(83.7 - 122.2)	24.7(20.5 - 28.7)	113.9(89.1 - 145.6)	7.8(7.1 - 8.5)	15.2(14.6 - 15.9)	2.6(2.5 - 2.6)	10.5(9.5 - 11.6)
**Philippines**	96(84.5 - 107.3)	112.6(97.6 - 129.9)	30.8(23.1 - 40.9)	20.4(16.6 - 24.3)	22.5(21.1 - 23.8)	11.3(10.1 - 12.5)	2.4(2.4 - 2.4)	8.5(7.1 - 10.1)
**Poland**	231.7(201.3 - 265.9)	158.6(125.6 - 199.6)	43.4(36.4 - 51.6)	48.6(38.1 - 62)	13.3(12.8 - 13.8)	15.5(14.8 - 16.3)	2.5(2.5 - 2.6)	12.2(11.1 - 13.6)
**Portugal**	445.6(410.3 - 483.4)	153.5(129.5 - 181.9)	13.7(11.7 - 16.2)	50.3(40.7 - 62.6)	9.9(9.6 - 10.2)	11.6(11.5 - 11.8)	2.7(2.7 - 2.7)	16(14.2 - 18.2)
**Qatar**	138.1(112.8 - 169.2)	182.2(140.3 - 237.4)	14.8(12 - 17.6)	32.5(24.9 - 42)	10.5(10.3 - 10.8)	12.3(11.6 - 12.9)	2.7(2.7 - 2.7)	17.2(14.8 - 19.9)
**Romania**	202.0(176.3 - 232.3)	338.4(270.1 - 422.6)	38.6(32.5 - 46.1)	60.4(47.5 - 76.5)	11.1(10.7 - 11.5)	14.4(13.6 - 15.1)	2.2(2.2 - 2.2)	19.9(18 - 22)
**Russia**	92.2(80.7 - 105.1)	173.5(137.3 - 217.9)	40.6(34 - 48.3)	163(128.7 - 206.1)	11.4(11 - 11.9)	14.1(13.4 - 14.9)	2.5(2.5 - 2.6)	20.2(18.3 - 22.4)
**Rwanda**	125.4(104.6 - 149.6)	297.1(241.6 - 363.4)	1.1(0.9 - 1.3)	8.2(5.8 - 11.4)	6.8(6.5 - 7)	23.6(23.1 - 24)	2.4(2.4 - 2.4)	11.4(10.1 - 12.7)
**Saint Lucia**	78.4(67.3 - 91.2)	91.8(76.1 - 110.8)	6.3(5.3 - 7.4)	36.3(28.2 - 46.1)	10.7(9.9 - 11.5)	7.6(7.2 – 8.0)	2.4(2.4 - 2.5)	14.5(13.1 - 16)
**Saint Vincent and the Grenadines**	149.1(128.4 - 173.4)	105.3(86.9 - 127)	13.6(11.3 - 16)	54.1(42.4 - 69.2)	10.1(9.3 - 10.9)	9(8.6 - 9.5)	2.6(2.5 - 2.6)	19.8(17.9 - 21.9)
**Samoa**	169.2(149.3 - 188.9)	372.2(321.3 - 429.9)	14(10.6 - 18.6)	23.7(19.2 - 28.2)	27.1(25.7 - 28.5)	7.5(6.6 - 8.3)	2.6(2.6 - 2.6)	23.3(19.6 - 27.7)
**Sao Tome and Principe**	126.8(105.8 - 151.9)	36.7(29.9 - 45)	13.2(10.9 - 15.5)	11.6(8.1 - 16.3)	12.5(12.2 - 12.9)	10.5(10.3 - 10.8)	2.7(2.7 - 2.7)	32.9(29.3 - 37)
**Saudi Arabia**	163.5(133.8 - 199.8)	163.5(125 - 214.2)	50.6(43 - 58.1)	32(24.2 - 42.4)	11.6(11.3 - 11.8)	12.8(12 - 13.5)	2.7(2.7 - 2.7)	33.1(28.5 - 38.4)
**Senegal**	53.5(44.5 - 64.1)	80.2(65.3 - 98)	13.5(11.1 - 16)	13.7(9.7 - 19.3)	9.6(9.3 - 9.9)	13.1(12.8 - 13.4)	2.7(2.7 - 2.8)	44.2(39.1 - 49.9)
**Serbia**	155.1(135.3 - 178.3)	436.9(361.4 - 527.3)	24.2(20.2 - 28.8)	64.9(51.2 - 82)	14.3(13.8 - 14.8)	10.1(9.5 - 10.7)	2.5(2.5 - 2.6)	34.1(30.8 - 37.7)
**Seychelles**	106.5(89.1 - 127.6)	216.7(176.9 - 266.2)	10.3(8.4 - 12.2)	25.6(18.2 - 36.3)	9.9(9.6 - 10.2)	11.5(11.3 - 11.7)	2.6(2.6 - 2.6)	22.4(19.8 - 25.3)
**Sierra Leone**	93.1(77.6 - 111.3)	126.1(102.5 - 155)	50.6(43.2 - 58)	17.6(12.6 - 24.6)	12.2(11.9 - 12.6)	10.4(10.2 - 10.7)	2.6(2.6 - 2.7)	21.6(19.2 - 24.3)
**Singapore**	159.3(140.7 - 177.7)	151.9(130.7 - 176.2)	14.3(10.7 - 19.2)	39(31.5 - 46.4)	11.6(10.6 - 12.7)	7.5(6.6 - 8.5)	2.6(2.6 - 2.7)	19.5(16.3 - 23.1)

**Slovakia**	81.6(70.9 - 93.6)	97.7(77.6 - 123.1)	42.1(35.3 - 50.1)	27.7(21.7 - 35.4)	13(12.5 - 13.5)	10.8(10.2 - 11.4)	2.5(2.5 - 2.6)	26.4(23.9 - 29.2)
**Slovenia**	79.6(69.5 - 91)	110.4(87.2 - 138.7)	27.8(23.4 - 33.1)	48(37.9 - 60.5)	13.2(12.7 - 13.7)	10.7(10.1 - 11.3)	2.6(2.5 - 2.6)	19.7(17.8 - 21.8)
**Solomon Islands**	72.2(63.8 - 80.5)	193.4(166.6 - 224)	15.4(11.6 - 20.1)	38.9(31.5 - 46.4)	12.5(11.5 - 13.6)	7.5(6.6 - 8.4)	2.6(2.6 - 2.6)	4(3.3 - 4.7)
**South Africa**	32.2(26.8 - 38.6)	134.8(110 - 165.6)	13.6(11 - 16.2)	176.1(133.9 - 230.4)	11.3(11 - 11.6)	11.8(11.5 - 12)	2.9(2.8 - 2.9)	18.8(16.6 - 21.1)
**South Korea**	117.8(104.1 - 131.7)	132.8(114.5 - 153.9)	1.9(1.5 - 2.6)	34.8(28.3 - 41.4)	6.6(5.9 - 7.3)	6.4(5.5 - 7.2)	2.5(2.4 - 2.5)	11(9.2 - 13.1)
**South Sudan**	64.9(54.1 - 77.6)	148.3(120.5 - 181.6)	3.4(2.8 - 4.1)	31.8(22.6 - 44.5)	9.6(9.3 - 9.8)	11.3(11 - 11.5)	2.5(2.5 - 2.6)	31.6(28.2 - 35.4)
**Spain**	72.3(65.4 - 79.9)	105.1(88.7 - 124.3)	25.9(22 - 30.6)	44.4(35.9 - 55.1)	13.2(12.8 - 13.6)	11.1(11 - 11.3)	2.8(2.8 - 2.8)	16.3(14.4 - 18.4)
**Sri Lanka**	78.3(66.9 - 91.6)	177.8(138.4 - 226.6)	5.5(4.9 - 6.2)	37.2(29.5 - 46.6)	10(9.3 - 10.8)	11.4(11 - 11.9)	2.3(2.3 - 2.4)	11(9.8 - 12.3)
**Sudan**	82.8(69 - 99.3)	136.6(111.1 - 167.9)	5.2(4.2 - 6.1)	26.3(18.6 - 37.2)	11(10.7 - 11.3)	12.9(12.6 - 13.1)	2.6(2.5 - 2.6)	48.5(43.3 - 54.4)
**Suriname**	108.4(93.3 - 125.8)	187.5(154.3 - 225.3)	36.1(30.4 - 41.6)	68.5(53.3 - 87)	7.4(6.8 - 8.1)	11.9(11.4 - 12.5)	2.7(2.7 - 2.7)	11.9(10.7 - 13.1)
**Swaziland**	91.5(76.4 - 109.3)	170.8(139.3 - 209.5)	1.9(1.6 - 2.3)	30.6(21.8 - 43.2)	7.9(7.7 - 8.2)	10.5(10.3 - 10.7)	2.5(2.5 - 2.5)	44.9(40.1 - 50.4)
**Sweden**	136.4(123.5 - 150.6)	158.3(133.4 - 187.2)	26.2(22.2 - 30.9)	55.7(44.8 - 69.1)	14.5(14.1 - 14.9)	24(23.8 - 24.3)	2.5(2.5 - 2.5)	20.5(18.2 - 23.3)
**Switzerland**	158.7(143.8 - 174.8)	117(98.8 - 138.1)	16.5(14.1 - 19.4)	38.5(31.1 - 47.3)	14.2(13.9 - 14.6)	14.2(14 - 14.4)	2.7(2.6 - 2.7)	14.7(13 - 16.7)
**Syria**	117(95.6 - 143.7)	151.2(115.9 - 195.8)	10.4(8.5 - 12.2)	33.1(25.3 - 43.1)	13.9(13.6 - 14.2)	8.7(8.1 - 9.2)	2.8(2.7 - 2.8)	33.1(28.5 - 38.3)
**Taiwan**	161.2(142.1 - 180.6)	283.1(245.2 - 327.2)	17.7(13.2 - 23.5)	57.8(46.8 - 68.8)	10.6(9.7 - 11.6)	8.5(7.5 - 9.6)	2.8(2.8 - 2.8)	22.6(19.1 - 26.8)
**Tajikistan**	101.5(88.4 - 116.4)	64.7(51 - 81.5)	70.3(60.7 - 81.5)	44.9(35.3 - 57.2)	8.5(8.1 - 8.9)	19.9(19 - 20.8)	2.6(2.5 - 2.6)	7.2(6.5 - 7.9)
**Tanzania**	59.4(49.7 - 71)	178.2(144.8 - 217)	2.4(1.9 - 2.8)	48.9(34.7 - 68.3)	11.1(10.8 - 11.5)	9.5(9.3 - 9.7)	2.6(2.6 - 2.6)	15.5(13.7 - 17.4)
**Thailand**	113(99.8 - 126.6)	175.5(151.8 - 202.6)	8.9(6.7 - 11.8)	32.3(26.2 - 38.3)	10.5(9.6 - 11.5)	10.6(9.5 - 11.8)	2.5(2.5 - 2.5)	7.4(6.2 - 8.8)
**The Bahamas**	85.9(73.9 - 99.7)	106(87 - 128.4)	13.9(11.6 - 16.2)	46.2(36.1 - 59.3)	11.7(10.9 - 12.6)	7.3(6.9 - 7.7)	2.6(2.6 - 2.6)	20.2(18.3 - 22.3)
**The Gambia**	31(25.9 - 37.1)	51.2(41.7 - 62.6)	22.4(18.4 - 26.2)	11.8(8.4 - 16.5)	10(9.7 - 10.2)	9.7(9.5 - 10)	2.7(2.7 - 2.7)	19.8(17.6 - 22.3)
**Timor-Leste**	63.9(56.3 - 71.4)	156.5(135.1 - 180.9)	17.5(13.3 - 23.2)	16.9(13.7 - 20.1)	12.5(11.5 - 13.5)	7.4(6.5 - 8.3)	2.6(2.6 - 2.6)	9(7.5 - 10.7)
**Togo**	54.8(45.6 - 65.5)	157.8(127.4 - 193.6)	14.8(12.1 - 17.3)	22(15.5 - 31.3)	12.3(12 - 12.6)	10.4(10.2 - 10.7)	2.6(2.6 - 2.7)	13.8(12.3 - 15.6)
**Tonga**	153.3(135.2 - 170.9)	150.1(129.6 - 173.1)	8.2(6.2 - 10.8)	26.9(22.1 - 31.9)	13.4(12.4 - 14.5)	7.9(6.9 - 8.8)	2.6(2.5 - 2.6)	9.2(7.8 - 11)
**Trinidad and Tobago**	70.7(61.1 - 82.2)	87.1(72 - 105.6)	30.4(25.5 - 35.3)	62(48.2 - 79)	9.8(9.1 - 10.6)	8.4(7.9 - 8.8)	2.6(2.6 - 2.6)	7.8(7.1 - 8.7)
**Tunisia**	72.5(59.3 - 88.7)	219.6(168 - 286.6)	0.7(0.5 - 0.8)	37.1(28.5 - 48.3)	10.2(10 - 10.5)	12.7(11.9 - 13.4)	3(3 - 3)	38.4(33 - 44.6)
**Turkey**	89.1(72.6 - 109.2)	128.6(98.6 - 168.1)	3.3(2.7 - 3.9)	46.4(35.5 - 60.1)	11.3(11 - 11.6)	11.2(10.5 - 11.9)	2.9(2.8 - 2.9)	22.1(19.1 - 25.6)
**Turkmenistan**	73.5(64.1 - 84.4)	118.7(94 - 148.9)	59.2(50.8 - 68.6)	71.6(56.3 - 90.8)	12(11.6 - 12.5)	13.2(12.6 - 14)	2.5(2.5 - 2.6)	12.8(11.6 - 14.2)
**Uganda**	53.9(44.9 - 64.1)	116.5(94.8 - 142.3)	3.1(2.5 - 3.7)	18.2(12.9 - 25.5)	12.1(11.8 - 12.4)	10.3(10.1 - 10.5)	2.6(2.6 - 2.6)	16.3(14.5 - 18.3)
**Ukraine**	95.7(83.2 - 109.7)	175.4(138.8 - 221.3)	39.9(33.9 - 47.3)	32.7(25.7 - 41.4)	10.9(10.5 - 11.3)	14.2(13.4 - 15)	2.6(2.5 - 2.6)	19.8(17.9 - 21.9)
**United Arab Emirates**	122.7(100 - 151.6)	136.8(104 - 177.9)	23.2(18.9 - 27.4)	26.1(20 - 34.1)	8.7(8.4 - 8.9)	8.2(7.6 - 8.8)	2.4(2.4 - 2.4)	12.8(11 - 14.9)

**United Kingdom**	107.8(97.6 - 119.1)	123.1(103.9 - 145.3)	27.3(23.1 - 32.1)	35.4(28.5 - 43.8)	12.8(12.4 - 13.2)	13.9(13.8 - 14.1)	2.6(2.6 - 2.6)	11.2(9.9 - 12.7)
**United States**	96.4(87.3 - 106.6)	131.5(111.4 - 155.4)	18.8(16 - 22)	27.5(22.1 - 34.1)	11.7(11.4 - 12.1)	11.8(11.6 - 11.9)	2.8(2.8 - 2.8)	13.8(12.2 - 15.6)
**Uruguay**	101.2(87 - 117.3)	167.7(138.8 - 201.2)	40.4(34.2 - 46.6)	68.3(53.4 - 87.1)	11.6(10.8 - 12.5)	11.5(11 - 12.1)	2.6(2.6 - 2.6)	33.1(30 - 36.6)
**Uzbekistan**	89.2(77.7 - 102.1)	235.5(186.8 - 296.2)	39(32.9 - 46.4)	92(71.9 - 117.1)	10.5(10.1 - 10.9)	17.6(16.8 - 18.5)	2.6(2.5 - 2.6)	19.6(17.8 - 21.6)
**Vanuatu**	48.8(42.9 - 54.6)	78.6(68 - 91)	4.7(3.5 - 6.3)	37.3(30.2 - 44.6)	12.5(11.5 - 13.5)	7.5(6.6 - 8.4)	2.6(2.6 - 2.6)	17.6(14.8 - 21)
**Venezuela (Bolivarian Republic of)**	112.6(97.2 - 130.3)	93.4(76.9 - 113.6)	25.7(21.5 - 30)	42.2(33 - 54.1)	5.2(4.7 - 5.8)	5.5(5.2 - 5.9)	2.4(2.4 - 2.5)	6.7(6 - 7.4)
**Vietnam**	99.9(88.3 - 111.5)	183.3(158.4 - 211.6)	7(5.2 - 9.2)	44.8(36.4 - 53.2)	5.4(4.8 - 6)	3.8(3.3 - 4.4)	2.2(2.2 - 2.2)	6.6(5.5 - 7.8)
**Yemen**	46.5(37.9 - 56.9)	72.5(55.5 - 94.2)	11.6(9.5 - 13.7)	20.6(15.8 - 26.9)	8.4(8.2 - 8.6)	12.4(11.8 - 13)	2.6(2.6 - 2.7)	18.2(15.7 - 21.2)
**Zambia**	50.9(42.6 - 61)	204.3(167.1 - 250.3)	8.2(6.8 - 9.7)	67.3(47.4 - 94.3)	12.3(12 - 12.7)	10.4(10.2 - 10.6)	2.6(2.6 - 2.7)	20.5(18.3 – 23.0)
**Zimbabwe**	82.9(69.2 - 98.9)	143.0(117.3 - 175.7)	5.2(4.3 - 6.2)	41.6(29.5 - 59)	9.0(8.8 - 9.3)	11.9(11.6 - 12.1)	2.7(2.7 - 2.7)	18.0(16.0 - 20.3)

Supplementary Table 6: National mean (95% UI) intakes of eight nutrients among females in 1990 across 185 countries and regions.

Country/Region	Fruits	Non-starchy vegetables	Total processed meats	Unprocessed red meats	Saturated fat	Monounsaturated fatty acids	Total omega-6 fat	Dietary fiber
**Afghanistan**	77.9(66.4 - 91.4)	159.5(124.3 – 205.0)	2.3(2.1 - 2.6)	32.7(26.1 - 41.1)	7.6(7.0 - 8.2)	4.9(4.6 - 5.2)	2.6(2.6 - 2.6)	62.5(56.4 - 69.3)
**Albania**	19.7(17.2 - 22.6)	68.9(54.7 - 86.5)	24.9(21 - 29.7)	7.0(5.5 - 8.8)	9.2(8.8 - 9.6)	12.4(11.8 - 13)	2.4(2.4 - 2.5)	6.2(5.6 - 6.9)
**Algeria**	75.5(61.4 - 93.1)	132.1(100.9 - 173.1)	8.9(7.2 - 10.6)	23.5(18.0 - 30.7)	12.4(12.1 - 12.7)	20.4(19.3 - 21.5)	2.6(2.6 - 2.6)	16.2(14 - 18.7)
**Angola**	45.4(37.7 - 54.1)	139.3(112.5 - 170.1)	3.2(2.6 - 3.7)	24.6(17.5 - 34)	12.2(11.9 - 12.5)	10.7(10.4 - 10.9)	2.6(2.6 - 2.6)	16.9(15 - 19.1)
**Antigua and Barbuda**	91.2(78.2 - 106.5)	197.3(163.8 - 237.9)	31(26.3 - 35.6)	35.3(27.4 - 45.4)	10.8(10.1 - 11.7)	8.6(8.1 - 9.1)	2.6(2.6 - 2.7)	18.8(17 - 20.7)
**Argentina**	93.4(80.4 - 108.2)	127.4(105.4 - 153.6)	13.4(11 - 15.6)	83(64.7 - 106.7)	10.5(9.7 - 11.3)	11.3(10.8 - 11.9)	2.6(2.5 - 2.6)	8.6(7.7 - 9.5)
**Armenia**	40.2(35.1 - 45.9)	86.7(69.5 - 108.4)	24(20.2 - 28.5)	22.1(17.2 - 28.2)	12.5(12.1 - 13)	21.1(20.1 - 22.3)	2.5(2.5 - 2.6)	6.7(6.1 - 7.4)
**Australia**	162.9(147.4 - 180.1)	107.2(90.3 - 126.6)	14.1(11.9 - 16.5)	95.1(76.2 - 118.6)	13.1(12.8 - 13.5)	11.1(10.9 - 11.2)	2.6(2.6 - 2.6)	17.4(15.4 - 19.7)
**Austria**	119.3(107.9 - 132.2)	65.4(55.3 - 77.3)	53.1(45.2 - 62.6)	128(103.1 - 157.8)	16.9(16.4 - 17.3)	13.1(12.9 - 13.3)	2.8(2.8 - 2.8)	18.1(16 - 20.5)
**Azerbaijan**	113.6(99 - 130.4)	55.8(44.3 - 70.3)	84(74.1 - 95.7)	35.4(27.8 - 45)	12.5(12 - 12.9)	32.6(31.6 - 33.7)	2.5(2.5 - 2.6)	6.1(5.5 - 6.8)
**Bahrain**	114.7(92.9 - 140.6)	178.5(136.3 - 233.6)	14.2(11.5 - 16.8)	35.9(27.3 - 46.6)	9.4(9.2 - 9.7)	12.5(11.8 - 13.1)	2.7(2.7 - 2.7)	7.7(6.6 – 9.0)
**Bangladesh**	50.4(43.2 - 58.7)	206.5(160.8 - 262.6)	0.5(0.4 - 0.6)	6.2(4.9 - 7.7)	2.4(2.1 - 2.7)	1.8(1.7 - 1.9)	2.4(2.4 - 2.4)	17.2(15.4 - 19.2)
**Barbados**	131.9(113.6 - 153.3)	136.7(112.8 - 164.9)	12.2(10.1 - 14.2)	222.3(176.8 - 278.4)	9.8(9.1 - 10.6)	8.3(7.9 - 8.8)	2.8(2.8 - 2.8)	36.7(33.3 - 40.6)
**Belarus**	94.9(82.5 - 108.8)	74.9(59.8 - 93.9)	28.1(23.6 - 33.5)	70.5(55.3 - 89)	12.7(12.3 - 13.2)	11.8(11.2 - 12.4)	2.5(2.4 - 2.5)	6.1(5.5 - 6.7)
**Belgium**	122.9(111.3 - 135.8)	71.9(60.9 - 84.8)	30.1(25.6 - 35.3)	64.6(52.2 - 80.1)	15.9(15.5 - 16.3)	13.8(13.6 - 14)	2.7(2.7 - 2.7)	17.8(15.7 - 20.1)
**Belize**	117(101 - 135.7)	83.6(68.6 - 100.5)	29(24.4 - 33.7)	68.9(53.8 - 87.7)	9.4(8.7 - 10.2)	10.6(10 - 11.1)	2.5(2.4 - 2.5)	8.5(7.7 - 9.4)
**Benin**	60.3(50.3 - 72.3)	95.4(77.9 - 116.6)	17.1(14.2 - 20.1)	43(30.5 - 60)	12.1(11.8 - 12.5)	16.9(16.5 - 17.2)	2.6(2.6 - 2.6)	7.7(6.8 - 8.7)
**Bhutan**	83(70.8 - 97.4)	271.6(212 - 346.8)	1.8(1.6 - 2)	19(15.2 - 23.7)	6(5.5 - 6.6)	5.9(5.6 - 6.2)	2.6(2.5 - 2.6)	22.6(20.2 - 25.3)
**Bolivia**	87.8(75.5 - 101.9)	115.8(95.6 - 139.9)	6.7(5.6 - 7.9)	79(61.4 - 100.9)	10.7(9.9 - 11.5)	10.8(10.3 - 11.4)	2.5(2.5 - 2.6)	5.9(5.3 - 6.5)
**Bosnia and Herzegovina**	44.7(39.1 - 51.3)	111.1(88 - 139.8)	9(7.6 - 10.7)	28.8(22.7 - 36.5)	11.6(11.2 - 12.1)	24.6(23.3 - 25.8)	2.5(2.5 - 2.6)	4.3(3.9 - 4.7)
**Botswana**	45.3(37.6 - 54.4)	111.3(90.3 - 137.7)	15.5(12.2 - 18.8)	43.3(28.7 - 58.3)	10.1(9.8 - 10.3)	11.5(11.3 - 11.8)	2.6(2.6 - 2.6)	51.1(45.5 - 57.4)
**Brazil**	88(75.6 - 102.1)	90(74.2 - 109.1)	12(10 - 14.1)	38.2(29.9 - 48.9)	8(7.3 - 8.7)	11.8(11.2 - 12.3)	2.7(2.6 - 2.7)	13.6(12.3 - 15)
**Brunei**	99.6(87.8 - 111)	132.9(115.1 - 153.4)	32.3(25.2 - 41.5)	78(63.5 - 92.6)	11.1(10.2 - 12.1)	8.5(7.5 - 9.4)	2.5(2.5 - 2.5)	11.9(10.1 - 14)
**Bulgaria**	96.5(84.4 - 110.7)	435.6(360 - 527)	29.4(24.6 - 34.9)	111.6(88.2 - 142.3)	12.6(12.1 - 13.1)	9.8(9.2 - 10.4)	3(2.9 - 3)	23.8(21.5 - 26.2)
**Burkina Faso**	34.5(28.8 - 41.3)	66.3(54.1 - 81.1)	2(1.7 - 2.4)	8.6(6.1 - 12.1)	11.9(11.6 - 12.2)	17.7(17.4 - 18.1)	2.6(2.5 - 2.6)	11.6(10.3 - 13)

**Burundi**	79.8(66.3 - 95.5)	193.9(156.8 - 238.1)	2.1(1.7 - 2.5)	17.1(12 - 24)	11(10.6 - 11.3)	13.7(13.3 - 14)	2.5(2.5 - 2.6)	27.2(24.1 - 30.6)
**Cambodia**	56.8(50.5 - 63.3)	111.0(95.9 - 127.9)	6.1(4.6 - 8.1)	18.3(14.8 - 21.8)	12.2(11.2 - 13.2)	6.9(6.1 - 7.8)	2.6(2.5 - 2.6)	4.9(4.1 - 5.8)
**Cameroon**	76.6(63.8 - 91.4)	131(106.7 - 159.9)	5.9(4.9 - 7)	43.6(30.9 - 61.3)	4.7(4.5 - 4.9)	16.9(16.6 - 17.2)	2.5(2.5 - 2.6)	18.8(16.6 - 21.1)
**Canada**	107.7(97.2 - 118.9)	110.6(93.8 - 130.3)	16.7(14.2 - 19.6)	52.9(42.6 - 65.5)	11.5(11.2 - 11.9)	12.3(12.1 - 12.5)	2.5(2.5 - 2.5)	11.3(10 - 12.8)
**Cape Verde**	73.1(61.2 - 87.4)	51(41.6 - 62.7)	4.7(3.8 - 5.5)	20.7(14.5 - 29.2)	10.2(9.9 - 10.4)	12.7(12.5 - 13)	2.6(2.6 - 2.6)	60.1(54.1 - 66.8)
**Central African Republic**	78.8(65.8 - 94.2)	121.8(99.4 - 149.6)	3.9(3.2 - 4.6)	90.6(64.9 - 126.4)	9.5(9.2 - 9.8)	10(9.8 - 10.2)	2.7(2.6 - 2.7)	20.1(17.9 - 22.7)
**Chad**	230.3(192.4 - 276.5)	133.4(108.4 - 164.3)	4.5(3.7 - 5.3)	53.2(37.5 - 75.3)	12(11.7 - 12.3)	10.6(10.3 - 10.8)	2.6(2.6 - 2.6)	15.3(13.6 - 17.2)
**Chile**	112.7(96.4 - 130.7)	133.6(110.8 - 161.5)	19.6(16.2 - 23)	16.6(12.9 - 21.3)	9.1(8.4 - 9.9)	8.9(8.5 - 9.3)	3.1(3.1 - 3.2)	16.1(14.6 - 17.8)
**China**	96.7(85.2 - 107.9)	25.5(22 - 29.5)	3.3(2.5 - 4.3)	22(17.9 - 26.3)	7(6.3 - 7.7)	6.7(6 - 7.5)	2.8(2.8 - 2.8)	4.4(3.7 - 5.2)
**Colombia**	102(87.6 - 118.4)	92.2(76.1 - 111.6)	64.9(53.6 - 75.9)	99.9(77.4 - 127.9)	8(7.3 - 8.7)	13(12.4 - 13.6)	2.6(2.5 - 2.6)	17.4(15.7 - 19.2)
**Comoros**	86.2(72.2 - 102.9)	98.1(79.5 - 121)	2.6(2.1 - 3.1)	34.2(24.5 - 47.7)	11.2(10.9 - 11.6)	13.9(13.6 - 14.2)	2.6(2.6 - 2.6)	18.7(16.6 - 21.1)
**Congo**	89.6(74.4 - 107.6)	146.6(118.9 - 179.3)	9.9(8.1 - 11.7)	15.9(11.3 - 22.4)	8.4(8.2 - 8.6)	13.6(13.4 - 13.9)	2.5(2.5 - 2.5)	23.6(21 - 26.6)
**Costa Rica**	118.9(101.7 - 138.6)	77.7(64.1 - 93.9)	32.5(27.3 - 37.6)	65.7(51.3 - 84.7)	10.2(9.4 - 11)	9.4(9 - 9.9)	2.5(2.5 - 2.5)	13.2(11.9 - 14.5)
**Cote d'Ivoire**	59.1(49.6 - 70.2)	95.1(77.6 - 116.5)	4(3.2 - 4.7)	23.7(16.8 - 33)	9(8.8 - 9.3)	14.1(13.9 - 14.4)	2.5(2.5 - 2.5)	21.4(19 - 24.1)
**Croatia**	58.5(51 - 67)	69.9(55.3 - 88.6)	31.2(26.1 - 37.3)	64.6(50.7 - 82.1)	11.7(11.2 - 12.1)	12.4(11.8 - 13)	2.5(2.5 - 2.6)	27.4(24.7 - 30.3)
**Cuba**	73.8(63.5 - 85.7)	30.4(25.2 - 36.7)	42(35.8 - 48.4)	95.8(74.8 - 123)	9.5(8.8 - 10.3)	21(20.1 - 22)	2.7(2.6 - 2.7)	2.8(2.6 - 3.1)
**Cyprus**	233.5(211.6 - 258.4)	139.8(118.2 - 165.2)	8.7(7.4 - 10.2)	76.5(61.7 - 94.7)	11.8(11.5 - 12.2)	12.2(12.1 - 12.4)	2.7(2.7 - 2.7)	10.3(9.1 - 11.7)
**Czech Republic**	76(66 - 87.5)	83(65.6 - 104.7)	38.6(32.5 - 45.6)	93.3(73.5 - 118.4)	14(13.5 - 14.5)	10.6(10 - 11.2)	2.5(2.5 - 2.6)	25(22.7 - 27.7)
**Democratic Republic of the Congo**	95.8(79.9 - 115.1)	227.4(183.6 - 279.9)	10.6(8.6 - 12.6)	8.6(6.1 - 12)	17.0(16.6 - 17.4)	16.1(15.9 - 16.4)	2.6(2.6 - 2.6)	11.6(10.3 - 13)
**Denmark**	109.3(98.5 - 121)	62.8(53.3 - 74.3)	30.2(25.7 - 35.3)	85.3(69 - 105.1)	15.4(14.9 - 15.8)	15(14.8 - 15.2)	2.6(2.6 - 2.6)	14.8(13 - 16.7)
**Djibouti**	33.8(28.1 - 40.7)	103.7(83.9 - 128.5)	5.9(4.8 - 6.9)	23.4(16.7 - 32.8)	12.5(12.2 - 12.9)	14.8(14.5 - 15.1)	2.7(2.7 - 2.7)	63.1(56.9 - 70.2)
**Dominica**	97.7(84.4 - 113.2)	100.3(83.3 - 121.3)	24.9(20.8 - 29)	38.7(30.2 - 49.3)	10.6(9.8 - 11.4)	12.9(12.3 - 13.6)	2.5(2.5 - 2.5)	12.3(11.1 - 13.7)
**Dominican Republic**	103.1(89 - 119.3)	19.2(15.9 - 23.2)	17.7(14.8 - 20.7)	45.2(35 - 58)	9(8.3 - 9.8)	8.4(8 - 8.8)	2.7(2.7 - 2.7)	9.4(8.5 - 10.4)
**Ecuador**	117.1(100.9 - 135.2)	106.6(88 - 129)	2.9(2.4 - 3.4)	48.6(37.9 - 62.1)	11.4(10.6 - 12.3)	7.8(7.4 - 8.2)	2.6(2.5 - 2.6)	14.0(12.6 - 15.4)
**Egypt**	102.1(83.7 - 124.5)	160.3(122.2 - 210)	3.7(3.0 - 4.4)	15(11.4 - 19.6)	6.8(6.6 – 7.0)	17.7(16.9 - 18.5)	2.6(2.6 - 2.6)	21.2(18.2 - 24.5)
**El Salvador**	106.5(91.7 - 123.8)	66.9(55.2 - 81.1)	23.4(19.6 - 27.3)	20.6(16 - 26.6)	7.5(6.8 - 8.1)	10.4(10 - 10.9)	2.6(2.5 - 2.6)	8.5(7.7 - 9.4)
**Equatorial Guinea**	73.2(61.2 - 87.7)	146.3(118.3 - 178.7)	4.1(3.4 - 4.9)	29.4(20.6 - 41.7)	10.6(10.3 - 10.9)	11.8(11.6 - 12.1)	2.6(2.6 - 2.6)	21.3(18.9 - 24)
**Eritrea**	55.2(45.9 - 66.4)	71(57.5 - 87.3)	2.1(1.7 - 2.5)	27.8(19.6 - 39.1)	11.1(10.8 - 11.5)	13.8(13.5 - 14.1)	2.6(2.5 - 2.6)	24.2(21.5 - 27.3)
**Estonia**	105.7(92.1 - 121.1)	100.9(80.5 - 126.5)	102.9(86.7 - 122.4)	105.7(83.5 - 134.1)	13.1(12.6 - 13.6)	12.8(12 - 13.5)	2.5(2.5 - 2.6)	14.6(13.2 - 16.1)
**Ethiopia**	35.4(29.6 - 42.3)	90.6(73.5 - 111.3)	4.7(3.9 - 5.6)	16.3(11.5 - 22.8)	11.9(11.6 - 12.3)	34.8(34.4 - 35.2)	2.6(2.5 - 2.6)	25.5(22.6 - 28.8)

**Federated States of Micronesia**	126.5(113.1 - 141.4)	170.5(146.1 - 198.4)	10.1(7.5 - 13.4)	16.7(13.2 - 20.2)	13.6(12.7 - 14.6)	7.7(6.5 - 8.8)	2.6(2.6 - 2.6)	6.7(5.6 - 8.1)
**Fiji**	128.7(113.4 - 144.4)	150.4(130.2 - 173.7)	15(11.3 - 19.9)	49.8(40.1 - 59.5)	17.1(15.9 - 18.3)	10.8(9.7 - 12)	2.6(2.6 - 2.6)	4.7(3.9 - 5.5)
**Finland**	116.9(105.9 - 129)	72.9(61.5 - 86.5)	41.7(35.4 - 49.1)	56.4(45.4 - 69.9)	15.1(14.7 - 15.5)	12(11.9 - 12.2)	2.5(2.5 - 2.5)	18.2(16.1 - 20.6)
**France**	52.2(47.2 - 57.6)	148.3(125.4 - 175)	32.4(27.4 - 38.1)	70.4(56.5 - 87.1)	14.6(14.2 - 15)	11.5(11.4 - 11.7)	2.6(2.6 - 2.7)	23(20.3 - 25.9)
**Gabon**	94.6(78.6 - 113.3)	122.1(98.6 - 150.9)	7.0(5.7 - 8.2)	51.6(36.8 - 72.4)	12.5(12.2 - 12.8)	13.9(13.6 - 14.2)	2.7(2.6 - 2.7)	42.9(38.2 - 48)
**Georgia**	74.9(65.3 - 85.6)	74.9(59.4 - 94.6)	37.7(31.9 - 44.7)	21.7(17.1 - 27.7)	12.5(12 - 12.9)	20.2(19.2 - 21.3)	2.5(2.5 - 2.6)	9.6(8.7 - 10.6)
**Germany**	172.1(155.9 - 189.2)	79.1(67 - 93.1)	62.6(53.2 - 73.9)	64.7(52.2 - 80.3)	15.6(15.2 - 16)	12.6(12.4 - 12.8)	2.6(2.6 - 2.6)	58.9(51.9 - 66.7)
**Ghana**	81.1(67.5 - 97.5)	139.9(114 - 172.2)	1.2(1 - 1.4)	37.1(26.2 - 51.9)	9.7(9.4 - 10)	8.8(8.6 - 9)	2.6(2.5 - 2.6)	8.6(7.6 - 9.6)
**Greece**	160.7(145.7 - 177.3)	101(85.4 - 119)	3.8(3.3 - 4.5)	69.4(55.8 - 85.7)	13.5(13.1 - 13.9)	12.3(12.1 - 12.5)	2.7(2.7 - 2.7)	21.5(18.9 - 24.4)
**Grenada**	141.8(121.8 - 165.2)	113.3(94.1 - 137.1)	6.7(5.5 - 7.8)	23(17.8 - 29.4)	11.4(10.6 - 12.3)	6.3(6 - 6.7)	2.5(2.5 - 2.6)	14.2(12.8 - 15.6)
**Guatemala**	65.2(56.1 - 75.6)	100.3(83.6 - 120.3)	23.6(19.7 - 27.4)	19.9(15.5 - 25.4)	3.7(3.3 - 4.2)	6.5(6.2 - 6.9)	2.7(2.7 - 2.7)	16.1(14.6 - 17.8)
**Guinea**	66.4(55.5 - 79.8)	121.3(98.5 - 148.7)	4.2(3.4 - 4.9)	32.7(23.2 - 46.2)	12.1(11.8 - 12.5)	10.7(10.4 - 10.9)	2.6(2.6 - 2.6)	16.8(14.9 - 18.9)
**Guinea-Bissau**	52.4(43.8 - 62.1)	20.7(16.9 - 25.3)	17.2(14.1 - 20.3)	3.3(2.3 - 4.6)	8.4(8.1 - 8.6)	10.1(9.9 - 10.3)	2.4(2.4 - 2.5)	20.5(18.2 - 23.1)
**Guyana**	109.6(94.1 - 127.1)	86.4(71.3 - 104.5)	75.1(66.9 - 83.4)	45.4(34.8 - 58.5)	7.5(6.9 - 8.2)	9.9(9.5 - 10.3)	2.3(2.2 - 2.3)	2.8(2.5 - 3.1)
**Haiti**	71(61.2 - 82.7)	87.6(72.3 - 106.2)	9.6(8 - 11.2)	14.6(11.4 - 18.5)	9.3(8.6 - 10.1)	19.3(18.3 - 20.3)	2.5(2.5 - 2.6)	12.7(11.4 - 14)
**Honduras**	88.4(76.2 - 102.3)	30.3(25.2 - 36.8)	26.1(21.9 - 30.4)	14.4(11.2 - 18.4)	8.6(7.9 - 9.3)	9.5(9.1 - 10)	2.5(2.5 - 2.5)	11.7(10.5 - 12.9)
**Hungary**	72.3(63.1 - 82.6)	58.9(46.4 - 74.1)	21.2(17.7 - 25.2)	29.6(23 - 37.8)	14(13.5 - 14.5)	10.5(9.9 - 11.1)	2.8(2.7 - 2.8)	22.8(20.6 - 25.3)
**Iceland**	91.4(82.7 - 100.9)	62.2(52.5 - 73.3)	32.6(27.5 - 38.5)	51.6(41.9 - 63.6)	16.9(16.4 - 17.3)	13.7(13.5 - 13.9)	2.4(2.4 - 2.4)	13.0(11.5 - 14.7)
**India**	30.7(26.1 - 35.8)	155.2(120.3 - 199.5)	3.2(2.8 - 3.6)	2.4(1.9 - 3)	5.4(4.9 - 5.9)	6.6(6.3 - 7)	2.7(2.7 - 2.7)	28.6(25.5 - 32)
**Indonesia**	75.4(66.2 - 84.2)	151.9(131.5 - 175.2)	11.5(8.6 - 15.3)	19.4(15.8 - 23.1)	14.3(13.2 - 15.4)	9.8(8.7 - 10.9)	2.6(2.5 - 2.6)	6.9(5.8 - 8.2)
**Iran**	183.1(149.6 - 224.5)	159.7(122.6 - 207)	3.9(3.2 - 4.7)	30.1(23.1 - 39.1)	8.6(8.4 - 8.9)	10.4(9.8 - 11.1)	2.8(2.8 - 2.9)	7.4(6.4 - 8.7)
**Iraq**	125.1(102.2 - 153)	193.2(146.5 - 253)	79.9(71.4 - 88.6)	176.7(137.7 - 227.5)	9.7(9.5 - 10)	14.2(13.5 - 14.9)	2.6(2.6 - 2.6)	46.1(39.8 - 53.2)
**Ireland**	92.1(83.5 - 101.3)	102.6(87.1 - 121)	38.4(32.6 - 45.3)	40.7(32.7 - 50.1)	14.7(14.3 - 15.1)	13.4(13.2 - 13.5)	2.6(2.6 - 2.6)	15.9(14.1 - 18)
**Israel**	217.3(176.5 - 266.3)	120.8(93.1 - 156.1)	101(88.2 - 115.4)	121.9(93.6 - 158.6)	6.8(6.6 - 7)	21.5(20.6 - 22.4)	2.6(2.6 - 2.6)	16.1(14 - 18.7)
**Italy**	200.1(180.7 - 221.4)	125.5(106.1 - 148.5)	21.2(17.9 - 24.9)	48.8(39.3 - 60.3)	10.7(10.4 - 11)	12.7(12.5 - 12.9)	2.4(2.4 - 2.4)	11.8(10.5 - 13.3)
**Jamaica**	174.6(150.7 - 202.5)	153.4(127 - 184.7)	7.8(6.5 - 9.2)	18.8(14.6 - 24.1)	5.5(5 - 6.1)	8.4(8 - 8.8)	2.8(2.8 - 2.8)	10.4(9.5 - 11.4)
**Japan**	119.4(105.4 - 133.4)	246.8(212.6 - 285.2)	13.3(10 - 17.6)	34(27.4 - 40.3)	7.1(6.4 - 7.9)	9.3(8.3 - 10.4)	2.6(2.6 - 2.6)	11.1(9.4 - 13.2)
**Jordan**	189(154.5 - 230.6)	132.4(100.5 - 173.2)	10.8(8.7 - 12.8)	47.8(36.2 - 62.4)	6.2(6 - 6.4)	5.2(4.9 - 5.6)	2.6(2.5 - 2.6)	45.9(39.6 - 53.3)
**Kazakhstan**	60.1(52.3 - 68.7)	105.1(83.3 - 132.3)	38.2(32.3 - 45.2)	98.1(77.2 - 124.7)	12.7(12.2 - 13.2)	12.7(12.1 - 13.4)	2.5(2.4 - 2.5)	13.1(11.8 - 14.4)
**Kenya**	102.1(85.3 - 121.5)	58.5(47.2 - 71.8)	0.8(0.7 - 0.9)	21.2(15 - 30.1)	12(11.7 - 12.3)	18(17.6 - 18.4)	2.6(2.5 - 2.6)	22.1(19.6 - 24.8)

**Kiribati**	82.6(73.8 - 92.3)	104.1(89.3 - 121)	9.9(7.4 - 13.2)	16.9(13.2 - 20.5)	12.7(11.8 - 13.6)	7.3(6.1 - 8.4)	2.6(2.6 - 2.6)	7.8(6.5 - 9.4)
**Kuwait**	61.1(49.9 - 75.3)	76(58.2 - 98.5)	4(3.3 - 4.8)	10.4(7.9 - 13.7)	9.5(9.3 - 9.8)	8.7(8.2 - 9.1)	2.6(2.5 - 2.6)	6.0(5.1 - 6.9)
**Kyrgyzstan**	80.6(70.4 - 92.3)	39.7(31.3 - 50.1)	38.8(32.8 - 45.7)	78.5(61.1 - 99.4)	12.6(12.1 - 13)	26.7(25.6 - 27.9)	2.5(2.4 - 2.5)	8.9(8 - 9.8)
**Laos**	134.2(118.4 - 150.2)	143.6(124.5 - 165.8)	0.9(0.6 - 1.1)	11.4(9.2 - 13.6)	12.3(11.3 - 13.3)	7.1(6.2 - 8)	2.6(2.5 - 2.6)	7.6(6.4 – 9.0)
**Latvia**	45.4(39.6 - 52)	134.3(105.5 - 169)	50.6(42.6 - 60.1)	213.7(170 - 269.1)	12.8(12.3 - 13.2)	10.5(10 - 11.1)	2.5(2.4 - 2.5)	8.8(7.9 - 9.6)
**Lebanon**	471.2(402.1 - 549.8)	366(278.9 - 476.3)	7.9(6.4 - 9.3)	20.6(15.8 - 26.6)	10.1(9.9 - 10.4)	21.1(20.2 - 21.9)	2.9(2.9 - 3)	63.7(54.8 - 73.8)
**Lesotho**	64.1(53.7 - 76.6)	438.1(358 - 535.6)	3.1(2.6 - 3.7)	21.7(15.5 - 30.7)	12(11.6 - 12.3)	10.5(10.2 - 10.7)	2.6(2.5 - 2.6)	39.4(35 - 44.2)
**Liberia**	55.8(46.7 - 66.8)	128.3(104.1 - 157.5)	17.1(14.1 - 20.3)	42.2(30.1 - 59.4)	12.4(12.1 - 12.7)	10.8(10.5 - 11)	2.6(2.6 - 2.7)	13(11.6 - 14.7)
**Libya**	57.5(46.9 - 70.2)	115.9(88.9 - 150.9)	11.8(9.6 - 13.9)	34.6(26.5 - 45.7)	9.8(9.6 - 10.1)	11.6(11 - 12.2)	2.5(2.5 - 2.6)	50.8(44.1 - 58.6)
**Lithuania**	63(54.9 - 72)	64.7(51 - 82.4)	29.7(24.9 - 35.2)	149.6(117.6 - 189.5)	11.7(11.2 - 12.1)	13.6(12.9 - 14.3)	2.5(2.4 - 2.5)	4(3.6 - 4.4)
**Luxembourg**	99.8(90.5 - 110.1)	94.2(79.7 - 111.5)	33.3(28.4 - 38.9)	69.9(56.7 - 86.4)	15.2(14.8 - 15.6)	14.4(14.3 - 14.6)	2.7(2.7 - 2.7)	18.1(16 - 20.5)
**Macedonia**	64(55.9 - 73.2)	126.8(100.6 - 159.9)	31.9(26.9 - 38)	29.0(22.7 - 36.8)	11.7(11.3 - 12.2)	12.2(11.6 - 12.8)	2.5(2.5 - 2.6)	22.6(20.4 - 25.1)
**Madagascar**	85.8(71.7 - 102.2)	177.6(143.7 - 218.3)	5.6(4.6 - 6.7)	46.9(33.2 - 66)	12(11.8 - 12.3)	10.6(10.4 - 10.8)	2.6(2.6 - 2.6)	27.3(24.3 - 30.7)
**Malawi**	113.3(94.5 - 136.1)	230.8(188.2 - 283.7)	2.4(2 - 2.9)	18.1(12.7 - 25.6)	5.3(5.1 - 5.5)	15.5(15.2 - 15.8)	2.5(2.5 - 2.5)	10.6(9.4 - 11.9)
**Malaysia**	124.3(109.9 - 139.1)	121.7(104.9 - 140.5)	15.7(11.8 - 20.9)	19.0(15.4 - 22.7)	13.3(12.2 - 14.4)	9.8(8.7 - 10.9)	2.5(2.5 - 2.5)	25.4(21.4 - 30.2)
**Maldives**	21(17.8 - 24.6)	37.5(28.9 - 48.6)	0.9(0.8 - 1)	5.4(4.3 - 6.8)	8.1(7.4 - 8.8)	9.3(8.8 - 9.9)	2.6(2.6 - 2.6)	12.2(10.8 - 13.6)
**Mali**	65.1(54.3 - 78.2)	100.8(81.9 - 123.1)	9.2(7.6 - 10.9)	22.3(15.9 - 31.5)	8.2(7.9 - 8.4)	15.2(14.8 - 15.5)	2.6(2.6 - 2.6)	51.7(46.1 - 58)
**Malta**	154.6(139.9 - 171.5)	76(64.2 - 89.7)	30.8(26 - 36.1)	71.4(57.9 - 88.2)	13.6(13.3 - 14)	12.8(12.6 - 13)	2.7(2.7 - 2.7)	12.2(10.8 - 13.8)
**Marshall Islands**	104(91.5 - 116.3)	173.8(148.9 - 202.7)	8(5.9 - 10.7)	36.7(29 - 44.6)	13.7(12.8 - 14.7)	7.8(6.6 - 8.9)	2.6(2.6 - 2.6)	7.2(6 - 8.7)
**Mauritania**	19.5(16.3 - 23.3)	66.7(54.2 - 82.3)	28.6(23.7 - 33.5)	35.1(25 - 48.8)	12.2(11.9 - 12.5)	15.4(15 - 15.7)	2.6(2.6 - 2.6)	68.8(62.7 - 75.4)
**Mauritius**	72.6(60.8 - 86.7)	163.6(132.8 - 201.2)	19.3(15.7 - 22.9)	61.1(43.3 - 86.1)	9.2(8.9 - 9.5)	10(9.8 - 10.2)	2.7(2.7 - 2.7)	26.2(23.3 - 29.4)
**Mexico**	84.4(72.4 - 97.7)	82.6(68.4 - 99.3)	19(15.7 - 22.2)	31.8(24.5 - 40.9)	9.4(8.6 - 10.2)	11.1(10.5 - 11.6)	2.3(2.3 - 2.4)	15.8(14.3 - 17.5)
**Moldova**	69.6(60.8 - 79.7)	73.6(58.8 - 92)	58.2(49.8 - 68)	163.8(127.8 - 208.3)	12.7(12.2 - 13.1)	17.7(16.8 - 18.6)	2.5(2.4 - 2.5)	13.1(11.8 - 14.4)
**Mongolia**	50.5(44.3 - 58)	175.3(138.9 - 219.8)	72.3(62.6 - 83)	116.4(91.7 - 148.1)	12(11.6 - 12.5)	15.1(14.3 - 15.9)	2.5(2.5 - 2.6)	32.2(29.1 - 35.6)
**Montenegro**	58.8(51.2 - 67.6)	109.8(87.1 - 138.8)	34.2(28.6 - 40.7)	74(58.4 - 93.6)	11.7(11.2 - 12.1)	8.8(8.3 - 9.2)	2.5(2.5 - 2.6)	32.9(29.7 - 36.3)
**Morocco**	67.8(55.4 - 82.7)	155.2(117.7 - 203.3)	10.7(8.7 - 12.8)	19.4(14.9 - 25.2)	6.5(6.3 - 6.7)	14(13.3 - 14.8)	2.5(2.4 - 2.5)	51.5(44.3 - 59.6)
**Mozambique**	106(88.7 - 126.7)	143.8(116.2 - 178.4)	1.2(1 - 1.5)	7(4.9 - 9.8)	9.6(9.3 - 9.8)	7.2(7 - 7.4)	2.5(2.5 - 2.5)	28(24.9 - 31.6)
**Myanmar**	89.8(79.3 - 100.3)	96.8(83.7 - 111.9)	3.1(2.3 - 4.1)	3.7(3 - 4.4)	12.3(11.3 - 13.3)	7.1(6.2 - 7.9)	2.6(2.5 - 2.6)	5.9(4.9 - 7)
**Namibia**	77.6(65 - 93.1)	164.4(133.6 - 201.9)	13.3(10.8 - 15.7)	58.5(41.3 - 82.5)	12.2(11.8 - 12.5)	18.7(18.3 - 19.1)	2.6(2.6 - 2.6)	28.7(25.6 - 32.3)
**Nepal**	14.8(12.7 - 17.4)	189.5(146.8 - 243.3)	0.6(0.5 - 0.7)	9.2(7.3 - 11.5)	3.9(3.5 - 4.3)	5.5(5.2 - 5.8)	2.3(2.3 - 2.3)	14.3(12.8 - 15.9)

**Netherlands**	119.9(108.5 - 132.4)	106(89.6 - 125.9)	21.7(18.4 - 25.5)	36.6(29.6 - 45.4)	14.6(14.2 - 15)	11.1(11 - 11.3)	2.8(2.7 - 2.8)	17.7(15.7 - 20)
**New Zealand**	193.3(175.5 - 213.2)	111.5(94.1 - 131.8)	26.1(22.1 - 30.9)	56.6(45.7 - 69.7)	15.7(15.3 - 16.1)	15(14.8 - 15.1)	2.6(2.6 - 2.7)	18.8(16.6 - 21.4)
**Nicaragua**	112.7(97.2 - 130.6)	112.5(93.1 - 135.9)	21.6(18.1 - 25.1)	71.6(55.9 - 91.9)	9.8(9.1 - 10.7)	15(14.2 - 15.8)	2.6(2.5 - 2.6)	8.8(7.9 - 9.6)
**Niger**	64(53.5 - 76.4)	105.6(85.8 - 129.8)	5.8(4.8 - 6.9)	11.9(8.3 - 16.7)	11.9(11.5 - 12.2)	16.2(15.9 - 16.6)	2.6(2.5 - 2.6)	20.8(18.5 - 23.5)
**Nigeria**	34.8(29 - 41.7)	95.4(78 - 116.9)	8.5(6.9 - 10)	28(19.9 - 39.6)	9.4(9.2 - 9.7)	13.4(13.1 - 13.6)	2.6(2.5 - 2.6)	10.3(9.1 - 11.5)
**Norway**	98.4(89 - 108.6)	66.1(55.9 - 77.9)	23(19.5 - 27)	64.9(52 - 80.9)	13.6(13.3 - 14)	11.3(11.2 - 11.5)	2.7(2.7 - 2.7)	19.9(17.5 - 22.4)
**Oman**	107.9(88.1 - 132)	157.8(120.1 - 206.5)	11.2(9.1 - 13.3)	25.5(19.5 - 33.4)	9.2(9 - 9.5)	12.3(11.7 - 13)	2.7(2.7 - 2.7)	22.5(19.4 - 26)
**Pakistan**	33.5(28.6 - 39.3)	86.4(66.7 - 110.9)	4.6(4.1 - 5.3)	19.9(15.9 - 24.9)	7.2(6.7 - 7.9)	5.2(5 - 5.5)	2.6(2.6 - 2.6)	13.6(12.2 - 15.2)
**Palestine**	107.1(87.5 - 131.1)	155(117.9 - 204.3)	12.5(10.1 - 14.8)	34.3(26.4 - 44.5)	11.7(11.4 - 12)	6.9(6.6 - 7.3)	2.7(2.7 - 2.7)	24.4(21 - 28.3)
**Panama**	123.4(106 - 143.4)	87.7(72.8 - 105.8)	36.3(30.6 - 41.7)	69.1(53.7 - 88.3)	9.1(8.4 - 9.9)	12.3(11.7 - 12.9)	2.7(2.6 - 2.7)	8.0(7.3 - 8.8)
**Papua New Guinea**	56.4(50.4 - 63)	81.5(70.5 - 94.3)	6.2(4.6 - 8.3)	31.7(25.9 - 37.7)	13.2(12.1 - 14.2)	7.5(6.6 - 8.4)	2.6(2.5 - 2.6)	12.7(10.7 - 15)
**Paraguay**	168.3(144.3 - 195)	73.4(60.7 - 88.4)	31(26.1 - 35.9)	72.5(56.4 - 92.6)	8.3(7.6 - 9.1)	8.5(8.1 - 8.9)	2.6(2.6 - 2.6)	12.7(11.5 - 14)
**Peru**	72.7(62.6 - 84.1)	92.5(76.4 - 111.8)	23.8(19.6 - 27.9)	73(56.5 - 93.2)	7.1(6.5 - 7.8)	16.8(16.1 - 17.6)	2.6(2.6 - 2.6)	2.6(2.3 - 2.8)
**Philippines**	116.7(102.8 - 130.3)	110.4(95.6 - 127)	10.9(8.1 - 14.5)	18.3(14.7 - 21.7)	19.7(18.4 - 21)	9(8.1 - 9.9)	2.4(2.3 - 2.4)	6.2(5.2 - 7.3)
**Poland**	184.9(161.1 - 212.1)	159.7(127.1 - 200.9)	48.1(40.3 - 57.5)	92(72.1 - 116.8)	12.6(12.2 - 13.1)	20.8(20 - 21.7)	2.4(2.4 - 2.5)	14.6(13.2 - 16.1)
**Portugal**	128.3(116.1 - 142)	141.2(119.4 - 166.6)	7.6(6.4 - 9)	55.1(44.6 - 68.2)	8.9(8.6 - 9.2)	12.5(12.4 - 12.7)	2.7(2.7 - 2.7)	12.4(10.9 - 14)
**Qatar**	122.8(100.5 - 150.9)	163.0(124.3 - 211.1)	14.1(11.5 - 16.6)	35.0(26.9 - 45.5)	9.3(9.1 - 9.6)	12.4(11.7 - 13)	2.7(2.7 - 2.7)	19.3(16.6 - 22.4)
**Romania**	199.5(174.3 - 228.9)	303.3(241.6 - 382.5)	83.4(69.8 - 99.1)	138.1(108.7 - 175)	11.7(11.3 - 12.2)	15.1(14.4 - 15.9)	2.2(2.1 - 2.2)	12.6(11.3 - 13.9)
**Russia**	72.7(63.3 - 83.6)	92.7(73.7 - 115.9)	34.5(28.9 - 41.2)	195.6(155.3 - 246.2)	12.8(12.3 - 13.2)	14.3(13.6 – 15.0)	2.5(2.4 - 2.5)	11.6(10.5 - 12.8)
**Rwanda**	122.4(102.5 - 146.3)	289(234.3 - 356.8)	1.0(0.8 - 1.2)	4.2(3.0 – 6.0)	11.8(11.5 - 12.1)	36.8(36.6 - 37.1)	2.5(2.5 - 2.6)	7.2(6.4 - 8.2)
**Saint Lucia**	147.5(126.8 - 171.7)	75(62.1 - 90.4)	4.4(3.7 - 5.2)	27.2(21.2 - 34.8)	8.1(7.4 - 8.8)	6.6(6.3 - 6.9)	2.4(2.4 - 2.4)	21.0(19 - 23.2)
**Saint Vincent and the Grenadines**	61.7(52.9 - 72.1)	48.6(39.9 - 58.9)	7.3(6.1 - 8.5)	30.0(23.2 - 38.5)	11.6(10.8 - 12.5)	8.2(7.8 - 8.6)	2.5(2.5 - 2.6)	5.4(4.9 - 5.9)
**Samoa**	139.1(123.6 - 155.2)	340.6(292.6 - 395.5)	7.4(5.5 - 9.9)	50.2(38.9 - 61.3)	27.5(26.3 - 28.8)	7.2(6.1 - 8.2)	2.6(2.5 - 2.6)	9.3(7.8 - 11.2)
**Sao Tome and Principe**	53.6(44.3 - 64.6)	33.7(27.1 - 41.9)	6.6(5.2 - 7.9)	3.9(2.8 - 5.5)	12.2(11.9 - 12.5)	10.7(10.5 - 10.9)	2.6(2.6 - 2.6)	22.8(20.2 - 25.7)
**Saudi Arabia**	101.7(83.2 - 124.2)	226.3(171.8 - 295.4)	46.3(39 - 53.5)	43(32.6 - 56.2)	11.1(10.8 - 11.3)	12.6(11.9 - 13.3)	2.7(2.7 - 2.7)	39.6(34 - 46.1)
**Senegal**	51.9(43.4 - 61.9)	74.1(60.1 - 91.6)	18.7(15.2 - 22.1)	12.1(8.5 - 17.1)	7.6(7.3 - 7.8)	11.1(10.9 - 11.3)	2.6(2.6 - 2.6)	36.6(32.5 - 41.2)
**Serbia**	151.5(132.1 - 173.6)	201.6(160 - 253.7)	32.4(27.2 - 38.7)	82.3(64.7 - 103.7)	15.2(14.7 - 15.7)	11.9(11.2 - 12.6)	2.5(2.5 - 2.6)	40.3(36.4 - 44.7)
**Seychelles**	115.9(96.7 - 138.4)	201.8(164.4 - 248.9)	7.7(6.3 - 9.1)	26(18.3 - 37.1)	11.5(11.2 - 11.8)	14(13.8 - 14.3)	2.6(2.6 - 2.6)	19.7(17.4 - 22.1)
**Sierra Leone**	91.1(76 - 109)	120.8(98.5 - 147.6)	31.6(26.2 - 36.7)	13.9(9.9 - 19.3)	12.1(11.8 - 12.5)	10.7(10.4 - 10.9)	2.6(2.6 - 2.6)	21.1(18.8 - 23.8)
**Singapore**	152.9(135.3 - 170)	134.4(115.7 - 156.7)	9.9(7.5 - 13.1)	20(16.2 - 23.9)	10.3(9.4 - 11.3)	6.6(5.9 - 7.4)	2.6(2.5 - 2.6)	18.1(15.3 - 21.4)

**Slovakia**	66.3(57.7 - 76)	82.8(65.1 - 104.2)	29.1(24.5 - 34.6)	50(39.2 - 63.9)	13.9(13.4 - 14.4)	11.4(10.7 - 12)	2.5(2.5 - 2.5)	42.4(38.4 - 46.8)
**Slovenia**	77.5(67.7 - 88.8)	90.5(71.3 - 114.9)	19.2(16.1 - 22.9)	43.6(34.3 - 55.5)	10.7(10.3 - 11.2)	10.6(10.1 - 11.2)	2.5(2.5 - 2.6)	13.2(11.9 - 14.6)
**Solomon Islands**	61.7(54.4 - 68.8)	172.5(149 - 199.3)	13.8(10.5 - 18.1)	33.4(27 - 39.7)	12.5(11.4 - 13.5)	7.3(6.4 - 8.2)	2.6(2.5 - 2.6)	3.2(2.6 - 3.7)
**South Africa**	66.3(55.7 - 79.1)	124.2(101.3 - 152.6)	12.2(9.9 - 14.4)	153.4(118.3 - 188.2)	10.2(9.9 - 10.5)	11(10.8 - 11.2)	2.7(2.7 - 2.7)	24.1(21.3 - 27.2)
**South Korea**	76.2(67.4 - 84.9)	126.9(109.5 - 147.4)	3.1(2.3 - 4.1)	28.8(23.2 - 34.2)	4.8(4.2 - 5.3)	9.2(8.2 - 10.2)	2.3(2.3 - 2.3)	4.9(4.1 - 5.9)
**South Sudan**	67.6(56.7 - 80.5)	143.6(116.6 - 175.2)	2.3(1.8 - 2.7)	25.7(18.2 - 36.4)	11.1(10.8 - 11.4)	13.7(13.4 - 14)	2.6(2.5 - 2.6)	26.7(23.6 - 29.9)
**Spain**	114.7(103.8 - 126.6)	82.4(69.5 - 97.6)	23.3(19.8 - 27.6)	56.5(45.5 - 69.8)	12.2(11.9 - 12.6)	10.8(10.6 - 10.9)	2.7(2.7 - 2.7)	24.4(21.5 - 27.7)
**Sri Lanka**	72.1(61.7 - 84.1)	191.8(148.6 - 248)	6.8(6 - 7.7)	57.4(45.7 - 71.6)	10.6(9.9 - 11.4)	10.7(10.3 - 11.1)	2.3(2.3 - 2.3)	8.3(7.5 - 9.3)
**Sudan**	79.5(66.2 - 94.8)	132.9(108.4 - 162.7)	3.3(2.7 - 3.9)	28.2(19.9 - 39.3)	10.6(10.3 - 10.9)	12.6(12.4 - 12.9)	2.5(2.5 - 2.5)	28.1(25 - 31.5)
**Suriname**	63.7(54.7 - 74.1)	182.8(151.2 - 220.6)	27.9(23.3 - 32.4)	40.2(31.3 - 51.5)	7.3(6.7 - 7.9)	9.2(8.8 - 9.6)	2.5(2.5 - 2.5)	13.6(12.3 - 15)
**Swaziland**	90.1(74.7 - 108.6)	146.3(118.2 - 181.2)	8.9(7 - 10.8)	29(20.5 - 40.7)	8.5(8.3 - 8.8)	9.8(9.6 - 9.9)	2.5(2.5 - 2.5)	27.2(23.9 - 30.9)
**Sweden**	122.9(111.3 - 135.6)	70.4(59.6 - 83.2)	21.7(18.3 - 25.6)	69.2(55.8 - 85.7)	15.4(15 - 15.8)	27.1(26.9 - 27.3)	2.5(2.5 - 2.5)	12.7(11.3 - 14.5)
**Switzerland**	178.8(161.6 - 197.7)	91.9(77.5 - 108.7)	19.4(16.4 - 23)	70.8(57.1 - 87.7)	14(13.6 - 14.4)	12.5(12.4 - 12.7)	2.6(2.6 - 2.6)	15.7(13.8 - 17.7)
**Syria**	104.6(85.6 - 127.8)	135.4(103.2 - 177.5)	8.5(6.9 - 10.1)	32.7(25.1 - 43)	12.8(12.5 - 13.1)	9(8.5 - 9.6)	2.7(2.7 - 2.8)	25.9(22.3 - 30.1)
**Taiwan**	153.7(135.9 - 171.1)	221.0(191.0 – 255.0)	13.0(9.7 - 17.4)	53.7(43.8 - 63.5)	7.9(7.1 - 8.7)	7.0(6.2 - 7.8)	2.8(2.7 - 2.8)	5.1(4.3 - 6.1)
**Tajikistan**	88.3(76.9 - 100.9)	59.9(47.6 - 75.2)	94.7(83.9 - 107.2)	25.2(20 - 31.8)	12.6(12.1 - 13)	29.2(28.1 - 30.3)	2.5(2.4 - 2.5)	10.8(9.8 - 11.9)
**Tanzania**	56.9(47.9 - 67.9)	179.0(145.0 – 222.0)	2.1(1.7 - 2.5)	40.5(28.4 - 56.6)	12(11.7 - 12.4)	10.6(10.4 - 10.8)	2.6(2.6 - 2.6)	12.7(11.2 - 14.3)
**Thailand**	100.3(88.9 - 112.1)	160.6(138.8 - 185.9)	7.5(5.6 - 9.9)	26.8(21.8 - 31.9)	10.9(9.9 - 11.8)	10.2(9.2 - 11.3)	2.4(2.4 - 2.4)	2.8(2.4 - 3.3)
**The Bahamas**	70.5(60.5 - 81.7)	102.7(84.6 - 124.1)	13.1(10.9 - 15.3)	91.1(70.8 - 117.2)	10.7(10 - 11.6)	6.9(6.5 - 7.3)	2.5(2.5 - 2.5)	13.3(12 - 14.7)
**The Gambia**	12(9.9 - 14.4)	60.8(48.9 - 75.4)	15.5(12.3 - 18.8)	17.1(12 - 23.8)	12.2(11.9 - 12.5)	10.7(10.4 - 10.9)	2.6(2.6 - 2.6)	41.5(36.7 - 46.9)
**Timor-Leste**	63.8(56.1 - 71.4)	136.5(116.8 - 158)	7.4(5.6 - 9.8)	20.8(16.3 - 25.2)	12.6(11.7 - 13.5)	7.1(6 - 8.2)	2.6(2.5 - 2.6)	5.9(4.9 - 7)
**Togo**	57.7(48.1 - 69.4)	169.5(138.1 - 207.6)	14.3(11.8 - 16.8)	25.5(18.1 - 35.8)	12.1(11.8 - 12.5)	10.6(10.4 - 10.8)	2.6(2.6 - 2.6)	13.8(12.2 - 15.5)
**Tonga**	119.2(106.6 - 133)	135.4(116.3 - 157.5)	7.4(5.5 - 10)	35.2(27.4 - 42.8)	13.6(12.6 - 14.5)	7.6(6.5 - 8.8)	2.6(2.5 - 2.6)	5.5(4.6 - 6.5)
**Trinidad and Tobago**	69.6(59.9 - 80.6)	60.5(49.7 - 73)	15.8(13.2 - 18.3)	29.7(23.2 - 38.1)	10.2(9.4 - 11)	8.8(8.3 - 9.2)	2.6(2.6 - 2.6)	8.3(7.5 - 9.2)
**Tunisia**	70.1(56.8 - 86.5)	106.3(81.7 - 138.2)	0.7(0.5 - 0.8)	36.9(28.3 - 47.9)	8.3(8.1 - 8.5)	13.6(12.9 - 14.4)	3.1(3.1 - 3.1)	27.5(23.8 - 31.9)
**Turkey**	84.9(69.2 - 103.6)	98.4(75.2 - 127.8)	3.7(3 - 4.4)	42(32.1 - 54.2)	9(8.7 - 9.2)	11.6(10.9 - 12.3)	2.8(2.7 - 2.8)	19.4(16.6 - 22.5)
**Turkmenistan**	41.3(36 - 47.3)	71.3(56.6 - 89.7)	62.2(53.4 - 72.6)	40.1(31.4 - 51.3)	12.6(12.2 - 13.1)	14.2(13.5 - 14.9)	2.5(2.4 - 2.5)	6.6(5.9 - 7.3)
**Uganda**	53.6(44.7 - 64.1)	116.7(95.1 - 142.8)	0.7(0.6 - 0.9)	11.5(8.2 - 16)	11.9(11.6 - 12.2)	10.5(10.3 - 10.8)	2.6(2.5 - 2.6)	10.2(9.1 - 11.5)
**Ukraine**	80.7(70.5 - 92.4)	152(121.2 - 191)	57(48.5 - 67.1)	89.7(70.2 - 113.6)	12.7(12.3 - 13.2)	14.6(13.9 - 15.3)	2.5(2.4 - 2.5)	7.9(7.2 - 8.8)
**United Arab Emirates**	149.6(122.2 - 184.5)	150.8(115 - 197.5)	54.4(46.3 - 62.2)	112.1(85.3 - 145.9)	9.5(9.2 - 9.7)	10.2(9.5 - 10.8)	2.5(2.4 - 2.5)	18.5(16 - 21.4)

**United Kingdom**	107.5(97.5 - 118.7)	99.5(84.3 - 117.5)	29.1(24.7 - 34.1)	42.7(34.5 - 53)	13.3(12.9 - 13.7)	13.8(13.6 - 13.9)	2.6(2.6 - 2.6)	14.1(12.5 - 16)
**United States**	97.5(88.4 - 107.6)	116.4(98.2 - 137.7)	21.8(18.5 - 25.6)	42.8(34.6 - 52.7)	11.5(11.2 - 11.9)	12.9(12.7 - 13.1)	2.7(2.7 - 2.7)	14.5(12.8 - 16.3)
**Uruguay**	100.2(86.1 - 116.6)	41.9(34.6 - 50.7)	28.3(23.6 - 32.8)	116.8(90.6 - 150.4)	10.6(9.8 - 11.4)	11.6(11 - 12.2)	2.6(2.5 - 2.6)	11.2(10.1 - 12.4)
**Uzbekistan**	86.7(75.4 - 99.6)	128.4(102.1 - 161.1)	46.1(39.2 - 54.6)	37(29 - 46.8)	12.6(12.2 - 13.1)	24.9(23.7 - 26)	2.5(2.4 - 2.5)	14.7(13.3 - 16.2)
**Vanuatu**	47.6(42.5 - 53.3)	70.7(60.9 - 82)	8.9(6.7 - 11.8)	33.8(26.3 – 41.0)	12.6(11.8 - 13.5)	7.1(6.0 - 8.3)	2.6(2.5 - 2.6)	10.8(9.0 - 12.9)
**Venezuela**	136.5(117.5 - 158.7)	43.5(35.9 - 52.1)	39.2(33.2 - 45.3)	57(44.4 - 72.8)	4.6(4.1 - 5.1)	6(5.6 - 6.4)	2.5(2.5 - 2.5)	7.8(7 - 8.6)
**Vietnam**	84.2(74.5 - 94.3)	169.3(146.7 - 195.4)	0.9(0.7 - 1.2)	34.8(28.3 - 41.2)	5.3(4.6 - 5.9)	3.7(3.1 - 4.2)	2.2(2.2 - 2.2)	2.7(2.3 - 3.2)
**Yemen**	14.7(12 - 17.9)	65.1(49.4 - 85.2)	7.3(5.9 - 8.6)	16.5(12.6 - 21.5)	7.8(7.6 - 8.1)	13.0(12.4 - 13.7)	2.6(2.6 - 2.7)	23.4(20.2 – 27.0)
**Zambia**	82.7(68.9 - 99.2)	266.8(217.6 - 327.3)	9.6(7.9 - 11.3)	52.0(36.6 - 73)	12.2(11.9 - 12.6)	10.7(10.4 - 10.9)	2.6(2.6 - 2.6)	26.1(23.2 - 29.3)
**Zimbabwe**	81.4(67.9 - 97.3)	48.4(34.4 - 68)	12.2(11.9 - 12.5)	14.0(13.7 - 14.3)	2.6(2.6 - 2.6)	18.4(16.3 - 20.7)	2.6(2.6 - 2.6)	18.4(16.3 - 20.7)

Supplementary Table 7: National mean (95% UI) intakes of eight nutrients among males in 1990 across 185 countries and regions.

Country/Region	Fruits	Non-starchy vegetables	Total processed meats	Unprocessed red meats	Saturated fat	Monounsaturated fatty acids	Total omega-6 fat	Dietary fiber
**Afghanistan**	75.2(64.2 - 88.5)	160.5(124.9 - 205.2)	2.0(1.8 - 2.3)	35.7(28.6 - 44.6)	7.8(7.2 - 8.5)	4.7(4.4 – 5.0)	2.6(2.6 - 2.6)	62.2(56.1 – 69.0)
**Albania**	16.2(14.2 - 18.5)	77.7(61.8 - 97.3)	19.5(16.4 - 23.2)	6.1(4.8 - 7.8)	9.5(9.1 - 9.9)	11.8(11.3 - 12.4)	2.5(2.4 - 2.5)	6.2(5.6 - 6.9)
**Algeria**	67.4(54.7 - 82.8)	148.7(113.6 - 195.3)	7.2(5.8 - 8.5)	22.6(17.2 - 29.7)	13.3(13 - 13.6)	19(17.9 - 20)	2.6(2.6 - 2.6)	16.3(14 - 18.9)
**Angola**	44.7(37.5 - 53.4)	144.6(118 - 177.5)	2.8(2.3 - 3.3)	25.7(18.3 - 36.4)	12.7(12.4 - 13)	10.3(10.1 - 10.5)	2.6(2.6 - 2.6)	17.3(15.4 - 19.5)
**Antigua and Barbuda**	85.5(73.5 - 99.4)	203(167.2 - 245.3)	28(22.5 - 33.3)	33.4(25.7 - 43.3)	11.4(10.6 - 12.3)	8.7(8.2 - 9.2)	2.7(2.6 - 2.7)	19(17.1 - 21)
**Argentina**	90.4(77.8 - 104.9)	128.8(107.2 - 155.4)	10.5(8.7 - 12.3)	78.1(61 - 99.9)	11(10.1 - 11.8)	10.7(10.2 - 11.3)	2.6(2.6 - 2.7)	8(7.2 - 8.8)
**Armenia**	33.1(28.9 - 37.8)	97(76.6 - 122.2)	18.7(15.8 - 22.2)	22(17.2 - 27.8)	12.9(12.5 - 13.4)	20(18.9 - 21)	2.5(2.5 - 2.6)	6.7(6.1 - 7.4)
**Australia**	138.8(125.6 - 153.3)	115.6(97.2 - 137.1)	8.2(7 - 9.6)	63.1(50.8 - 78.2)	13.4(13 - 13.8)	11(10.9 - 11.2)	2.6(2.6 - 2.6)	17.5(15.4 - 19.9)
**Austria**	92.8(84.1 - 102.6)	82.1(69.2 - 96.8)	42.1(35.7 - 49.5)	103.2(83.4 - 127.7)	17.1(16.7 - 17.5)	13.1(12.9 - 13.2)	2.8(2.8 - 2.8)	19.8(17.5 - 22.3)
**Azerbaijan**	90.1(78.5 - 103.2)	60.4(48.1 - 76.2)	73.4(63.6 - 84.8)	33.3(26.2 - 42.4)	12.9(12.4 - 13.3)	31.2(30.2 - 32.3)	2.5(2.5 - 2.6)	6.1(5.5 - 6.8)
**Bahrain**	100.4(81.9 - 123.2)	198.4(152.3 - 259.9)	11.4(9.2 - 13.5)	34.5(26.4 - 44.8)	10.0(9.8 - 10.3)	11.6(11 - 12.3)	2.7(2.7 - 2.7)	7.2(6.2 - 8.4)
**Bangladesh**	47.9(40.8 - 56.3)	224.8(175.9 - 290.3)	0.4(0.4 - 0.5)	6.2(5 - 7.7)	2.3(2.0 - 2.6)	1.6(1.5 - 1.7)	2.3(2.3 - 2.3)	17.8(16 - 19.8)
**Barbados**	118.9(102.1 - 138.3)	143(118 - 174.2)	10.9(9 - 12.8)	222.7(178.1 - 277.3)	10.2(9.4 - 11)	8.3(7.9 - 8.8)	2.8(2.8 - 2.8)	37.5(33.8 - 41.5)
**Belarus**	76.9(66.9 - 87.9)	84.0(66.8 - 105)	22.0(18.4 - 26.3)	66.6(52.5 - 84.8)	13.2(12.7 - 13.7)	11.2(10.6 - 11.7)	2.5(2.5 - 2.6)	6.1(5.5 - 6.7)
**Belgium**	97.1(87.7 - 107.4)	87.8(74.6 - 103.4)	23.8(20.4 - 28)	57.4(46.3 - 70.8)	16.2(15.8 - 16.6)	13.7(13.5 - 13.9)	2.7(2.7 - 2.7)	19.5(17.3 - 22.1)
**Belize**	107.1(92.2 - 124.5)	88.3(73.1 - 106.4)	27(22.6 - 31.5)	67.2(52.5 - 86.2)	9.9(9.1 - 10.7)	10.6(10 - 11.1)	2.5(2.5 - 2.5)	8.7(7.8 - 9.6)
**Benin**	57.6(48.2 - 69.1)	100.7(82.1 - 123.1)	15.1(12.4 - 17.7)	49.2(34.8 - 69)	12.6(12.3 - 12.9)	16.3(15.9 - 16.6)	2.6(2.6 - 2.6)	7.8(6.9 - 8.8)
**Bhutan**	78.4(66.8 - 91.3)	275.1(213.7 - 353)	1.5(1.4 - 1.7)	20.7(16.5 - 25.8)	6.3(5.7 - 6.8)	5.7(5.4 - 6)	2.6(2.5 - 2.6)	22.6(20.3 - 25.3)
**Bolivia**	85.5(73.4 - 99.5)	111.6(92.3 - 134.9)	6.2(5.1 - 7.3)	70.9(55.4 - 90.9)	11.5(10.7 - 12.4)	11.2(10.7 - 11.8)	2.6(2.5 - 2.6)	6.1(5.5 - 6.7)
**Bosnia and Herzegovina**	36.5(31.7 - 41.7)	127.3(100.3 - 160.2)	6.8(5.7 - 8.1)	27.4(21.7 - 34.8)	12(11.6 - 12.5)	23.2(22.1 - 24.4)	2.5(2.5 - 2.6)	4.3(3.9 - 4.7)
**Botswana**	45.9(37.9 - 55.2)	116.9(95.2 - 143.5)	14(11.5 - 16.5)	51.3(36.5 - 71.3)	10.5(10.2 - 10.8)	11.2(11 - 11.4)	2.6(2.6 - 2.6)	51.7(46.2 - 57.6)
**Brazil**	78.9(67.8 - 91.7)	96(79.5 - 115.9)	10.9(9.1 - 12.8)	34(26.4 - 43.6)	8.3(7.6 - 9.1)	11.8(11.3 - 12.4)	2.7(2.7 - 2.7)	13.4(12.1 - 14.8)
**Brunei**	80.9(71.3 - 90.6)	146.5(126.1 - 169.3)	32.4(25.1 - 41.8)	79.7(65.3 - 94.5)	11.6(10.6 - 12.6)	8.5(7.6 - 9.5)	2.5(2.5 - 2.5)	12.3(10.3 - 14.6)
**Bulgaria**	79.2(69.2 - 90.8)	441.2(366.9 - 527.6)	21.5(17.9 - 25.7)	95.1(75.1 - 120.3)	12.8(12.4 - 13.3)	9.2(8.6 - 9.8)	3.0(3.0 - 3.1)	21.0(19.0 - 23.2)
**Burkina Faso**	33.7(28.1 - 40.5)	72.3(58.7 – 89.0)	1.8(1.5 - 2.1)	9.0(6.4 - 12.7)	12.4(12 - 12.7)	17.1(16.7 - 17.5)	2.6(2.6 - 2.6)	11.8(10.5 - 13.3)

**Burundi**	77.3(64.5 - 92.3)	204.3(166.4 - 251.5)	1.8(1.5 - 2.2)	17.2(12.1 - 24.3)	11.4(11.1 - 11.7)	13.1(12.8 - 13.4)	2.6(2.5 - 2.6)	27.6(24.5 - 31)
**Cambodia**	47.9(42.0 - 53.5)	126.8(109.2 - 146.6)	6.1(4.5 – 8.0)	22.4(18.3 - 26.6)	12.6(11.6 - 13.7)	6.9(6.1 - 7.8)	2.6(2.6 - 2.6)	5(4.2 - 5.9)
**Cameroon**	75.6(63.5 - 90)	136.2(110.6 - 167.5)	5.2(4.3 - 6.2)	47.9(33.9 - 67.5)	5.1(4.9 - 5.2)	16.1(15.9 - 16.5)	2.6(2.5 - 2.6)	20.4(18.1 - 23)
**Canada**	88.6(80.3 - 97.6)	135.5(114.4 - 159.9)	11.8(10 - 13.8)	42.7(34.2 - 52.6)	11.8(11.4 - 12.1)	11.9(11.8 - 12.1)	2.5(2.5 - 2.5)	12.1(10.7 - 13.7)
**Cape Verde**	71.4(59.8 - 85.8)	52.8(43.1 - 64.6)	4.1(3.3 - 4.8)	21.5(15.2 - 30.1)	10.6(10.3 - 10.9)	12.2(12 - 12.5)	2.6(2.6 - 2.7)	60.3(54.2 - 67.1)
**Central African Republic**	77.3(64.7 - 92.6)	125.5(102.6 - 154.1)	3.4(2.8 - 4.1)	92.5(65.9 - 127.9)	9.9(9.6 - 10.2)	9.6(9.4 - 9.8)	2.7(2.7 - 2.7)	20.6(18.2 - 23.2)
**Chad**	229.5(191.2 - 275.5)	138.2(113.1 - 169)	3.9(3.2 - 4.7)	55.8(39.3 - 78.3)	12.5(12.1 - 12.8)	10.2(10 - 10.4)	2.6(2.6 - 2.6)	15.6(13.9 - 17.5)
**Chile**	101(86.9 - 117.7)	139.4(115.5 - 168.6)	18.5(15.4 - 21.7)	14.4(11.1 - 18.4)	9.7(9 - 10.5)	9.5(9.1 - 10)	3.2(3.1 - 3.2)	15.8(14.2 - 17.4)
**China**	76.2(67.5 - 85.5)	27.8(24.1 - 32.2)	3.3(2.5 - 4.4)	21.6(17.4 - 25.8)	7.1(6.3 - 7.8)	6.8(6 - 7.5)	2.8(2.8 - 2.9)	4.5(3.8 - 5.3)
**Colombia**	92.2(79.3 - 107)	99.4(82.2 - 120.7)	65.6(54.5 - 76.5)	112.1(86.8 - 143.9)	8.4(7.7 - 9.1)	13.4(12.8 - 14)	2.6(2.5 - 2.6)	18.4(16.7 - 20.3)
**Comoros**	82.6(68.7 - 99)	101.7(82.7 - 124.7)	2.3(1.9 - 2.7)	36.2(25.6 - 50.9)	11.7(11.4 - 12)	13.4(13.1 - 13.7)	2.6(2.6 - 2.6)	19(16.9 - 21.4)
**Congo**	85.2(71.3 - 101.3)	150.9(122 - 185.5)	8.7(7.1 - 10.3)	16.2(11.5 - 22.9)	8.8(8.5 - 9.1)	13.2(12.9 - 13.4)	2.5(2.5 - 2.5)	24(21.3 - 27)
**Costa Rica**	110.7(95.2 - 128.8)	81.4(67.4 - 98.3)	30.5(25.6 - 35.5)	63.4(49.2 - 81.1)	10.7(9.9 - 11.6)	9.4(9 - 9.9)	2.5(2.5 - 2.6)	13.4(12.1 - 14.9)
**Cote d'Ivoire**	57.6(48.2 - 68.6)	98.5(80.5 - 121)	3.5(2.9 - 4.1)	25.4(18 - 36.3)	9.4(9.2 - 9.7)	13.7(13.4 - 14)	2.5(2.5 - 2.5)	21.8(19.3 - 24.5)
**Croatia**	48.2(41.9 - 55.3)	79.4(63.1 - 99.4)	24(20.2 - 28.6)	62.5(49 - 79.1)	12.1(11.6 - 12.5)	11.7(11.1 - 12.3)	2.5(2.5 - 2.6)	27.3(24.6 - 30.3)
**Cuba**	67.7(58.1 - 78.7)	31.5(26.2 - 37.9)	39.2(33.3 - 45.2)	92.5(72.2 - 118.3)	10(9.3 - 10.8)	21(20.1 - 22)	2.7(2.6 - 2.7)	2.9(2.6 - 3.2)
**Cyprus**	189.9(172.2 - 209.3)	168.1(142.2 - 199.2)	6.7(5.7 - 7.9)	65(52.8 - 80.4)	12.4(12.1 - 12.8)	12.2(12 - 12.4)	2.7(2.7 - 2.7)	10.6(9.3 - 11.9)
**Czech Republic**	58.8(51.3 - 67.3)	104.2(82.7 - 130.7)	30.5(25.6 - 36.3)	88.7(70 - 111.5)	14.4(13.9 - 14.9)	10(9.4 - 10.6)	2.5(2.5 - 2.6)	25(22.6 - 27.7)
**Democratic Republic of the Congo**	91.6(76.1 - 110)	231.8(188.1 - 284.2)	9.3(7.6 - 11)	8.7(6.1 - 12.3)	18.1(17.7 - 18.5)	16.6(16.3 - 16.9)	2.6(2.6 - 2.6)	11.4(10.1 - 12.8)
**Denmark**	85.9(77.5 - 95.3)	78.8(66.7 - 92.8)	24.2(20.6 - 28.4)	71.7(57.8 - 88.9)	15.6(15.2 - 16)	15(14.8 - 15.2)	2.6(2.6 - 2.6)	15.5(13.7 - 17.5)
**Djibouti**	33.2(27.6 - 39.7)	107.1(87.1 - 131.1)	5.1(4.2 - 6)	24.5(17.4 - 34.5)	13(12.7 - 13.4)	14.2(13.9 - 14.5)	2.7(2.7 - 2.7)	63.2(57 - 70.2)
**Dominica**	92.9(79.8 - 107.4)	104.9(86.7 - 126.7)	23.1(19.3 - 26.9)	37.6(29.4 - 47.8)	11.1(10.3 - 12)	12.9(12.3 - 13.6)	2.5(2.5 - 2.5)	12.6(11.4 - 13.9)
**Dominican Republic**	95(81.5 - 110.1)	16.6(13.8 - 20)	16.5(13.7 - 19.2)	44.3(34.4 - 57)	9.5(8.7 - 10.3)	8.4(8 - 8.8)	2.7(2.7 - 2.8)	9.6(8.6 - 10.6)
**Ecuador**	110.7(95.3 - 128.6)	111.3(92.5 - 134.5)	2.7(2.3 - 3.2)	47.6(37.3 - 60.9)	11.9(11.1 - 12.8)	7.8(7.4 - 8.2)	2.6(2.6 - 2.6)	14.2(12.8 - 15.7)
**Egypt**	90.2(73.6 - 110.6)	175.2(133.4 - 231.1)	3(2.4 - 3.5)	15.3(11.7 - 20.2)	7.3(7.1 - 7.5)	16.5(15.7 - 17.4)	2.6(2.6 - 2.7)	21.3(18.4 - 24.8)
**El Salvador**	97.5(83.7 - 113.4)	69.8(58.1 - 84.3)	21.8(18.2 - 25.5)	20(15.7 - 25.7)	7.9(7.2 - 8.6)	10.4(10 - 10.9)	2.6(2.6 - 2.6)	8.7(7.8 - 9.6)
**Equatorial Guinea**	71.9(60 - 86.5)	152.2(124.1 - 184.9)	3.6(3 - 4.3)	30.6(21.7 - 42.3)	11(10.7 - 11.3)	11.4(11.2 - 11.7)	2.6(2.6 - 2.6)	21.7(19.3 - 24.5)
**Eritrea**	55.7(46.7 - 66.6)	72.3(58.7 - 88.5)	1.8(1.5 - 2.1)	28.9(20.6 - 40.9)	11.6(11.3 - 11.9)	13.3(13 - 13.6)	2.6(2.6 - 2.6)	24.6(21.8 - 27.8)
**Estonia**	79.7(69.5 - 91)	118.2(93.6 - 149.1)	77.7(64.8 - 93.1)	92.3(72.8 - 117)	13(12.6 - 13.5)	12.2(11.5 - 12.9)	2.6(2.5 - 2.6)	15.1(13.6 - 16.7)
**Ethiopia**	38.2(31.9 - 45.7)	94.9(77.2 - 116.2)	4.1(3.4 - 4.9)	17(12 - 24.1)	12.3(12 - 12.7)	33.8(33.4 - 34.2)	2.6(2.6 - 2.6)	26.1(23.1 - 29.4)

**Federated States of Micronesia**	101.0(90.3 - 112.9)	188.0(161.5 - 218.8)	10.1(7.5 - 13.5)	17.0(13.3 - 20.6)	14.0(13.1 - 15)	7.7(6.5 - 8.9)	2.6(2.6 - 2.6)	6.9(5.7 - 8.2)
**Fiji**	103.8(91.7 - 116.1)	168.5(145.8 - 194.5)	15.2(11.4 - 20.1)	50.9(41.1 - 61)	17.6(16.4 - 18.8)	10.9(9.7 - 12)	2.6(2.6 - 2.6)	4.8(4 - 5.7)
**Finland**	91(82.6 - 100.3)	90.3(76.3 - 107.1)	29.9(25.4 - 35.3)	43.9(35.5 - 54.4)	15.2(14.8 - 15.6)	11.7(11.5 - 11.9)	2.5(2.5 - 2.5)	18.8(16.6 - 21.2)
**France**	50.9(45.9 - 56.3)	145.3(123.8 - 170.8)	20(16.9 - 23.6)	42.9(34.7 - 53.1)	18.8(18.3 - 19.2)	15.4(15.2 - 15.6)	2.7(2.6 - 2.7)	15.8(14 - 17.9)
**Gabon**	90.6(75.6 - 108.7)	131.2(106.5 - 161.9)	6.1(5 - 7.2)	59.8(42.4 - 84.2)	13.0(12.7 - 13.3)	13.4(13.1 - 13.7)	2.7(2.7 - 2.7)	43.5(38.8 - 48.7)
**Georgia**	61.3(53.6 - 69.9)	85.2(67 - 107)	30.2(25.4 - 35.9)	19.9(15.6 - 25.3)	12.9(12.4 - 13.4)	19.2(18.2 - 20.1)	2.5(2.5 - 2.6)	9.6(8.7 - 10.6)
**Germany**	139.2(126.1 - 153.9)	95.6(80.8 - 113)	48.6(41.3 - 57.1)	50.9(40.7 - 63.2)	15.9(15.5 - 16.3)	12.4(12.2 - 12.5)	2.7(2.6 - 2.7)	60.9(54 - 69)
**Ghana**	79.1(66.4 - 94.2)	146.3(119 - 179.7)	1(0.8 - 1.2)	41.3(29.2 - 58.5)	9.6(9.4 - 9.9)	7.9(7.7 - 8.1)	2.6(2.6 - 2.6)	8.8(7.8 - 9.9)
**Greece**	128.6(116.3 - 141.9)	123.3(104.1 - 145.4)	3.4(2.9 - 4)	61.3(49.1 - 76)	14.2(13.8 - 14.6)	12.2(12.1 - 12.4)	2.7(2.7 - 2.7)	21.8(19.2 - 24.7)
**Grenada**	130.1(111.7 - 150.8)	118.7(98.8 - 143.3)	6.2(5.1 - 7.3)	22.3(17.3 - 28.3)	11.9(11.1 - 12.8)	6.3(6 - 6.7)	2.6(2.5 - 2.6)	14.4(13 - 15.9)
**Guatemala**	57.5(49.5 - 66.6)	91.7(76 - 110.6)	21.9(18.3 - 25.5)	19.5(15.2 - 25)	4.1(3.7 - 4.5)	7.2(6.9 - 7.6)	2.7(2.6 - 2.7)	18(16.3 - 19.9)
**Guinea**	65.3(54.4 - 78.6)	125.6(101.9 - 155.1)	3.7(3 - 4.3)	34.1(24.3 - 47.4)	12.6(12.3 - 12.8)	10.3(10.1 - 10.5)	2.6(2.6 - 2.6)	17.1(15.2 - 19.2)
**Guinea-Bissau**	50.9(42.6 - 61)	21.4(17.3 - 26.4)	15.1(12.5 - 17.7)	3.6(2.5 - 5)	8.7(8.4 - 9)	9.8(9.6 - 9.9)	2.5(2.4 - 2.5)	20.8(18.4 - 23.4)
**Guyana**	99.8(85.7 - 115.4)	89.2(73.5 - 107.5)	71.6(63.3 - 79.8)	41.8(32.6 - 53.5)	7.9(7.3 - 8.6)	9.9(9.5 - 10.3)	2.3(2.2 - 2.3)	2.9(2.6 - 3.2)
**Haiti**	69.6(60 - 80.8)	85.7(70.7 - 103.5)	8.9(7.3 - 10.4)	15.1(11.8 - 19.4)	9.8(9 - 10.6)	19.2(18.2 - 20.3)	2.6(2.5 - 2.6)	12.9(11.7 - 14.3)
**Honduras**	85.4(73.4 - 98.8)	31.1(25.7 - 37.5)	24.3(20.2 - 28.4)	12.7(9.9 - 16.2)	9.1(8.4 - 9.8)	9.5(9.1 - 10)	2.5(2.5 - 2.6)	11.8(10.7 - 13.1)
**Hungary**	58.5(51.1 - 67.2)	69.1(54.8 - 86.8)	18.4(15.5 - 21.7)	30(23.6 - 37.9)	13.7(13.2 - 14.2)	9.8(9.3 - 10.4)	2.8(2.8 - 2.9)	24(21.7 - 26.6)
**Iceland**	70.4(63.6 - 78)	75.7(64.2 - 89.5)	26.5(22.5 - 31.2)	44.7(36.3 - 55.4)	16.7(16.3 - 17.1)	13.6(13.5 - 13.8)	2.4(2.4 - 2.4)	14.1(12.5 – 16.0)
**India**	35.1(29.8 - 41.2)	163.2(126.3 - 209.4)	2.8(2.4 - 3.1)	3.9(3.2 - 4.9)	5.6(5.1 - 6.2)	6.4(6.1 - 6.8)	2.7(2.7 - 2.7)	23.0(20.5 - 25.7)
**Indonesia**	60.9(53.8 - 68)	171.7(148.2 - 199.1)	11.6(8.7 - 15.2)	20.9(16.9 - 24.9)	15.3(14.2 - 16.5)	10.7(9.5 - 11.8)	2.6(2.6 - 2.6)	7.1(5.9 - 8.4)
**Iran**	157(127.6 - 193.4)	194.5(148.4 - 254.9)	3.7(3.0 - 4.4)	28.7(21.8 - 37.7)	9.1(8.8 - 9.3)	10.7(10.1 - 11.4)	2.9(2.8 - 2.9)	7.8(6.8 - 9.1)
**Iraq**	108.7(88.8 - 132.3)	215.8(164.4 - 282.1)	71.5(62.9 - 80.2)	170.7(132.4 - 218.5)	10.4(10.1 - 10.6)	13.3(12.6 - 14)	2.6(2.6 - 2.6)	45.6(39.4 - 52.9)
**Ireland**	74(67.1 - 81.6)	125.4(105.7 - 147.9)	30.3(25.7 - 35.7)	33(26.6 - 40.8)	16.6(16.2 - 17)	13.3(13.2 - 13.5)	2.6(2.6 - 2.6)	15.9(14.1 - 18.1)
**Israel**	188.2(154.1 - 229.6)	138(106.2 - 179.3)	97.6(85.2 - 112.2)	108.4(82.8 - 142.3)	7.7(7.4 - 7.9)	15.7(14.9 - 16.4)	2.6(2.6 - 2.7)	16.3(14.1 - 18.9)
**Italy**	183.5(166.9 - 202.4)	143.5(121.4 - 169.6)	16.3(13.9 - 19.2)	41.4(33.5 - 51.3)	12.6(12.2 - 13)	16.9(16.7 - 17.1)	2.5(2.5 - 2.5)	10.5(9.3 - 11.9)
**Jamaica**	157.4(134.9 - 183.4)	160.6(132.9 - 193.9)	8.3(6.9 - 9.7)	17.9(13.9 - 23)	6.1(5.5 - 6.8)	8.4(8.0 - 8.8)	2.8(2.8 - 2.8)	10.7(9.7 - 11.9)
**Japan**	86.6(76.6 - 97.1)	279.1(241.6 - 322.7)	16.5(12.4 - 21.9)	36(29.1 - 42.8)	7.8(7.1 - 8.6)	10.6(9.5 - 11.8)	2.6(2.6 - 2.7)	13.5(11.3 - 16)
**Jordan**	171.7(140.1 - 210.1)	131.4(101.3 - 170.5)	8.6(7 - 10.2)	50(38 - 65)	6.7(6.5 - 6.9)	4.7(4.3 - 5)	2.6(2.5 - 2.6)	46.3(40 - 53.4)
**Kazakhstan**	48.9(42.6 - 56.2)	120.7(95.8 - 152.3)	30.4(25.4 - 36.2)	101.8(79.5 - 130.1)	13.1(12.6 - 13.6)	12.1(11.5 - 12.7)	2.5(2.5 - 2.6)	13.1(11.8 - 14.5)
**Kenya**	98.7(82 - 118.5)	60.7(49.3 - 74.8)	0.7(0.5 - 0.8)	23.9(16.8 - 33.6)	12.5(12.1 - 12.8)	17.4(17 - 17.8)	2.6(2.6 - 2.6)	23.2(20.6 - 26.1)

**Kiribati**	66.9(59.6 - 75)	114.3(98 - 133.2)	9.9(7.5 - 13.3)	17.3(13.6 - 21)	13.1(12.3 - 14.1)	7.3(6.2 - 8.4)	2.6(2.6 - 2.6)	8.0(6.7 - 9.6)
**Kuwait**	56.1(45.8 - 68.5)	84.3(64.5 - 108.9)	3.2(2.6 - 3.8)	10.9(8.3 - 14.2)	10.1(9.9 - 10.4)	8(7.6 - 8.5)	2.6(2.6 - 2.6)	5.9(5.1 - 6.9)
**Kyrgyzstan**	66.8(58.2 - 76.6)	44.1(34.9 - 55.4)	30.9(26 - 36.9)	87.4(68.8 - 111.6)	13(12.6 - 13.5)	25.3(24.1 - 26.5)	2.5(2.5 - 2.6)	8.8(8.0 - 9.8)
**Laos**	106.7(93.8 - 119.4)	160.6(138.9 - 185.5)	0.8(0.6 - 1.1)	11.0(9 - 13)	12.7(11.7 - 13.7)	7.1(6.3 – 8.0)	2.6(2.6 - 2.6)	8.6(7.3 - 10.2)
**Latvia**	37(32.2 - 42.4)	153.8(121.8 - 194.2)	38.9(32.5 - 46.2)	202.9(160.4 - 255.1)	13.2(12.7 - 13.7)	10.0(9.5 - 10.5)	2.5(2.5 - 2.6)	8.7(7.9 - 9.6)
**Lebanon**	471.5(401.7 - 550.8)	361.6(277.3 - 469.7)	4.2(3.4 - 4.9)	14.3(10.9 - 18.7)	12.0(11.7 - 12.3)	18.4(17.6 - 19.1)	3.1(3.1 - 3.1)	45.2(39 - 52.3)
**Lesotho**	63.1(52.5 - 75.3)	455.6(374.3 - 550.4)	2.7(2.2 - 3.3)	24.8(17.6 - 34.5)	12.4(12.1 - 12.7)	10.1(9.9 - 10.3)	2.6(2.6 - 2.6)	39.9(35.5 - 44.7)
**Liberia**	57.8(48.1 - 69.4)	121.1(98 - 148.5)	15(12.3 - 17.7)	39(27.6 - 54.2)	12.8(12.5 - 13.2)	10.4(10.1 - 10.6)	2.7(2.6 - 2.7)	13.3(11.8 - 14.9)
**Libya**	52.2(42.7 - 63.6)	125.4(95.5 - 164)	9.5(7.7 - 11.1)	33.2(25.5 - 43.4)	10.4(10.2 - 10.7)	10.8(10.2 - 11.3)	2.5(2.5 - 2.6)	50.3(43.5 - 58.1)
**Lithuania**	49.6(43.4 - 56.7)	74.6(59.3 - 93.5)	23.4(19.6 - 27.6)	138.1(108.1 - 175.9)	12(11.5 - 12.4)	12.9(12.2 - 13.6)	2.5(2.5 - 2.6)	3.9(3.6 - 4.3)
**Luxembourg**	80.8(73.2 - 89.3)	113.2(96 - 133.2)	26.5(22.5 - 31.2)	58.9(47.7 - 72.9)	15.9(15.5 - 16.3)	14.4(14.2 - 14.6)	2.7(2.7 - 2.7)	18.5(16.3 - 20.9)
**Macedonia**	51.2(44.7 - 58.6)	148.9(118.1 - 186.4)	25.0(20.9 - 29.9)	27.6(21.7 - 35.2)	12.1(11.7 - 12.6)	11.6(10.9 - 12.2)	2.5(2.5 - 2.6)	22.5(20.4 - 24.9)
**Madagascar**	89.1(74.3 - 106.7)	164.1(133.2 - 200.5)	4.9(4 - 5.8)	41.5(29.4 - 58.5)	12.5(12.2 - 12.8)	10.2(10 - 10.4)	2.6(2.6 - 2.6)	27.7(24.6 - 31.1)
**Malawi**	107.8(90.2 - 129.4)	247(201.2 - 302.5)	2.1(1.8 - 2.5)	19.8(14 - 28)	5.6(5.4 - 5.8)	15(14.7 - 15.3)	2.5(2.5 - 2.5)	10.8(9.5 - 12.1)
**Malaysia**	97.8(86.3 - 109.4)	135.1(116.5 - 156.3)	17.2(12.8 - 23.1)	18(14.7 - 21.4)	11.7(10.7 - 12.7)	9.5(8.4 - 10.6)	2.5(2.5 - 2.5)	19.9(16.7 - 23.7)
**Maldives**	20.6(17.5 - 24.2)	33.3(25.6 - 43.2)	0.8(0.7 - 0.9)	6(4.8 - 7.6)	8.4(7.7 - 9.1)	9(8.5 - 9.6)	2.6(2.6 - 2.6)	12.3(10.9 - 13.7)
**Mali**	70.1(58.7 - 83.7)	97(78.6 - 119.2)	8.1(6.6 - 9.5)	26.9(19.2 - 37.8)	8.5(8.3 - 8.8)	14.6(14.3 - 14.9)	2.6(2.6 - 2.7)	52(46.5 - 58.5)
**Malta**	119.8(108.7 - 132.1)	92.5(77.9 - 109.7)	24.2(20.5 - 28.7)	61.1(49.8 - 75.4)	14.5(14.2 - 14.9)	12(11.8 - 12.2)	2.7(2.7 - 2.7)	13(11.4 - 14.7)
**Marshall Islands**	81.5(72.8 - 91.4)	193.7(166.9 - 224)	7.7(5.8 - 10.3)	38.7(31.2 - 46.1)	14(12.9 - 15)	8(7.1 - 9)	2.6(2.6 - 2.6)	7.5(6.3 - 8.9)
**Mauritania**	19.6(16.4 - 23.3)	68.4(55.4 - 84)	25.3(20.8 - 29.7)	36.6(25.8 - 50.7)	12.7(12.3 - 13)	14.8(14.5 - 15.2)	2.6(2.6 - 2.6)	68.7(62.7 - 75.1)
**Mauritius**	65.8(55.2 - 78.7)	181.3(145.8 - 223.4)	15.5(12.7 - 18.4)	65.5(46.3 - 91.5)	9.6(9.3 - 9.9)	9.6(9.4 - 9.8)	2.7(2.7 - 2.8)	26.6(23.6 - 29.9)
**Mexico**	78(67.2 - 90.5)	92.4(76.2 - 111.6)	18(14.9 - 21.1)	27.6(21.5 - 35.7)	9.8(9.1 - 10.6)	11.5(11 - 12.1)	2.3(2.3 - 2.4)	16.4(14.8 - 18.1)
**Moldova**	57(49.7 - 65.3)	83.1(65.9 - 105)	48.3(41.2 - 56.8)	151.2(118.3 - 192.6)	13.1(12.6 - 13.6)	16.8(15.9 - 17.6)	2.5(2.5 - 2.6)	13(11.8 - 14.4)
**Mongolia**	40.4(35.3 - 46.5)	196(155.1 - 246.9)	61.6(52.6 - 71.9)	111(87.5 - 140.2)	12.5(12 - 12.9)	14.3(13.6 - 15.1)	2.5(2.5 - 2.6)	32.2(29.2 - 35.6)
**Montenegro**	47(41 - 53.8)	124.3(98.3 - 156.5)	26.2(22 - 31.1)	70.8(55.5 - 90.4)	12.1(11.6 - 12.5)	8.3(7.8 - 8.7)	2.5(2.5 - 2.6)	32.8(29.7 - 36.2)
**Morocco**	53.6(43.7 - 65.6)	183.1(139.1 - 238.2)	8.7(7 - 10.3)	17.9(13.4 - 23.4)	7.1(6.9 - 7.3)	13.3(12.5 - 14)	2.5(2.4 - 2.5)	51.3(44.1 - 59.3)
**Mozambique**	94.7(79 - 113.2)	151.5(122.9 - 186.7)	1.1(0.9 - 1.3)	7.4(5.2 - 10.4)	9.8(9.5 - 10.1)	6.5(6.3 - 6.6)	2.5(2.5 - 2.6)	28.7(25.4 - 32.4)
**Myanmar**	75.4(66.7 - 84.1)	104.7(90.8 - 121)	3.1(2.3 - 4.1)	3.8(3.1 - 4.5)	12.7(11.7 - 13.8)	7.1(6.3 - 8)	2.6(2.6 - 2.6)	6(5.1 - 7.2)
**Namibia**	90.2(74.9 - 107.9)	149.0(121.8 - 183.5)	11.7(9.6 - 13.9)	53.8(38.5 - 75)	12.6(12.3 - 12.9)	18.0(17.7 - 18.4)	2.6(2.6 - 2.6)	29.2(25.9 - 32.7)
**Nepal**	13.1(11.2 - 15.3)	185.4(144.5 - 236.5)	0.5(0.5 - 0.6)	10.1(8.1 - 12.6)	3.8(3.4 - 4.3)	5.4(5.1 - 5.7)	2.3(2.3 - 2.3)	15(13.4 - 16.8)

**Netherlands**	101.3(91.7 - 111.7)	114.7(97.3 - 135.1)	17.9(15.2 - 21.2)	32.3(26 - 40)	14.7(14.3 - 15.1)	11.1(11 - 11.3)	2.7(2.7 - 2.7)	17.5(15.5 - 19.8)
**New Zealand**	143.6(130 - 158.3)	139.2(116.9 - 164.4)	26.7(22.8 - 31.3)	60.9(49.3 - 75.5)	18.9(18.5 - 19.3)	14.9(14.7 - 15.1)	2.7(2.7 - 2.7)	22.8(20.1 - 25.7)
**Nicaragua**	103.7(89.2 - 120.2)	117.7(97.4 - 142.6)	20.2(16.8 - 23.7)	69.5(54.1 - 89.5)	10.3(9.6 - 11.2)	15(14.2 - 15.8)	2.6(2.5 - 2.6)	8.9(8 - 9.8)
**Niger**	61.9(51.8 - 74)	112.9(91.9 - 138.3)	5.1(4.1 - 6)	12.6(8.9 - 17.7)	12.3(12 - 12.7)	15.6(15.2 - 15.9)	2.6(2.6 - 2.6)	21.2(18.8 - 23.8)
**Nigeria**	33.4(27.7 - 39.9)	102.5(83.3 - 125.7)	7.4(6.1 - 8.8)	33.6(23.9 - 47.6)	9.8(9.5 - 10.1)	12.9(12.6 - 13.1)	2.6(2.6 - 2.6)	10.4(9.3 - 11.7)
**Norway**	76.9(69.8 - 85.1)	83.2(70.2 - 98.6)	18(15.3 - 21.3)	57.7(46.7 - 71.6)	14(13.6 - 14.4)	11.3(11.1 - 11.4)	2.6(2.6 - 2.7)	21.6(19.1 - 24.4)
**Oman**	95.0(77.5 - 116.5)	175.2(133.3 - 230)	9.0(7.4 - 10.7)	24.4(18.7 - 31.5)	9.8(9.6 - 10.1)	11.5(10.8 - 12.1)	2.7(2.7 - 2.7)	22.5(19.4 - 26.1)
**Pakistan**	32.5(27.8 - 38.1)	87.4(68.1 - 111.9)	4.0(3.5 - 4.6)	21.3(17.1 - 26.6)	7.6(7.0 - 8.2)	5.0(4.8 - 5.3)	2.6(2.6 - 2.6)	14.8(13.3 - 16.6)
**Palestine**	93.8(76.8 - 114.3)	173.2(132.8 - 226.9)	10.1(8.2 - 11.9)	33.0(25.1 – 43.0)	12.4(12.1 - 12.7)	6.4(6.1 - 6.8)	2.7(2.7 - 2.7)	24.2(20.9 - 28.1)
**Panama**	113.5(97.4 - 132.2)	91(75.5 - 109.6)	34.3(28.8 - 39.8)	67(52.4 - 85.5)	9.5(8.8 - 10.3)	12.3(11.7 - 13)	2.7(2.7 - 2.7)	8.1(7.4 – 9.0)
**Papua New Guinea**	47.8(42.1 - 53.3)	87.6(75 - 102.5)	6.4(4.8 - 8.6)	31.4(24.7 - 38.3)	13.8(12.9 - 14.7)	7.3(6.1 - 8.4)	2.6(2.6 - 2.6)	12.8(10.7 - 15.4)
**Paraguay**	157.3(135.5 - 182.9)	78.5(64.6 - 94.6)	29.1(24.5 - 33.6)	70.3(54.8 - 90.1)	8.8(8.1 - 9.5)	8.5(8.1 - 8.9)	2.6(2.6 - 2.7)	12.9(11.7 - 14.3)
**Peru**	64.6(55.4 - 75.2)	103.1(85.3 - 124.3)	22.1(18.4 - 25.7)	80.9(62.5 - 103.7)	7.5(6.9 - 8.2)	16.8(16.1 - 17.6)	2.6(2.6 - 2.6)	2.6(2.4 - 2.9)
**Philippines**	104.5(92.3 - 116.8)	112.5(96.8 - 130.3)	11.7(8.8 - 15.6)	19(15.5 - 22.7)	21.8(20.4 - 23.1)	9.1(8.2 - 10)	2.4(2.4 - 2.4)	6.2(5.2 - 7.3)
**Poland**	156.2(135.8 - 178.8)	158(125.5 - 198.9)	30.9(25.9 - 36.7)	63.2(49.8 - 80.1)	13.7(13.2 - 14.1)	16(15.2 - 16.7)	2.5(2.4 - 2.5)	11.5(10.4 - 12.8)
**Portugal**	109.6(98.9 - 121.2)	155.5(130.2 - 185.1)	6.2(5.3 - 7.3)	40.8(32.9 - 50.3)	8.9(8.6 - 9.2)	12.2(12 - 12.4)	2.7(2.7 - 2.7)	12.8(11.3 - 14.5)
**Qatar**	107.2(86.7 - 131.8)	181.3(138.3 - 235.3)	11.4(9.3 - 13.5)	33.7(25.9 - 43.9)	10.0(9.7 - 10.2)	11.5(10.9 - 12.1)	2.7(2.7 - 2.7)	19.1(16.5 - 22.1)
**Romania**	150.3(131.2 - 172.7)	342.2(270.9 - 430.6)	64.9(54.3 - 77.2)	137.2(108.1 - 173.4)	11.5(11.1 – 12.0)	15.1(14.4 - 15.9)	2.2(2.2 - 2.3)	12.2(11.0 - 13.5)
**Russia**	58.2(50.8 - 66.7)	104.5(82.3 - 131.7)	26.4(22.1 - 31.4)	190.5(150.6 - 240.9)	13.2(12.7 - 13.7)	13.5(12.8 - 14.2)	2.5(2.5 - 2.6)	11.5(10.4 - 12.7)
**Rwanda**	113.9(94.6 - 136.3)	305(246.9 - 372.7)	0.9(0.7 - 1)	4.6(3.2 - 6.4)	12.2(11.9 - 12.6)	36(35.7 - 36.4)	2.6(2.5 - 2.6)	7.3(6.5 - 8.3)
**Saint Lucia**	136.7(118.4 - 158.8)	78.5(65.3 - 94.8)	4.1(3.4 - 4.8)	26.3(20.6 - 33.5)	8.6(7.9 - 9.3)	6.6(6.3 – 7.0)	2.4(2.4 - 2.5)	21.3(19.3 - 23.5)
**Saint Vincent and the Grenadines**	56.3(48.5 - 65.4)	50.8(42 - 61.4)	6.7(5.6 - 7.9)	29(22.6 - 36.9)	12.1(11.3 - 13)	8.2(7.8 - 8.6)	2.5(2.5 - 2.6)	5.5(5 - 6.1)
**Samoa**	109.6(98 - 122.7)	382.5(330.3 - 442.7)	7.2(5.4 - 9.6)	52.9(42.7 - 62.8)	27.7(26.3 - 29.1)	7.4(6.5 - 8.3)	2.6(2.6 - 2.6)	9.6(8.1 - 11.4)
**Sao Tome and Principe**	54.7(45.2 - 66)	32.2(25.9 - 40)	5.7(4.5 - 7)	3.4(2.4 - 4.8)	12.7(12.4 - 13)	10.3(10.1 - 10.6)	2.6(2.6 - 2.6)	23.2(20.5 - 26.3)
**Saudi Arabia**	88.7(71.9 - 108.7)	252.1(192.5 - 328.2)	38.7(32.2 - 45.2)	41.3(31.7 - 53.4)	11.7(11.4 - 12)	11.7(11.1 - 12.4)	2.7(2.7 - 2.7)	38.7(33.4 - 44.9)
**Senegal**	49.8(41.6 - 59.5)	81.9(66.9 - 100.3)	16.5(13.5 - 19.4)	13.1(9.4 - 18.4)	7.9(7.7 - 8.2)	10.7(10.5 - 10.9)	2.6(2.6 - 2.6)	37.2(33 - 41.7)
**Serbia**	114.3(99.6 - 130.8)	236.5(188.1 - 296.8)	24.9(20.9 - 29.6)	82.2(64.3 - 104.5)	16.1(15.5 - 16.6)	10.7(10.1 - 11.4)	2.5(2.5 - 2.6)	42.2(38.3 - 46.6)
**Seychelles**	109.8(91.9 - 131)	217(177.1 - 267.2)	7.1(5.8 - 8.4)	23.1(16.1 - 32.3)	11.9(11.6 - 12.2)	13.6(13.3 - 13.8)	2.6(2.6 - 2.7)	20(17.8 - 22.5)
**Sierra Leone**	82.3(68.5 - 99.4)	127.5(104.2 - 156.3)	27.9(23 - 32.6)	15.8(11.2 - 21.9)	12.6(12.2 - 12.9)	10.3(10.1 - 10.5)	2.6(2.6 - 2.6)	21.5(19.1 - 24.1)
**Singapore**	120.6(106.4 - 134.9)	151.9(131.2 - 175.5)	8.8(6.6 - 11.9)	19.4(15.7 - 23.1)	10.3(9.3 - 11.2)	6.7(5.9 - 7.4)	2.6(2.6 - 2.6)	19.3(16.2 - 22.9)

**Slovakia**	52.6(45.8 - 60.5)	96.9(77.1 - 121.9)	20.7(17.4 - 24.6)	45.3(35.6 - 57.4)	14.3(13.8 - 14.8)	10.7(10.1 - 11.3)	2.5(2.5 - 2.6)	42.2(38.3 - 46.4)
**Slovenia**	59(51.5 - 67.7)	107.1(84.7 - 135.6)	14.9(12.4 - 17.8)	42.2(33.3 - 53.5)	11.1(10.6 - 11.5)	10(9.5 - 10.6)	2.5(2.5 - 2.6)	13.3(12 - 14.7)
**Solomon Islands**	50.5(44.6 - 56.5)	190.4(164.7 - 219.1)	13.9(10.4 - 18.5)	34.1(27.8 - 40.4)	12.9(11.9 - 13.9)	7.4(6.5 - 8.3)	2.6(2.6 - 2.6)	3.2(2.7 - 3.8)
**South Africa**	62.3(52 - 74.7)	135.1(109.3 - 166)	12(9.8 - 14.2)	166.1(129.9 - 201.7)	10.1(9.8 - 10.4)	10.7(10.5 - 10.9)	2.7(2.7 - 2.8)	23.2(20.5 - 26.1)
**South Korea**	53.9(47.6 - 60.2)	132.6(114.5 - 153.5)	3(2.3 - 4)	26.7(21.5 - 31.8)	5(4.4 - 5.5)	9.4(8.3 - 10.4)	2.3(2.3 - 2.4)	5.3(4.4 - 6.2)
**South Sudan**	66.7(55.5 - 80.4)	147.4(119.7 - 180.7)	2(1.6 - 2.3)	26.9(19 - 38)	11.5(11.2 - 11.8)	13.2(13 - 13.5)	2.6(2.6 - 2.6)	27.2(24.2 - 30.5)
**Spain**	93.1(84 - 102.9)	103.4(87.5 - 122)	18.6(15.8 - 21.8)	47.1(37.9 - 58.2)	12.6(12.3 - 13)	10.8(10.6 - 10.9)	2.7(2.7 - 2.7)	25.8(22.7 - 29.1)
**Sri Lanka**	69.4(59 - 81.2)	180.5(140.3 - 231.8)	5.7(5 - 6.4)	52.6(42 - 66.2)	10.9(10.2 - 11.7)	10.4(10 - 10.8)	2.3(2.3 - 2.3)	8.4(7.5 - 9.4)
**Sudan**	77.5(64.5 - 93.2)	137.2(111.2 - 169)	2.9(2.4 - 3.4)	29.5(20.6 - 41.5)	11.1(10.8 - 11.3)	12.2(12 - 12.5)	2.5(2.5 - 2.5)	28.6(25.3 - 32.2)
**Suriname**	58.9(50.7 - 68.3)	193.5(159.5 - 233)	26(21.8 - 30.1)	39.1(30.3 - 49.8)	7.7(7 - 8.3)	9.2(8.8 - 9.6)	2.5(2.5 - 2.6)	13.8(12.5 - 15.3)
**Swaziland**	87.1(71.9 - 104.9)	149.8(120.8 - 185.4)	7.8(6.2 - 9.4)	26.8(18.9 - 37.9)	8.9(8.7 - 9.2)	9.4(9.3 - 9.6)	2.5(2.5 - 2.5)	27.6(24.4 - 31.3)
**Sweden**	93.2(84.3 - 102.8)	87(73.5 - 102.8)	17.2(14.6 - 20.2)	54.9(44 - 68.4)	15.3(14.9 - 15.7)	20.9(20.8 - 21.1)	2.5(2.5 - 2.6)	13.5(11.9 - 15.2)
**Switzerland**	141(127.7 - 155.9)	112.2(95 - 132.4)	15.2(13 - 17.8)	58.3(47.1 - 71.9)	14.9(14.5 - 15.2)	14.2(14 - 14.3)	2.6(2.6 - 2.6)	15.4(13.6 - 17.5)
**Syria**	91(74 - 111.4)	151.1(115.4 - 197.6)	6.9(5.5 - 8.2)	31.6(24 - 41.6)	13.5(13.2 - 13.8)	8.3(7.8 - 8.9)	2.7(2.7 - 2.8)	25.8(22.4 - 29.8)
**Taiwan**	124.4(109.6 - 138.5)	254.9(219.9 - 294.7)	13.7(10.3 - 18.4)	48.0(39.2 - 57)	7.9(7.1 - 8.7)	7.0(6.3 - 7.8)	2.8(2.7 - 2.8)	5.9(5.0 – 7.0)
**Tajikistan**	72.6(63.5 - 83.2)	65.7(51.9 - 82.6)	83.8(73.2 - 96.4)	25.1(19.7 - 31.8)	13(12.5 - 13.5)	27.7(26.5 - 28.9)	2.5(2.5 - 2.6)	10.8(9.7 - 12)
**Tanzania**	69.4(58.1 - 82.9)	179.3(145.2 - 221.5)	1.8(1.5 - 2.1)	46.5(33.2 - 64.5)	12.5(12.1 - 12.8)	10.2(10 - 10.4)	2.6(2.6 - 2.6)	12.9(11.4 - 14.5)
**Thailand**	80.5(70.9 - 90.2)	177.5(153.8 - 205.3)	7.5(5.6 - 9.9)	27.4(22.2 - 32.7)	11.3(10.3 - 12.3)	10.3(9.2 - 11.3)	2.4(2.4 - 2.4)	2.9(2.4 - 3.4)
**The Bahamas**	65.7(56.3 - 76.5)	106.5(88 - 128.8)	12.1(10.1 - 14.2)	88.1(68.7 - 112.6)	11.2(10.4 - 12.1)	6.9(6.6 - 7.3)	2.5(2.5 - 2.6)	13.5(12.2 - 14.9)
**The Gambia**	12(10 - 14.4)	62.1(50.2 - 77.2)	13.5(10.8 - 16.3)	18.3(12.9 - 25.6)	12.6(12.3 - 12.9)	10.3(10.1 - 10.5)	2.6(2.6 - 2.6)	41.9(37.1 - 47.2)
**Timor-Leste**	47.3(42.3 - 52.8)	157.2(135.8 - 182.2)	7.2(5.4 - 9.6)	24.2(19.7 - 28.8)	12.9(11.8 - 13.9)	7.3(6.4 - 8.2)	2.6(2.6 - 2.6)	6.1(5.1 - 7.2)
**Togo**	57.1(47.8 - 68.3)	179.3(146.4 - 219)	12.6(10.3 - 14.8)	26.7(18.9 - 37.3)	12.6(12.3 - 12.9)	10.3(10 - 10.5)	2.6(2.6 - 2.6)	14(12.4 - 15.8)
**Tonga**	98.3(87.6 - 109.9)	149(127.8 - 173.7)	7.5(5.6 - 10.2)	35.8(28 - 43.7)	14(13.1 - 15)	7.6(6.4 - 8.8)	2.6(2.6 - 2.6)	5.6(4.6 - 6.7)
**Trinidad and Tobago**	65(56 - 75.5)	63.4(52.4 - 76.8)	14.6(12.2 - 17)	28.7(22.4 - 36.5)	10.7(9.9 - 11.5)	8.8(8.3 - 9.2)	2.6(2.6 - 2.6)	8.4(7.6 - 9.3)
**Tunisia**	57.9(47.2 - 70.5)	122(92.8 - 158.9)	0.5(0.4 - 0.7)	37.4(28.5 - 48.8)	8.9(8.6 - 9.1)	12.4(11.6 - 13.1)	3(3 - 3.1)	26.5(22.9 - 30.7)
**Turkey**	71.4(58 - 87.6)	118.5(91 - 154.6)	2.7(2.2 - 3.2)	39.3(29.8 - 51.2)	9.6(9.3 - 9.8)	10.8(10.1 - 11.4)	2.8(2.8 - 2.8)	19.5(16.8 - 22.6)
**Turkmenistan**	33.2(29 - 38.1)	81.1(64.4 - 102.4)	52(44.1 - 60.9)	38(29.9 - 48.6)	13(12.6 - 13.5)	13.4(12.7 - 14.2)	2.5(2.5 - 2.6)	6.6(6 - 7.3)
**Uganda**	58.6(48.9 - 69.8)	114.6(93.2 - 140.8)	0.6(0.5 - 0.7)	10.5(7.4 - 14.6)	12.4(12.0 - 12.7)	10.1(9.9 - 10.4)	2.6(2.6 - 2.6)	10.4(9.2 - 11.6)
**Ukraine**	63.4(55.4 - 73)	177.5(140.3 - 222.8)	46.8(39.7 - 55)	83.3(65.1 - 106)	13.2(12.7 - 13.7)	13.8(13.1 - 14.6)	2.5(2.5 - 2.6)	7.9(7.2 - 8.8)
**United Arab Emirates**	126.4(102.9 - 154.4)	174.3(133 - 226.1)	46.2(38.9 - 53.5)	107.5(82.6 - 139.6)	9.9(9.6 - 10.1)	8.4(7.8 - 9)	2.4(2.4 - 2.4)	18.4(15.9 - 21.3)

**United Kingdom**	82.8(75.2 - 91.2)	123.3(104.1 - 146.2)	23.3(19.8 - 27.5)	37.9(30.6 - 46.8)	14(13.6 - 14.4)	13.7(13.5 - 13.9)	2.6(2.6 - 2.6)	14.8(13.1 - 16.8)
**United States**	90(81.5 - 99.4)	131.2(111 - 153.9)	16.3(13.9 - 19.3)	33.5(27 - 41.6)	11.4(11.1 - 11.8)	12.7(12.5 - 12.9)	2.7(2.7 - 2.7)	15.3(13.5 - 17.4)
**Uruguay**	88.6(75.7 - 103.2)	46.7(38.7 - 56.4)	26.4(22 - 30.7)	112.7(87.5 - 144.8)	11.1(10.3 - 12)	11.6(11 - 12.1)	2.6(2.6 - 2.6)	11.4(10.3 - 12.6)
**Uzbekistan**	70.9(61.5 - 81.7)	145.5(116.3 - 181.8)	37.2(31.4 - 43.9)	39.4(31 - 50)	13(12.6 - 13.5)	23.6(22.5 - 24.8)	2.5(2.5 - 2.6)	14.7(13.3 - 16.2)
**Vanuatu**	40.1(35.8 - 44.8)	77.8(67.1 - 90.3)	9.0(6.7 - 11.9)	34.4(27.1 - 41.9)	13.1(12.2 - 14)	7.2(6.1 - 8.3)	2.6(2.6 - 2.6)	11(9.2 - 13.1)
**Venezuela (Bolivarian Republic of)**	126.6(108.9 - 147.4)	45.2(37.3 - 54.6)	36.7(31.2 - 42.3)	55.3(42.8 - 71.4)	4.7(4.2 - 5.2)	5.4(5 - 5.7)	2.5(2.5 - 2.5)	7.5(6.8 - 8.3)
**Vietnam**	64.9(57.1 - 72.4)	189(163.9 - 217.8)	0.9(0.7 - 1.2)	37.5(30.3 - 44.4)	5.7(5 - 6.3)	3.8(3.2 - 4.3)	2.2(2.2 - 2.2)	2.8(2.3 - 3.3)
**Yemen**	13.2(10.8 - 16.1)	70.9(54.1 - 92.1)	5.9(4.8 – 7.0)	17.3(13.2 - 22.4)	8.4(8.1 - 8.6)	12.1(11.5 - 12.7)	2.6(2.6 - 2.7)	23.3(20.1 - 26.8)
**Zambia**	84.2(70.2 - 100.8)	263.8(215.1 - 325)	8.4(6.9 - 10)	47.2(33.3 - 66.5)	12.7(12.4 - 13)	10.3(10.1 - 10.6)	2.6(2.6 - 2.6)	26.5(23.6 - 29.8)
**Zimbabwe**	81.8(68.3 - 97.4)	143.7(117 - 175.8)	3.5(2.9 - 4.1)	39.5(28 - 55.6)	12.6(12.3 - 13)	13.5(13.2 - 13.8)	2.6(2.6 - 2.6)	18.7(16.6 - 21.2)

Supplementary Table 8: National mean (95% UI) intakes of eight nutrients in 2018 across 185 countries, stratified by area of residence for all age groups.

Rgion	Age	Fruits	Non-starchy vegetables	Total processed meats	Unprocessed red meats	Saturated fat	Monounsaturated fatty acids	Total omega-6 fat	Dietary fiber
**Asia**	0.5	31.0 (21.2-42.8)	28.1 (19.7-38.1)	7.0 (4.7-9.8)	4.0 (2.7-5.6)	13.7 (9.7-18.5)	10.0 (7-13.5)	2.3 (1.6-3.1)	2.0 (1.3-2.8)
**Asia**	1.5	44.6 (31.1-61.8)	47.6 (33.9-65.2)	12.8 (8.6-18.1)	9.0 (6.1-12.5)	13.2 (9.4-18)	9.2 (6.6-12.3)	2.3 (1.6-3.2)	3.1 (2.1-4.4)
**Asia**	3.5	59.2 (41-82.4)	71.0 (50.3-96.5)	20.1 (13.7-27.8)	17.0 (11.6-23.6)	12.8 (9.1-17.3)	8.7 (6.1-11.8)	2.3 (1.6-3.2)	4.4 (3.1-6.2)
**Asia**	7.5	75.5 (51.9-103.8)	101.7 (71.8-139.1)	28.8 (19.8-40.3)	30.1 (21.0-41.7)	12.4 (8.7-16.8)	8.2 (5.8-11.1)	2.3 (1.6-3.2)	6.1 (4.2-8.7)
**Asia**	12.5	83.3 (57-115.7)	124.2 (87.6-168.7)	30.9 (21.2-43.5)	40.1 (27.8-56.0)	12.2 (8.6-16.5)	7.9 (5.6-10.7)	2.3 (1.6-3.3)	7.6 (5.2-10.7)
**Asia**	17.5	82.2 (56.8-114.4)	135.9 (96.1-186.1)	26.7 (18.4-37.3)	43.2 (30.1-59.8)	12.1 (8.5-16.4)	7.8 (5.5-10.7)	2.3 (1.6-3.2)	8.6 (6.0-12.0)
**Asia**	22.5	76.9 (53.1-106.4)	140.8 (98.3-190.9)	20.6 (14.2-28.8)	42.3 (29.1-58.5)	11.9 (8.4-16.2)	7.7 (5.4-10.4)	2.3 (1.6-3.2)	9.4 (6.5-13.0)
**Asia**	27.5	71.5 (49.1-99.6)	142.2 (100-195.9)	15.4 (10.5-21.7)	39.3 (27.0-54.9)	11.8 (8.4-16.0)	7.6 (5.4-10.4)	2.3 (1.6-3.2)	10.1 (6.9-14.1)
**Asia**	32.5	67.8 (47-93.7)	142.1 (100.4-195.1)	11.7 (8.0-16.4)	36.2 (25.1-50.2)	11.6 (8.2-15.8)	7.5 (5.3-10.1)	2.3 (1.6-3.2)	10.7 (7.4-14.9)
**Asia**	37.5	66.4 (45.6-91.8)	141.0 (98.8-191.0)	9.4 (6.3-13.4)	32.9 (22.8-45.0)	11.4 (8.0-15.7)	7.2 (5.1-9.9)	2.3 (1.6-3.2)	11.1 (7.7-15.4)
**Asia**	42.5	66.1 (45.4-92)	142.3 (100-196.3)	7.9 (5.3-11.2)	30.4 (21.0-42.0)	11.1 (7.7-15.1)	7.0 (5.0-9.5)	2.3 (1.6-3.2)	11.4 (7.9-15.8)
**Asia**	47.5	67.6 (46.5-92.8)	142.3 (99-196.7)	6.9 (4.7-9.9)	28.3 (19.6-39.4)	10.8 (7.6-14.8)	6.7 (4.7-9.0)	2.3 (1.6-3.2)	11.6 (8.1-16.1)
**Asia**	52.5	70.7 (48.6-97.6)	143.0 (101.6-194.7)	6.3 (4.2-8.9)	26.2 (18.2-36.4)	10.4 (7.4-14.1)	6.4 (4.4-8.7)	2.3 (1.6-3.2)	11.7 (8.0-16.3)
**Asia**	57.5	75.1 (51.2-104.2)	143.2 (101.6-197.6)	6.0 (4.0-8.5)	24.6 (16.9-34.5)	10.1 (7.2-13.8)	6.0 (4.3-8.2)	2.3 (1.5-3.2)	11.7 (8.0-16.1)
**Asia**	62.5	79.7 (54.7-110.5)	144.2 (102.3-196.9)	5.7 (3.8-8.1)	23.2 (16.0-32.0)	9.7 (6.9-13.2)	5.7 (4.0-7.8)	2.3 (1.6-3.1)	11.7 (8.1-16.3)
**Asia**	67.5	84.9 (58.4-117.3)	144.9 (103-198.4)	5.6 (3.7-8.0)	21.8 (15.1-30.3)	9.4 (6.6-13.0)	5.4 (3.8-7.4)	2.2 (1.6-3.1)	11.8 (8.2-16.2)
**Asia**	72.5	90.7 (62.6-125.5)	146.3 (102.6-200)	5.4 (3.6-7.6)	20.7 (14.1-29)	9.2 (6.5-12.4)	5.1 (3.6-7.0)	2.3 (1.5-3.2)	11.7 (8.1-16.2)
**Asia**	77.5	95.7 (66.2-133.2)	146.2 (104-199.4)	5.2 (3.4-7.5)	19.8 (13.8-27.2)	8.9 (6.2-12.3)	4.9 (3.4-6.8)	2.2 (1.5-3.1)	11.8 (8.1-16.2)
**Asia**	82.5	100.6 (69.8-138.7)	147.3 (103.6-201.8)	5.1 (3.4-7.4)	18.7 (12.8-25.9)	8.7 (6.1-11.8)	4.7 (3.3-6.4)	2.2 (1.5-3.1)	11.8 (8-16.4)
**Asia**	87.5	105.6 (72.1-147.1)	147.9 (104.3-201.9)	4.9 (3.0.3-7)	17.9 (12.3-24.9)	8.5 (6.0-11.7)	4.5 (3.1-6.2)	2.2 (1.5-3.1)	11.8 (8.1-16.3)
**Asia**	92.5	109.8 (75.1-153.3)	148.6 (104.9-203.2)	4.8 (3.2-6.9)	17.1 (11.7-24.1)	8.3 (5.7-11.3)	4.3 (3.1-5.9)	2.2 (1.5-3.1)	11.7 (8.1-16.4)
**Asia**	97.5	115.4 (79.1-160.6)	149.5 (106.4-205.4)	4.7 (3.1-6.7)	12.6 (4.8-20)	8.2 (5.7-11.3)	4.2 (2.9-5.7)	2.2 (1.5-3.2)	11.7 (8.0-16.4)
**CEECA**	0.5	23.1 (15.7-32.2)	15.5 (10.4-21.9)	8.4 (5.6-12.1)	2.8 (1.8-4.1)	7.1 (5.1-9.6)	8.2 (5.8-11.1)	2 .0(1.3-2.8)	6.8 (4.7-9.5)
**CEECA**	1.5	35.3 (24.0-49.6)	26.1 (17.6-36.9)	16.0 (10.7-22.6)	8.7 (5.7-12.4)	8 (5.7-10.9)	9.3 (6.5-12.7)	2.1 (1.4-2.9)	9.8 (6.9-13.5)
**CEECA**	3.5	48.5 (32.6-68.1)	38.9 (26.3-54.5)	25.5 (17.5-35.6)	21.5 (14.4-30.5)	8.7 (6.1-11.9)	10.2 (7.3-13.8)	2.2 (1.5-3.0)	12.9 (8.9-17.8)

**CEECA**	7.5	64.6 (44.2-90.5)	56.3 (37.0-80.6)	37.9 (26.2-52.7)	48.0 (32.1-68)	9.4 (6.7-12.7)	11.1 (7.8-14.9)	2.3 (1.5-3.2)	16.4 (11.5-22.6)
**CEECA**	12.5	76.6 (52.1-106.5)	74.8 (50.9-105.7)	47.0 (32.7-64.9)	72 (48.9-101.5)	9.9 (7.1-13.4)	11.8 (8.2-16.2)	2.3 (1.6-3.2)	18.6 (12.9-25.4)
**CEECA**	17.5	85.3 (58.4-119)	95.6 (63.9-136.9)	51.4 (36.6-70.6)	82.3 (56.1-116.2)	10.1 (7.2-13.6)	12.1 (8.7-16.6)	2.3 (1.6-3.3)	20.3 (14.1-28.1)
**CEECA**	22.5	92.5 (63.7-128.6)	118.7 (80.2-166.9)	53.3 (37.4-73.0)	83.1 (55.6-117.5)	10.3 (7.3-13.9)	12.5 (8.8-17.0)	2.3 (1.6-3.3)	20.7 (14.5-28.6)
**CEECA**	27.5	97.0 (66.3-134.8)	139.9 (95.6-198.1)	53.9 (37.8-73.2)	79.2 (53.8-111.6)	10.4 (7.4-14.0)	12.8 (9.1-17.4)	2.3 (1.6-3.3)	20.6 (14.3-28.4)
**CEECA**	32.5	100.2 (67.7-139.2)	155.2 (106.1-216.7)	53.7 (37.4-73.6)	74.6 (50.1-105.2)	10.4 (7.4-14.2)	13.0 (9.2-17.6)	2.3 (1.6-3.3)	20.4 (14.2-28.0)
**CEECA**	37.5	101.3 (69.7-141.3)	165.8 (111.9-232.2)	53.6 (37.2-73.5)	70.2 (47.0-99.3)	10.4 (7.4-14.0)	13.0 (9.3-17.7)	2.3 (1.6-3.3)	20.1 (14.0-27.6)
**CEECA**	42.5	101.6 (70.6-140)	172.7 (118.9-238.8)	52.7 (37.0-72.2)	66.0 (44.0-94.7)	10.4 (7.3-14.0)	13.0 (9.2-17.6)	2.3 (1.6-3.3)	19.9 (13.8-28.0)
**CEECA**	47.5	101.0 (70-139.9)	173.8 (117.9-242.3)	51.2 (36.2-69.9)	62.6 (41.7-88.7)	10.4 (7.3-14.1)	12.8 (9.0-17.5)	2.3 (1.6-3.2)	19.6 (13.5-27.0)
**CEECA**	52.5	99.7 (68.4-139.7)	173.1 (118.4-241.4)	49.9 (34.7-69.1)	59.9 (40.5-84.9)	10.3 (7.3-13.9)	12.5 (8.9-16.8)	2.3 (1.6-3.2)	19.4 (13.4-26.6)
**CEECA**	57.5	97.2 (66.8-136.2)	167.9 (115.6-233.1)	48.2 (33.4-65.9)	57.4 (38.0-81.5)	10.2 (7.3-13.9)	12.2 (8.7-16.7)	2.3 (1.6-3.2)	19.1 (13.2-26.3)
**CEECA**	62.5	94.8 (65.0-133.2)	161.6 (109.4-224.6)	47.1 (32.6-65.1)	55.3 (37.3-79)	10.1 (7.2-13.8)	11.9 (8.4-16.4)	2.3 (1.6-3.2)	18.7 (13-25.7)
**CEECA**	67.5	92.9 (63.9-129.7)	155.5 (104.6-219.2)	46.0 (31.9-63.4)	53 (35.3-75.2)	10.0 (7.2-13.7)	11.7 (8.3-15.8)	2.3 (1.6-3.2)	18.5 (12.9-25.3)
**CEECA**	72.5	91.5 (63.3-125.5)	149.4 (102.8-208.8)	44.8 (31.5-61.7)	51.3 (34.5-73.3)	9.9 (7.1-13.5)	11.5 (8.2-15.7)	2.3 (1.6-3.2)	18.3 (12.7-25.5)
**CEECA**	77.5	90.2 (62.2-124.7)	146.9 (100-205.6)	43.1 (29.9-59.1)	49.7 (33.4-70.8)	9.9 (7.0-13.3)	11.2 (8.0-15.1)	2.3 (1.6-3.2)	18.1 (12.7-25.0)
**CEECA**	82.5	88.1 (59.9-124.4)	141.8 (96.2-199.6)	42.2 (29.3-58.6)	48.3 (32.2-68.9)	9.8 (6.9-13.3)	11 (7.9-15.0)	2.3 (1.6-3.2)	17.9 (12.3-24.7)
**CEECA**	87.5	87.2 (59.3-123.1)	138.4 (94-193.1)	41.2 (28.6-57.4)	46.6 (31.3-66.1)	9.8 (7.0-13.1)	10.8 (7.6-14.7)	2.3 (1.5-3.2)	17.7 (12.4-24.4)
**CEECA**	92.5	86.1 (59.5-119.9)	134.8 (92.3-188.5)	40.0 (27.4-55.3)	44.9 (30.3-63.5)	9.7 (6.9-13.1)	10.6 (7.5-14.5)	2.2 (1.5-3.1)	17.5 (12.0-24.3)
**CEECA**	97.5	84.6 (58.1-117.2)	130.4 (88.1-183.8)	38.7 (26.9-53.5)	43.9 (29.6-62.3)	9.6 (6.8-13.1)	10.5 (7.3-14.4)	2.2 (1.5-3.1)	17.1 (12.0-23.5)
**HIC**	0.5	99.5 (69.3-136.5)	26.5 (19.0-35.7)	3.9 (2.6-5.6)	4.2 (2.9-5.8)	14.2 (10.2-19.1)	11.8 (8.5-16.0)	2.3 (1.6-3.2)	4.8 (3.3-6.6)
**HIC**	1.5	81.3 (56.7-112.1)	32.6 (23.2-43.8)	7.7 (5.2-10.9)	9.4 (6.6-12.9)	13.1 (9.4-17.6)	11.4 (8.2-15.5)	2.3 (1.6-3.2)	6.8 (4.7-9.3)
**HIC**	3.5	69.5 (48.5-96.0)	38.2 (27.4-51.9)	13.0 (8.8-18.2)	17.7 (12.6-24.0)	12.3 (8.7-16.5)	11.0 (7.8-15.1)	2.3 (1.6-3.2)	9.0 (6.3-12.4)
**HIC**	7.5	60.7 (42.1-83.8)	44.3 (31.7-59.4)	20.9 (14.1-29.5)	31.5 (22.5-42.3)	11.6 (8.3-15.5)	10.8 (7.7-14.5)	2.3 (1.6-3.2)	11.5 (8.1-15.7)
**HIC**	12.5	59.1 (41.4-81.8)	52 (37.2-70.0)	26.8 (18.5-37.4)	44.4 (31.1-60.5)	11.2 (8.0-15.1)	10.7 (7.7-14.5)	2.3 (1.6-3.3)	13.3 (9.3-18.0)
**HIC**	17.5	62.6 (43.9-85.5)	62.1 (44.1-83.6)	29.5 (19.9-40.9)	52.8 (37.7-71.8)	11.0 (7.9-14.8)	10.8 (7.6-14.6)	2.3 (1.6-3.2)	14.6 (10.3-19.9)
**HIC**	22.5	69.4 (48.6-95.0)	74.3 (53.4-100.3)	30.1 (20.5-42.3)	58.0 (41.3-78.3)	11.1 (7.9-14.9)	11.1 (7.9-14.9)	2.3 (1.6-3.2)	15.3 (10.8-20.7)
**HIC**	27.5	77.7 (54.4-106.2)	87.8 (63.2-118.7)	29.5 (20.1-41.2)	61.0 (43.3-82.2)	11.0 (7.8-14.9)	11.3 (8.1-15.2)	2.3 (1.6-3.2)	15.8 (11.1-21.8)
**HIC**	32.5	85.7 (60.2-117.6)	99.5 (70.8-133.2)	28.9 (19.7-40.7)	62.5 (44.6-84.0)	11.1 (7.9-15.0)	11.4 (8.1-15.4)	2.3 (1.6-3.3)	16.1 (11.3-22.0)
**HIC**	37.5	92.8 (64.9-128.4)	109.1 (78.2-145.9)	28 (19.1-39.2)	62.7 (44.8-84.9)	11.1 (7.9-15.0)	11.5 (8.1-15.7)	2.3 (1.6-3.2)	16.4 (11.5-22.3)
**HIC**	42.5	98.6 (69.0-135.9)	115.4 (83.5-154.9)	27.3 (18.3-38.3)	62.0(44.4-83.8)	11.2 (7.9-15.2)	11.6 (8.2-15.7)	2.3 (1.6-3.3)	16.6 (11.8-22.5)

**HIC**	47.5	103.5 (73-142.1)	118.0 (84.6-159.1)	26.5 (17.8-37.2)	61.1 (43.4-83.5)	11.1 (8-14.9)	11.5 (8.2-15.6)	2.3 (1.6-3.3)	16.7 (11.8-22.6)
**HIC**	52.5	107 (75.6-146.9)	117.9 (83.8-158.6)	25.8 (17.9-35.9)	59.5 (42.7-80.4)	11.2 (8.1-15.1)	11.5 (8.2-15.6)	2.4 (1.6-3.3)	16.8 (12-22.8)
**HIC**	57.5	109.6 (76.7-149.0)	116.3 (83.5-156.8)	25.4 (17.3-35.4)	57.8 (41.3-78.4)	11.2 (8-15.1)	11.4 (8.1-15.6)	2.4 (1.6-3.3)	16.9 (11.8-23.2)
**HIC**	62.5	111.8 (78.7-152.7)	113.5 (81.1-153.9)	24.9 (17.2-34.7)	55.9 (40.1-74.8)	11.2 (7.9-15.1)	11.3 (8.1-15.3)	2.3 (1.6-3.3)	17.1 (12.2-23.4)
**HIC**	67.5	114.1 (79.7-156.2)	110.7 (79.3-150.1)	24.6 (16.8-34.7)	54.2 (38.2-73.6)	11.2 (8.0-15.3)	11.2 (8.0-15.2)	2.3 (1.6-3.3)	17.2 (12.1-23.3)
**HIC**	72.5	115.5 (80.4-157.5)	108.2 (78.1-146.7)	24.0 (16.3-33.7)	52.6 (37.3-71.6)	11.2 (8.0-15.1)	11.1 (7.9-15.1)	2.3 (1.6-3.3)	17.2 (12.2-23.5)
**HIC**	77.5	117.3 (82.5-160.6)	105.9 (76.2-142.7)	23.5 (16.0-33.0)	51.0 (36.1-69.5)	11.1 (7.9-15)	11.1 (7.9-15.0)	2.3 (1.6-3.3)	17.3 (12.2-23.5)
**HIC**	82.5	120.1 (84.9-164)	104.6 (74.9-140.7)	23.1 (15.7-32.6)	49.3 (35.3-66)	11.2 (7.9-15.2)	11.0 (7.9-14.9)	2.3 (1.6-3.3)	17.4 (12.3-23.8)
**HIC**	87.5	122.5 (86.3-168.9)	103.7 (74.9-138.2)	22.5 (15.3-31.5)	48.2 (34.3-65.2)	11.2 (7.9-15.1)	11.0 (7.8-14.9)	2.3 (1.6-3.3)	17.4 (12.1-23.8)
**HIC**	92.5	125.1 (87.3-171.6)	102.9 (73.6-138.9)	21.9 (15.0-30.7)	46.7 (33.3-62.6)	11.2 (7.9-15.1)	10.9 (7.7-14.7)	2.3 (1.6-3.3)	17.6 (12.4-24.0)
**HIC**	97.5	128.6 (89.8-175.9)	102.4 (73.3-137.8)	21.4 (14.6-30)	45.6 (32.3-61.7)	11.2 (8-15.2)	10.8 (7.7-14.7)	2.3 (1.6-3.3)	17.6 (12.4-24.0)
**LAC**	0.5	17.3 (12.2-23.6)	12.0 (8.4-16.5)	4.8 (3.1-6.9)	1.8 (1.2-2.7)	14.1 (10.1-18.9)	12.2 (8.7-16.7)	2.1 (1.4-2.9)	4.9 (3.3-6.9)
**LAC**	1.5	32.6 (23.1-44.3)	26.2 (18.3-35.9)	9.3 (6.2-13.2)	6.0 (4.0-8.7)	11.5 (8.2-15.7)	10.5 (7.5-14.3)	2.2 (1.5-3.1)	6.9 (4.7-9.6)
**LAC**	3.5	53.1 (37.5-71.8)	47.7 (33.2-65.4)	15.8 (10.7-22.5)	16.0 (10.6-22.7)	9.8 (6.9-13.2)	9.4 (6.8-12.7)	2.2 (1.5-3.2)	8.9 (6.1-12.4)
**LAC**	7.5	82.0 (57.7-111.7)	81.7 (57.1-112.8)	24.3 (16.4-34.3)	37.8 (25.2-54.2)	8.4 (5.9-11.3)	8.5 (5.9-11.8)	2.3 (1.6-3.2)	11.3 (7.8-15.8)
**LAC**	12.5	100.2 (71.8-136.1)	107.7 (75.7-146.9)	29.6 (20.2-41.5)	54.7 (37.3-76.8)	7.6 (5.4-10.3)	8.1 (5.7-11.1)	2.3 (1.6-3.2)	13.0 (9.2-18.0)
**LAC**	17.5	104.1 (73.0-141.6)	118.4 (84.1-162.0)	31.0 (21.1-43.2)	58.1 (39.2-81.4)	7.3 (5.1-10)	8.0 (5.7-10.9)	2.3 (1.6-3.3)	13.9 (9.6-19.1)
**LAC**	22.5	99.9 (70.6-136.8)	119.4 (83.9-163.8)	30.6 (21-43.3)	53.5 (36.2-75.2)	7.1 (5.0-9.6)	8.1 (5.7-11.0)	2.4 (1.6-3.3)	14.2 (9.8-19.7)
**LAC**	27.5	93.6 (65.9-128.0)	115.7 (81.9-158.2)	29.0 (19.6-40.7)	47.2 (31.8-66.7)	7 .0(5.0-9.5)	8.2 (5.8-11.2)	2.4 (1.6-3.3)	14.3 (9.8-19.8)
**LAC**	32.5	88.1 (62.8-118.5)	112.2 (79.2-152.2)	27.2 (18.4-38.1)	42.0 (28.0-59.7)	7.0 (5.0-9.7)	8.3 (5.9-11.4)	2.4 (1.6-3.3)	14.2 (9.8-19.5)
**LAC**	37.5	85.1 (60.6-115.2)	109.8 (77.0-151.4)	25.4 (17.3-35.9)	38.6 (25.9-55.1)	7.1 (5.0-9.6)	8.4 (5.9-11.5)	2.4 (1.6-3.3)	14.2 (9.9-19.7)
**LAC**	42.5	83.9 (60.3-113.3)	108.7 (76.9-146.9)	24.0 (16.2-33.7)	36.7 (24.4-52.1)	7.1 (4.9-9.7)	8.4 (5.9-11.5)	2.4 (1.6-3.3)	14.1 (9.7-19.5)
**LAC**	47.5	83.9 (59.9-113.4)	109.1 (76.6-148.4)	22.6 (15.2-31.8)	36.4 (24.5-51.3)	7.1 (5.0-9.6)	8.3 (5.9-11.3)	2.4 (1.6-3.3)	13.9 (9.6-19.3)
**LAC**	52.5	85.6 (60-117.0)	110.7 (77.6-151.9)	21.4 (14.4-30.2)	37.4 (24.9-52.9)	7.1 (5.0-9.6)	8.3 (5.8-11.2)	2.4 (1.6-3.3)	13.7 (9.5-18.9)
**LAC**	57.5	89 (63.9-120.7)	114.4 (80.5-154.8)	20.4 (13.9-28.9)	39.6 (26.7-56.2)	7.2 (5.1-9.8)	8.1 (5.7-11)	2.3 (1.6-3.3)	13.7 (9.5-19)
**LAC**	62.5	92.8 (66.1-126.9)	116.7 (81.9-160.1)	19.3 (13.1-27.3)	42 (27.8-60)	7.2 (5.1-9.8)	8 (5.7-10.9)	2.3 (1.6-3.3)	13.6 (9.5-18.7)
**LAC**	67.5	96.5 (68.1-131.4)	120.2 (85-163.3)	18.5 (12.4-26.3)	44.6 (29.8-63.1)	7.2 (5.1-9.9)	7.9 (5.6-10.7)	2.3 (1.6-3.3)	13.5 (9.3-18.7)
**LAC**	72.5	100.6 (71.4-136.6)	123.2 (86.7-167.7)	17.8 (12-25.2)	46.8 (31.4-65.8)	7.2 (5.1-9.8)	7.8 (5.5-10.6)	2.3 (1.6-3.2)	13.3 (9.2-18.5)
**LAC**	77.5	103.7 (74.1-142.7)	125.5 (88.4-170.6)	17.1 (11.5-24.2)	49.2 (33.0-69.0)	7.2 (5.1-9.8)	7.7 (5.5-10.4)	2.3 (1.6-3.2)	13.3 (9.3-18.2)
**LAC**	82.5	107 (76.3-144.1)	127.8 (90.3-173.4)	16.5 (11.2-23.5)	51.1 (34.6-72.5)	7.3 (5.1-9.9)	7.6 (5.3-10.5)	2.3 (1.6-3.2)	13.1 (9.0-18.0)

**LAC**	87.5	110.1 (76.9-150)	130.5 (91.8-179.3)	15.9 (10.7-22.5)	53.4 (36.5-75.2)	7.3 (5.1-10)	7.5 (5.4-10.2)	2.3 (1.6-3.2)	13.0 (9.1-18.0)
**LAC**	92.5	112.8 (79.2-153.6)	132.3 (93.7-180.9)	15.3 (10.3-21.9)	55.3 (37.6-77.3)	7.3 (5.1-9.8)	7.4 (5.2-10.1)	2.3 (1.6-3.2)	12.9 (9.0-17.6)
**LAC**	97.5	115.2 (82.2-156.9)	134.1 (94.8-183.3)	14.7 (9.9-20.7)	56.3 (38.9-78.2)	7.2 (5.1-9.9)	7.3 (5.1-9.9)	2.3 (1.6-3.2)	12.8 (8.9-17.6)
**MENA**	0.5	9.3 (6.5-12.6)	4.6 (3.2-6.4)	3.7 (2.4-5.4)	1.0 (0.6-1.5)	7.6 (5.3-10.4)	8.0 (5.6-11.0)	2.6 (1.8-3.6)	5.8 (3.8-8.5)
**MENA**	1.5	20.2 (14.2-27.7)	13.6 (9.5-18.8)	7.1 (4.6-10.4)	3.7 (2.4-5.3)	7.9 (5.6-10.6)	8.5 (6.0-11.6)	2.5 (1.7-3.5)	8.9 (6.0-12.7)
**MENA**	3.5	37.2 (26-50.9)	31.8 (22.5-43.3)	12.0 (7.7-17.5)	11.2 (7.4-16.1)	8.1 (5.8-11.0)	9.0(6.4-12.3)	2.4 (1.7-3.4)	12.4 (8.4-17.5)
**MENA**	7.5	64.3 (45.3-87.4)	68.2 (47.9-92.8)	18.3 (12.0-26.2)	30.3 (20.2-42.8)	8.3 (5.9-11.3)	9.4 (6.6-12.9)	2.4 (1.6-3.3)	16.8 (11.6-23.3)
**MENA**	12.5	86 (61.1-117.1)	105.5 (75.5-143.8)	22.8 (15.0-32.6)	47.5 (32.5-66.8)	8.5 (6.0-11.6)	9.9 (7.0-13.5)	2.3 (1.6-3.3)	20.1 (14.0-27.5)
**MENA**	17.5	95 (66.8-129.4)	126.6 (89.9-170.3)	24.3 (16.0-35.0)	50.9 (34.5-71.2)	8.7 (6.2-11.8)	10.4 (7.4-14.2)	2.4 (1.6-3.3)	23.1 (15.9-31.8)
**MENA**	22.5	96.8 (68.4-133.3)	135.1 (95.5-184.7)	24.2 (15.9-34.9)	45.9 (31.3-64.9)	8.9 (6.3-12.1)	11.0 (7.8-15.0)	2.4 (1.6-3.2)	24.4 (16.7-34.0)
**MENA**	27.5	94.6 (65.4-130.9)	137.3 (96.8-189.4)	23.6 (15.5-34.3)	39.3 (26.2-55.6)	9.0 (6.3-12.1)	11.4 (8.0-15.7)	2.4 (1.6-3.3)	25.3 (17.6-35.1)
**MENA**	32.5	93.3 (65.7-128.1)	137.8 (97-187.5)	22.3 (14.7-32.1)	34.4 (22.9-49.2)	9.0 (6.4-12.4)	11.7 (8.4-16.1)	2.4 (1.6-3.4)	25.5 (17.7-35.4)
**MENA**	37.5	93.3 (65.6-128.2)	138.6 (97.3-188.2)	20.8 (13.5-30.2)	31.1 (20.4-44.9)	9.0 (6.4-12.2)	11.8 (8.3-16.1)	2.4 (1.7-3.4)	25.7 (17.7-35.6)
**MENA**	42.5	93.5 (66.1-128.3)	140.6 (99.2-191.3)	19.4 (12.6-28.2)	29 (19.5-41.0)	9.0 (6.3-12.2)	11.7 (8.4-15.9)	2.4 (1.7-3.3)	25.6 (17.6-35.6)
**MENA**	47.5	94.7 (66.4-129.5)	142.0 (98.5-194.1)	17.9 (11.6-25.9)	28.4 (18.7-40.1)	8.9 (6.3-12.0)	11.5 (8.1-15.7)	2.4 (1.7-3.4)	25.4 (17.4-35.6)
**MENA**	52.5	97.3 (68.6-132.6)	144.4 (101.1-195.9)	16.7 (11.0-24.2)	29.1 (19.1-41.3)	8.8 (6.2-12.0)	11.2 (7.9-15.3)	2.4 (1.7-3.4)	25.2 (17.2-35.3)
**MENA**	57.5	100 (70-135.1)	148.5 (105.2-203.2)	15.6 (10.3-22.4)	30.6 (20.6-43.3)	8.6 (6.1-11.8)	10.8 (7.7-14.7)	2.4 (1.6-3.3)	25.1 (17.1-35.2)
**MENA**	62.5	103.8 (72.9-141.1)	152.7 (109.1-205.2)	14.5 (9.6-21.0)	32.2 (21.5-46.1)	8.5 (5.9-11.7)	10.4 (7.4-14.1)	2.4 (1.6-3.3)	24.9 (17.0-34.5)
**MENA**	67.5	108 (75.4-150.1)	156.9 (110.7-212.3)	13.5 (8.9-19.4)	33.9 (22.6-48.2)	8.4 (5.9-11.5)	10.0 (7.0-13.7)	2.4 (1.6-3.3)	24.6 (17.1-34.0)
**MENA**	72.5	111 (77.7-150.9)	161.7 (114.1-220.1)	12.4 (8.1-17.9)	35.2 (23.5-49.8)	8.3 (5.9-11.3)	9.7 (6.9-13.2)	2.4 (1.6-3.3)	24.6 (17.0-34.4)
**MENA**	77.5	115 (80.9-156.9)	165.5 (117.3-224.0)	11.8 (7.7-17.0)	37.1 (24.5-53.0)	8.2 (5.7-11.0)	9.4 (6.6-12.9)	2.3 (1.6-3.2)	24.4 (16.8-33.9)
**MENA**	82.5	118.2 (83.4-162.7)	169.2 (119.2-230.9)	11.1 (7.3-15.9)	38.7 (26.2-54.7)	8.1 (5.7-11.0)	9.0(6.4-12.2)	2.3 (1.6-3.2)	24.4 (16.5-34.4)
**MENA**	87.5	120.8 (84.8-165.4)	173 (123-234.8)	10.3 (6.8-14.7)	40 (26.5-56.9)	8 (5.6-10.8)	8.7 (6.2-11.8)	2.3 (1.6-3.3)	24.6 (17-34.3)
**MENA**	92.5	124.2 (88.3-167.8)	176.7 (123.9-240.8)	9.4 (6.2-13.7)	40.9 (27.3-57.9)	7.9 (5.5-10.7)	8.5 (6-11.6)	2.3 (1.6-3.3)	24.9 (17.3-34.8)
**MENA**	97.5	126.6 (88.9-173)	181.4 (128.5-245)	8.6 (5.6-12.4)	41.6 (28-59)	7.8 (5.5-10.6)	8.2 (5.8-11.1)	2.3 (1.6-3.2)	25.4 (17.6-35.3)
**SAARC**	0.5	13.3 (9.0-18.8)	5.8 (4.0-7.9)	1.1 (0.7-1.8)	1 (0.6-1.6)	10.4 (7.4-14.3)	8.2 (5.8-11.3)	2.2 (1.5-3)	11 (7.5-15.3)
**SAARC**	1.5	27.1 (18.6-37.9)	18.2 (12.7-25.0)	1.8 (1.1-2.8)	2.8 (1.8-4.1)	8.3 (5.8-11.3)	7 (4.9-9.6)	2.2 (1.5-3.1)	15.6 (10.6-21.7)
**SAARC**	3.5	46.2 (31.4-64.6)	44.7 (31.8-60.9)	2.7 (1.7-4.2)	6.5 (4.2-9.4)	6.8 (4.7-9.4)	6.2 (4.3-8.5)	2.2 (1.5-3.1)	19.9 (13.7-27.5)
**SAARC**	7.5	75.2 (51.4-106.0)	99.6 (70.3-135.1)	3.9 (2.4-5.9)	14.2 (9.4-20.4)	5.7 (4.0-7.9)	5.5 (3.8-7.5)	2.2 (1.5-3.1)	24.1 (17-33.2)
**SAARC**	12.5	94.8 (65.4-133.1)	153.1 (108-208.8)	4.6 (2.8-7.0)	20.5 (13.6-29.7)	5.1 (3.5-7.1)	5.2 (3.6-7.1)	2.2 (1.5-3.2)	26.5 (18.5-36.4)

**SAARC**	17.5	98.8 (67.2-138)	182.2 (129.4-247)	4.7 (2.9-7.1)	22.7 (15-32.7)	4.9 (3.4-6.7)	5.0(3.5-7.0)	2.2 (1.5-3.1)	31.2 (21.5-43.2)
**SAARC**	22.5	93.7 (63.4-131.2)	189.7 (135.3-256.7)	4.4 (2.7-6.7)	21.9 (14.5-31.3)	4.8 (3.3-6.6)	5.0 (3.4-6.9)	2.2 (1.5-3.1)	31.9 (22-44.3)
**SAARC**	27.5	86 (58.4-120.7)	187.3 (132.5-255.6)	3.9 (2.4-6.0)	20.2 (13.3-29.2)	4.8 (3.3-6.6)	5.0 (3.4-6.9)	2.2 (1.5-3.1)	32.2 (22.3-44.5)
**SAARC**	32.5	80.6 (54.9-113.6)	180.5 (127.6-244.5)	3.6 (2.2-5.5)	18.2 (12.1-25.9)	4.8 (3.3-6.7)	5.0 (3.5-6.9)	2.2 (1.5-3.1)	32.1 (22.1-44.2)
**SAARC**	37.5	77.4 (53.0-108.0)	173.6 (122.6-236.2)	3.4 (2.1-5.2)	16.6 (11.0-24.0)	4.8 (3.3-6.7)	5.0 (3.5-6.9)	2.2 (1.5-3.1)	32.6 (22.8-45.2)
**SAARC**	42.5	75.8 (51.3-106.1)	169.4 (119.8-230.3)	3.4 (2.1-5.2)	15.6 (10.2-22.5)	4.9 (3.4-6.8)	5.1 (3.5-7.1)	2.2 (1.5-3.1)	32.6 (22.6-44.9)
**SAARC**	47.5	75.5 (51.5-104.8)	165.3 (116.9-224.7)	3.3 (2.0-5.0)	14.9 (9.8-21.4)	5 (3.5-6.9)	5.1 (3.6-7.0)	2.2 (1.5-3.1)	32.7 (23-44.8)
**SAARC**	52.5	76.7 (52.5-108)	162.3 (115.6-220)	3.4 (2.1-5.1)	14.6 (9.7-20.9)	5.1 (3.5-7.0)	5.2 (3.6-7.1)	2.2 (1.5-3.1)	32.7 (22.8-45.2)
**SAARC**	57.5	78.6 (53.7-109.5)	160.9 (114.7-219)	3.4 (2.1-5.2)	14.6 (9.7-20.8)	5.1 (3.6-7.1)	5.2 (3.7-7.2)	2.2 (1.6-3.1)	32.8 (23.1-45)
**SAARC**	62.5	81.2 (55.4-113.4)	159.0 (112.0-214.9)	3.5 (2.2-5.4)	14.5 (9.6-21.0)	5.2 (3.6-7.2)	5.3 (3.7-7.4)	2.3 (1.5-3.2)	33.1 (22.9-46)
**SAARC**	67.5	83.6 (57-116.8)	158.7 (112.2-216.5)	3.7 (2.3-5.6)	14.5 (9.6-21.0)	5.3 (3.7-7.1)	5.3 (3.7-7.2)	2.3 (1.6-3.2)	33.3 (23.1-45.8)
**SAARC**	72.5	85.4 (57.6-120.1)	157.8 (111.0-214.5)	3.7 (2.3-5.6)	14.6 (9.6-21.0)	5.3 (3.7-7.3)	5.3 (3.7-7.4)	2.3 (1.6-3.1)	33.4 (22.9-46.7)
**SAARC**	77.5	86.7 (58.5-121.3)	157.5 (112.2-213.9)	3.8 (2.3-5.8)	14.8 (9.7-21.4)	5.4 (3.7-7.5)	5.3 (3.7-7.4)	2.3 (1.6-3.1)	32.9 (22.9-45.1)
**SAARC**	82.5	87.7 (59.8-122.8)	155.8 (109.9-211.3)	3.8 (2.3-5.8)	14.9 (9.8-21.5)	5.4 (3.8-7.4)	5.3 (3.7-7.3)	2.3 (1.6-3.1)	33.1 (22.7-45.9)
**SAARC**	87.5	88.7 (60.2-124.5)	154.2 (109.2-208.5)	3.8 (2.4-5.8)	15 (9.8-21.3)	5.4 (3.8-7.6)	5.3 (3.7-7.4)	2.3 (1.6-3.1)	33.3 (23.2-45.8)
**SAARC**	92.5	88.5 (60.4-124.3)	152.2 (107.2-206.2)	3.8 (2.3-5.8)	15.1 (10-21.6)	5.5 (3.8-7.5)	5.3 (3.8-7.3)	2.3 (1.5-3.2)	33 (23.2-45.5)
**SAARC**	97.5	86.9 (59-123.3)	150.2 (105.9-204.3)	3.7 (2.2-5.7)	13.5 (5.2-21.4)	5.4 (3.8-7.5)	5.3 (3.7-7.3)	2.3 (1.5-3.2)	32.7 (22.7-45.1)
**SSA**	0.5	8.1 (5.6-11.3)	9.0 (6.2-12.5)	2.3 (1.4-3.4)	0.7 (0.4-1.0)	9.7 (6.9-13.1)	9.7 (6.8-13.3)	2.3 (1.6-3.2)	7.6 (5.3-10.7)
**SSA**	1.5	17.9 (12.4-24.9)	22.0 (15.3-30.1)	4.3 (2.7-6.3)	2.1 (1.3-3.0)	8.9 (6.3-12.2)	9.4 (6.7-12.7)	2.3 (1.6-3.2)	11.1 (7.7-15.4)
**SSA**	3.5	33.4 (23.4-46)	44.4 (30.8-61)	6.9 (4.4-10.1)	5.5 (3.7-7.9)	8.3 (5.9-11.3)	9.2 (6.5-12.4)	2.4 (1.6-3.3)	14.9 (10.3-20.6)
**SSA**	7.5	58 (39.7-80.8)	83.1 (57.5-115.7)	10.5 (6.7-15.3)	13.6 (9.1-19.4)	7.8 (5.5-10.7)	8.9 (6.3-12.1)	2.4 (1.6-3.3)	18.8 (13.1-26)
**SSA**	12.5	74.6 (52.4-103.1)	110.6 (75.8-154.0)	12.2 (7.8-17.8)	19.4 (12.9-27.7)	7.5 (5.3-10.1)	9.1 (6.4-12.4)	2.4 (1.6-3.3)	21.0 (14.7-29)
**SSA**	17.5	83 (57.7-114.3)	129.2 (90.5-176.8)	12.7 (8.1-18.6)	20.7 (13.7-29.6)	7.5 (5.2-10.1)	9.2 (6.5-12.4)	2.4 (1.6-3.3)	24.4 (17.3-33.7)
**SSA**	22.5	82.7 (58.1-113.2)	130.5 (90.5-179.3)	12.0 (7.7-17.5)	17.9 (12-25.7)	7.6 (5.4-10.3)	9.3 (6.5-12.7)	2.4 (1.6-3.3)	25.3 (17.7-34.9)
**SSA**	27.5	78.7 (55.2-108.4)	126.2 (88.5-174.3)	11.0 (7.2-16.2)	14.9 (10.0-21.0)	7.7 (5.5-10.4)	9.6 (6.7-13.1)	2.3 (1.6-3.3)	25.8 (17.8-35.6)
**SSA**	32.5	75.2 (51.3-105.2)	121.7 (85.5-166.8)	10.1 (6.5-14.7)	13 (8.7-18.3)	7.8 (5.5-10.7)	9.8 (6.9-13.4)	2.3 (1.6-3.2)	26.0 (18.0-36.0)
**SSA**	37.5	72.1 (50-100.2)	117.7 (82.6-162.9)	9.2 (5.9-13.3)	12.3 (8.2-17.5)	7.9 (5.5-10.8)	9.9 (6.9-13.6)	2.3 (1.6-3.2)	26.2 (18.2-36)
**SSA**	42.5	70.1 (48.9-95.7)	115.6 (80.5-160.4)	8.4 (5.4-12.3)	12.5 (8.3-17.8)	8.0 (5.7-10.8)	10.1 (7.1-13.7)	2.3 (1.6-3.2)	26.2 (18.3-36.3)
**SSA**	47.5	69 (48.6-94.6)	115.1 (79.5-159.5)	7.8 (5.0-11.3)	13.6 (9.1-19.6)	8.1 (5.6-11.1)	10.1 (7.2-13.6)	2.3 (1.6-3.2)	26.0 (17.9-35.7)
**SSA**	52.5	68.9 (47.8-95.4)	115.6 (80.0-159.7)	7.2 (4.6-10.8)	15.7 (10.6-22.2)	8.1 (5.8-11.1)	10.2 (7.2-14.0)	2.3 (1.6-3.2)	25.9 (18.1-35.8)

**SSA**	57.5	69.1 (47.9-95.5)	116.4 (82.0-159.4)	6.8 (4.4-10.2)	18.8 (12.6-26.6)	8.2 (5.7-11.1)	10.2 (7.2-13.8)	2.3 (1.6-3.2)	25.6 (17.8-35.5)
**SSA**	62.5	70 (49.4-95.5)	118.7 (82.7-164.6)	6.4 (4.1-9.6)	22.8 (15.2-32.6)	8.2 (5.8-11.2)	10.2 (7.3-13.9)	2.3 (1.6-3.2)	25.3 (17.7-34.7)
**SSA**	67.5	70.4 (48.9-96.6)	119.2 (82.5-164.6)	6.1 (3.9-9.0)	27.2 (18.2-38.4)	8.3 (5.9-11.3)	10.2 (7.2-13.9)	2.3 (1.6-3.2)	25.1 (17.6-34.4)
**SSA**	72.5	71.5 (49.5-97.9)	121.3 (84.2-166.8)	5.8 (3.7-8.6)	32.5 (22.1-45.2)	8.4 (5.9-11.4)	10.2 (7.3-13.8)	2.3 (1.6-3.2)	24.9 (17.4-34.2)
**SSA**	77.5	71.7 (49.7-99)	122.7 (86.4-168.9)	5.5 (3.5-8.2)	38.1 (25.6-53.4)	8.4 (6.0-11.4)	10.2 (7.2-13.7)	2.3 (1.6-3.2)	24.7 (17.1-34.1)
**SSA**	82.5	72 (49.6-100)	124.2 (86.8-170.1)	5.3 (3.4-7.8)	43.7 (29.6-61.2)	8.5 (6.0-11.6)	10.2 (7.2-13.8)	2.3 (1.6-3.2)	24.5 (17.1-33.6)
**SSA**	87.5	72.3 (50.3-99.3)	124.2 (86.1-172.4)	5.1 (3.2-7.6)	49.7 (33.5-70.4)	8.5 (6.1-11.5)	10.2 (7.2-13.7)	2.3 (1.6-3.2)	24.3 (16.9-33.6)
**SSA**	92.5	72.3 (50.2-98.8)	124.8 (86.4-171.4)	4.8 (3-7.1)	56.4 (38.1-79.3)	8.5 (6.1-11.5)	10.2 (7.3-13.8)	2.3 (1.6-3.2)	24.0 (16.8-33.2)
**SSA**	97.5	71.9 (49.4-99.5)	124.1 (86.8-170.4)	4.7 (3.0-7.0)	65 (43.8-92.4)	8.6 (6.1-11.7)	10.2 (7.3-13.6)	2.3 (1.5-3.2)	23.8 (16.6-32.5)
**world**	0.5	24.2 (15.5-35.6)	14.2 (9.4-20.2)	4.5 (2.9-6.7)	2.0 (1.2-3.0)	10.9 (7.7-14.9)	10.0 (7.1-13.6)	2.2 (1.5-3.1)	5.5 (3.7-7.8)
**world**	1.5	34.2 (23-48)	26.8 (18.4-37.5)	8.5 (5.4-12.3)	5.6 (3.6-8.3)	10.1 (7.2-13.7)	9.7 (6.9-13.2)	2.3 (1.6-3.1)	8.0 (5.4-11.4)
**world**	3.5	47.9 (33.3-66.2)	45.1 (31.5-62.1)	13.7 (8.8-19.9)	13.3 (8.7-19.5)	9.6 (6.8-13.3)	9.4 (6.6-12.8)	2.3 (1.6-3.2)	10.7 (7.3-14.9)
**world**	7.5	66.6 (46.5-91.4)	73.6 (51-101.5)	20.7 (13.4-30.3)	29.1 (19.3-41.4)	9.2 (6.5-12.6)	9.3 (6.6-12.6)	2.3 (1.6-3.2)	13.8 (9.4-19.5)
**world**	12.5	80.2 (56.2-110.6)	98.3 (67.4-137.0)	24.8 (16.2-35.8)	42.3 (27.8-60.7)	8.9 (6.3-12.1)	9.3 (6.5-12.7)	2.3 (1.6-3.2)	16.0 (11.0-22.1)
**world**	17.5	85.6 (59.3-118.4)	113.1 (78.6-155.5)	25.7 (16.9-37.0)	46.1 (30.6-65.3)	8.8 (6.2-12.1)	9.4 (6.6-13.0)	2.3 (1.6-3.3)	17.8 (12.1-24.8)
**world**	22.5	85.6 (59.7-117.7)	121.6 (84.7-167.6)	24.6 (16.1-35.8)	44.2 (28.8-63.6)	8.9 (6.2-12.2)	9.6 (6.7-13.1)	2.3 (1.6-3.3)	18.6 (12.6-26.2)
**world**	27.5	84.4 (58.9-115.7)	126.0 (88.0-171.9)	23.0 (14.9-33.6)	40.4 (26.5-58.4)	8.9 (6.2-12.2)	9.8 (6.9-13.4)	2.3 (1.6-3.2)	19.0 (13.0-26.6)
**world**	32.5	83.3 (57.3-114.6)	128.4 (88.7-177.9)	21.4 (13.5-31.5)	37.2 (24.3-54.1)	8.9 (6.3-12.2)	9.9 (6.9-13.5)	2.3 (1.6-3.2)	19.3 (13.2-26.7)
**world**	37.5	82.1 (56.9-113.3)	129.9 (91.6-178.9)	20.0 (12.7-29.6)	34.7 (22.5-50.7)	8.9 (6.2-12.1)	9.9 (6.9-13.5)	2.3 (1.6-3.2)	19.4 (13.3-27.0)
**world**	42.5	82 (56.6-113.5)	131.0 (92.1-180.1)	18.8 (11.7-28.5)	33 (21.4-48.3)	8.9 (6.3-12.1)	9.9 (7.0-13.4)	2.3 (1.6-3.2)	19.4 (13.4-26.9)
**world**	47.5	82.6 (57.4-115.4)	130.8 (91.0-180)	17.7 (11.3-26.5)	32.4 (21.2-47.3)	8.8 (6.2-12.2)	9.8 (6.8-13.4)	2.3 (1.6-3.2)	19.4 (13.5-27.0)
**world**	52.5	83.7 (58.1-116.1)	131.6 (91.6-181.8)	16.7 (10.5-24.9)	32.5 (21.2-47.2)	8.8 (6.2-12.0)	9.7 (6.8-13.1)	2.3 (1.6-3.2)	19.3 (13.4-27.1)
**world**	57.5	84.9 (58.9-117.3)	131.3 (92.2-181.2)	15.8 (9.8-23.6)	33.3 (22-47.8)	8.7 (6.2-11.8)	9.5 (6.7-13.2)	2.3 (1.6-3.3)	19.3 (13.4-26.7)
**world**	62.5	86.7 (60.4-119.7)	132.1 (92.8-181.2)	15.1 (9.4-22.7)	34.5 (23.2-49.3)	8.7 (6.2-11.7)	9.3 (6.5-12.9)	2.3 (1.6-3.2)	19.2 (13.1-26.7)
**world**	67.5	88.6 (61.8-122.5)	132.5 (92.7-181.5)	14.6 (9.1-21.9)	35.7 (23.6-51.1)	8.6 (6.1-11.7)	9.2 (6.3-12.5)	2.3 (1.6-3.2)	19.1 (13.1-26.3)
**world**	72.5	90.5 (62.6-125)	133.5 (93.6-184.3)	14.0 (8.8-20.9)	37.3 (25.0-52.7)	8.6 (6.0-11.7)	9.0 (6.2-12.6)	2.3 (1.6-3.2)	19.0 (13.1-26.4)
**world**	77.5	92.2 (64-128)	134.2 (94.4-183.3)	13.4 (8.3-20.3)	38.8 (25.9-55.7)	8.6 (6.1-11.7)	8.9 (6.3-12.1)	2.3 (1.6-3.2)	19.0 (13.1-26.4)
**world**	82.5	93.8 (65.3-129.4)	134.6 (94.3-185.3)	12.8 (8.0-19.4)	40.2 (27.4-56.7)	8.5 (6.0-11.7)	8.8 (6.1-12.0)	2.3 (1.6-3.2)	18.9 (13.1-26.3)
**world**	87.5	95.2 (65.1-132)	134.5 (94.0-183.8)	12.3 (7.6-18.6)	42.0 (27.8-60.5)	8.5 (6.0-11.6)	8.6 (6.1-11.7)	2.3 (1.6-3.2)	18.8 (13.0-26.0)
**world**	92.5	96.3 (66.3-133.3)	135.8 (94.7-187.1)	11.6 (7.3-17.3)	43.5 (29.0-62.1)	8.4 (5.9-11.5)	8.5 (5.9-11.7)	2.3 (1.6-3.2)	18.8 (12.9-26.0)

**world**	97.5	97.0 (67.5-133.3)	136.0 (94.3-186.5)	11.2 (6.9-17.2)	46.9 (30.8-67.9)	8.4 (5.9-11.3)	8.4 (5.9-11.4)	2.3 (1.6-3.2)	18.9 (13.1-26.1)

In previous global dietary database reports, Central Europe, Eastern Europe, and Central Asia were referred to as the former Soviet Union, while Southeast Asia and East Asia were collectively called Asia. UI, Uncertainty Interval. Midpoint ages correspond to specific age groups.Ages 0.5, 1.5, and 3.5 represent 0–1 year, 1–2 years, and 3–4 years, respectively. From 7.5 onwards, midpoint ages represent standard 5-year intervals (e.g. 5–9 years, 10–14 years, etc.).

Supplementary Table 9: National mean (95% UI) intakes of eight nutrients among males in 2018 across 185 countries, stratified by area of residence for all age groups.

Region	Age	Fruits	Non-starchy vegetables	Total processed meats	Unprocessed red meats	Saturated fat	Monounsaturated fatty acids	Total omega-6 fat	Dietary fiber
**Asia**	0.5	27.7 (19.1-38.5)	26.7 (18.9-36.3)	6.6 (4.5-9.3)	4.6 (3.1-6.5)	13.6 (9.6-18.5)	9.9 (7.0-13.3)	2.3 (1.5-3.2)	2.0 (1.3-2.8)
**Asia**	1.5	39.7 (27.6-55.1)	44.8 (31.7-61.6)	12.2 (8.2-17.5)	10.4 (7.0-14.5)	12.9 (9.1-17.6)	9.1 (6.4-12.5)	2.3 (1.6-3.1)	3.0 (2.1-4.2)
**Asia**	3.5	52.6 (35.9-72.8)	67.2 (47.7-91.2)	19.3 (13.0-27.2)	19.7 (13.4-27.5)	12.6 (8.8-17.2)	8.5 (6.1-11.6)	2.3 (1.6-3.2)	4.3 (3.0-6.0)
**Asia**	7.5	67.6 (46.6-92.6)	96.4 (68.4-132.1)	28.2 (19.4-39.4)	34.8 (23.9-48.0)	12.2 (8.6-16.7)	8.1 (5.8-11.1)	2.3 (1.6-3.2)	6.0 (4.2-8.4)
**Asia**	12.5	74.5 (51.8-102.9)	117.6 (84.2-158.4)	30.0 (20.4-41.7)	45.6 (31-63.5)	12 (8.5-16.4)	7.8 (5.6-10.6)	2.3 (1.6-3.2)	7.4 (5.1-10.1)
**Asia**	17.5	72.9 (50.3-100.6)	128.6 (90.8-176.0)	25.7 (17.5-35.9)	48.8 (33.2-68.1)	11.9 (8.2-16.3)	7.7 (5.3-10.5)	2.3 (1.6-3.3)	8.5 (5.9-11.8)
**Asia**	22.5	68.8 (47.9-95.4)	133.7 (94.7-181.6)	19.8 (13.5-27.8)	47.9 (32.9-66.8)	11.8 (8.3-16.0)	7.6 (5.3-10.3)	2.3 (1.6-3.2)	9.3 (6.4-12.8)
**Asia**	27.5	63.8 (44.1-88.4)	134.8 (95.6-184.2)	14.7 (9.9-20.8)	45.0 (30.8-62.6)	11.6 (8.2-15.8)	7.5 (5.4-10.2)	2.3 (1.6-3.2)	9.9 (6.9-13.7)
**Asia**	32.5	60.7 (42.5-83.3)	135.1 (95.7-183.3)	11.2 (7.5-15.9)	41.2 (28.4-57.6)	11.5 (8.1-15.6)	7.3 (5.2-9.9)	2.3 (1.6-3.2)	10.4 (7.3-14.4)
**Asia**	37.5	58.6 (40.0-80.8)	134.1 (95.5-183.5)	8.9 (6.0-12.6)	38.0 (26.2-53.1)	11.3 (7.9-15.3)	7.2 (5.1-9.7)	2.3 (1.6-3.2)	10.8 (7.6-14.9)
**Asia**	42.5	58.9 (40.7-81.9)	134.2 (94.8-184.4)	7.5 (5-10.5)	35.1 (23.6-49.2)	10.9 (7.7-14.9)	6.9 (5-9.4)	2.3 (1.6-3.2)	11.1 (7.7-15.4)
**Asia**	47.5	60.5 (42.1-83.4)	134.3 (95.3-183.3)	6.5 (4.4-9.3)	32.7 (22.6-45.5)	10.6 (7.4-14.4)	6.6 (4.7-8.9)	2.3 (1.6-3.2)	11.2 (7.8-15.5)
**Asia**	52.5	62.8 (43.9-86.8)	134.7 (95.1-183.5)	6.0 (4.0-8.4)	30.5 (20.8-42.3)	10.3 (7.2-13.9)	6.3 (4.4-8.6)	2.3 (1.5-3.2)	11.4 (7.8-15.9)
**Asia**	57.5	66.7 (46.2-92.8)	135.7 (95.4-185.9)	5.7 (3.8-8.2)	28.6 (19.6-39.8)	9.9 (7.0-13.6)	5.9 (4.2-8.1)	2.3 (1.6-3.2)	11.4 (7.7-15.9)
**Asia**	62.5	71.1 (48.4-98.8)	135.8 (96.7-183.4)	5.4 (3.6-7.7)	27.0 (18.4-37.9)	9.6 (6.8-13.1)	5.6 (3.9-7.6)	2.3 (1.5-3.1)	11.4 (7.9-15.8)
**Asia**	67.5	75.7 (52.2-104.9)	136.8 (97.9-186.9)	5.3 (3.6-7.6)	25.6 (17.4-36)	9.3 (6.5-12.7)	5.3 (3.7-7.3)	2.2 (1.5-3.1)	11.5 (7.9-15.7)
**Asia**	72.5	80.2 (55.5-112)	138.0 (97.2-188.4)	5.1 (3.4-7.4)	24.3 (16.4-33.9)	8.9 (6.2-12.3)	5.0 (3.6-6.8)	2.3 (1.5-3.2)	11.5 (8.0-15.9)
**Asia**	77.5	83.9 (57.8-116.7)	138.3 (97.5-188.8)	4.9 (3.2-7.1)	23.1 (15.9-32.1)	8.7 (6.1-11.9)	4.8 (3.3-6.6)	2.2 (1.5-3.1)	11.5 (8.0-15.9)
**Asia**	82.5	88.1 (61.0-122.5)	138.3 (96.9-189.5)	4.8 (3.2-6.9)	22 (15.0-31.1)	8.5 (5.8-11.6)	4.6 (3.2-6.3)	2.2 (1.5-3.1)	11.5 (8.0-16.1)
**Asia**	87.5	92.4 (63.5-129.1)	138.7 (99.1-188.6)	4.7 (3.1-6.8)	21.1 (14.5-29.5)	8.3 (5.7-11.4)	4.4 (3.0-6.1)	2.2 (1.5-3.1)	11.5 (8.0-15.9)
**Asia**	92.5	96.0 (66.8-132.6)	139.1 (98.6-189.6)	4.6 (3.0-6.6)	20 (13.6-28.2)	8.1 (5.6-11.1)	4.2 (3.0-5.8)	2.2 (1.5-3.1)	11.4 (7.9-15.7)
**Asia**	97.5	101.0 (69.7-138.9)	139.6 (97.9-188.8)	4.5 (3.0-6.4)	18.8 (12.7-26.4)	7.9 (5.5-10.8)	4 (2.8-5.6)	2.2 (1.5-3.1)	11.4 (8.0-15.7)
**CEECA**	0.5	20.6 (13.9-29.3)	14.5 (9.7-20.6)	9.1 (6.0-13.0)	3.3 (2.1-4.7)	7.0 (4.9-9.5)	8.4 (5.9-11.3)	2.0 (1.3-2.7)	6.8 (4.7-9.5)
**CEECA**	1.5	31.5 (21.3-43.9)	24.4 (16.4-34.1)	17.2 (11.4-24.3)	10.2 (6.8-14.6)	7.9 (5.6-10.6)	9.5 (6.6-13)	2.1 (1.4-2.9)	9.8 (6.8-13.6)
**CEECA**	3.5	43.3 (29.4-60.9)	36.4 (24.3-52.3)	27.6 (18.8-38.7)	25.0 (17.0-35.4)	8.6 (6.1-11.6)	10.4 (7.4-14.1)	2.2 (1.0.5-3)	12.9 (9-17.9)

**CEECA**	7.5	57.6 (39.4-80.8)	52.3 (35.3-73.5)	41.5 (28.5-57.8)	55.2 (37.4-77.6)	9.2 (6.5-12.4)	11.4 (8.0-15.6)	2.2 (1.5-3.1)	16.4 (11.4-22.8)
**CEECA**	12.5	69.0 (47.4-96.1)	69.9 (47.7-97.3)	51.1 (35.8-70.5)	80.8 (55.0-112.8)	9.7 (6.8-13.2)	12.0 (8.5-16.2)	2.3 (1.6-3.2)	18.6 (12.9-25.5)
**CEECA**	17.5	77.3 (52.1-108.8)	89.4 (59.9-127.1)	56.0 (39.3-77.2)	91.3 (62.1-129.4)	9.9 (7.0-13.4)	12.4 (8.8-16.8)	2.3 (1.6-3.2)	20.3 (14.1-28.0)
**CEECA**	22.5	83.6 (57.1-117.3)	111.5 (74.8-158.5)	58.2 (40.7-79.7)	92.4 (63.9-128)	10.1 (7.2-13.9)	12.8 (9.1-17.4)	2.3 (1.6-3.2)	20.7 (14.6-28.4)
**CEECA**	27.5	88.2 (60.3-122.2)	131.4 (89.0-184.9)	58.7 (41.4-80.1)	88.8 (61.3-124.4)	10.2 (7.3-13.9)	13.1 (9.3-17.7)	2.3 (1.6-3.3)	20.7 (14.3-28.6)
**CEECA**	32.5	90.2 (62.2-126.3)	148.0 (100.5-208.8)	58.6 (41.1-80.0)	84.1 (57.4-118.6)	10.3 (7.2-14.0)	13.2 (9.4-18.1)	2.3 (1.6-3.3)	20.5 (14.3-28.3)
**CEECA**	37.5	91.3 (63.1-127.4)	157.5 (108.1-219.2)	58.0 (40.8-79.3)	79.8 (54.1-112.2)	10.3 (7.4-13.9)	13.4 (9.6-18.2)	2.3 (1.6-3.3)	20.1 (13.9-27.8)
**CEECA**	42.5	91.4 (62.7-127.3)	163.2 (112.9-225.5)	57.4 (40.0-79.3)	75.7 (51.8-105.9)	10.2 (7.3-13.9)	13.3 (9.5-17.9)	2.3 (1.6-3.2)	19.8 (13.7-27.4)
**CEECA**	47.5	91.0 (62.4-126.9)	165.2 (113.0-230.1)	56.2 (39.5-77.4)	71.6 (48.5-100.2)	10.1 (7.2-13.7)	13.1 (9.3-17.6)	2.3 (1.6-3.2)	19.6 (13.5-27.2)
**CEECA**	52.5	89.1 (61-123.8)	163.4 (112.3-227.8)	54.5 (38.3-75.1)	68.4 (46.5-96.2)	10.1 (7.2-13.7)	12.8 (9-17.6)	2.3 (1.6-3.2)	19.3 (13.4-26.5)
**CEECA**	57.5	87.1 (59.0-123.1)	158.6 (109.5-219.3)	53.1 (37.5-72.4)	65.5 (44.7-92.3)	10.1 (7.2-13.6)	12.5 (8.9-17.0)	2.3 (1.6-3.2)	19.1 (13.3-26.1)
**CEECA**	62.5	84.3 (57.7-118.2)	152.2 (103.7-214.6)	52.0 (36.0-71.1)	63.8 (43.2-89.8)	9.9 (7.1-13.4)	12.3 (8.7-16.6)	2.3 (1.6-3.2)	18.8 (12.9-25.8)
**CEECA**	67.5	82.5 (56.0-116.8)	146.1 (100.2-203.8)	50.9 (35.2-70.1)	61.9 (42.3-86.9)	9.8 (7.0-13.3)	12.1 (8.5-16.4)	2.3 (1.6-3.2)	18.4 (12.9-25.2)
**CEECA**	72.5	80.9 (55.5-112.2)	139.8 (94.4-195.1)	49.9 (35.2-68.3)	59.9 (40.8-84.4)	9.7 (6.9-13.3)	11.9 (8.4-16.2)	2.3 (1.6-3.2)	18.1 (12.6-24.9)
**CEECA**	77.5	79.3 (54.2-111.3)	136 (92.6-192.4)	48.5 (33.7-67.0)	57.9 (39.0-82.2)	9.7 (6.8-13.2)	11.6 (8.2-15.6)	2.3 (1.5-3.1)	18.0 (12.3-25.0)
**CEECA**	82.5	77.5 (53.4-108.4)	131.4 (89.5-182.7)	47.6 (32.9-66.3)	56.6 (38.4-79.2)	9.6 (6.9-13.1)	11.4 (8.1-15.5)	2.3 (1.5-3.2)	17.8 (12.3-24.3)
**CEECA**	87.5	76.2 (51.7-107.3)	128.2 (87.4-178.5)	46.6 (32.2-63.7)	55.3 (37.2-77.0)	9.5 (6.8-12.8)	11.2 (7.9-15.2)	2.2 (1.5-3.1)	17.7 (12.3-24.4)
**CEECA**	92.5	75.1 (50.7-105.8)	124.7 (85.5-174.0)	45.9 (32.1-63.1)	54.2 (36.8-76.5)	9.5 (6.7-12.7)	11.0 (7.8-14.9)	2.2 (1.5-3.1)	17.5 (12.1-24.2)
**CEECA**	97.5	75.1 (51.5-105.9)	121.9 (82.6-171.1)	44.7 (31.4-61.3)	53.0 (36.1-74.4)	9.3 (6.6-12.6)	11.0 (7.8-14.9)	2.2 (1.5-3.1)	17.3 (12.0-23.7)
**HIC**	0.5	89.2 (62.3-123.3)	24.2 (17.3-32.6)	4.3 (2.9-6.2)	4.3 (2.9-5.9)	13.9 (10-18.5)	11.8 (8.4-16.0)	2.3 (1.6-3.2)	4.7 (3.3-6.4)
**HIC**	1.5	72.9 (50.9-99.0)	29.8 (21.1-40.4)	8.5 (5.7-11.9)	9.5 (6.8-13.0)	12.8 (9.2-17.3)	11.4 (8.1-15.3)	2.3 (1.6-3.2)	6.7 (4.7-9.2)
**HIC**	3.5	62.1 (43.7-85.1)	34.8 (24.7-47.4)	14.3 (9.8-20.0)	18.1 (12.7-24.7)	12.0 (8.6-16.3)	11.0 (7.8-14.9)	2.3 (1.6-3.2)	8.8 (6.2-12.1)
**HIC**	7.5	54.2 (38.3-74.7)	40.3 (28.9-54.7)	23.1 (15.8-32.2)	31.9 (22.6-43.4)	11.3 (8.0-15.4)	10.8 (7.7-14.7)	2.3 (1.6-3.2)	11.2 (7.8-15.3)
**HIC**	12.5	53.2 (37.7-72.5)	47.2 (33.7-63.7)	29.7 (20.4-41.8)	45.0 (32.1-61.2)	10.9 (7.8-14.7)	10.7 (7.6-14.6)	2.3 (1.6-3.2)	13.1 (9.2-17.9)
**HIC**	17.5	56.1 (39.2-77.4)	56.6 (40.4-76.9)	32.8 (22.6-45.7)	53.8 (38.1-73.8)	10.8 (7.7-14.6)	10.8 (7.7-14.6)	2.3 (1.6-3.2)	14.3 (10.1-19.4)
**HIC**	22.5	62.3 (43.6-85.9)	67.7 (48.6-90.8)	33.3 (22.7-47.0)	59.1 (41.5-80.4)	10.8 (7.7-14.5)	11.1 (7.9-15.0)	2.3 (1.6-3.2)	15.0 (10.6-20.4)
**HIC**	27.5	69.4 (48.5-95.6)	79.7 (57.0-108.2)	32.8 (22.5-45.8)	62.0 (44.2-83.1)	10.8 (7.8-14.6)	11.2 (7.9-15.4)	2.3 (1.6-3.2)	15.5 (10.9-21.1)
**HIC**	32.5	76.6 (53.3-105.2)	90.6 (64.7-124.1)	32.0 (22.1-44.7)	63.6 (45.4-86.4)	10.8 (7.8-14.7)	11.4 (8.2-15.4)	2.3 (1.6-3.2)	15.8 (11.2-21.3)
**HIC**	37.5	83.0 (57.9-114.1)	99.5 (71.0-134.1)	31.0 (21.0-43.6)	63.9 (45.7-85.5)	10.8 (7.7-14.6)	11.5 (8.2-15.8)	2.3 (1.6-3.3)	16.0 (11.4-21.9)
**HIC**	42.5	88.2 (61.6-122.1)	104.5 (75.2-140.4)	30.3 (20.6-42.5)	63.1 (45.0-85.4)	10.9 (7.7-14.7)	11.6 (8.3-15.7)	2.3 (1.6-3.3)	16.2 (11.4-22.1)

**HIC**	47.5	92.7 (65.5-127.2)	107.2 (77-144.1)	29.3 (20-41.1)	61.8 (44.0-83.5)	10.9 (7.7-14.7)	11.6 (8.3-15.7)	2.3 (1.6-3.2)	16.3 (11.6-22.0)
**HIC**	52.5	95.8 (67.1-1320.0)	106.9 (75.7-143.8)	28.6 (19.6-40.0)	60.7 (43.5-82.8)	10.8 (7.8-14.6)	11.5 (8.2-15.6)	2.3 (1.6-3.3)	16.5 (11.8-22.4)
**HIC**	57.5	98.1 (67.7-136.0)	105 (75.6-141.7)	28.1 (19.3-39.4)	58.8 (41.6-79.7)	10.9 (7.8-14.8)	11.4 (8.1-15.4)	2.3 (1.6-3.3)	16.6 (11.8-22.7)
**HIC**	62.5	100.2 (70.3-137.1)	102.8 (73.7-138.9)	27.7 (18.7-38.6)	57.3 (41.2-77.8)	10.8 (7.8-14.6)	11.4 (8.1-15.3)	2.3 (1.6-3.2)	16.7 (11.9-22.9)
**HIC**	67.5	101.4 (70.7-138.8)	100.1 (71.3-135.8)	27.3 (18.5-38.3)	55.9 (39.9-75.5)	10.9 (7.8-14.7)	11.3 (8.0-15.4)	2.3 (1.6-3.3)	16.8 (12.0-22.9)
**HIC**	72.5	103.7 (73.4-142.8)	97.9 (71.1-130.9)	27.0 (18.4-37.9)	54.4 (38.7-73.9)	10.9 (7.8-14.5)	11.1 (7.9-15.1)	2.3 (1.6-3.2)	16.8 (11.9-22.8)
**HIC**	77.5	104.5 (72.9-143.6)	96.0 (69.0-130.0)	26.5 (18.1-37.1)	53.1 (38.0-71.5)	10.8 (7.6-14.5)	11.1 (7.9-15.2)	2.3 (1.6-3.2)	16.9 (11.9-23.0)
**HIC**	82.5	105.7 (73.7-146.5)	93.9 (67.2-126.7)	26.2 (18.0-36.5)	51.8 (36.9-70.3)	10.9 (7.7-14.7)	11.1 (7.9-15.1)	2.3 (1.6-3.3)	16.9 (11.8-23.2)
**HIC**	87.5	107.5 (74.7-147.9)	92.1 (66.0-125.0)	25.8 (17.6-36.0)	50.7 (35.8-69.0)	10.8 (7.7-14.7)	11.0 (7.8-14.8)	2.3 (1.6-3.3)	17 (12.1-23.3)
**HIC**	92.5	108.7 (75.9-149.6)	90.9 (65.5-122.7)	25.7 (17.6-35.5)	50.0 (35.6-67.5)	10.9 (7.7-14.8)	10.9 (7.8-14.8)	2.3 (1.6-3.2)	17.1 (12.1-23.5)
**HIC**	97.5	109.6 (76.4-149.9)	89.4 (64.0-120.4)	25.5 (17.4-35.9)	48.9 (34.9-66.4)	10.8 (7.7-14.7)	10.8 (7.6-14.7)	2.3 (1.6-3.3)	17.2 (12-23.5)
**LAC**	0.5	16.6 (11.6-22.8)	11.8 (8.1-16.2)	4.6 (3.1-6.7)	1.8 (1.2-2.6)	13.8 (9.8-18.7)	12.1 (8.7-16.4)	2.1 (1.4-3.0)	5.0 (3.4-6.9)
**LAC**	1.5	31.1 (22.0-42.4)	25.7 (17.9-35.3)	9.1 (6.2-12.9)	6.0 (4.2-8.4)	11.3 (8.1-15.4)	10.5 (7.4-14.3)	2.2 (1.5-3.0)	7.0 (4.8-9.5)
**LAC**	3.5	50.9 (36.2-69.0)	46.8 (33.1-63.9)	15.5 (10.5-22.1)	16 (11.0-22.5)	9.5 (6.8-13.0)	9.4 (6.7-12.8)	2.2 (1.5-3.1)	9.1 (6.3-12.6)
**LAC**	7.5	78.4 (55.0-106.9)	79.7 (56.4-109.1)	24.2 (16.3-34.1)	38.3 (26.5-53.2)	8.1 (5.7-11.1)	8.5 (6.0-11.4)	2.3 (1.6-3.2)	11.6 (8.1-16.1)
**LAC**	12.5	96.1 (68.4-130.1)	105.2 (73.7-144.2)	29.7 (20.3-41.1)	57 (38.8-78.7)	7.4 (5.2-10.1)	8.0 (5.7-10.9)	2.3 (1.6-3.3)	13.2 (9.3-18.0)
**LAC**	17.5	100.2 (70.9-135.8)	116.1 (82.1-158.9)	31.4 (21.4-43.9)	60.8 (42.0-85.3)	7.0 (4.9-9.6)	7.9 (5.6-10.7)	2.3 (1.6-3.3)	14.1 (9.9-19.3)
**LAC**	22.5	95.2 (66.7-129.2)	116.4 (81.9-159.4)	30.7 (20.7-43.1)	55.6 (38.2-77.7)	6.9 (4.7-9.4)	8.0 (5.6-10.9)	2.4 (1.6-3.3)	14.5 (10-19.9)
**LAC**	27.5	89.4 (63.7-121.6)	113.2 (79.7-153.7)	29.1 (19.8-41.1)	48.2 (33.1-67.2)	6.9 (4.9-9.4)	8.2 (5.8-11)	2.4 (1.6-3.3)	14.5 (10.2-20)
**LAC**	32.5	84.1 (59.5-113.5)	109.5 (77.8-148.6)	27.3 (18.3-38.9)	42.4 (29.2-58.9)	6.8 (4.8-9.4)	8.3 (5.8-11.4)	2.4 (1.6-3.3)	14.5 (10.1-20.1)
**LAC**	37.5	81.4 (57.4-111.4)	107.6 (75.6-147.3)	25.4 (17.1-35.6)	38.9 (26.7-53.5)	6.8 (4.8-9.3)	8.3 (5.8-11.3)	2.4 (1.6-3.3)	14.4 (10.0-19.8)
**LAC**	42.5	79.8 (56.4-107.9)	106.7 (74.8-145.4)	23.9 (16.0-34.2)	37.2 (25.8-51.7)	6.8 (4.8-9.3)	8.3 (5.9-11.2)	2.4 (1.6-3.3)	14.4 (10.1-19.8)
**LAC**	47.5	80.1 (56.3-108.7)	107.0 (75.7-146.3)	22.5 (15.2-31.6)	37.1 (25.4-52.2)	6.9 (4.9-9.4)	8.2 (5.8-11.3)	2.4 (1.6-3.3)	14.3 (9.9-19.8)
**LAC**	52.5	82.1 (58.4-111.0)	108.9 (76.8-148.2)	21.3 (14.3-29.9)	38.3 (26.9-52.5)	6.9 (4.8-9.5)	8.2 (5.8-11.2)	2.4 (1.6-3.3)	14.1 (9.9-19.5)
**LAC**	57.5	85.2 (60.4-117.3)	111.6 (79.1-152.1)	20.3 (13.8-28.9)	40.8 (28-56.4)	6.9 (4.9-9.5)	8.1 (5.8-11)	2.3 (1.6-3.3)	13.9 (9.7-19)
**LAC**	62.5	88.9 (62.4-121.2)	114.6 (80.8-155.9)	19.2 (13-27.2)	43.5 (30-60.2)	6.9 (4.9-9.6)	7.9 (5.6-10.8)	2.3 (1.6-3.3)	13.8 (9.5-19.1)
**LAC**	67.5	92.2 (65.2-126.3)	117.9 (84-160)	18.3 (12.5-25.9)	46.2 (31.9-64.7)	6.9 (5-9.4)	7.8 (5.5-10.7)	2.3 (1.6-3.2)	13.7 (9.6-19)
**LAC**	72.5	95.6 (68.1-129.5)	121 (85.2-164.3)	17.8 (12-25.2)	49.1 (33.7-68.1)	7 (5-9.6)	7.7 (5.4-10.5)	2.3 (1.6-3.2)	13.6 (9.5-18.8)
**LAC**	77.5	98.8 (69.9-133.2)	122.7 (86.6-167.3)	16.9 (11.4-23.9)	51.7 (36-71.9)	7 (5-9.7)	7.6 (5.4-10.3)	2.3 (1.6-3.2)	13.5 (9.3-18.7)
**LAC**	82.5	101.7 (71.4-137.6)	125.6 (88.5-173.8)	16.4 (11-23.2)	54.5 (37.6-75.4)	7 (4.9-9.6)	7.5 (5.3-10.2)	2.3 (1.6-3.2)	13.3 (9.3-18.4)

**LAC**	87.5	104.3 (73.5-142.2)	127.2 (89.9-172.6)	15.8 (10.6-22.3)	57.3 (39.4-79.6)	7 (5-9.4)	7.4 (5.2-10.1)	2.3 (1.6-3.2)	13.2 (9.2-18.2)
**LAC**	92.5	106.8 (75.7-145.8)	129.5 (90.3-179.2)	15.3 (10.3-21.6)	59.7 (40.8-83.4)	7 (4.9-9.5)	7.3 (5.1-10)	2.3 (1.6-3.2)	13.2 (9.1-18.2)
**LAC**	97.5	109.5 (77.3-149.5)	131.4 (91.7-179.7)	14.7 (9.9-20.7)	61.6 (42.7-84.9)	6.9 (4.9-9.4)	7.3 (5.1-10.1)	2.3 (1.6-3.2)	13 (9-18)
**MENA**	0.5	8.7 (6.1-12.0)	4.4 (3.0-6.1)	3.8 (2.4-5.6)	0.9 (0.6-1.4)	7.3 (5.1-9.9)	8.4 (5.9-11.4)	2.6 (1.8-3.6)	5.9 (3.8-8.5)
**MENA**	1.5	19.1 (13.3-26.4)	12.9 (9-17.6)	7.4 (4.8-10.8)	3.5 (2.4-4.9)	7.6 (5.4-10.3)	8.9 (6.3-12.1)	2.5 (1.7-3.5)	8.9 (5.9-12.7)
**MENA**	3.5	35 .0(24.5-47.6)	30.2 (21.3-41.4)	12.3 (8.0-17.7)	10.5 (7.3-14.6)	7.8 (5.6-10.7)	9.4 (6.6-13)	2.4 (1.6-3.3)	12.5 (8.4-17.7)
**MENA**	7.5	60.1 (42-82.5)	64.8 (45.5-88.8)	19.2 (12.6-27.7)	28.8 (20-39.9)	8 (5.6-10.9)	9.9 (6.9-13.5)	2.4 (1.6-3.3)	16.9 (11.6-23.5)
**MENA**	12.5	80.5 (57.4-110.1)	99.8 (70.6-136.3)	23.9 (15.9-34.4)	46.1 (32-64)	8.2 (5.8-11.1)	10.3 (7.3-14.2)	2.3 (1.6-3.2)	20.3 (14.2-27.9)
**MENA**	17.5	89.4 (62.8-121.1)	120.3 (85.4-165.6)	25.3 (16.7-36.4)	50.4 (35.1-69.7)	8.4 (6-11.4)	10.9 (7.6-15.0)	2.3 (1.6-3.3)	23.4 (16.1-32.7)
**MENA**	22.5	91.2 (63.5-125.1)	129.2 (91.0-175.7)	25.2 (16.6-36.5)	45.5 (31.6-63.1)	8.6 (6.1-11.7)	11.4 (8.1-15.6)	2.4 (1.6-3.3)	24.9 (17.1-34.8)
**MENA**	27.5	89.8 (63.4-122.1)	131 (92.2-179)	24.5 (16.2-35.4)	38.4 (27.0-53.0)	8.7 (6.2-11.7)	11.9 (8.4-16.1)	2.4 (1.7-3.3)	25.6 (17.6-35.3)
**MENA**	32.5	88.7 (63-120.2)	131.9 (93.2-179.2)	23 (15.2-33.4)	33.2 (22.7-45.8)	8.7 (6.2-11.9)	12.2 (8.6-16.6)	2.4 (1.7-3.4)	26 .0(17.9-36.5)
**MENA**	37.5	88.2 (61.6-121.7)	132.9 (94.9-179.9)	21.4 (14-30.7)	29.5 (20.8-40.5)	8.7 (6.2-11.8)	12.3 (8.7-16.7)	2.4 (1.7-3.3)	26 .0(18.2-36.1)
**MENA**	42.5	88.4 (61.8-120.5)	134.3 (94.3-182.7)	19.8 (13-28.6)	27.5 (19.3-37.7)	8.6 (6.2-11.7)	12.2 (8.6-16.6)	2.4 (1.7-3.4)	25.9 (17.7-35.9)
**MENA**	47.5	89.7 (62.7-122.2)	135.8 (97.4-184.1)	18.4 (12.3-26.6)	26.7 (18.4-37)	8.6 (6.1-11.8)	12 (8.5-16.5)	2.4 (1.6-3.3)	25.7 (17.9-35.9)
**MENA**	52.5	91.6 (64.8-125)	138.6 (98.8-187.7)	17.3 (11.3-25)	27.2 (18.9-37.3)	8.6 (6.1-11.6)	11.6 (8.2-15.9)	2.4 (1.7-3.3)	25.6 (17.5-35.6)
**MENA**	57.5	94.8 (67.2-129.7)	141.6 (99.7-192.4)	16 (10.5-22.9)	28.2 (19.9-38.9)	8.4 (6-11.4)	11.3 (8.1-15.3)	2.4 (1.6-3.3)	25.2 (17.3-34.8)
**MENA**	62.5	98.1 (69.5-132.1)	146 (103.3-197.7)	14.7 (9.7-21.1)	29.4 (20.8-40)	8.2 (5.8-11.2)	10.8 (7.6-14.8)	2.4 (1.6-3.3)	25.1 (17.3-34.8)
**MENA**	67.5	101.1 (71.6-137.3)	149.9 (106.1-204.9)	14.0 (9.2-20.3)	30.9 (21.7-42.8)	8.1 (5.8-11)	10.4 (7.3-14.1)	2.4 (1.6-3.3)	24.9 (17.1-34.8)
**MENA**	72.5	104.8 (73.4-144)	154 (109.7-209.4)	12.9 (8.5-18.6)	32.3 (22.6-44.2)	8.0 (5.7-11)	10.1 (7.1-13.8)	2.3 (1.6-3.3)	24.8 (17.3-34.2)
**MENA**	77.5	107.4 (75.9-145.3)	157.3 (112.6-213.2)	12.2 (8.1-17.4)	33.6 (23.7-45.9)	7.9 (5.6-10.7)	9.8 (6.9-13.4)	2.3 (1.6-3.2)	24.8 (17.2-34.5)
**MENA**	82.5	110.5 (77.3-152.3)	160.2 (112.1-219.6)	11.5 (7.5-16.6)	35 (24.5-47.9)	7.7 (5.5-10.7)	9.5 (6.6-13)	2.3 (1.6-3.2)	24.7 (17.1-34.0)
**MENA**	87.5	112.3 (78.7-152.3)	163.7 (115.8-220.9)	10.6 (7-15.3)	36.1 (25.5-49.2)	7.6 (5.3-10.5)	9.2 (6.5-12.6)	2.3 (1.6-3.2)	24.9 (17.0-35.0)
**MENA**	92.5	114.3 (80.9-155.9)	167.6 (117.6-228.8)	9.8 (6.3-14.3)	36.8 (25.9-49.7)	7.6 (5.4-10.3)	8.9 (6.2-12.2)	2.3 (1.6-3.2)	25.4 (17.6-34.9)
**MENA**	97.5	116.7 (83.1-159.7)	170.5 (120-232.3)	8.9 (5.9-13)	37.5 (26.4-50.9)	7.5 (5.3-10.1)	8.7 (6.1-11.8)	2.3 (1.6-3.3)	25.9 (17.9-36)
**SAARC**	0.5	13.1 (8.9-18.7)	5.8 (4-8.1)	1.1 (0.7-1.7)	1.1 (0.7-1.6)	10.3 (7.3-14)	8.2 (5.8-11.2)	2.2 (1.5-3.1)	10.8 (7.4-15.2)
**SAARC**	1.5	26.3 (17.6-37)	18.4 (13.0-25.0)	1.8 (1.1-2.8)	2.9 (1.9-4.1)	8.1 (5.7-11.2)	7.1 (4.9-9.7)	2.2 (1.5-3.1)	15.4 (10.6-21.5)
**SAARC**	3.5	45.3 (30.7-64.1)	45.2 (32-61.2)	2.8 (1.7-4.2)	6.7 (4.6-9.4)	6.7 (4.7-9.1)	6.2 (4.4-8.5)	2.2 (1.5-3.1)	19.7 (13.3-27.7)
**SAARC**	7.5	73.3 (50-103.4)	100.2 (71.6-135.4)	3.9 (2.4-6.0)	14.4 (9.9-20.0)	5.6 (3.9-7.6)	5.5 (3.8-7.6)	2.2 (1.5-3.1)	23.8 (16.6-32.8)
**SAARC**	12.5	92.7 (62.6-129.7)	154.7 (108.4-211.3)	4.6 (2.9-7.0)	21.0 (14.4-29.1)	5 (3.4-6.9)	5.2 (3.6-7.1)	2.2 (1.5-3.1)	26.6 (18.6-36.7)

**SAARC**	17.5	96.4 (65.2-134.7)	183.7 (131.3-247.7)	4.6 (2.8-7.0)	22.8 (15.4-31.6)	4.8 (3.3-6.7)	5 (3.5-7.0)	2.2 (1.5-3.1)	30.9 (21.4-43.2)
**SAARC**	22.5	90.6 (61.5-127.1)	190.9 (135.2-259.5)	4.3 (2.7-6.4)	21.6 (15.0-30.0)	4.7 (3.2-6.5)	5 (3.5-7.0)	2.2 (1.5-3.2)	31.6 (22.0-43.9)
**SAARC**	27.5	83 (56.2-118)	187.4 (134.7-254.1)	3.8 (2.3-5.9)	19.5 (13.2-27.4)	4.6 (3.2-6.4)	5 (3.4-6.9)	2.2 (1.5-3.1)	31.5 (21.7-44.1)
**SAARC**	32.5	77.8 (53-110)	181.2 (128.3-245.7)	3.5 (2.1-5.4)	17.6 (12-24.5)	4.7 (3.3-6.5)	5 (3.5-6.9)	2.2 (1.5-3.1)	31.9 (21.9-44.4)
**SAARC**	37.5	75 (51-105.5)	174.8 (123.2-235.5)	3.4 (2.1-5.1)	16.1 (10.9-22.6)	4.7 (3.3-6.5)	5 (3.5-6.9)	2.2 (1.5-3.1)	32.1 (22.1-44.3)
**SAARC**	42.5	73.8 (50.3-104.3)	170 (121.6-230.5)	3.3 (2-4.9)	15.3 (10.4-21.7)	4.8 (3.3-6.7)	5.1 (3.6-7.1)	2.2 (1.5-3.1)	32.3 (22.0-45.0)
**SAARC**	47.5	73.5 (49.8-103)	165.9 (118.4-224.4)	3.3 (1.9-5)	14.7 (10.1-20.3)	4.9 (3.4-6.7)	5.2 (3.6-7.1)	2.2 (1.5-3.1)	32.3 (22.7-45.1)
**SAARC**	52.5	74.4 (50.3-105.2)	164 (115.9-222.4)	3.3 (2.0-5.0)	14.3 (9.8-19.9)	5 .0(3.4-6.9)	5.2 (3.6-7.2)	2.2 (1.5-3.1)	32.6 (22.6-45)
**SAARC**	57.5	76.7 (52.1-108.4)	162 (113.2-221.9)	3.4 (2.1-5.2)	14.2 (9.8-19.8)	5 .0(3.5-6.9)	5.2 (3.6-7.2)	2.2 (1.5-3.1)	32.5 (22.2-44.8)
**SAARC**	62.5	78.7 (53.3-111.2)	160.3 (113.4-217.6)	3.5 (2.2-5.4)	14.3 (9.8-20.2)	5.1 (3.5-7.1)	5.3 (3.6-7.4)	2.3 (1.5-3.2)	32.7 (22.6-45.2)
**SAARC**	67.5	81.6 (55-115.8)	159.2 (112.7-214.4)	3.6 (2.2-5.5)	14.4 (9.7-20.1)	5.1 (3.5-7.2)	5.3 (3.7-7.3)	2.2 (1.5-3.1)	32.9 (23-45.2)
**SAARC**	72.5	83.7 (55.9-117.8)	159.3 (113.6-215.5)	3.7 (2.3-5.7)	14.4 (9.9-20.2)	5.2 (3.6-7.2)	5.4 (3.8-7.5)	2.3 (1.6-3.2)	33 (22.7-45.9)
**SAARC**	77.5	84.6 (57.7-120.1)	158.6 (112.5-214.6)	3.7 (2.3-5.6)	14.5 (9.9-20.1)	5.3 (3.6-7.3)	5.4 (3.8-7.5)	2.3 (1.6-3.1)	32.8 (22.8-45.4)
**SAARC**	82.5	85 (57.6-120.6)	156.6 (110.5-212.2)	3.7 (2.3-5.7)	14.4 (9.8-20)	5.3 (3.7-7.4)	5.4 (3.7-7.4)	2.3 (1.5-3.1)	32.6 (22.6-44.9)
**SAARC**	87.5	85.5 (58-119.6)	155.8 (111.2-209.7)	3.8 (2.3-5.7)	14.4 (9.8-20.1)	5.3 (3.7-7.4)	5.4 (3.7-7.5)	2.3 (1.5-3.1)	32.7 (22.6-45.5)
**SAARC**	92.5	84.9 (58-119.1)	153.3 (108.6-209)	3.7 (2.3-5.7)	14.2 (9.8-19.9)	5.3 (3.7-7.4)	5.4 (3.7-7.3)	2.3 (1.6-3.1)	32.4 (22.5-44.9)
**SAARC**	97.5	82.9 (56.1-117.3)	150.9 (107.1-205.4)	3.6 (2.2-5.5)	14.1 (9.6-19.6)	5.3 (3.7-7.3)	5.3 (3.7-7.3)	2.3 (1.6-3.1)	31.9 (22-44.4)
**SSA**	0.5	8.1 (5.5-11.3)	8.8 (6.0-12.2)	2.3 (1.5-3.5)	0.7 (0.4-1.1)	9.5 (6.7-12.9)	9.8 (7-13.3)	2.3 (1.6-3.2)	7.4 (5.2-10.3)
**SSA**	1.5	17.7 (12.2-24.4)	21.7 (15.1-30.1)	4.3 (2.7-6.3)	2.3 (1.5-3.2)	8.7 (6.1-11.7)	9.5 (6.7-12.9)	2.3 (1.6-3.2)	10.9 (7.5-15.1)
**SSA**	3.5	33.1 (23.0-46.0)	43.6 (30.0-59.9)	6.9 (4.4-10.3)	6.0 (4.0-8.5)	8.1 (5.7-11)	9.2 (6.5-12.5)	2.3 (1.6-3.3)	14.5 (10-20.2)
**SSA**	7.5	57.6 (39.8-80.3)	81.4 (56.5-112)	10.5 (6.8-15.6)	14.9 (10.1-20.8)	7.6 (5.4-10.4)	9 (6.3-12.4)	2.4 (1.6-3.3)	18.5 (12.9-25.4)
**SSA**	12.5	76.1 (52.6-106.1)	112.9 (78.5-154)	12.6 (8.1-18.4)	22.1 (15.3-30.8)	7.4 (5.2-10)	9 (6.4-12.4)	2.4 (1.6-3.3)	21.1 (14.7-29.0)
**SSA**	17.5	82.7 (57.7-113.6)	127.0 (88.0-176.2)	12.8 (8.2-18.8)	22.8 (15.4-31.9)	7.4 (5.2-10)	9.2 (6.5-12.6)	2.4 (1.6-3.3)	23.9 (16.6-32.6)
**SSA**	22.5	81.8 (57.2-112.8)	127.2 (87.8-174.8)	12 (7.8-17.5)	19.7 (13.6-27.7)	7.4 (5.2-10.1)	9.4 (6.7-12.8)	2.3 (1.6-3.2)	24.9 (17.2-34.3)
**SSA**	27.5	78.5 (55.2-108.4)	123.9 (86-170.9)	11.1 (7.1-16.2)	16.3 (11.1-22.7)	7.5 (5.3-10.3)	9.6 (6.9-13.1)	2.3 (1.6-3.3)	25.4 (17.8-35.1)
**SSA**	32.5	74.7 (51.5-103.2)	119.2 (83.1-164.2)	10.1 (6.4-14.9)	14.3 (9.7-20.2)	7.6 (5.3-10.4)	9.9 (6.9-13.5)	2.3 (1.6-3.3)	25.6 (17.9-35.4)
**SSA**	37.5	71.5 (50.3-98.7)	115.8 (80.5-158.4)	9.2 (5.8-13.4)	13.5 (9.1-19.1)	7.7 (5.4-10.4)	10 (7.1-13.7)	2.3 (1.6-3.2)	25.6 (18.0-35.3)
**SSA**	42.5	69.7 (48.7-95.8)	113.5 (78.8-156.6)	8.4 (5.4-12.4)	13.7 (9.2-19.5)	7.8 (5.5-10.5)	10.2 (7.2-14)	2.3 (1.6-3.2)	25.8 (18.1-35.1)
**SSA**	47.5	68.6 (48.1-94.8)	112.9 (78.6-155.7)	7.8 (4.9-11.4)	15 (10.2-21.2)	7.8 (5.5-10.7)	10.3 (7.3-14.1)	2.3 (1.6-3.2)	25.6 (17.7-35.3)
**SSA**	52.5	68.4 (47.1-94.4)	113.4 (78.3-157.7)	7.2 (4.6-10.6)	17.2 (11.6-24.2)	7.9 (5.6-10.8)	10.3 (7.3-13.9)	2.3 (1.6-3.2)	25.4 (17.6-35.2)

**SSA**	57.5	68.5 (47.3-95.4)	114.1 (78.5-156.6)	6.8 (4.3-10.1)	20.6 (13.8-29.3)	8 (5.6-10.8)	10.2 (7.2-14.1)	2.3 (1.6-3.2)	25.0 (17.4-34.2)
**SSA**	62.5	69.7 (48-97)	115.6 (80.7-159.3)	6.4 (4.1-9.5)	24.9 (16.6-34.9)	8 (5.7-11.0)	10.3 (7.3-13.9)	2.3 (1.6-3.2)	24.9 (17.2-34.3)
**SSA**	67.5	70.1 (48.9-95.7)	117.4 (81.5-161.2)	6.1 (3.9-9.2)	29.7 (20.4-41.5)	8.1 (5.7-11.0)	10.4 (7.3-14)	2.3 (1.6-3.2)	24.7 (17.1-34)
**SSA**	72.5	70.8 (49.6-97.9)	119.5 (83.1-165.5)	5.8 (3.7-8.6)	35.3 (24-49.5)	8.2 (5.8-11.1)	10.3 (7.4-14)	2.3 (1.6-3.2)	24.5 (17.1-33.5)
**SSA**	77.5	71.3 (50.1-98.7)	120.2 (83.8-164)	5.6 (3.5-8.2)	41.3 (28.5-57.6)	8.2 (5.8-11.2)	10.4 (7.3-14.1)	2.3 (1.6-3.2)	24.2 (16.9-33.2)
**SSA**	82.5	71.7 (49.4-98.8)	121.2 (84.9-167.1)	5.3 (3.4-7.9)	47.4 (32.1-65.9)	8.2 (5.8-11.2)	10.4 (7.3-14)	2.3 (1.6-3.2)	23.9 (16.6-32.9)
**SSA**	87.5	71.9 (50.2-98.1)	122.3 (85.2-169.2)	5.2 (3.3-7.8)	54 (37-75.6)	8.2 (5.8-11.2)	10.4 (7.4-14)	2.3 (1.5-3.2)	23.8 (16.5-33)
**SSA**	92.5	71.9 (50.3-98.6)	121.7 (84.2-168)	4.9 (3.1-7.3)	61 (41.7-85.8)	8.3 (5.8-11.2)	10.3 (7.4-14)	2.3 (1.5-3.2)	23.5 (16.5-32.4)
**SSA**	97.5	71.3 (49.1-99)	119.6 (81.5-166.4)	4.9 (3.1-7.1)	68.2 (46.2-95.6)	8.3 (5.9-11.2)	10.4 (7.4-14)	2.3 (1.5-3.1)	23.2 (16.2-31.9)
**world**	0.5	22.2 (14.3-32.6)	13.5 (9.0-19.1)	4.6 (2.8-6.7)	2.1 (1.3-3.2)	10.6 (7.4-14.5)	10.1 (7.1-14)	2.2 (1.5-3.1)	5.4 (3.6-7.8)
**world**	1.5	31.8 (21.5-44.5)	25.6 (17.4-35.8)	8.6 (5.6-12.7)	6.1 (4.0-8.9)	9.9 (7-13.4)	9.7 (6.9-13.2)	2.3 (1.6-3.2)	7.9 (5.4-11.1)
**world**	3.5	44.4 (30.6-61.6)	43.2 (30.2-59.3)	14.1 (9-20.4)	14.3 (9.4-20.7)	9.4 (6.6-12.9)	9.5 (6.7-12.9)	2.3 (1.6-3.2)	10.6 (7.2-14.8)
**world**	7.5	62.2 (43.6-86)	70.7 (49.1-98.0)	21.4 (13.9-31.1)	31.2 (20.9-44.5)	9 (6.3-12.3)	9.3 (6.5-12.7)	2.3 (1.6-3.2)	13.6 (9.4-19.0)
**world**	12.5	75.2 (52.4-103.2)	94.4 (65.1-130.2)	25.8 (16.8-37.3)	45.4 (30.8-63.5)	8.7 (6.1-12)	9.4 (6.6-12.8)	2.3 (1.6-3.2)	15.9 (10.9-22.2)
**world**	17.5	80 .0(55.5-110.2)	109 (75.6-151.6)	26.7 (17.3-38.9)	49.6 (32.9-70.5)	8.6 (6.1-11.8)	9.5 (6.6-13)	2.3 (1.6-3.3)	17.7 (12.0-24.7)
**world**	22.5	80.5 (56-111.1)	116.5 (80.8-161)	25.6 (16.7-37.2)	47 (30.8-68.1)	8.6 (6-11.8)	9.6 (6.8-13.2)	2.3 (1.6-3.2)	18.5 (12.6-25.7)
**world**	27.5	79.5 (55.4-109.6)	120.8 (83.3-165.5)	23.9 (15.5-35.2)	43 (28.7-61.4)	8.7 (6.1-11.9)	9.8 (6.9-13.5)	2.3 (1.6-3.3)	18.8 (12.9-25.9)
**world**	32.5	77.7 (54.2-106.7)	123.1 (86.5-168)	22.3 (14.3-32.4)	39.5 (26.2-56.6)	8.7 (6.1-11.9)	10 .0(7.0-13.6)	2.3 (1.6-3.3)	19.0 (13.2-26.5)
**world**	37.5	77.3 (53.9-107.3)	125 (87.5-171.8)	20.9 (13.3-31.1)	36.9 (24.2-53.5)	8.7 (6.1-11.9)	10 .0(7-13.6)	2.3 (1.6-3.3)	19.1 (13.2-26.7)
**world**	42.5	76.6 (52.8-106.9)	125.6 (87.8-174.4)	19.6 (12.2-29.4)	35.3 (23-50.5)	8.6 (6.0-11.9)	10 .0(7-13.7)	2.3 (1.6-3.3)	19.1 (13.1-26.7)
**world**	47.5	77.4 (54.5-107.1)	125.6 (88-173)	18.4 (11.3-27.3)	34.6 (22.7-50.2)	8.6 (6.0-11.8)	9.9 (6.9-13.6)	2.3 (1.6-3.2)	19.2 (13.3-27.1)
**world**	52.5	78.3 (54.6-108.7)	126.2 (88.9-174.1)	17.6 (10.9-26.6)	34.7 (23.2-49.9)	8.6 (6.0-11.7)	9.8 (6.8-13.4)	2.3 (1.6-3.2)	19.1 (13-26.9)
**world**	57.5	79.4 (54.8-109.7)	126.4 (88.7-173.3)	16.6 (10.3-24.9)	35.5 (23.7-50.1)	8.5 (6.0-11.6)	9.6 (6.7-13.2)	2.3 (1.6-3.2)	19.0 (13.0-26.7)
**world**	62.5	81.3 (56.6-112.8)	127.1 (88.8-174.5)	15.9 (9.8-24.2)	36.9 (24.7-52.1)	8.5 (6.0-11.5)	9.4 (6.5-13.0)	2.3 (1.6-3.2)	18.9 (12.9-26.5)
**world**	67.5	83.1 (57.7-114.9)	127.3 (89.4-172.6)	15.3 (9.6-23)	38.5 (26.1-54.6)	8.4 (5.9-11.4)	9.3 (6.5-12.8)	2.3 (1.6-3.2)	18.9 (12.9-26.4)
**world**	72.5	84.4 (58.5-115.9)	127.8 (90.6-173.8)	14.9 (9.1-22.6)	40.1 (27.4-55.6)	8.3 (5.9-11.3)	9.1 (6.3-12.4)	2.3 (1.6-3.2)	18.7 (13-25.9)
**world**	77.5	85.6 (59.3-118.9)	128.1 (89.3-176.5)	14.2 (8.8-21.2)	41.9 (28.3-58.5)	8.3 (5.8-11.2)	9.0 (6.3-12.3)	2.3 (1.6-3.2)	18.6 (12.8-25.7)
**world**	82.5	87.2 (60.7-120.8)	128.5 (89.5-175.7)	13.6 (8.3-20.7)	43.6 (29.3-61.5)	8.3 (5.8-11.3)	8.8 (6.2-12.2)	2.3 (1.6-3.2)	18.6 (12.8-25.6)
**world**	87.5	88.3 (61.1-122.5)	128.9 (90.1-177.6)	13.0 (8-19.7)	45.4 (30.4-63.6)	8.2 (5.8-11.2)	8.7 (6.1-12.0)	2.3 (1.6-3.2)	18.5 (12.5-25.9)
**world**	92.5	89.2 (62.8-123.5)	129.4 (89.8-178.4)	12.7 (7.8-19.3)	47.5 (32.3-66.7)	8.1 (5.7-11.1)	8.7 (6.0-11.9)	2.3 (1.5-3.2)	18.5 (12.7-25.6)

**world**	97.5	90.2 (62.2-124.9)	129.1 (90.6-177.6)	12.4 (7.6-18.9)	49.1 (33.0-69.9)	8.1 (5.7-11)	8.5 (5.9-11.9)	2.3 (1.5-3.2)	18.6 (12.7-25.8)

In previous global dietary database reports, Central Europe, Eastern Europe, and Central Asia were referred to as the former Soviet Union, while Southeast Asia and East Asia were collectively called Asia. UI, Uncertainty Interval. Midpoint ages correspond to specific age groups. Ages 0.5, 1.5, and 3.5 represent 0–1 year, 1–2 years, and 3–4 years, respectively. From 7.5 onwards, midpoint ages represent standard 5-year intervals (e.g. 5–9 years, 10–14 years, etc.).

Supplementary Table 10: National mean (95% UI) intakes of eight nutrients among females in 2018 across 185 countries, stratified by area of residence for all age groups.

Region	Age	Fruits	Non-starchy vegetables	Total processed meats	Unprocessed red meats	Saturated fat	Monounsaturated fatty acids	Total omega-6 fat	Dietary fiber
**Asia**	0.5	34.3 (23.6-47.7)	29.6 (20.9-40.2)	6.6 (4.5-9.4)	4.7 (3.1-6.6)	13.9 (9.8-19)	10.0 (7.1-13.6)	2.3 (1.6-3.2)	2 .0(1.3-2.8)
**Asia**	1.5	49.5 (33.8-68.2)	49.9 (35.1-68.7)	12.3 (8.3-17.4)	10.6 (7.2-15.1)	13.4 (9.5-18.2)	9.2 (6.4-12.5)	2.3 (1.6-3.2)	3.1 (2.1-4.4)
**Asia**	3.5	65.5 (45.2-91)	74.6 (52.8-101.2)	19.4 (13.1-27.3)	20.1 (13.6-27.7)	12.9 (9.2-17.6)	8.6 (6.1-11.6)	2.3 (1.6-3.2)	4.5 (3.0-6.2)
**Asia**	7.5	83.9 (57.8-115.8)	106.7 (75.6-146.2)	28.4 (19.5-39.2)	35.3 (24.3-49.8)	12.5 (8.9-17)	8.1 (5.7-11.0)	2.3 (1.6-3.2)	6.2 (4.2-8.6)
**Asia**	12.5	92.6 (64.3-127.7)	130.7 (91.3-179.5)	30.1 (20.6-42.3)	46.3 (31.6-64.5)	12.3 (8.7-16.7)	7.9 (5.6-10.7)	2.3 (1.6-3.3)	7.7 (5.3-10.6)
**Asia**	17.5	91 (62.8-125.4)	143.4 (101.1-198.4)	25.8 (17.6-36.2)	49.7 (34.4-68.2)	12.2 (8.5-16.6)	7.7 (5.5-10.5)	2.3 (1.6-3.2)	8.7 (6.0-12.0)
**Asia**	22.5	85.1 (58.5-117.7)	148 (103-202.2)	20 (13.6-28.2)	48.6 (33.4-67.1)	12.1 (8.5-16.4)	7.6 (5.5-10.5)	2.3 (1.6-3.2)	9.6 (6.6-13.2)
**Asia**	27.5	79.2 (54.2-109.1)	150 (104.9-206.8)	14.8 (10-21.1)	45.4 (31-63.3)	11.9 (8.4-16.1)	7.6 (5.4-10.3)	2.3 (1.6-3.2)	10.2 (7.1-14.1)
**Asia**	32.5	75.4 (51.7-104.5)	149.8 (105.7-204.4)	11.3 (7.5-16.1)	41.9 (28.5-58.6)	11.7 (8.3-15.8)	7.4 (5.2-10.1)	2.3 (1.6-3.2)	10.7 (7.4-14.8)
**Asia**	37.5	73.5 (50.9-102.4)	149.7 (104.6-206.1)	9 (5.9-12.8)	38.6 (26.3-53.6)	11.5 (8.1-15.8)	7.2 (5.1-9.8)	2.3 (1.6-3.2)	11 (7.6-15.3)
**Asia**	42.5	73.3 (50.6-101.9)	149.4 (105.2-203.8)	7.5 (5.1-10.6)	35.5 (24.2-49.5)	11.2 (7.9-15.5)	7 (4.9-9.5)	2.3 (1.6-3.2)	11.4 (7.8-15.8)
**Asia**	47.5	74.9 (51.7-103.6)	149.2 (105.5-203.6)	6.6 (4.4-9.4)	33.3 (22.8-46.5)	10.9 (7.7-14.8)	6.6 (4.7-9)	2.3 (1.6-3.2)	11.6 (8-16)
**Asia**	52.5	78.1 (53.9-107.5)	149.6 (105.6-203.6)	6.1 (4.1-8.7)	31 (21.1-43.5)	10.6 (7.5-14.5)	6.3 (4.5-8.6)	2.3 (1.6-3.2)	11.7 (8.2-16.1)
**Asia**	57.5	82.4 (57.1-115)	150.4 (106.3-204.6)	5.7 (3.8-8.1)	29 .0(19.7-40.1)	10.2 (7.2-14.0)	6 .0(4.2-8.2)	2.3 (1.6-3.2)	11.7 (8.1-16.3)
**Asia**	62.5	88.2 (60.7-123.5)	150.9 (105.7-208.8)	5.5 (3.7-7.8)	27.5 (18.6-38.7)	9.8 (6.9-13.4)	5.7 (4.1-7.7)	2.3 (1.6-3.1)	11.8 (8.2-16.1)
**Asia**	67.5	93.5 (63.7-130.7)	152.3 (107.6-206.8)	5.3 (3.5-7.5)	26.1 (17.6-37.1)	9.6 (6.6-13.3)	5.4 (3.8-7.3)	2.3 (1.6-3.2)	11.7 (8.1-16.2)
**Asia**	72.5	98.7 (68.1-136.2)	152.2 (107.7-207.6)	5.1 (3.4-7.3)	24.9 (16.7-34.9)	9.2 (6.5-12.5)	5.1 (3.6-6.9)	2.3 (1.6-3.2)	11.8 (8.2-16.2)
**Asia**	77.5	104 (72.8-143.6)	153 (108.2-209.9)	4.9 (3.3-7.1)	23.7 (15.9-33.3)	9.0 (6.3-12.2)	4.9 (3.4-6.7)	2.3 (1.5-3.1)	11.9 (8.1-16.5)
**Asia**	82.5	108.6 (74.1-151.5)	153.6 (108.1-211.5)	4.8 (3.2-6.9)	22.7 (15.6-31.7)	8.7 (6.2-12)	4.7 (3.2-6.4)	2.2 (1.5-3.1)	11.8 (8.2-16.3)
**Asia**	87.5	113.3 (78.5-157.2)	153.1 (108.4-209.1)	4.7 (3.1-6.8)	21.7 (14.8-30.4)	8.6 (6.0-11.7)	4.4 (3.1-6.1)	2.2 (1.5-3.1)	11.7 (8.1-16.2)
**Asia**	92.5	117.3 (80.5-162)	154 (109.1-211.6)	4.6 (3.0-6.6)	20.8 (14-29.3)	8.4 (5.9-11.5)	4.3 (3.0-5.8)	2.2 (1.5-3.1)	11.6 (8.1-16)
**Asia**	97.5	122.7 (85.4-171.8)	153.9 (109.5-210.4)	4.6 (3.0-6.5)	19.8 (13.3-27.9)	8.2 (5.8-11.2)	4.1 (2.9-5.6)	2.2 (1.5-3.1)	11.6 (7.9-16.2)
**CEECA**	0.5	25.7 (17.5-36.5)	16.4 (11.0-23.1)	7.1 (4.7-10.4)	3.1 (2.0-4.4)	7.3 (5.2-9.8)	7.9 (5.5-10.8)	2.0 (1.3-2.8)	6.8 (4.6-9.5)
**CEECA**	1.5	39.1 (26.6-54.7)	27.7 (18.6-39.5)	13.6 (9.1-19.4)	9.6 (6.4-13.8)	8.1 (5.7-11.1)	8.9 (6.3-12.2)	2.1 (1.4-2.9)	9.7 (6.7-13.5)
**CEECA**	3.5	54.1 (37.3-74.9)	41.6 (27.9-59.4)	22 (14.9-31)	23.6 (15.9-33.5)	8.9 (6.4-12)	9.8 (6.9-13.5)	2.2 (1.5-3.0)	12.9 (9.0-17.9)

**CEECA**	7.5	72.1 (48.3-102)	59.8 (40.4-84.4)	33.2 (22.8-46.5)	52.3 (35.4-74)	9.5 (6.7-12.9)	10.6 (7.5-14.3)	2.3 (1.6-3.1)	16.4 (11.3-22.6)
**CEECA**	12.5	85.6 (59.5-119.8)	79.4 (53.8-112.1)	41.7 (28.9-58.3)	76.4 (52.1-107)	10 .0(7.2-13.5)	11.3 (8.1-15.4)	2.3 (1.6-3.2)	18.4 (12.9-25.2)
**CEECA**	17.5	94.2 (64.3-131.9)	101.9 (68.7-144.6)	46 (32.4-63.8)	86.9 (59.2-121.5)	10.2 (7.2-13.9)	11.7 (8.2-15.9)	2.3 (1.6-3.2)	20.1 (14.0-27.8)
**CEECA**	22.5	102.1 (70.1-142.2)	125.9 (85.3-178.4)	47.7 (33.4-65.8)	88.7 (61.3-122.9)	10.4 (7.4-14)	12.1 (8.6-16.3)	2.3 (1.6-3.3)	20.6 (14.2-28.4)
**CEECA**	27.5	106.9 (73.8-149.8)	147.3 (99.5-204.9)	48.6 (33.9-67.1)	85.2 (58.7-119.2)	10.5 (7.5-14.1)	12.4 (8.8-16.8)	2.4 (1.6-3.3)	20.6 (14.3-28.3)
**CEECA**	32.5	109.8 (76.4-153.9)	163.2 (112.1-225.6)	48.5 (33.7-67.2)	80.3 (54.7-111.8)	10.6 (7.6-14.3)	12.6 (9.0-17.1)	2.4 (1.6-3.3)	20.3 (14-28.3)
**CEECA**	37.5	111.2 (76.9-153.9)	174.6 (120.4-244.4)	48.0 (33.2-66.0)	75.5 (51.8-105.3)	10.5 (7.4-14.4)	12.6 (8.9-17.1)	2.3 (1.6-3.3)	20.1 (14.0-27.6)
**CEECA**	42.5	111.5 (76.4-154.6)	181.7 (124.4-253.7)	47.3 (33.0-65.2)	71.6 (48.7-100.4)	10.5 (7.5-14.1)	12.5 (8.9-17.0)	2.3 (1.6-3.3)	19.7 (13.7-27.3)
**CEECA**	47.5	110.8 (75.8-153.4)	183.3 (125.6-255.5)	45.9 (31.5-63.2)	67.7 (46.3-94.6)	10.5 (7.5-14.2)	12.4 (8.7-16.8)	2.3 (1.6-3.3)	19.5 (13.6-26.9)
**CEECA**	52.5	108.7 (75.0-151.1)	181.6 (124.5-254.8)	44.5 (30.8-60.9)	64.6 (44.1-90.5)	10.4 (7.4-14)	12.1 (8.5-16.4)	2.3 (1.6-3.2)	19.1 (13.2-26.5)
**CEECA**	57.5	106.2 (72.9-146.5)	177.1 (120.6-248.6)	43.3 (30-59.8)	62.3 (42.3-87.7)	10.4 (7.3-14.1)	11.8 (8.3-16.1)	2.3 (1.6-3.2)	18.9 (13.1-26)
**CEECA**	62.5	103 (70.9-142.5)	169.3 (116.3-235.8)	42.2 (28.9-58.4)	60.5 (40.9-85.3)	10.2 (7.3-13.9)	11.6 (8.3-15.8)	2.3 (1.6-3.2)	18.6 (12.9-25.8)
**CEECA**	67.5	100.7 (69.5-140.6)	162.8 (112.8-228.6)	41.5 (28.8-58.1)	58.6 (39.8-83)	10.1 (7.1-13.8)	11.4 (8.1-15.5)	2.3 (1.6-3.2)	18.5 (12.8-25.7)
**CEECA**	72.5	98.5 (67.9-135.5)	154.9 (104.6-215.9)	40.8 (28.1-56.5)	56.8 (38.4-80.4)	10 .0(7.1-13.6)	11.2 (7.9-15.1)	2.3 (1.6-3.2)	18.2 (12.5-25.2)
**CEECA**	77.5	96.3 (66.6-134.1)	152.1 (104.6-212.3)	39.6 (27.3-54.9)	55.2 (37.7-76.8)	10 .0(7.1-13.6)	10.9 (7.8-14.8)	2.3 (1.6-3.2)	17.9 (12.3-25.1)
**CEECA**	82.5	94.7 (64.1-131.9)	147.1 (98.9-207.0)	38.8 (26.7-54.1)	53.5 (36.2-74.9)	9.9 (7-13.5)	10.7 (7.6-14.6)	2.3 (1.5-3.2)	17.8 (12.2-24.8)
**CEECA**	87.5	93 (63.6-128.6)	142.7 (96.5-200.7)	38.3 (26.3-53.8)	52.2 (35.5-73.8)	9.8 (7-13.3)	10.5 (7.6-14.2)	2.3 (1.5-3.2)	17.6 (12.3-24.2)
**CEECA**	92.5	90.8 (62.8-126.6)	139.7 (95.1-199.2)	37.5 (25.6-52.2)	51 (34.5-72.6)	9.7 (6.9-13.1)	10.4 (7.4-14.1)	2.2 (1.5-3.1)	17.3 (12.0-24.0)
**CEECA**	97.5	88.2 (60.1-123.8)	133.8 (90.3-189.4)	36.8 (25.2-51.5)	49.6 (34.3-69.5)	9.7 (6.7-13.2)	10.3 (7.3-13.9)	2.2 (1.6-3.1)	17.1 (11.9-23.6)
**HIC**	0.5	109.8 (77.6-150.3)	29.1 (20.8-39.5)	3.4 (2.3-5)	3.6 (2.5-5)	14.6 (10.4-19.7)	11.8 (8.3-16)	2.3 (1.6-3.2)	4.8 (3.4-6.6)
**HIC**	1.5	90.0 (63.9-122.8)	35.6 (25.3-48.1)	6.7 (4.6-9.4)	8.0 (5.6-11)	13.4 (9.5-18.0)	11.3 (8.1-15.2)	2.3 (1.6-3.2)	6.9 (4.8-9.4)
**HIC**	3.5	77.1 (53.5-105.9)	41.7 (29.9-55.9)	11.3 (7.7-15.8)	15.1 (10.8-20.4)	12.5 (8.9-16.8)	10.9 (7.8-14.7)	2.3 (1.6-3.2)	9.1 (6.4-12.4)
**HIC**	7.5	67.3 (47.6-92.4)	48.3 (34.5-65.1)	18.1 (12.3-25.3)	26.7 (19-36.3)	11.8 (8.4-16.1)	10.7 (7.6-14.7)	2.3 (1.6-3.2)	11.6 (8.2-15.9)
**HIC**	12.5	65.5 (45.5-89.3)	56.8 (40.6-76.5)	23.4 (15.9-32.7)	37.5 (26.5-50.6)	11.4 (8.1-15.6)	10.6 (7.6-14.2)	2.3 (1.6-3.2)	13.5 (9.5-18.5)
**HIC**	17.5	69.7 (48.3-95.6)	67.6 (48.2-91.3)	25.7 (17.5-36.0)	44.9 (32.1-60.7)	11.3 (8.0-15.3)	10.7 (7.6-14.5)	2.3 (1.6-3.3)	14.8 (10.4-20.0)
**HIC**	22.5	77.2 (54.1-104.7)	81.2 (58.2-108.7)	26.4 (17.8-37.2)	49.3 (34.9-66.9)	11.3 (8.1-15.4)	10.9 (7.8-14.9)	2.3 (1.6-3.2)	15.6 (11.0-21.3)
**HIC**	27.5	86.3 (60.2-118.7)	95.4 (67.8-128.3)	25.8 (17.6-36.1)	51.9 (37.1-70.2)	11.3 (8.0-15.3)	11.2 (8.0-15.1)	2.3 (1.6-3.3)	16.1 (11.4-22)
**HIC**	32.5	94.9 (67.1-130.2)	108.5 (78.2-146.3)	25.2 (17.4-34.8)	53.2 (37.9-71.6)	11.3 (8.0-15.3)	11.4 (8.2-15.5)	2.4 (1.6-3.3)	16.3 (11.6-22.3)
**HIC**	37.5	102.3 (71-140.5)	118.8 (84.4-161.2)	24.6 (16.7-34.5)	53.3 (38.1-71.9)	11.4 (8.1-15.4)	11.5 (8.2-15.7)	2.3 (1.6-3.3)	16.6 (11.8-22.5)
**HIC**	42.5	109 (75.7-148.5)	125.5 (89.8-169.7)	23.9 (16.3-33.6)	52.9 (37.3-72.3)	11.4 (8.1-15.5)	11.4 (8.1-15.5)	2.4 (1.6-3.2)	16.8 (11.9-22.9)

**HIC**	47.5	114.6 (80.5-157)	128.5 (92.3-172.8)	23.2 (15.8-32.2)	51.8 (36.6-70.4)	11.5 (8.3-15.5)	11.5 (8.2-15.6)	2.4 (1.6-3.2)	17 .0(12.0-23.2)
**HIC**	52.5	118.1 (83.2-160.5)	128.2 (92.1-171.4)	22.7 (15.4-32)	50.6 (36.3-68.1)	11.4 (8.2-15.5)	11.4 (8.1-15.5)	2.4 (1.6-3.3)	17.1 (12.0-23.2)
**HIC**	57.5	120.7 (84.2-164.1)	126.7 (91.7-170.8)	22.1 (15.2-30.8)	49 (34.8-66)	11.4 (8-15.6)	11.3 (8-15.5)	2.3 (1.6-3.3)	17.2 (12.2-23.6)
**HIC**	62.5	123.4 (85.6-169.7)	123.5 (88.1-165.8)	21.9 (14.9-30.8)	47.8 (34.2-64.7)	11.5 (8.1-15.5)	11.2 (8-15)	2.3 (1.6-3.2)	17.2 (12.3-23.5)
**HIC**	67.5	124.9 (88.2-171.2)	120.1 (85.4-162)	21.5 (14.5-30.3)	46.5 (33.4-63)	11.4 (8.1-15.5)	11.1 (7.8-15.2)	2.4 (1.6-3.2)	17.4 (12.3-23.7)
**HIC**	72.5	126.9 (90.2-172.7)	117.3 (84.3-157.7)	21.2 (14.4-29.6)	45.4 (32.2-61.6)	11.4 (8.2-15.5)	11.1 (8-14.9)	2.3 (1.6-3.3)	17.4 (12.2-23.8)
**HIC**	77.5	128.1 (91.2-173.2)	114.7 (81.8-154.6)	20.8 (14-29.2)	44.1 (31.2-59.5)	11.4 (8.1-15.3)	11 (7.8-14.8)	2.3 (1.6-3.3)	17.4 (12.4-23.8)
**HIC**	82.5	130.1 (91-177.9)	112.3 (80.5-150.8)	20.7 (14.2-29.1)	43.3 (30.8-58.1)	11.4 (8.2-15.3)	10.9 (7.7-14.8)	2.3 (1.6-3.3)	17.6 (12.5-24.1)
**HIC**	87.5	131.4 (92-180)	110.6 (78.8-149)	20.3 (14-28.4)	42.4 (30.1-56.9)	11.4 (8.1-15.4)	10.9 (7.7-14.7)	2.3 (1.6-3.2)	17.6 (12.6-24.0)
**HIC**	92.5	133.1 (92.8-181.9)	108.5 (77.7-146)	20.1 (13.6-28.1)	41.5 (29.8-56)	11.4 (8.1-15.4)	10.8 (7.6-14.6)	2.4 (1.6-3.3)	17.8 (12.6-24.5)
**HIC**	97.5	134.4 (94.3-184.4)	106.7 (76.7-143.2)	19.9 (13.7-28)	40.9 (29.2-55.6)	11.4 (8-15.4)	10.7 (7.5-14.4)	2.3 (1.6-3.2)	17.8 (12.4-24.3)
**LAC**	0.5	18 (12.8-24.5)	12.3 (8.7-16.9)	4.3 (2.8-6.2)	1.8 (1.2-2.6)	14.3 (10.2-19.2)	12.1 (8.6-16.6)	2.1 (1.4-3)	5.1 (3.5-7.1)
**LAC**	1.5	33.9 (24-46.5)	26.7 (18.7-36.5)	8.5 (5.7-12)	5.9 (3.9-8.4)	11.7 (8.3-16)	10.5 (7.4-14.3)	2.2 (1.5-3.1)	7.1 (4.9-9.9)
**LAC**	3.5	55.1 (39.0-74.6)	48.7 (34.3-66.9)	14.4 (9.7-20.2)	15.5 (10.6-21.8)	10 (7.1-13.6)	9.4 (6.6-12.8)	2.3 (1.6-3.2)	9.3 (6.5-12.8)
**LAC**	7.5	85.5 (60.7-117.5)	83.0 (58.8-113.6)	22.7 (15.2-32.4)	37.2 (25.9-51.8)	8.5 (6.1-11.6)	8.5 (6.0-11.5)	2.3 (1.6-3.2)	11.8 (8.3-16.2)
**LAC**	12.5	104.4 (74.1-142.8)	109.5 (77.4-149.6)	27.7 (18.8-38.7)	55.1 (37.8-76.4)	7.7 (5.4-10.6)	8 (5.7-10.9)	2.3 (1.6-3.3)	13.5 (9.3-18.8)
**LAC**	17.5	108.2 (76.5-147.7)	120.7 (85.6-164.7)	29.3 (19.6-41.7)	59 (40.8-82.6)	7.4 (5.2-10.0)	8 .0(5.6-10.9)	2.4 (1.6-3.3)	14.4 (10.1-19.9)
**LAC**	22.5	103.9 (73.5-141.6)	121.0 (84.8-165.3)	28.7 (19.7-39.9)	54.2 (37.3-75.4)	7.2 (5.1-9.8)	8 .0(5.7-11.0)	2.4 (1.6-3.3)	14.7 (10.2-20.2)
**LAC**	27.5	97 .0(68.7-131.7)	117.6 (82.8-161.1)	27.2 (18.5-37.9)	46.7 (32.3-64.9)	7.2 (5.1-9.9)	8.2 (5.8-11.1)	2.4 (1.6-3.3)	14.9 (10.3-20.8)
**LAC**	32.5	91.4 (64-124.6)	114.2 (80.2-156.9)	25.7 (17.3-36.2)	41.2 (28.1-57.1)	7.2 (5.1-9.8)	8.3 (5.8-11.3)	2.4 (1.6-3.3)	14.8 (10.4-20.2)
**LAC**	37.5	88.2 (62.5-120.5)	111.9 (78.9-154.4)	23.8 (16.2-33.2)	37.8 (26.5-52.4)	7.2 (5.2-9.8)	8.3 (5.9-11.3)	2.4 (1.6-3.3)	14.7 (10.3-19.9)
**LAC**	42.5	86.5 (61.0-117.9)	110.5 (77.2-151)	22.3 (14.8-31.3)	36.2 (24.9-50.4)	7.3 (5.1-9.9)	8.3 (5.9-11.3)	2.4 (1.6-3.3)	14.6 (10.3-20.1)
**LAC**	47.5	87.2 (62.0-119.1)	111 (77.2-152.4)	21 .0(14.1-29.8)	36 .0(24.7-49.8)	7.3 (5.1-10.0)	8.3 (5.8-11.3)	2.4 (1.6-3.3)	14.5 (10.1-19.8)
**LAC**	52.5	89.2 (63.1-122.5)	113 (79.5-154.1)	19.9 (13.3-28)	37.2 (25.6-51.7)	7.3 (5.1-9.8)	8.2 (5.8-11.2)	2.4 (1.6-3.3)	14.2 (9.9-19.5)
**LAC**	57.5	92.6 (65.6-125.6)	116.3 (82.5-159.1)	18.9 (12.8-26.7)	39.5 (27.2-55.1)	7.3 (5.2-10)	8.1 (5.7-11.0)	2.4 (1.6-3.3)	14.2 (9.9-19.5)
**LAC**	62.5	96.1 (67.7-129.6)	119.2 (84.1-163.6)	17.9 (12-25.2)	42.1 (29.1-58)	7.3 (5.2-10)	8.0 (5.5-11.0)	2.3 (1.6-3.2)	14.1 (9.9-19.4)
**LAC**	67.5	100.1 (70.8-136.2)	122 (86-166.2)	17 (11.3-24.3)	44.9 (31.1-62.1)	7.3 (5.2-10)	7.8 (5.5-10.7)	2.3 (1.6-3.2)	14 .0(9.7-19.3)
**LAC**	72.5	103.9 (73.2-141.1)	125 (88-171.2)	16.6 (11.2-23.6)	47.9 (33.5-66.6)	7.4 (5.2-10)	7.7 (5.4-10.6)	2.3 (1.6-3.3)	13.8 (9.7-19.0)
**LAC**	77.5	107.2 (76.3-145.5)	127.7 (90.3-174)	15.9 (10.6-22.4)	50.6 (35.1-70.8)	7.4 (5.2-10.1)	7.6 (5.4-10.5)	2.3 (1.6-3.2)	13.8 (9.5-19.0)
**LAC**	82.5	110.6 (77.4-150.9)	129.9 (91.4-177.2)	15.2 (10.1-21.7)	53.4 (37.2-74.6)	7.4 (5.2-10)	7.5 (5.3-10.3)	2.3 (1.6-3.2)	13.6 (9.5-18.6)

**LAC**	87.5	113.1 (80.3-153.5)	132.5 (94.2-180.4)	14.8 (9.9-21)	55.6 (38.2-77.7)	7.4 (5.2-10)	7.5 (5.3-10.1)	2.3 (1.6-3.2)	13.5 (9.4-18.6)
**LAC**	92.5	116.3 (83.2-155.8)	134.4 (94.4-184.5)	14.2 (9.5-20.2)	58.2 (39.8-80.7)	7.4 (5.2-10.1)	7.4 (5.2-10.1)	2.3 (1.6-3.2)	13.3 (9.3-18.2)
**LAC**	97.5	118.7 (83.3-161.3)	135.7 (94.6-186.9)	13.7 (9.1-19.6)	60 (41.5-83.1)	7.3 (5.2-9.9)	7.3 (5.1-10)	2.3 (1.6-3.2)	13.2 (9.1-18.1)
**MENA**	0.5	9.8 (6.8-13.4)	4.8 (3.4-6.7)	3.1 (2.0-4.7)	0.9 (0.6-1.3)	7.9 (5.5-10.8)	7.5 (5.3-10.2)	2.6 (1.8-3.6)	5.8 (3.7-8.4)
**MENA**	1.5	21.6 (15.2-29.3)	14.2 (9.9-19.7)	6 (3.8-8.8)	3.3 (2.2-4.6)	8.1 (5.8-11)	8 (5.7-10.8)	2.5 (1.7-3.5)	8.7 (5.8-12.3)
**MENA**	3.5	39.5 (27.5-54.2)	33.3 (23.5-44.9)	10 (6.4-14.5)	10 (7-13.8)	8.4 (6-11.3)	8.5 (6-11.5)	2.4 (1.7-3.4)	12 .0(8.2-16.9)
**MENA**	7.5	68 (48.2-93.1)	71.6 (50.6-97.3)	15.7 (10.3-22.7)	27.4 (19.1-37.6)	8.6 (6-11.8)	8.9 (6.3-12.2)	2.4 (1.6-3.3)	16.5 (11.3-23)
**MENA**	12.5	90.8 (64.3-123.9)	110 (78.1-148.6)	19.7 (12.9-28.6)	44.1 (31-60.6)	8.8 (6.3-11.9)	9.3 (6.6-12.8)	2.3 (1.6-3.2)	19.7 (13.5-27.5)
**MENA**	17.5	101.2 (71.3-138.3)	132.8 (93.7-180.3)	21.2 (13.9-30.9)	47.7 (33.2-66)	8.9 (6.3-12.1)	9.8 (6.9-13.3)	2.4 (1.6-3.3)	22.6 (15.6-31.5)
**MENA**	22.5	103.2 (73.1-141.6)	142.4 (101.5-191.2)	21.1 (13.8-30.9)	42.9 (30.3-58.7)	9.1 (6.4-12.4)	10.4 (7.3-14.1)	2.4 (1.6-3.3)	23.8 (16.5-33.3)
**MENA**	27.5	101.7 (72.6-138)	143.9 (101.7-193.1)	20.5 (13.4-29.7)	36.3 (25.6-50.1)	9.2 (6.6-12.4)	10.7 (7.7-14.7)	2.4 (1.7-3.3)	24.4 (16.9-33.6)
**MENA**	32.5	100.2 (71.2-135.5)	145.4 (102.8-197.8)	19.4 (12.8-28)	31.3 (21.9-43.1)	9.3 (6.6-12.7)	11 (7.7-15.1)	2.4 (1.6-3.3)	24.7 (17.1-34.5)
**MENA**	37.5	99.8 (70.1-136.9)	146.1 (102.1-200.5)	18.1 (11.7-26.3)	27.9 (19.4-38.8)	9.4 (6.6-12.7)	11.2 (7.9-15.2)	2.4 (1.7-3.3)	24.8 (17-34.5)
**MENA**	42.5	100.1 (70-136.4)	147.8 (103.4-202.6)	16.8 (10.9-24.5)	25.9 (18-35.4)	9.3 (6.7-12.6)	11.1 (7.8-15.2)	2.4 (1.7-3.4)	24.7 (17-34.4)
**MENA**	47.5	101.7 (71.9-137.3)	149.2 (105.7-202.4)	15.4 (9.9-22.5)	25.3 (17.7-34.8)	9.2 (6.6-12.4)	10.9 (7.6-14.9)	2.4 (1.6-3.4)	24.7 (17-34.1)
**MENA**	52.5	103.8 (72.9-142.8)	152.5 (109.1-205.2)	14.3 (9.4-20.5)	25.6 (17.7-35)	9.1 (6.4-12.2)	10.5 (7.4-14.3)	2.4 (1.7-3.3)	24.5 (16.9-34.1)
**MENA**	57.5	107 (74.9-145.9)	156.2 (111.8-211.4)	13.3 (8.7-19.5)	26.8 (18.6-37.1)	8.9 (6.3-12.1)	10.2 (7.2-14)	2.4 (1.7-3.3)	24.4 (16.8-34)
**MENA**	62.5	110.7 (77.8-152)	160.7 (114.3-217.6)	12.3 (8-17.9)	27.9 (19.8-38.2)	8.8 (6.3-11.9)	9.8 (6.9-13.3)	2.4 (1.6-3.3)	24.2 (16.6-33.6)
**MENA**	67.5	114.4 (79.9-155.4)	165.2 (117.2-222.3)	11.4 (7.4-16.5)	29.1 (20.5-39.8)	8.6 (6.1-11.7)	9.4 (6.6-12.9)	2.4 (1.7-3.3)	24 (16.7-33)
**MENA**	72.5	118 (82.8-160.4)	169.8 (121.6-227.9)	10.7 (7-15.4)	30.8 (21.5-42.5)	8.5 (6.1-11.6)	9.2 (6.5-12.5)	2.4 (1.6-3.3)	24.1 (16.6-33.6)
**MENA**	77.5	121.4 (85.7-165.5)	173 (122.7-235.1)	10 (6.5-14.4)	32 (22.6-44.1)	8.4 (6-11.4)	8.8 (6.2-12)	2.4 (1.6-3.3)	23.9 (16.3-33.1)
**MENA**	82.5	125 (87.8-171.2)	176.5 (125.2-239.9)	9.5 (6.2-13.7)	33.3 (23.5-45.2)	8.3 (5.9-11.4)	8.5 (5.9-11.6)	2.3 (1.6-3.3)	23.8 (16.4-32.9)
**MENA**	87.5	127.5 (90.1-172.7)	180.2 (128.4-244.4)	8.9 (5.8-12.9)	34.5 (24-47.4)	8.2 (5.9-11.2)	8.2 (5.8-11.3)	2.3 (1.6-3.3)	24.1 (16.4-33.5)
**MENA**	92.5	130 (92.4-176.8)	184.1 (129.2-248.6)	8.1 (5.3-11.7)	35.2 (25.1-48)	8.1 (5.8-11)	8.1 (5.7-11.2)	2.3 (1.6-3.2)	24.4 (16.5-34.1)
**MENA**	97.5	132 (92.3-180.6)	187.9 (133.2-256.8)	7.5 (4.9-11)	36.3 (25.6-49.6)	8 (5.6-10.8)	7.9 (5.5-10.8)	2.3 (1.6-3.2)	24.9 (16.8-34.7)
**SAARC**	0.5	13.7 (9.3-19.3)	5.7 (3.9-7.8)	1 (0.6-1.5)	0.7 (0.5-1.1)	10.6 (7.4-14.3)	8 (5.6-11)	2.2 (1.5-3)	10.9 (7.4-15.3)
**SAARC**	1.5	27.6 (18.8-38.6)	18 (12.7-24.6)	1.6 (1-2.4)	2.3 (1.5-3.3)	8.4 (5.9-11.5)	6.8 (4.7-9.4)	2.2 (1.5-3.1)	15.5 (10.7-21.4)
**SAARC**	3.5	47.4 (32.3-66.6)	44.1 (31.3-59.6)	2.4 (1.4-3.6)	6.2 (4.2-8.9)	6.9 (4.8-9.5)	6 (4.1-8.4)	2.2 (1.5-3.1)	19.8 (13.7-27.8)
**SAARC**	7.5	76.4 (52.2-107.3)	98.4 (69.6-134.4)	3.4 (2.1-5.1)	15.3 (10.3-21.6)	5.7 (4-7.9)	5.3 (3.7-7.4)	2.2 (1.5-3.1)	24.3 (16.9-33.2)
**SAARC**	12.5	97.4 (65.8-138.2)	151.3 (108.2-206)	4 (2.5-6.1)	22.7 (15.4-31.7)	5.2 (3.5-7.1)	5 (3.5-6.9)	2.2 (1.5-3.1)	26.9 (18.8-37.2)

**SAARC**	17.5	101.6 (69-142.3)	178.2 (126.1-244.5)	4.1 (2.5-6.2)	23.4 (15.7-32.9)	4.9 (3.4-6.8)	4.9 (3.4-6.7)	2.2 (1.5-3.1)	31.4 (21.4-44.1)
**SAARC**	22.5	96.6 (65.7-134.9)	185.8 (129.6-254)	3.8 (2.3-5.8)	20.2 (13.7-28.2)	4.9 (3.3-6.6)	4.8 (3.4-6.7)	2.2 (1.5-3.1)	31.9 (22.1-44.4)
**SAARC**	27.5	89 (60.6-124.2)	182.8 (127.5-249.2)	3.5 (2.1-5.2)	16.9 (11.4-23.9)	4.9 (3.4-6.7)	4.9 (3.4-6.8)	2.2 (1.5-3.1)	32.3 (22.4-44.7)
**SAARC**	32.5	84 (56.9-119)	176.4 (124.8-239.1)	3.2 (1.9-4.8)	14.8 (10.1-20.7)	4.9 (3.4-6.8)	4.8 (3.3-6.7)	2.2 (1.5-3.2)	32.7 (22.4-45.6)
**SAARC**	37.5	80.6 (55.2-112.9)	170.3 (121.1-232.3)	3 (1.9-4.7)	13.9 (9.4-19.7)	4.9 (3.4-6.8)	4.9 (3.4-6.7)	2.2 (1.5-3.1)	32.8 (22.9-45.9)
**SAARC**	42.5	78.2 (53.9-110.3)	166.7 (119-226.2)	3 (1.8-4.5)	14.1 (9.6-19.8)	5.0 (3.5-6.9)	5 .0(3.5-6.9)	2.2 (1.5-3.1)	32.9 (23-45.2)
**SAARC**	47.5	77.8 (53.2-109)	162.5 (114.1-220.2)	2.9 (1.8-4.5)	15.3 (10.4-21.6)	5.1 (3.5-7)	5.0 (3.5-6.9)	2.2 (1.5-3.2)	32.8 (22.6-45.6)
**SAARC**	52.5	78.6 (53.9-109.4)	160.3 (113.4-219.2)	2.9 (1.8-4.6)	17.7 (12-24.7)	5.1 (3.6-7.1)	5.0 (3.5-6.9)	2.2 (1.5-3.1)	33.1 (22.8-46.2)
**SAARC**	57.5	80.4 (54.9-112.5)	158.3 (113.5-213.2)	3 (1.8-4.5)	21.1 (14.4-29.7)	5.2 (3.6-7.2)	5.1 (3.5-7.1)	2.2 (1.5-3.1)	33.1 (23.1-45.7)
**SAARC**	62.5	83.6 (56.8-117.6)	156.6 (109.8-211.2)	3.1 (1.9-4.6)	25.6 (17.5-35.6)	5.2 (3.6-7.3)	5.1 (3.6-7.1)	2.2 (1.5-3.2)	33.2 (23-46.1)
**SAARC**	67.5	85.7 (58.1-122)	156.6 (110.5-214.5)	3.2 (1.9-4.8)	30.6 (20.9-42.7)	5.3 (3.7-7.3)	5.1 (3.6-7)	2.3 (1.5-3.1)	33.4 (22.9-46.1)
**SAARC**	72.5	87.3 (59.1-121.7)	156.2 (110.8-213.5)	3.2 (1.9-4.9)	36.3 (24.8-50.7)	5.3 (3.7-7.4)	5.2 (3.6-7.2)	2.3 (1.5-3.1)	33.3 (23-46.4)
**SAARC**	77.5	88.8 (60.7-124.7)	155.6 (110.3-212.7)	3.3 (2.1-5.1)	42.3 (28.8-59.8)	5.4 (3.7-7.4)	5.2 (3.6-7.2)	2.3 (1.6-3.1)	33.1 (22.8-45.7)
**SAARC**	82.5	89.8 (61.3-125.4)	153.5 (108.2-208.9)	3.3 (2-5.1)	48.7 (33.3-67.3)	5.5 (3.8-7.5)	5.2 (3.6-7.2)	2.3 (1.6-3.1)	33.1 (23-45.8)
**SAARC**	87.5	90.9 (62.2-127.5)	151.5 (106.6-207.4)	3.4 (2-5.2)	55 (37.7-76.4)	5.5 (3.8-7.6)	5.2 (3.6-7.1)	2.3 (1.5-3.2)	33.3 (23.2-46.1)
**SAARC**	92.5	91.1 (61.8-128.6)	150.2 (105.9-205.4)	3.3 (2-5.1)	62.1 (42.8-86.3)	5.5 (3.8-7.6)	5.2 (3.6-7.2)	2.3 (1.6-3.2)	33.5 (23.3-46.3)
**SAARC**	97.5	89.6 (61.3-126.3)	148.3 (104.6-202.2)	3.3 (2-5)	69.3 (47.5-96.6)	5.5 (3.8-7.6)	5.1 (3.5-7.0)	2.3 (1.6-3.2)	33 (22.9-45.3)
**SSA**	0.5	8.1 (5.6-11.3)	9.1 (6.3-12.5)	2.1 (1.3-3.1)	1.1 (0.7-1.6)	9.8 (7-13.3)	9.5 (6.8-12.9)	2.3 (1.6-3.3)	7.6 (5.2-10.5)
**SSA**	1.5	18 (12.3-24.8)	22.4 (15.5-30.9)	3.8 (2.4-5.6)	3 .0(2.0-4.3)	9 (6.3-12.3)	9.2 (6.4-12.4)	2.3 (1.6-3.2)	11.0 (7.6-15.4)
**SSA**	3.5	33.4 (23.1-46.1)	45.2 (31.2-62.5)	6.1 (3.9-9)	7.1 (4.8-10)	8.4 (5.9-11.5)	8.9 (6.4-12)	2.3 (1.6-3.3)	14.8 (10.2-20.4)
**SSA**	7.5	58.1 (40.4-80.5)	83.9 (57.7-114.9)	9.2 (5.9-13.4)	15.1 (10.5-20.9)	7.9 (5.6-10.7)	8.7 (6.1-11.9)	2.4 (1.6-3.3)	18.7 (13.1-25.7)
**SSA**	12.5	77.1 (53.1-107.7)	116.3 (80.7-160.2)	11.1 (7.1-16.3)	22 (15.1-30.5)	7.7 (5.3-10.5)	8.7 (6.1-12)	2.4 (1.6-3.3)	21.4 (15-29.3)
**SSA**	17.5	83.6 (58.2-114.5)	131.2 (90.5-179.7)	11.3 (7.2-16.6)	24.3 (16.7-33.6)	7.6 (5.4-10.4)	8.9 (6.3-12.1)	2.4 (1.6-3.3)	24.3 (16.9-33.5)
**SSA**	22.5	82.8 (57.8-112.6)	132.1 (91.8-181.6)	10.7 (6.8-15.7)	23 (15.9-31.6)	7.7 (5.5-10.5)	9.1 (6.5-12.4)	2.4 (1.6-3.3)	25.2 (17.5-35)
**SSA**	27.5	79.3 (55.9-108.9)	128.1 (88.7-176.9)	9.8 (6.2-14.5)	20.7 (14.3-28.7)	7.7 (5.5-10.5)	9.3 (6.7-12.7)	2.4 (1.6-3.3)	25.8 (18-35)
**SSA**	32.5	75.5 (52.7-103.4)	122.7 (85.2-169.8)	8.9 (5.7-13.1)	18.7 (12.9-25.7)	7.9 (5.6-10.7)	9.5 (6.7-13)	2.3 (1.6-3.2)	26.1 (18.1-35.9)
**SSA**	37.5	72.3 (50.5-99.8)	119.5 (82.8-166)	8.1 (5.2-12)	17.3 (11.8-23.8)	8 (5.6-10.8)	9.7 (6.9-13.2)	2.3 (1.6-3.2)	26 (18.2-35.6)
**SSA**	42.5	70.6 (49.1-97)	117.3 (82-161)	7.4 (4.8-10.8)	16.1 (11.1-22.5)	8.1 (5.8-11)	9.8 (7-13.3)	2.3 (1.6-3.2)	26.1 (18.3-36.4)
**SSA**	47.5	69.2 (47.8-95.5)	115.9 (79.4-162.2)	6.9 (4.4-10.2)	15.5 (10.6-21.6)	8.1 (5.8-11)	9.9 (7-13.4)	2.3 (1.6-3.2)	26 (18.1-35.9)
**SSA**	52.5	69.5 (48.3-95.4)	116.6 (81.5-161.3)	6.4 (4.1-9.4)	15.2 (10.5-21.3)	8.3 (5.9-11.3)	9.9 (7-13.4)	2.3 (1.6-3.2)	25.7 (17.8-35.5)

**SSA**	57.5	69.5 (49-96.4)	117.5 (82.3-161.5)	6 (3.8-8.9)	15.1 (10.4-21.2)	8.3 (5.9-11.2)	10 .0(7.1-13.6)	2.3 (1.6-3.2)	25.5 (17.7-35.2)
**SSA**	62.5	70.2 (49.4-97.4)	119.1 (82.4-164.4)	5.7 (3.7-8.4)	15 (10.2-21)	8.4 (5.8-11.5)	10.0 (7.1-13.6)	2.3 (1.6-3.2)	25.4 (17.8-35.1)
**SSA**	67.5	70.7 (49-97.1)	120.6 (82.8-166.9)	5.4 (3.4-7.9)	15.3 (10.6-21.4)	8.4 (6-11.3)	10.0 (7.1-13.4)	2.3 (1.6-3.2)	25.1 (17.6-34.6)
**SSA**	72.5	71.4 (49.2-98.1)	122.4 (85.3-168.9)	5.2 (3.3-7.6)	15.3 (10.5-21.4)	8.5 (6-11.5)	10.0 (7.1-13.7)	2.3 (1.6-3.2)	24.9 (17.4-34.5)
**SSA**	77.5	71.4 (50-98)	123.7 (86.7-170.3)	4.9 (3.1-7.2)	15.3 (10.5-21.4)	8.5 (6-11.7)	9.9 (7.0-13.5)	2.3 (1.6-3.2)	24.5 (17-33.8)
**SSA**	82.5	71.7 (49.8-98.7)	124.3 (86.1-170.3)	4.7 (3-7)	15.3 (10.5-21.3)	8.6 (6-11.7)	10 .0(7-13.7)	2.3 (1.6-3.2)	24.4 (16.9-33.4)
**SSA**	87.5	72.3 (50.7-99.9)	125.4 (87.9-171.7)	4.6 (2.8-6.8)	15.3 (10.5-21.4)	8.6 (6.1-11.6)	10 .0(7-13.8)	2.3 (1.6-3.2)	24.1 (16.7-33.1)
**SSA**	92.5	72 (49.3-99.8)	126 (86.6-176.3)	4.4 (2.8-6.4)	15.2 (10.4-21.2)	8.6 (6.1-11.7)	10 .0(7-13.9)	2.3 (1.6-3.2)	23.8 (16.7-32.8)
**SSA**	97.5	71.8 (50.6-99.1)	124.5 (84.7-170.9)	4.2 (2.7-6.3)	15 (10.3-21)	8.6 (6-11.7)	10 .0(7-13.5)	2.3 (1.6-3.1)	23.8 (16.5-33.0)
**world**	0.5	26.1 (16.7-38.6)	14.9 (9.9-21.2)	4.0 (2.5-5.9)	2.1 (1.3-3.1)	11 (7.7-15.1)	9.8 (7-13.3)	2.2 (1.5-3.1)	5.5 (3.7-7.8)
**world**	1.5	36.7 (24.6-51.6)	27.9 (19.2-38.6)	7.5 (4.7-11.1)	5.9 (3.8-8.6)	10.3 (7.4-14)	9.5 (6.7-12.8)	2.3 (1.6-3.2)	8.0 (5.5-11.3)
**world**	3.5	51.2 (35.3-71.5)	46.9 (32.3-64.5)	12.3 (7.9-18.1)	13.8 (9.2-19.7)	9.8 (6.9-13.3)	9.3 (6.5-12.7)	2.3 (1.6-3.2)	10.7 (7.4-15.0)
**world**	7.5	71.1 (49.8-97.3)	76.4 (52.4-105.3)	18.7 (12.2-27.2)	30.1 (20.2-42.9)	9.3 (6.5-12.8)	9.1 (6.4-12.4)	2.3 (1.6-3.2)	13.9 (9.5-19.3)
**world**	12.5	85.3 (59.6-117.4)	102.1 (70-141.3)	22.6 (14.4-32.9)	43.9 (29.7-62.5)	9.1 (6.4-12.6)	9.1 (6.4-12.4)	2.3 (1.6-3.2)	16 .0(10.8-22.3)
**world**	17.5	90.7 (62.3-125)	117.8 (82.1-162.2)	23.4 (15.2-33.9)	47.9 (32.2-67.9)	9 .0(6.4-12.3)	9.2 (6.4-12.5)	2.4 (1.6-3.3)	17.8 (12.2-24.8)
**world**	22.5	91.2 (63.4-125.8)	126.6 (87.9-173.8)	22.4 (14.6-32.5)	45.4 (30.1-64.3)	9.0 (6.3-12.3)	9.4 (6.6-12.9)	2.4 (1.6-3.3)	18.6 (12.7-25.6)
**world**	27.5	89.7 (63.3-122.9)	130.8 (91.9-179.7)	20.7 (13.2-30.6)	41.6 (27.5-59.9)	9 .0(6.3-12.2)	9.5 (6.7-13)	2.4 (1.6-3.3)	19 .0(13.0-26.5)
**world**	32.5	88 (61.1-120.7)	133.8 (93.7-185.6)	19.5 (12.5-28.7)	37.9 (25.3-54.4)	9 .0(6.4-12.4)	9.6 (6.8-13.3)	2.4 (1.6-3.3)	19.3 (13.2-27.0)
**world**	37.5	87.5 (60.9-120.3)	134.0 (93.1-184.1)	18.1 (11.5-27.0)	35.4 (23.5-50.6)	9.0 (6.4-12.4)	9.7 (6.8-13.3)	2.3 (1.6-3.3)	19.3 (13.3-26.9)
**world**	42.5	86.8 (60.3-118.5)	135.7 (94.6-187.6)	16.8 (10.8-25.0)	33.8 (22.4-47.8)	9 .0(6.3-12.2)	9.7 (6.8-13.2)	2.3 (1.6-3.2)	19.3 (13.3-26.7)
**world**	47.5	87.4 (61-120.6)	135.7 (94.7-186.9)	15.8 (9.9-23.7)	33.2 (22.1-47)	9.0 (6.3-12.3)	9.6 (6.7-13.1)	2.3 (1.6-3.2)	19.3 (13.2-27.1)
**world**	52.5	88.1 (60.4-120.7)	135.8 (95.1-185.3)	15 .0(9.4-22.5)	33.4 (22.3-47.7)	8.9 (6.3-12.3)	9.5 (6.7-12.9)	2.3 (1.6-3.2)	19.3 (13.2-26.6)
**world**	57.5	90.1 (62.6-125.4)	136.1 (95.2-187.2)	14.2 (8.8-21.5)	34.3 (23.0-48.6)	8.9 (6.2-12.1)	9.3 (6.6-12.5)	2.3 (1.6-3.3)	19.1 (13.1-26.6)
**world**	62.5	91.8 (63.4-125.9)	136.8 (94.2-190.2)	13.7 (8.4-20.7)	35.4 (23.7-50.3)	8.8 (6.1-11.9)	9.1 (6.4-12.4)	2.3 (1.6-3.2)	19.1 (13.2-26.7)
**world**	67.5	93.1 (64.4-129.1)	137.0 (94.7-189.7)	13.0 (8.1-19.6)	37.0 (25.1-51.9)	8.7 (6.2-11.9)	9.0 (6.3-12.3)	2.3 (1.6-3.2)	19.0 (13.0-26.5)
**world**	72.5	95.3 (66.1-131.3)	137.2 (96.2-186.8)	12.7 (7.9-19.0)	38.9 (26.4-54.8)	8.7 (6.1-12.0)	8.8 (6.1-12.2)	2.3 (1.6-3.2)	18.9 (13.1-26.2)
**world**	77.5	96.9 (67.3-134.2)	138.0 (97.4-188.2)	12.0 (7.5-18.0)	40.4 (27.3-56.5)	8.7 (6.1-11.8)	8.7 (6.1-11.8)	2.3 (1.6-3.2)	18.8 (13.0-26.0)
**world**	82.5	98.0 (67.9-136.0)	137.7 (95-191.3)	11.6 (7.3-17.6)	42.3 (28.6-59.3)	8.6 (.6.0-11.7)	8.6 (6.0-11.8)	2.3 (1.6-3.2)	18.8 (12.8-26.4)
**world**	87.5	99.1 (68.4-136.7)	138.5 (95.6-191)	11.1 (6.9-16.9)	44 (29.7-61.5)	8.6 (6.0-11.7)	8.4 (5.8-11.6)	2.3 (1.6-3.2)	18.8 (12.9-26.1)
**world**	92.5	100.1 (69.6-139.2)	139 .0(96.2-193.2)	10.6 (6.6-16.2)	46.3 (31.1-65.4)	8.5 (6.0-11.6)	8.3 (5.7-11.4)	2.3 (1.6-3.2)	18.8 (13.1-26.0)

**world**	97.5	100.0 (68.2-138.4)	139 .0(96-192.1)	10.4 (6.3-15.5)	48.3 (32.2-69.2)	8.4 (5.9-11.6)	8.2 (5.8-11.4)	2.3 (1.6-3.2)	18.7 (12.9-25.9)

In previous global dietary database reports, Central Europe, Eastern Europe, and Central Asia were referred to as the former Soviet Union, while Southeast Asia and East Asia were collectively called Asia. UI, Uncertainty Interval. Midpoint ages correspond to specific age groups. Ages 0.5, 1.5, and 3.5 represent 0–1 year, 1–2 years, and 3–4 years, respectively. From 7.5 onwards, midpoint ages represent standard 5-year intervals (e.g. 5–9 years, 10–14 years, etc.).

Supplementary Table 11: Search strategies including the key terms and the queries for each database.

Nutritional factors	PubMed	Web of Science	Cochrane	Embase
Fruits	#1 Fruits [All Fields] OR Fruit [All Fields] OR Berries [All Fields] OR Berry [All Fields] OR Plant Capsule [All Fields] OR Capsule, Plant [All Fields] OR Capsules, Plant [All Fields] OR Plant Capsules [All Fields] #2 Inflammatory Bowel Disease [All Fields] OR Bowel Diseases, Inflammatory [All Fields] OR IBD [All Fields] OR ulcer colitis [All Fields] OR colitis ulcer [All Fields] OR morbus crohn [All Fields] OR crohn's disease [All Fields] OR crohn disease [All Fields] #3 #1 AND	#1 TS=Fruits OR Fruit OR Berries OR Berry OR Plant Capsule OR Capsule, Plant OR Capsules, Plant OR Plant Capsules #2 TS=Inflammatory Bowel Disease OR IBD OR ulcer colitis OR colitis ulcer OR morbus crohn OR crohn's disease OR crohn disease #3 #1 AND #2<br><br>N=1234	#1 MeSH descriptor: [Fruits] explode all trees #2 (Fruits):ti,ab,kw OR (Fruit):ti,ab,kw OR (Berries):ti,ab,kw OR (Berry):ti,ab,kw OR (Plant Capsule):ti,ab,kw OR (Capsule,Plant):ti,ab,kw OR (Capsules,Plant):ti,ab,kw OR (Plant Capsules):ti,ab,kw #3 #1 OR #2 #4 MeSH descriptor: [Inflammatory Bowel Disease] explode all trees #5 (Inflammatory Bowel Disease):ti,ab,kw OR (Bowel Diseases, Inflammatory):ti,ab,kw OR (IBD):ti,ab,kw OR (ulcer colitis):ti,ab,kw OR (colitis ulcer):ti,ab,kw OR (morbus	#1 'fruit'/exp OR 'fruit' #2 'Fruits':ti,ab,kw OR 'Fruit':ti,ab,kw OR 'Berries':ti,ab,kw OR 'Berry':ti,ab,kw OR 'Plant Capsule':ti,ab,kw OR 'Capsule,Plant':ti,ab,kw OR 'Capsules,Plant':ti,ab,kw OR 'Plant Capsules':ti,ab,kw #3 #1 OR #2 #4 'Inflammatory Bowel Disease'/exp OR 'Inflammatory Bowel Disease' #5 'Inflammatory Bowel Disease':ti,ab,kw OR 'Bowel Diseases, Inflammatory':ti,ab,kw OR 'IBD':ti,ab,kw OR 'ulcer colitis':ti,ab,kw OR 'colitis ulcer':ti,ab,kw OR 'morbus crohn':ti,ab,kw OR 'crohn disease':ti,ab,kw

	#2<br><br>N=768		crohn):ti,ab,kw OR (crohn's disease):ti,ab,kw OR (crohn disease):ti,ab,kw<br>#6 #4 OR #5<br>#7 #3 AND #6<br><br>N=61	#6 #4 OR #5<br>#7 #3 AND #6<br><br>N=1424
Vegetables	#1 vegetable [All Fields] OR vegetables [All Fields] OR Non-starchy vegetables [All Fields]<br>#2 Inflammatory Bowel Disease [All Fields] OR Bowel Diseases, Inflammatory [All Fields] OR IBD [All Fields] OR ulcer colitis [All Fields] OR colitis ulcer [All Fields] OR morbus crohn [All Fields] OR crohn's disease [All Fields] OR crohn disease	#1 TS=vegetable OR vegetables OR Non-starchy vegetables<br>#2 TS=Inflammatory Bowel Disease OR IBD OR ulcer colitis OR colitis ulcer OR morbus crohn OR crohn's disease OR crohn disease<br>#3 #1 AND #2<br><br>N=908	#1 MeSH descriptor: [vegetable] explode all trees<br>#2 (vegetable):ti,ab,kw OR (vegetables):ti,ab,kw OR (Nonstarchy vegetables):ti,ab,kw<br>#3 #1 OR #2<br>#4 MeSH descriptor: [Inflammatory Bowel Disease] explode all trees<br>#5 (Inflammatory Bowel Disease):ti,ab,kw OR (Bowel Diseases, Inflammatory):ti,ab,kw OR (IBD):ti,ab,kw OR (ulcer	#1 'vegetable'/exp OR 'vegetable'<br>#2 'vegetable’:ti,ab,kw OR 'vegetables':ti,ab,kw OR 'Nonstarchy vegetables':ti,ab,kw<br>#3 #1 OR #2<br>#4 'Inflammatory Bowel Disease'/exp OR 'Inflammatory Bowel Disease'<br>#5 'Inflammatory Bowel Disease':ti,ab,kw OR 'Bowel Diseases, Inflammatory':ti,ab,kw OR 'IBD':ti,ab,kw OR 'ulcer colitis':ti,ab,kw OR 'colitis ulcer':ti,ab,kw OR 'morbus

	[All Fields]<br>#3 #1 AND #2<br><br>N=370		colitis):ti,ab,kw OR (colitis ulcer):ti,ab,kw OR (morbus crohn):ti,ab,kw OR (crohn's disease):ti,ab,kw OR (crohn disease):ti,ab,kw<br>#6 #4 OR #5<br>#7 #3 AND #6<br><br>N=61	crohn':ti,ab,kw OR 'crohn disease':ti,ab,kw<br>#6 #4 OR #5<br>#7 #3 AND #6<br><br>N=1230
Dietary fiber	#1 Dietary fiber [All Fields] OR Dietary Fibers [All Fields] OR Fibers, Dietary [All Fields] OR Fiber, Dietary [All Fields] OR Wheat Bran [All Fields] OR Bran, Wheat [All Fields] OR Wheat Brans [All Fields] OR Roughage [All Fields] OR Roughages [All Fields]<br>#2 Inflammatory Bowel Disease [All Fields] OR Bowel Diseases, Inflammatory [All Fields] OR	#1 TS=Dietary fiber OR Dietary Fibers OR Fibers, Dietary OR Fiber, Dietary OR Wheat Bran OR Bran, Wheat OR Wheat Brans OR Roughage OR Roughages<br>#2 TS=Inflammatory Bowel Disease OR IBD OR ulcer colitis OR colitis ulcer OR morbus crohn OR	#1 MeSH descriptor: [Dietary fiber] explode all trees<br>#2 (Dietary fiber):ti,ab,kw OR (Dietary Fibers):ti,ab,kw OR (Fibers, Dietary):ti,ab,kw OR (Fiber, Dietary):ti,ab,kw OR (Wheat Bran):ti,ab,kw OR (Bran, Wheat):ti,ab,kw OR (Wheat Brans):ti,ab,kw OR (Roughage):ti,ab,kw OR (Roughages):ti,ab,kw<br>#3 #1 OR #2<br>#4 MeSH descriptor: [Inflammatory Bowel Disease]	#1 'Dietary fiber'/exp OR 'Dietary fiber'<br>#2 'Dietary Fibers’:ti,ab,kw OR 'Fibers, Dietary':ti,ab,kw OR 'Fiber, Dietary':ti,ab,kw OR 'Wheat Bran':ti,ab,kw OR 'Bran, Wheat':ti,ab,kw OR 'Wheat Brans':ti,ab,kw OR 'Roughage':ti,ab,kw OR 'Roughages':ti,ab,kw<br>#3 #1 OR #2<br>#4 'Inflammatory Bowel Disease'/exp OR 'Inflammatory Bowel Disease'<br>#5 'Inflammatory Bowel Disease':ti,ab,kw

	IBD [All Fields] OR ulcer colitis [All Fields] OR colitis ulcer [All Fields] OR morbus crohn [All Fields] OR crohn's disease [All Fields] OR crohn disease [All Fields]<br>#3 #1 AND #2<br><br>N=920	crohn's disease OR crohn disease<br>#3 #1 AND #2<br><br>N=1640	explode all trees<br>#5 (Inflammatory Bowel Disease):ti,ab,kw OR (Bowel Diseases, Inflammatory):ti,ab,kw OR (IBD):ti,ab,kw OR (ulcer colitis):ti,ab,kw OR (colitis ulcer):ti,ab,kw OR (morbus crohn):ti,ab,kw OR (crohn's disease):ti,ab,kw OR (crohn disease):ti,ab,kw<br>#6 #4 OR #5<br>#7 #3 AND #6<br><br>N=138	OR 'Bowel Diseases, Inflammatory':ti,ab,kw OR 'IBD':ti,ab,kw OR 'ulcer colitis':ti,ab,kw OR 'colitis ulcer':ti,ab,kw OR 'morbus crohn':ti,ab,kw OR 'crohn disease':ti,ab,kw<br>#6 #4 OR #5<br>#7 #3 AND #6<br><br>N=2419
Saturated Fatty Acid	#1 Saturated fatty acid [All Fields] OR Fatty Acid [All Fields] OR Aliphatic Acids [All Fields] OR Aliphatic Acid [All Fields] OR Acid, Aliphatic [All Fields] OR Fatty Acids, Esterified[All Fields] OR Esterified Fatty Acids [All Fields] OR Esterified Fatty Acid [All Fields] OR	#1 TS=Saturated fatty acid OR Fatty Acid OR Aliphatic Acids OR Aliphatic Acid OR Acid, Aliphatic OR Fatty Acids, Esterified[All Fields] OR Esterified Fatty Acids OR Esterified Fatty Acid OR Acid,	#1 MeSH descriptor: [Saturated fatty acid] explode all trees<br>#2 (Saturated fatty acid):ti,ab,kw OR (Fatty Acid):ti,ab,kw OR (Aliphatic Acids):ti,ab,kw OR (Aliphatic Acid):ti,ab,kw OR (Acid, Aliphatic):ti,ab,kw OR (Fatty Acids, Esterified):ti,ab,kw OR (Esterified Fatty Acid):ti,ab,kw OR	#1 'Saturated fatty acid'/exp OR 'Saturated fatty acid'<br>#2 'Saturated fatty acid':ti,ab,kw OR 'Fatty Acid':ti,ab,kw OR 'Aliphatic Acids':ti,ab,kw OR 'Aliphatic Acid':ti,ab,kw OR 'Acid, Aliphatic':ti,ab,kw OR 'Fatty Acids, Esterified':ti,ab,kw OR 'Esterified Fatty Acids':ti,ab,kw OR 'Esterified Fatty

	Acid, Esterified Fatty [All Fields] OR Fatty Acid, Esterified [All Fields] OR Fatty Acids, Saturated [All Fields] OR Saturated Fatty Acids [All Fields] OR Saturated Fatty Acid [All Fields] OR Acid, Saturated Fatty [All Fields] OR Fatty Acid, Saturated [All Fields]<br>#2 Inflammatory Bowel Disease [All Fields] OR Bowel Diseases, Inflammatory [All Fields] OR IBD [All Fields] OR ulcer colitis [All Fields] OR colitis ulcer [All Fields] OR morbus crohn [All Fields] OR crohn's disease [All Fields] OR crohn disease [All Fields]<br>#3 #1 AND #2	Esterified Fatty OR Fatty Acid, Esterified OR Fatty Acids, Saturated OR Saturated Fatty Acids OR Saturated Fatty Acid OR Acid, Saturated Fatty OR Fatty Acid, Saturated<br>#2 TS=Inflammatory Bowel Disease OR IBD OR ulcer colitis OR colitis ulcer OR morbus crohn OR crohn's disease OR crohn disease<br>#3 #1 AND #2<br><br>N=6125	(Esterified Fatty Acids):ti,ab,kw OR (Acid, Esterified Fatty):ti,ab,kw OR (Fatty Acid, Esterified):ti,ab,kw OR (Fatty Acids, Saturated):ti,ab,kw OR (Saturated Fatty Acids):ti,ab,kw OR (Saturated Fatty Acid):ti,ab,kw OR (Acid, Saturated Fatty):ti,ab,kw OR (Fatty Acid, Saturated):ti,ab,kw<br>#3 #1 OR #2<br>#4 MeSH descriptor: [Inflammatory Bowel Disease] explode all trees<br>#5 (Inflammatory Bowel Disease):ti,ab,kw OR (Bowel Diseases, Inflammatory):ti,ab,kw OR (IBD):ti,ab,kw OR (ulcer colitis):ti,ab,kw OR (colitis ulcer):ti,ab,kw OR (morbus crohn):ti,ab,kw OR (crohn's disease):ti,ab,kw OR (crohn disease):ti,ab,kw<br>#6 #4 OR #5<br>#7 #3 AND #6	Acid':ti,ab,kw OR 'Acid, Esterified Fatty':ti,ab,kw OR 'Fatty Acid, Esterified':ti,ab,kw OR 'Fatty Acids, Saturated':ti,ab,kw OR 'Saturated Fatty Acids':ti,ab,kw OR 'Saturated Fatty Acid':ti,ab,kw OR 'Acid, Saturated Fatty':ti,ab,kw OR 'Fatty Acid, Saturated':ti,ab,kw<br>#3 #1 OR #2<br>#4 'Inflammatory Bowel Disease'/exp OR 'Inflammatory Bowel Disease'<br>#5 'Inflammatory Bowel Disease':ti,ab,kw OR 'Bowel Diseases, Inflammatory':ti,ab,kw OR 'IBD':ti,ab,kw OR 'ulcer colitis':ti,ab,kw OR 'colitis ulcer':ti,ab,kw OR 'morbus crohn':ti,ab,kw OR 'crohn disease':ti,ab,kw<br>#6 #4 OR #5<br>#7 #3 AND #6<br><br>N=1605

	N=4006		N=257	
Monounsaturated Fatty Acid	#1 Fatty Acids, Monounsaturated [All Fields] OR Acids, Monounsaturated Fatty [All Fields] OR Monounsaturated Fatty Acids [All Fields] OR Monounsaturated Fatty Acid [All Fields] OR Acid, Monounsaturated Fatty [All Fields] OR Fatty Acid, Monounsaturated[All Fields] OR MUFAs [All Fields] OR MUFA [All Fields]	#1 TS=Fatty Acids, Monounsaturated OR Acids, Monounsaturated Fatty OR Monounsaturated Fatty Acids OR Monounsaturated Fatty Acid OR Acid, Monounsaturated Fatty OR Fatty Acid, Monounsaturated OR MUFAs OR MUFA<br>#2 TS=Inflamm	#1 MeSH descriptor:[Fatty Acids, Monounsaturated] explode all trees<br>#2 (Fatty Acids, Monounsaturated):ti,ab,kw OR (Acids, Monounsaturated Fatty):ti,ab,kw OR (Monounsaturated Fatty Acids):ti,ab,kw OR (Monounsaturated Fatty Acid):ti,ab,kw OR (Acid, Monounsaturated Fatty):ti,ab,kw OR (Fatty Acid, Monounsaturated):ti,ab,kw OR (MUFAs):ti,ab,kw OR (MUFA):ti,ab,kw	#1 'Fatty Acids, Monounsaturated'/exp OR 'Fatty Acids, Monounsaturated'<br>#2 'Fatty Acids, Monounsaturated':ti,ab,kw OR 'Acids, Monounsaturated Fatty':ti,ab,kw OR 'Monounsaturated Fatty Acids':ti,ab,kw OR 'Monounsaturated Fatty Acid':ti,ab,kw OR 'Acid, Monounsaturated Fatty':ti,ab,kw OR 'Fatty Acid, Monounsaturated':ti,ab,kw OR 'MUFAs':ti,ab,kw OR 'MUFA':ti,ab,kw<br>#3 #1 OR #2

	#2 Inflammatory Bowel Disease [All Fields] OR Bowel Diseases, Inflammatory [All Fields] OR IBD [All Fields] OR ulcer colitis [All Fields] OR colitis ulcer [All Fields] OR morbus crohn [All Fields] OR crohn's disease [All Fields] OR crohn disease [All Fields]<br>#3 #1 AND #2<br><br>N=165	atory Bowel Disease OR IBD OR ulcer colitis OR colitis ulcer OR morbus crohn OR crohn's disease OR crohn disease<br>#3 #1 AND #2<br><br>N=107	#3 #1 OR #2<br>#4 MeSH descriptor: [Inflammatory Bowel Disease] explode all trees<br>#5 (Inflammatory Bowel Disease):ti,ab,kw OR (Bowel Diseases, Inflammatory):ti,ab ,kw OR (IBD):ti,ab,kw OR (ulcer colitis):ti,ab,kw OR (colitis ulcer):ti,ab,kw OR (morbus crohn):ti,ab,kw OR (crohn's disease):ti,ab,kw OR (crohn disease):ti,ab,kw<br>#6 #4 OR #5<br>#7 #3 AND #6<br><br>N=12	#4 'Inflammatory Bowel Disease'/exp OR 'Inflammatory Bowel Disease'<br>#5 'Inflammatory Bowel Disease':ti,ab,kw OR 'Bowel Diseases, Inflammatory':ti,ab ,kw OR 'IBD':ti,ab,kw OR 'ulcer colitis':ti,ab,kw OR 'colitis ulcer':ti,ab,kw OR 'morbus crohn':ti,ab,kw OR 'crohn disease':ti,ab,kw<br>#6 #4 OR #5<br>#7 #3 AND #6<br><br>N=137

Red Meat and Processed Meat	#1 Red Meat [All Fields] OR Unprocessed red meats [All Fields] OR Meat, Red [All Fields] OR Meats, Red[All Fields] OR Red Meats [All Fields] OR Beef [All Fields] OR Lamb Meat [All Fields] OR Lamb Meats [All Fields] OR Meat, Lamb [All Fields] OR Meats, Lamb [All Fields] OR Veal [All Fields] OR Total processed meats [All Fields] OR Meat Product [All Fields] OR Product, Meat [All Fields] OR Products, Meat [All Fields]<br>#2 Inflammatory Bowel Disease [All Fields] OR Bowel Diseases, Inflammatory [All Fields] OR IBD [All Fields] OR ulcer colitis [All Fields] OR	#1 TS=Red Meat OR Unprocessed red meats OR Meat, Red OR Meats, Red OR Red Meats OR Beef OR Lamb Meat OR Lamb Meats OR Meat, Lamb OR Meats, Lamb OR Veal OR Total processed meats OR Meat Product OR Product, Meat OR Products, Meat<br>#2 TS=Inflammatory Bowel Disease OR IBD OR ulcer colitis OR colitis ulcer OR morbus crohn OR crohn's disease OR crohn disease<br>#3 #1 AND #2<br><br>N=523	#1 MeSH descriptor:[Red Meat] explode all trees<br>#2 (Red Meat):ti,ab,kw OR (Unprocessed red meats):ti,ab,kw OR (Meat, Red):ti,ab,kw OR (Meats, Red):ti,ab,kw OR (Red Meats):ti,ab,kw OR (Beef):ti,ab,kw OR (Lamb Meat):ti,ab,kw OR (Lamb Meats):ti,ab,kw OR (Meat, Lamb):ti,ab,kw OR (Meats, Lamb):ti,ab,kw OR (Veal):ti,ab,kw OR (Total processed meats):ti,ab,kw OR (processed meat):ti,ab,kw OR (Meat Product):ti,ab,kw OR (Product, Meat):ti,ab,kw OR (Products, Meat):ti,ab,kw<br>#3 #1 OR #2<br>#4 MeSH descriptor: [Inflammatory Bowel Disease] explode all trees<br>#5 (Inflammatory Bowel Disease):ti,ab,kw OR (Bowel	#1 'red meat'/exp OR 'red meat'<br>#2 'processed meat'/exp OR 'processed meat'<br>#3 'Red Meat':ti,ab,kw OR 'Unprocessed red meats':ti,ab,kw OR 'Meat, Red':ti,ab,kw OR 'Meats, Red':ti,ab,kw OR 'Red Meats':ti,ab,kw OR 'Beef':ti,ab,kw OR 'Lamb Meat':ti,ab,kw OR 'Lamb Meats':ti,ab,kw OR 'Meat, Lamb':ti,ab,kw OR 'Meats, Lamb':ti,ab,kw OR 'Veal':ti,ab,kw OR 'Total processed meats':ti,ab,kw OR 'processed meat':ti,ab,kw OR 'Meat Product':ti,ab,kw OR 'Product, Meat':ti,ab,kw OR 'Products, Meat':ti,ab,kw<br>#4 #1 OR #2 OR #3<br>#5 'Inflammatory Bowel Disease'/exp OR 'Inflammatory Bowel Disease'<br>#6 'Inflammatory Bowel Disease':ti,ab,kw

	colitis ulcer [All Fields] OR morbus crohn [All Fields] OR crohn's disease [All Fields] OR crohn disease [All Fields]<br>#3 #1 AND #2<br><br>N=187		Diseases, Inflammatory):ti,ab ,kw OR (IBD):ti,ab,kw OR (ulcer colitis):ti,ab,kw OR (colitis ulcer):ti,ab,kw OR (morbus crohn):ti,ab,kw OR (crohn's disease):ti,ab,kw OR (crohn disease):ti,ab,kw<br>#6 #4 OR #5<br>#7 #3 AND #6<br><br>N=27	OR 'Bowel Diseases, Inflammatory':ti,ab ,kw OR 'IBD':ti,ab,kw OR 'ulcer colitis':ti,ab,kw OR 'colitis ulcer':ti,ab,kw OR 'morbus crohn':ti,ab,kw OR 'crohn disease':ti,ab,kw<br>#7 #5 OR #6<br>#8 #4 AND #7<br><br>N=339

Omega-6 Fatty Acid	#1 Omega 6 Fatty Acid [All Fields] OR Omega 6 Fatty Acids [All Fields] OR Omega-6 Fatty Acids [All Fields] OR Fatty Acids, Omega-6 [All Fields] OR Acids, Omega-6 Fatty [All Fields] OR Fatty Acids, Omega 6 [All Fields] OR N-6 Fatty Acids [All Fields] OR Acids, N-6 Fatty [All Fields] OR Fatty Acids, N-6 [All Fields] OR N 6 Fatty Acids [All Fields] OR Omega-6 Fatty Acids [All Fields] OR Omega 6 Fatty Acids [All Fields] OR N-6 Fatty Acid [All Fields] OR Acid, N-6 Fatty [All Fields] OR Fatty Acid, N-6 [All Fields] OR N 6 Fatty Acid [All Fields] OR Omega-6 Fatty Acid [All	#1 TS=Omega 6 Fatty Acid OR Omega 6 Fatty Acids OR Omega-6 Fatty Acids OR Fatty Acids, Omega-6 OR Acids, Omega-6 Fatty OR Fatty Acids, Omega 6 OR N-6 Fatty Acids OR Acids, N-6 Fatty OR Fatty Acids, N-6 OR N 6 Fatty Acids OR Omega-6 Fatty Acids OR Omega 6 Fatty Acids OR N-6 Fatty Acid OR Acid, N-6 Fatty OR Fatty Acid, N-6 OR N 6 Fatty Acid OR Omega-6 Fatty Acid OR Acid, Omega-6 Fatty OR Fatty Acid, Omega-6 OR Omega 6 Fatty Acid<br>#2 TS=Inflamm	#1 [Fatty Acids, Omega-6] explode all trees<br>#2 (Omega 6 Fatty Acid):ti,ab,kw OR (Omega 6 Fatty Acids):ti,ab,kw OR (Omega-6 Fatty Acids):ti,ab,kw OR (Fatty Acids, Omega-6):ti,ab,kw OR (Acids, Omega-6 Fatty):ti,ab,kw OR (Fatty Acids, Omega 6):ti,ab,kw OR (N-6 Fatty Acids):ti,ab,kw OR (Acids, N-6 Fatty):ti,ab,kw OR (Fatty Acids, N-6):ti,ab,kw OR (N 6 Fatty Acids):ti,ab,kw OR (Omega-6 Fatty Acids):ti,ab,kw OR (Omega 6 Fatty Acids):ti,ab,kw OR (N-6 Fatty Acid):ti,ab,kw OR (Acid, N-6 Fatty):ti,ab,kw OR (Fatty Acid, N-6):ti,ab,kw OR (N 6 Fatty Acid):ti,ab,kw OR (Omega-6 Fatty Acid):ti,ab,kw OR (Acid, Omega-6 Fatty):ti,ab,kw OR (Fatty Acid, Omega-6):ti,ab,kw OR (Omega 6 Fatty Acid):ti,ab,kw	#1 'omega 6 fatty acid'/exp OR 'omega 6 fatty acid'<br>#2 'Omega 6 Fatty Acid':ti,ab,kw OR 'Omega 6 Fatty Acids':ti,ab,kw OR 'Omega-6 Fatty Acids':ti,ab,kw OR 'Fatty Acids, Omega-6':ti,ab,kw OR 'Acids, Omega-6 Fatty':ti,ab,kw OR 'Fatty Acids, Omega 6':ti,ab,kw OR 'N-6 Fatty Acids':ti,ab,kw OR 'Acids, N-6 Fatty':ti,ab,kw OR 'Fatty Acids, N-6':ti,ab,kw OR 'N 6 Fatty Acids':ti,ab,kw OR 'Omega-6 Fatty Acids':ti,ab,kw OR 'Omega 6 Fatty Acids':ti,ab,kw OR 'N-6 Fatty Acid':ti,ab,kw OR 'Acid, N-6 Fatty':ti,ab,kw OR 'Fatty Acid, N-6':ti,ab,kw OR 'N 6 Fatty Acid':ti,ab,kw OR 'Omega-6 Fatty Acid':ti,ab,kw OR 'Acid, Omega-6 Fatty':ti,ab,kw OR 'Fatty Acid, Omega-6':ti,ab,kw OR 'Omega 6 Fatty Acid':ti,ab,kw<br>#3 #1 OR #2<br>#4 'Inflammatory

	Fields] OR Acid, Omega-6 Fatty [All Fields] OR Fatty Acid, Omega-6 [All Fields] OR Omega 6 Fatty Acid [All Fields]<br>#2 Inflammatory Bowel Disease [All Fields] OR Bowel Diseases, Inflammatory [All Fields] OR IBD [All Fields] OR ulcer colitis [All Fields] OR colitis ulcer [All Fields] OR morbus crohn [All Fields] OR crohn's disease [All Fields] OR crohn disease [All Fields]<br>#3 #1 AND #2<br><br>N=162	atory Bowel Disease OR IBD OR ulcer colitis OR colitis ulcer OR morbus crohn OR crohn's disease OR crohn disease<br>#3 #1 AND #2<br><br>N=745	#3 #1 OR #2<br>#4 MeSH descriptor: [Inflammatory Bowel Disease] explode all trees<br>#5 (Inflammatory Bowel Disease):ti,ab,kw OR (Bowel Diseases, Inflammatory):ti,ab,kw OR (IBD):ti,ab,kw OR (ulcer colitis):ti,ab,kw OR (colitis ulcer):ti,ab,kw OR (morbus crohn):ti,ab,kw OR (crohn's disease):ti,ab,kw OR (crohn disease):ti,ab,kw<br>#6 #4 OR #5<br>#7 #3 AND #6<br><br>N=77	Bowel Disease'/exp OR 'Inflammatory Bowel Disease'<br>#5 'Inflammatory Bowel Disease':ti,ab,kw OR 'Bowel Diseases, Inflammatory':ti,ab,kw OR 'IBD':ti,ab,kw OR 'ulcer colitis':ti,ab,kw OR 'colitis ulcer':ti,ab,kw OR 'morbus crohn':ti,ab,kw OR 'crohn disease':ti,ab,kw<br>#6 #4 OR #5<br>#7 #3 AND #6<br><br>N=248

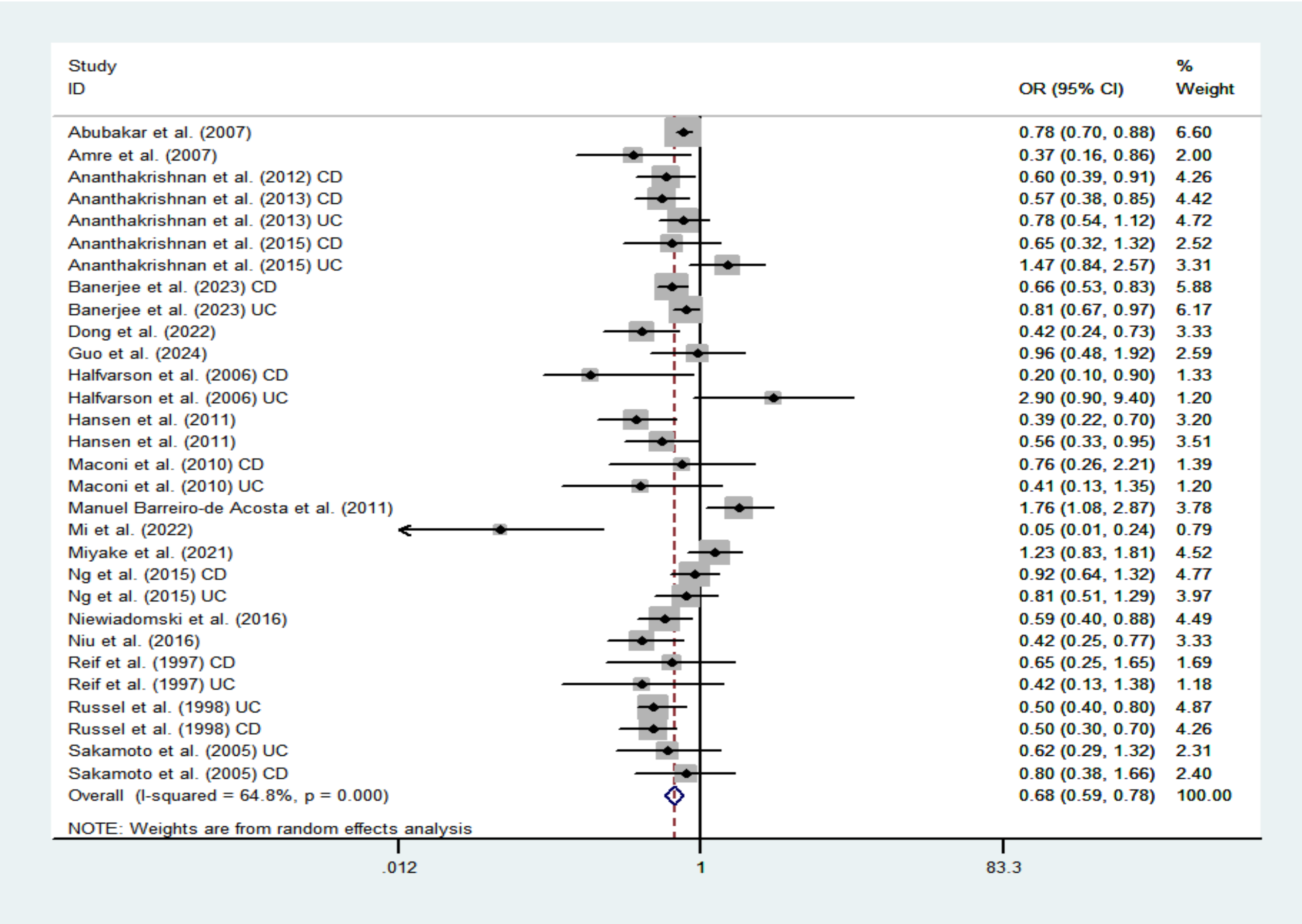

Supplementary Figure 1: Forest plot of the association between Fruit consumption and the risk of IBD using a random-effects model.

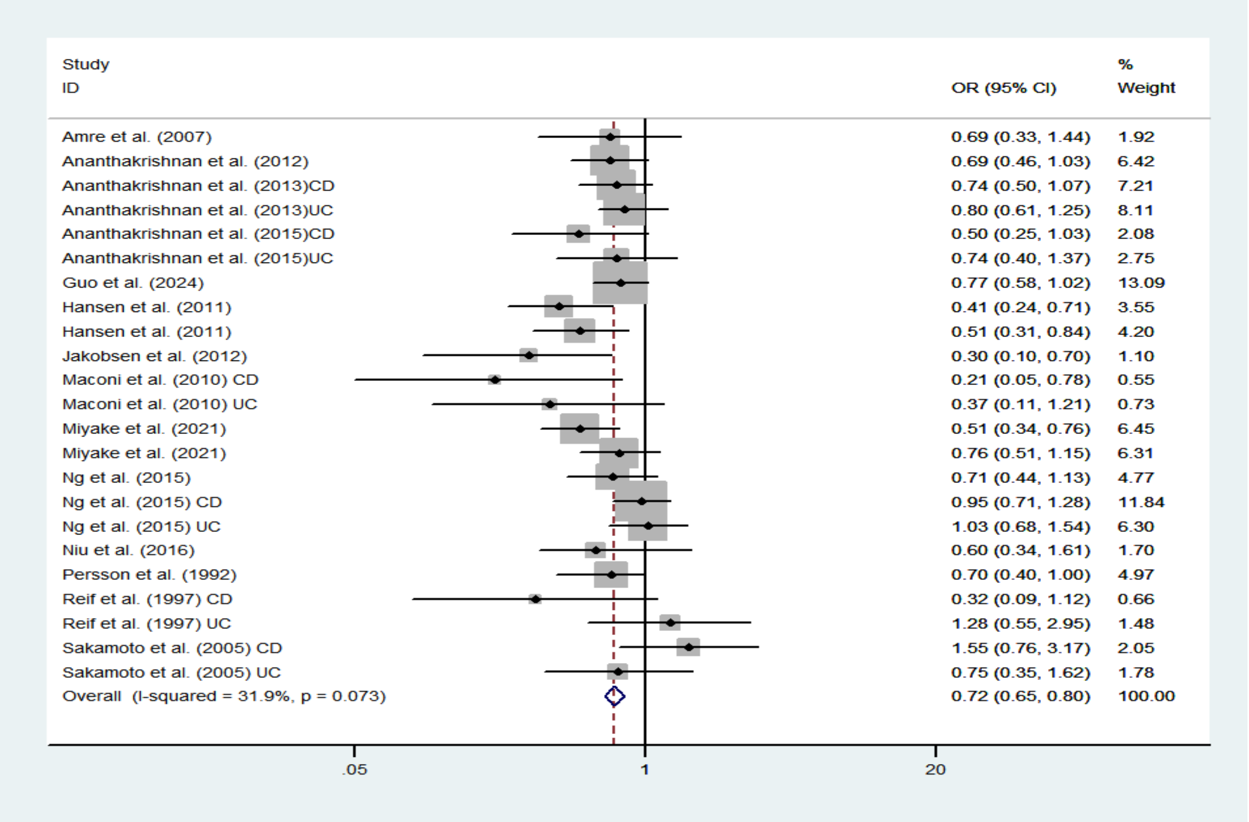

Supplementary Figure 2: Forest plot of the association between Vegetables consumption and the risk of IBD using a fixed-effects model.

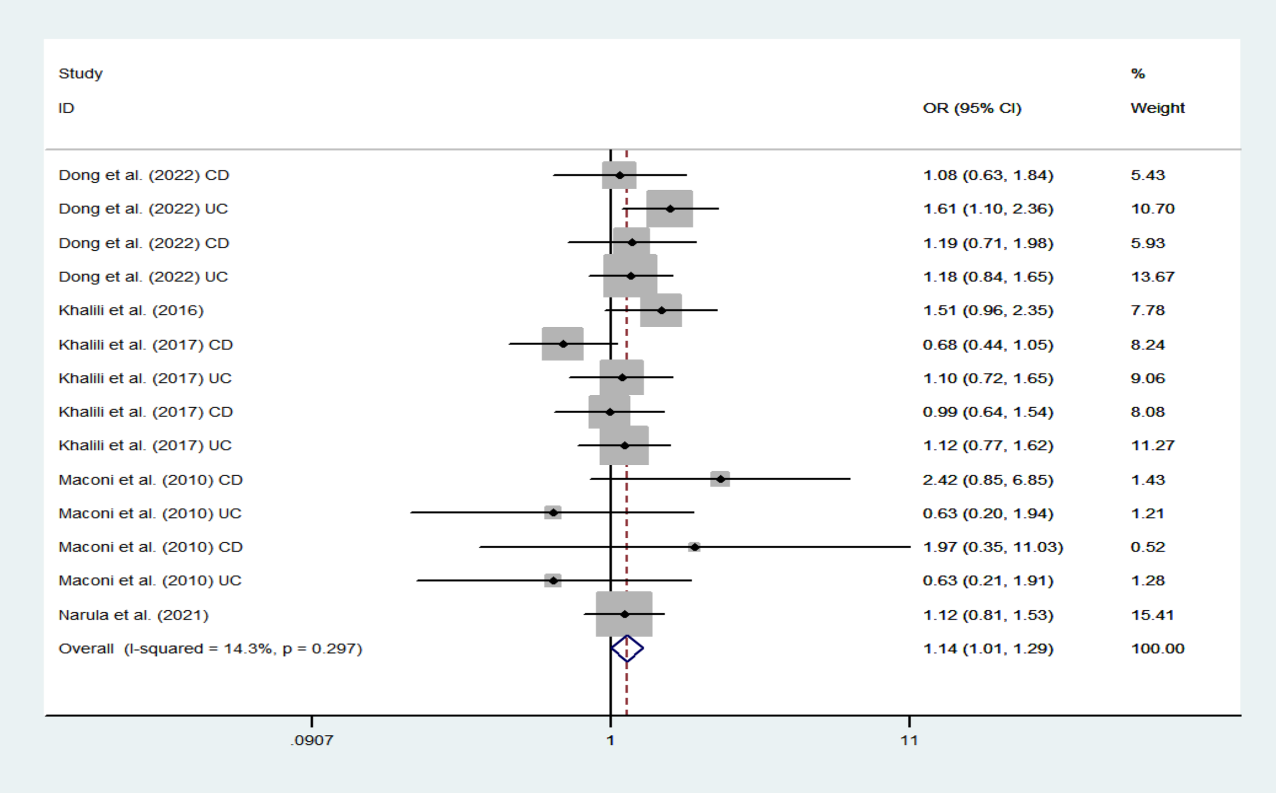

Supplementary Figure 3: Forest plot of the association between Total processed meats and Unprocessed red meats consumption and the risk of IBD using a fixed-effects model.

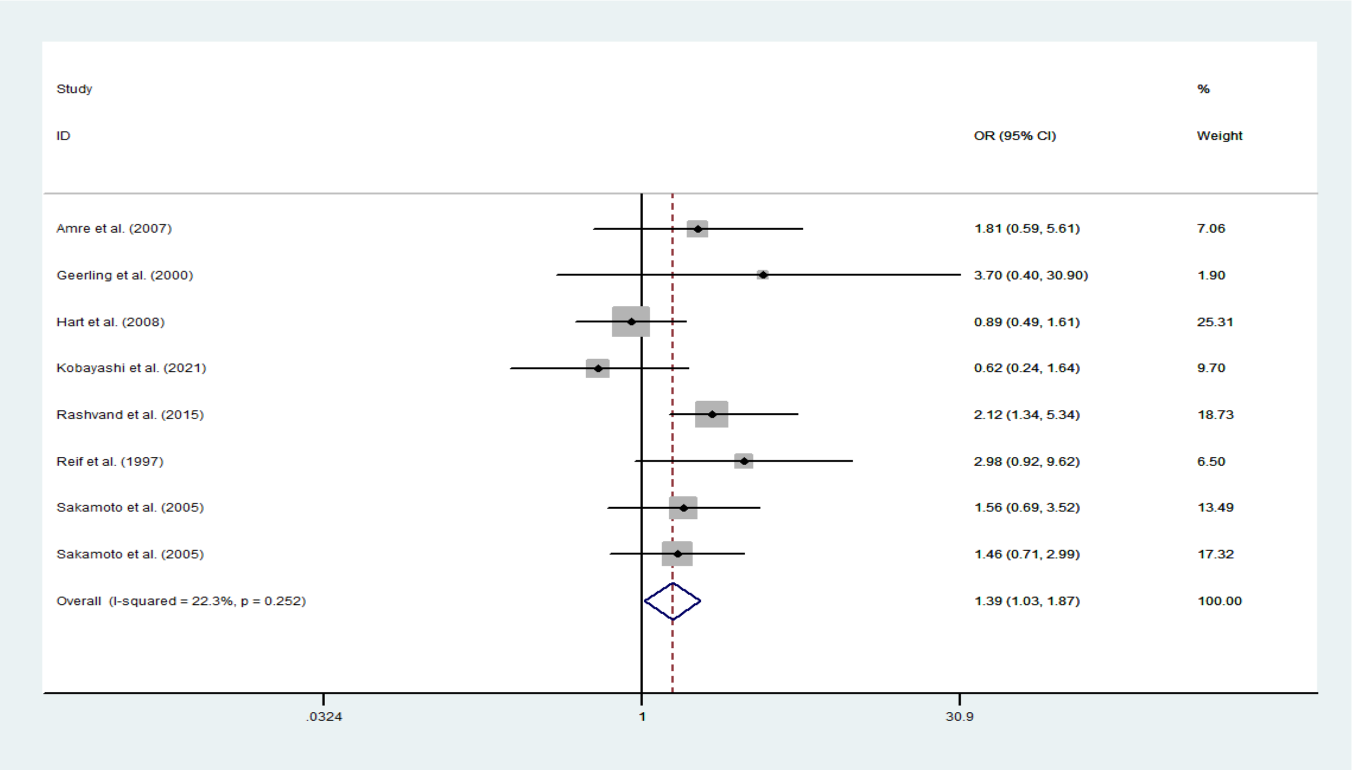

Supplementary Figure 4: Forest plot of the association between Saturated fat consumption and the risk of IBD using a fixed-effects model.

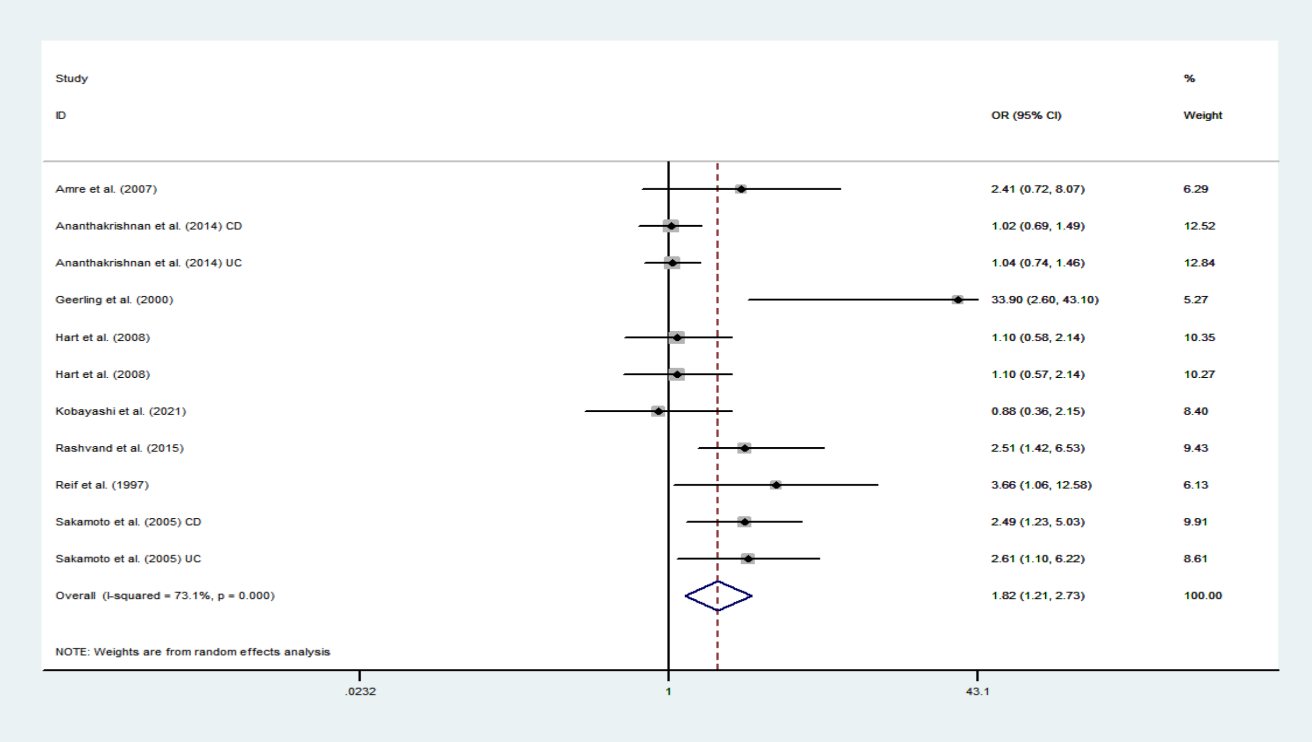

Supplementary Figure 5: Forest plot of the association between Monounsaturated fatty acids consumption and the risk of IBD using a fixed-effects model.

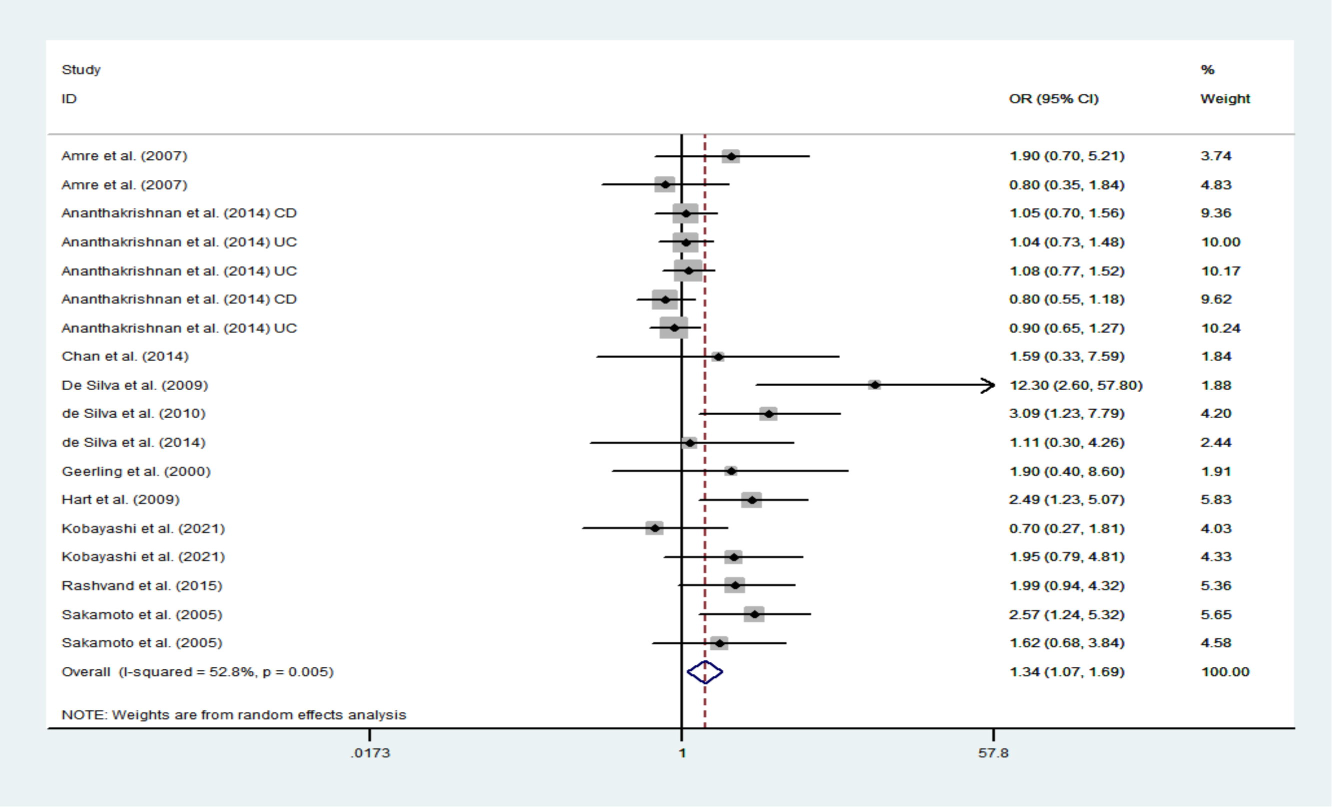

Supplementary Figure 6: Forest plot of the association between Omega-6 consumption and the risk of IBD using a random-effects model.

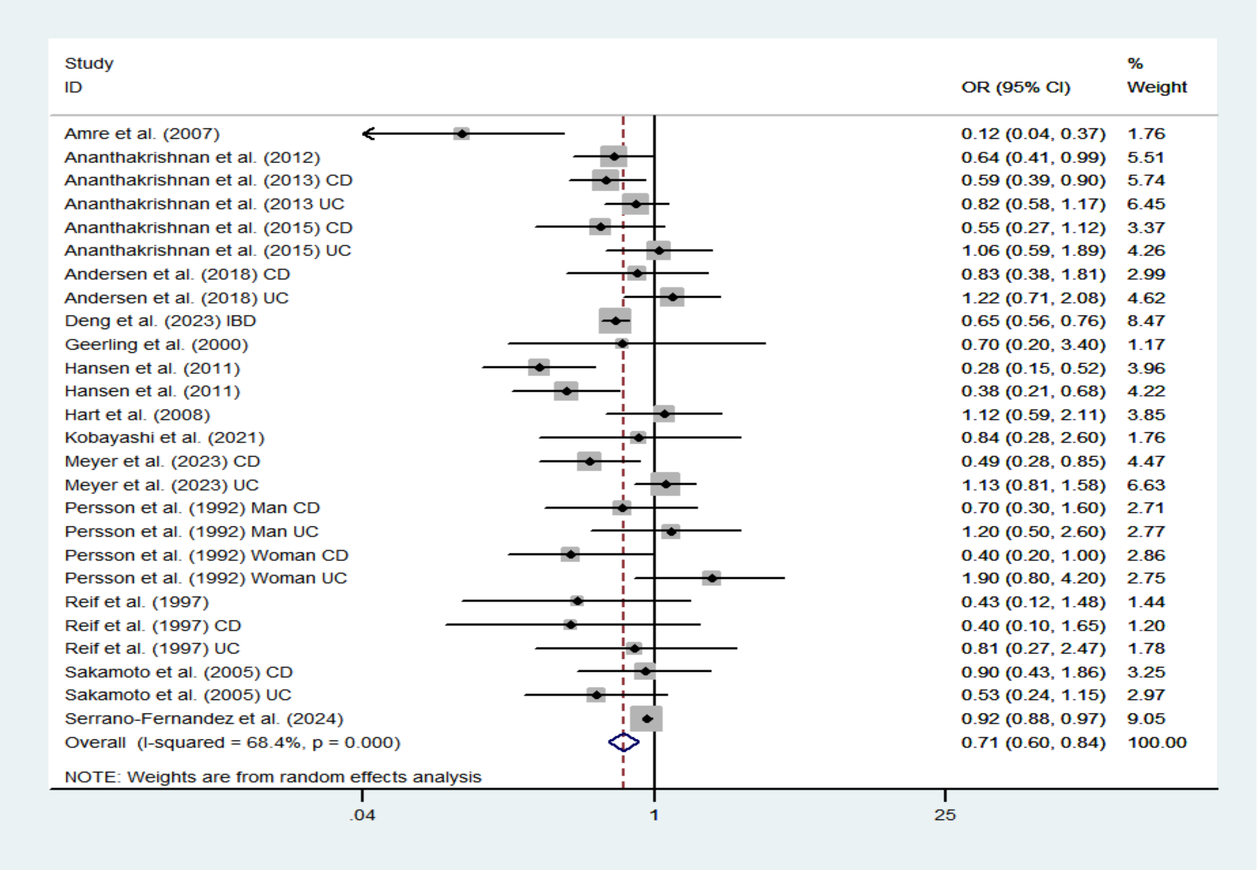

Supplementary Figure 7: Forest plot of the association between Dietary Fiber consumption and the risk of IBD using a random-effects model.

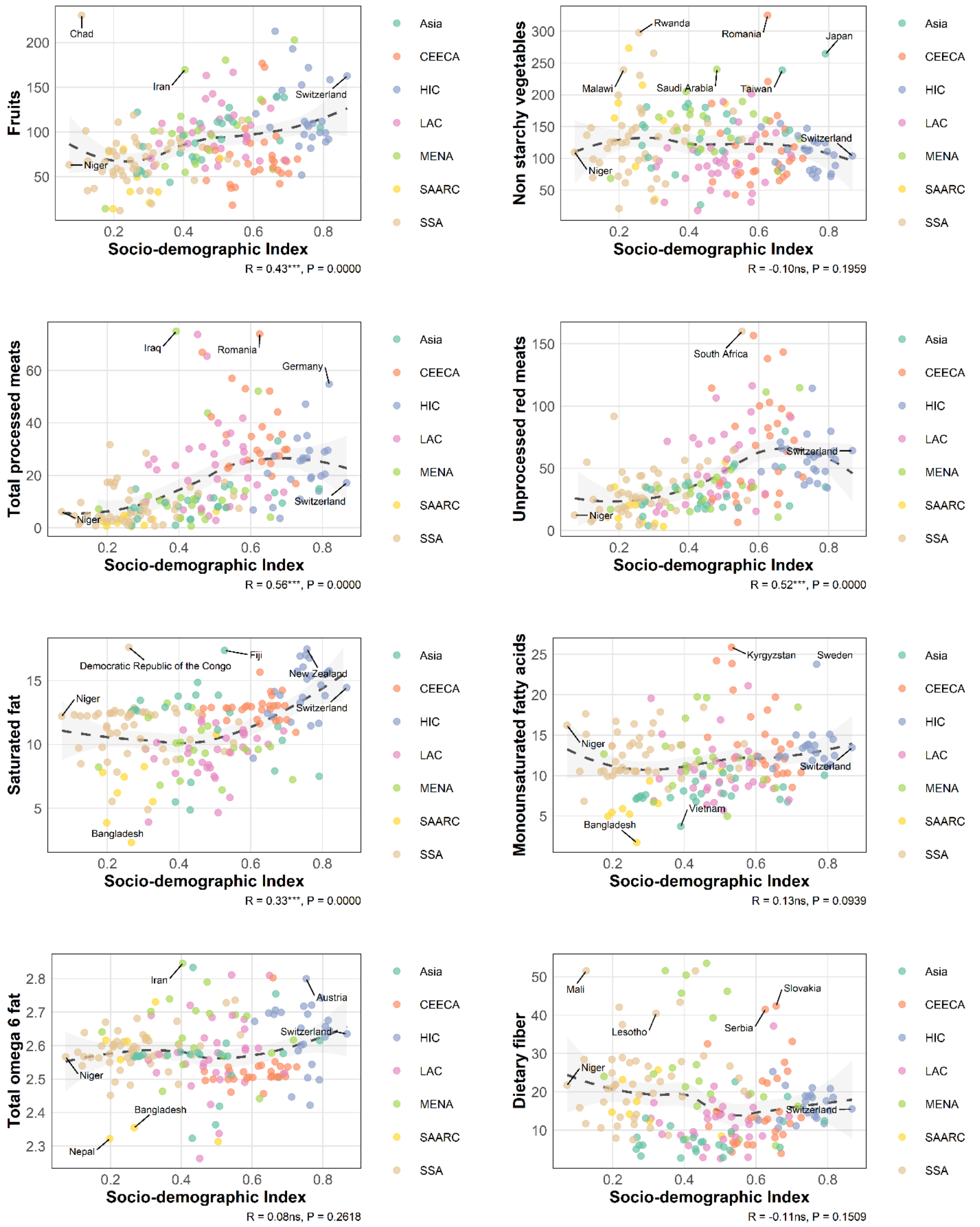

Supplementary Figure 8: Spearman correlation was assessed in this analysis between SDI and the intakes of 8 dietary components among a total of 185 countries (1990).

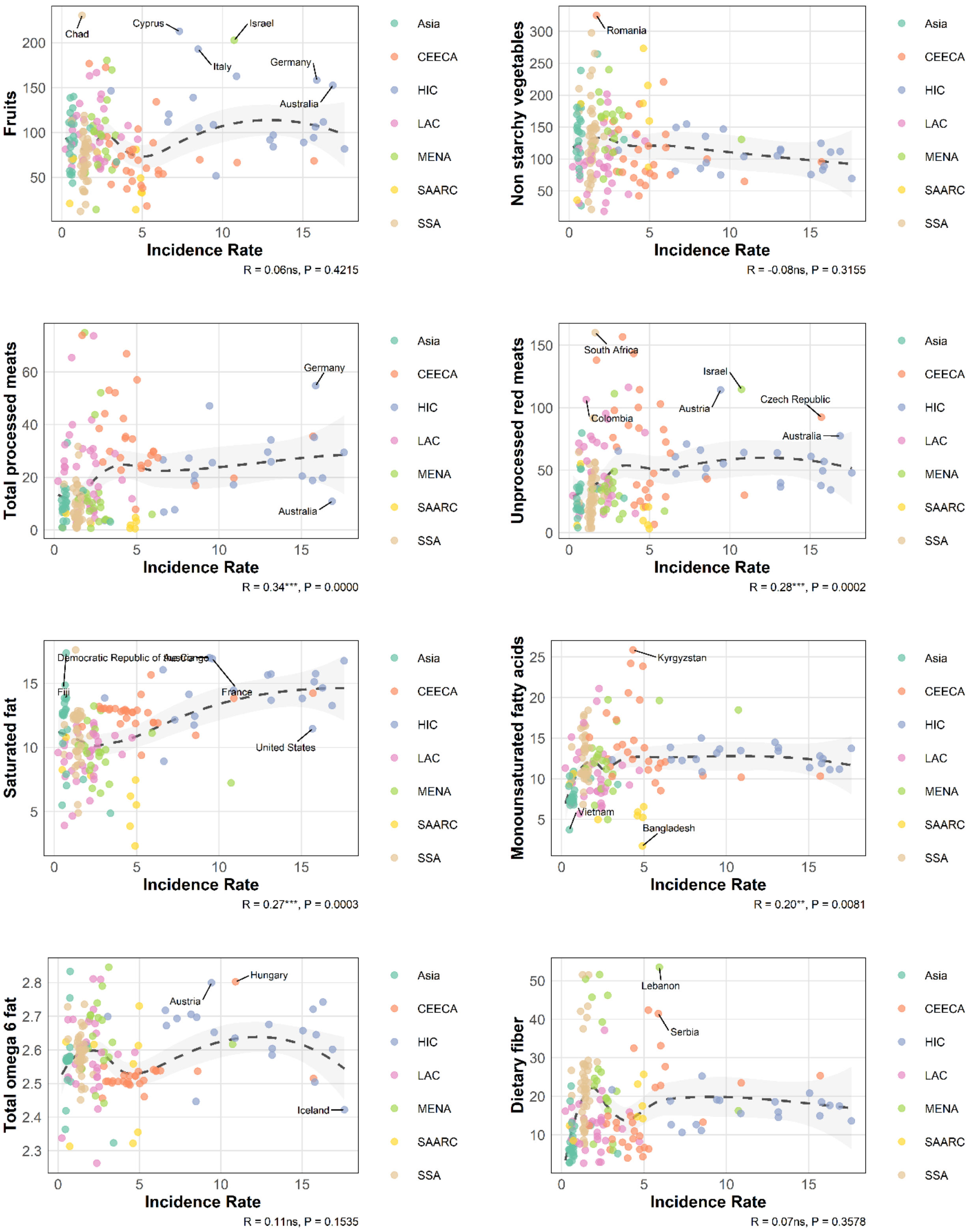

Supplementary Figure 9: In this analysis, the Spearman correlation was assessed between the IBD incidence rate and the intake of 8 dietary components across 185 countries. (1990)

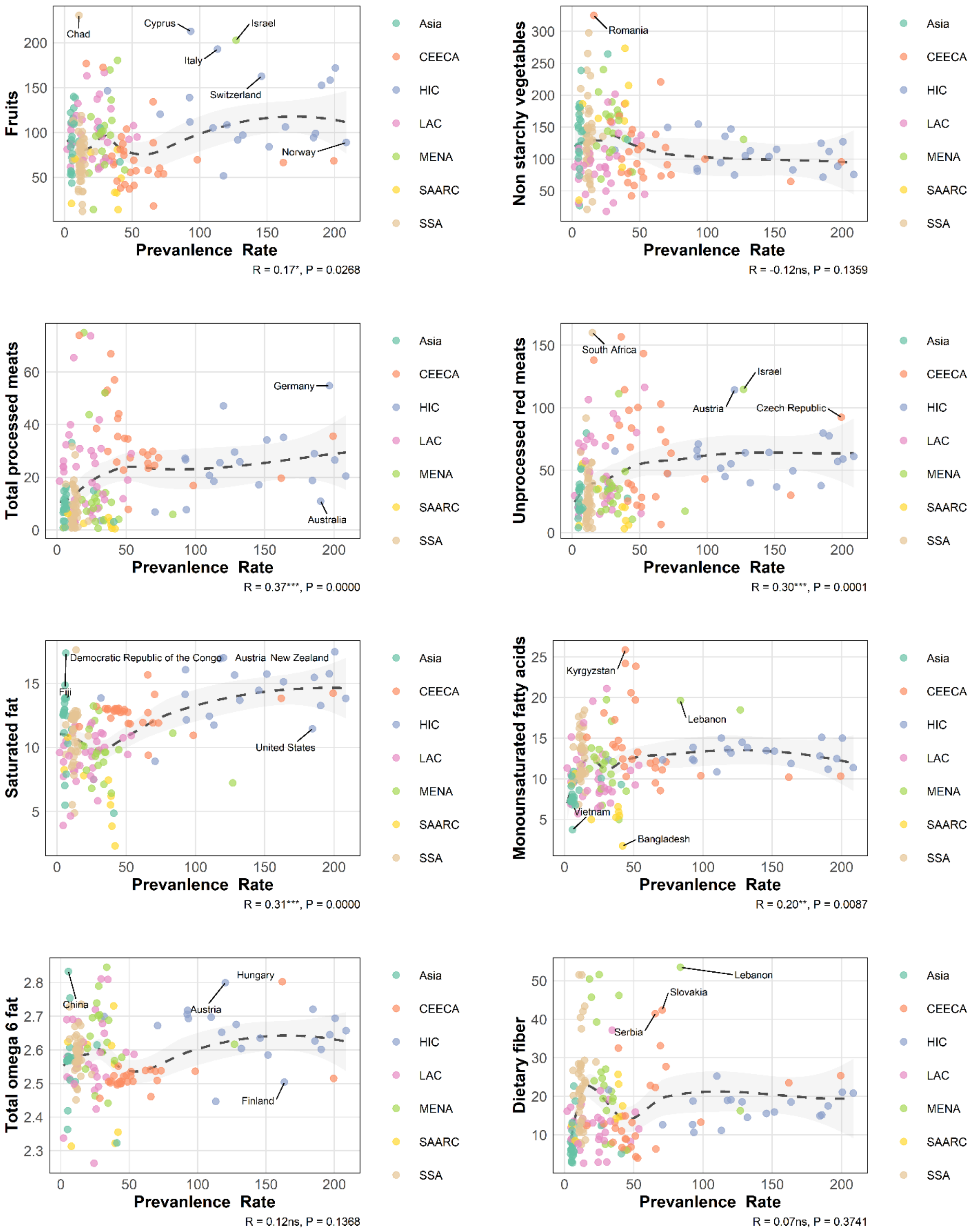

Supplementary Figure 10: In this analysis, the Spearman correlation was assessed between the IBD prevalence rate and the intake of 8 dietary components across 185 countries. (1990)

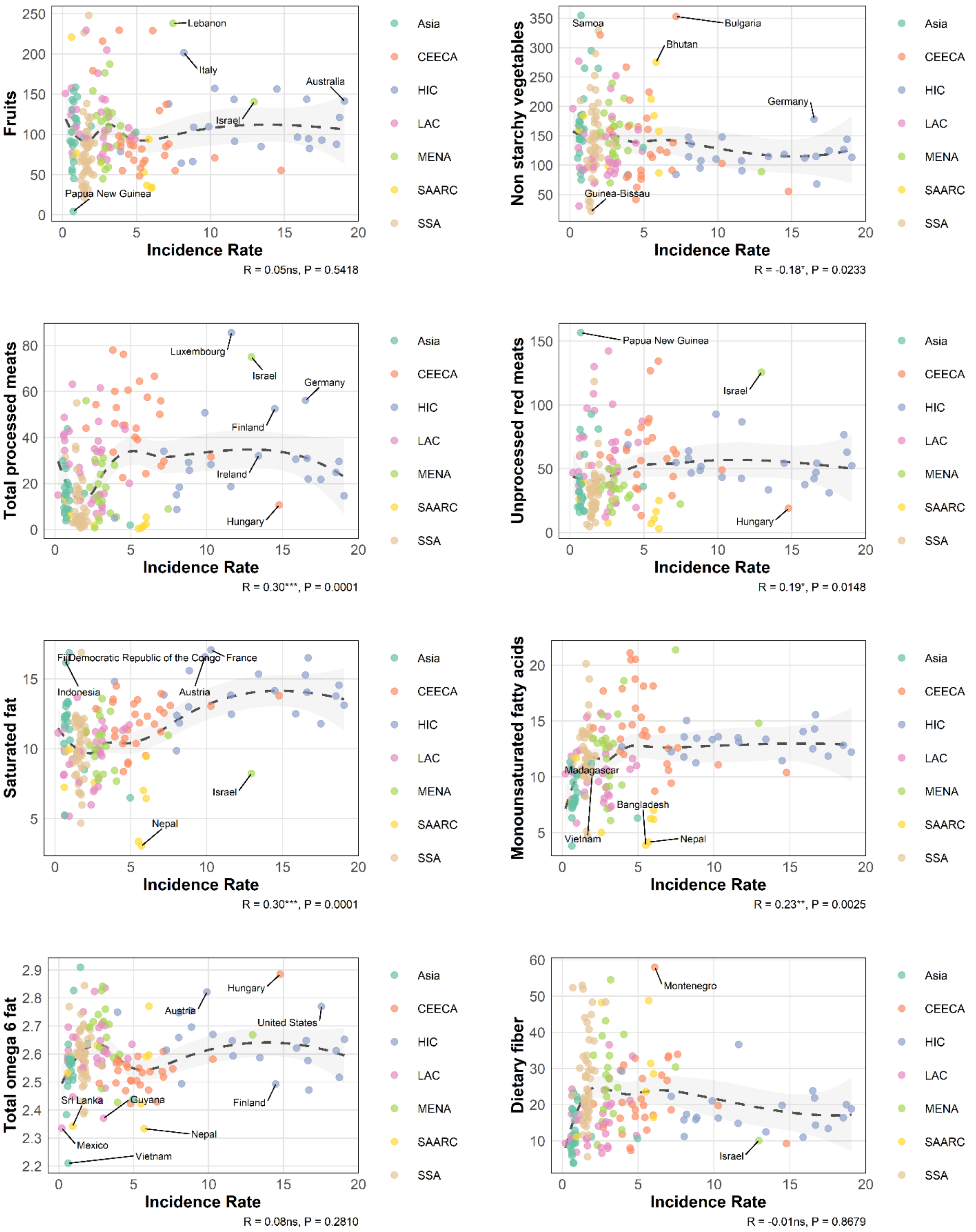

Supplementary Figure 11: In this analysis, the Spearman correlation was assessed between the IBD incidence rate and the intake of 8 dietary components across 185 countries. (2018)

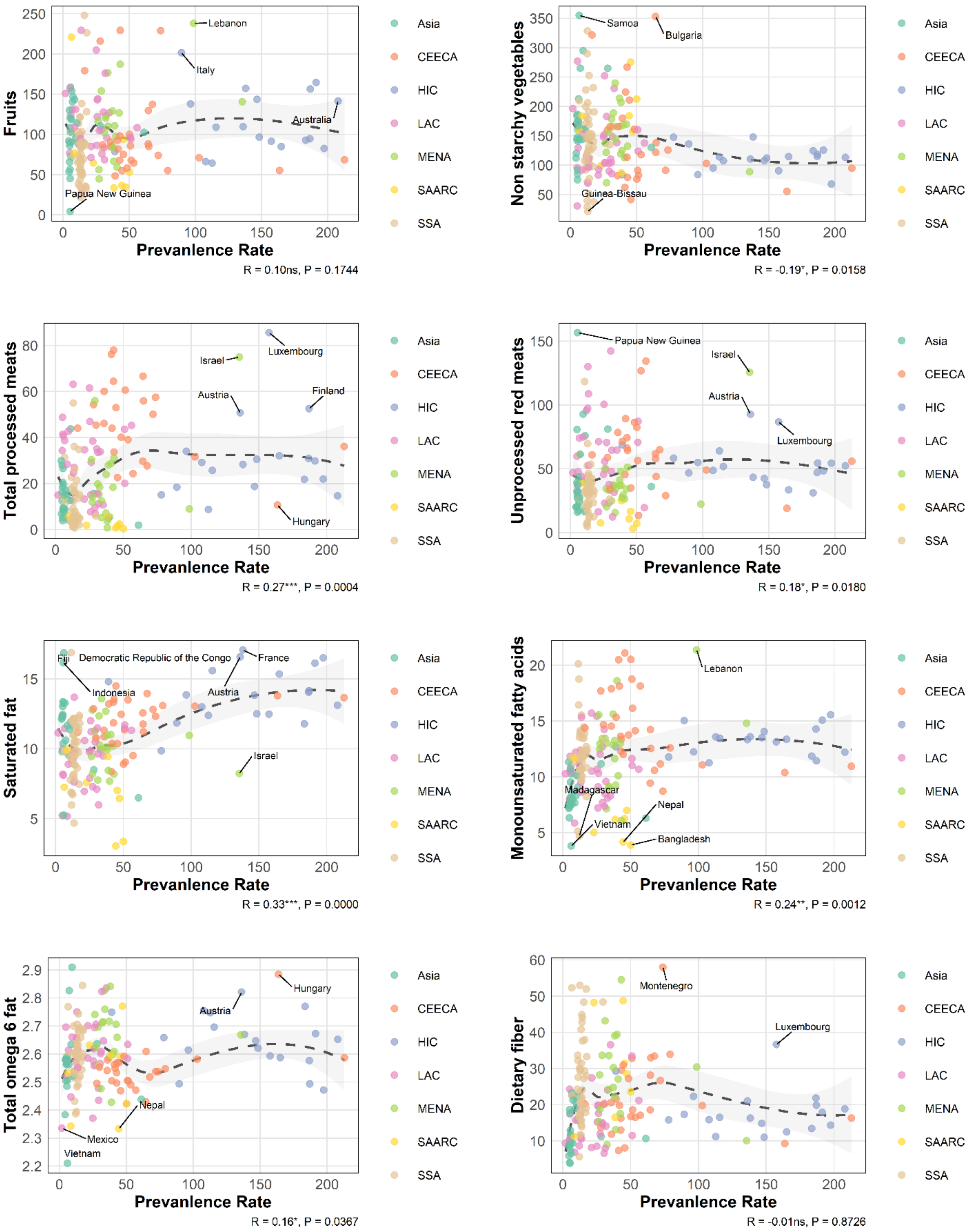

Supplementary Figure 12: In this analysis, the Spearman correlation was assessed between the IBD prevalence rate and the intake of 8 dietary components across 185 countries. (2018)
